# A spruce gene map infers ancient plant genome reshuffling and subsequent slow evolution in the gymnosperm lineage leading to extant conifers

**DOI:** 10.1186/1741-7007-10-84

**Published:** 2012-10-26

**Authors:** Nathalie Pavy, Betty Pelgas, Jérôme Laroche, Philippe Rigault, Nathalie Isabel, Jean Bousquet

**Affiliations:** 1 Canada Research Chair in Forest and Environmental Genomics, Centre for Forest Research and Institute for Systems and Integrative Biology, Université Laval, Québec, Québec G1V 0A6, Canada; 2 Natural Resources Canada, Canadian Forest Service, Laurentian Forestry Centre, 1055 Rue du PEPS, CP 10380, Succ. Sainte-Foy, Québec, Québec G1V 4C7, Canada; 3 Bioinformatics Platform, Institute for Systems and Integrative Biology, Université Laval, Québec, Québec G1V 0A6, Canada; 4 Gydle Inc., 1363 Avenue Maguire, Québec, Québec G1T 1Z2, Canada

**Keywords:** Angiosperm, duplication, evolution, gene families, genetic map, gymnosperm, phylogenomics, *Picea*, spruce, structural genomics

## Abstract

**Background:**

Seed plants are composed of angiosperms and gymnosperms, which diverged from each other around 300 million years ago. While much light has been shed on the mechanisms and rate of genome evolution in flowering plants, such knowledge remains conspicuously meagre for the gymnosperms. Conifers are key representatives of gymnosperms and the sheer size of their genomes represents a significant challenge for characterization, sequencing and assembling.

**Results:**

To gain insight into the macro-organisation and long-term evolution of the conifer genome, we developed a genetic map involving 1,801 spruce genes. We designed a statistical approach based on kernel density estimation to analyse gene density and identified seven gene-rich isochors. Groups of co-localizing genes were also found that were transcriptionally co-regulated, indicative of functional clusters. Phylogenetic analyses of 157 gene families for which at least two duplicates were mapped on the spruce genome indicated that ancient gene duplicates shared by angiosperms and gymnosperms outnumbered conifer-specific duplicates by a ratio of eight to one. Ancient duplicates were much more translocated within and among spruce chromosomes than conifer-specific duplicates, which were mostly organised in tandem arrays. Both high synteny and collinearity were also observed between the genomes of spruce and pine, two conifers that diverged more than 100 million years ago.

**Conclusions:**

Taken together, these results indicate that much genomic evolution has occurred in the seed plant lineage before the split between gymnosperms and angiosperms, and that the pace of evolution of the genome macro-structure has been much slower in the gymnosperm lineage leading to extent conifers than that seen for the same period of time in flowering plants. This trend is largely congruent with the contrasted rates of diversification and morphological evolution observed between these two groups of seed plants.

## Background

Gene duplication plays an important role in providing raw material to evolution [1]. In plants, gene duplicates arise through diverse molecular mechanisms, ranging from whole-genome duplication to more restricted duplications of smaller chromosomal regions [2]. The evolution of the flowering plant genomes has been intensively studied since the completion of the genome sequence for several angiosperm species. Lineage-specific whole-genome duplications greatly contributed to the expansion of plant genomes and gene families (for examples, see [3-9]), with whole-genome duplications found in basal angiosperms, monocots and eudicots [9-12].

Little is known about the large-scale evolutionary history of gene duplications for other seed plants, as well as before the origin of angiosperms. Spermatophytes encompass the angiosperms and the gymnosperms, whose seeds are not enclosed in an ovary. The two groups diverged around 300 million years ago (Mya) in the Late Carboniferous [13,14]. Contrary to angiosperms, which underwent massive adaptive radiation to supplant the gymnosperms as the dominant vascular plant group [15,16], extant gymnosperms are divided into a relatively small number of groups including the Pinophyta (conifers), Cycadophyta (cycads), Gnetophyta (gnetophytes) and Ginkgophyta (*Ginkgo*), and they contain about 1,000 species [17]. Polyploidy is rare in gymnosperms. Only 5% of them, and 1.5% of the subgroup conifers, have been reported as polyploid species [18,19], as indicated by cytological analysis [18], distributions of synonymous substitution rates [19,20] or phylogenetic analysis [20]. Nevertheless, the genomes of some gymnosperms, such as in the conifer family Pinaceae, are among the largest of all known organisms [21], with haploid genome sizes up to 37 Gb for *Pinus gerardiana *[22,23].

Several issues need to be addressed regarding the evolution of the seed plant genome, and that of the plant genome predating the gymnosperm-angiosperm (GA) divergence. How many gene duplications are shared between angiosperms and gymnosperms, which would predate their divergence and make them ancient? How frequently have gene duplications occurred solely in gymnosperms since their split from angiosperms? Are ancient duplicates, those preceding the GA split, relatively more abundant and more translocated through the gymnosperm genome than most recent duplicates specific to the gymnosperms?

These questions could be addressed through a phylogenomic approach, where the members of different gene families are mapped in a gymnosperm taxon with these families further sampled in completely sequenced angiosperm taxa to reconstruct their multiple phylogenies. Given that the gene complement of model angiosperms has been entirely determined by complete genome sequencing, but not that of a gymnosperm taxon, such gene phylogenies would give rise to mixed angiosperm-gymnosperm nodes and gymnosperm-specific nodes. With respect to the divergence time between pro-angiosperms and pro-gymnosperms (approximately 300 Mya), different grouping of gene duplicates could help determine the relative age of duplications, such that mixed angiosperm-gymnosperm nodes predating the split between angiosperms and gymnosperms would indicate ancient duplications, while gymnosperm-specific nodes postdating this split would indicate more recent duplications. The various proportions of these nodes over a large number of gene phylogenies would provide a glance at the relative frequency of ancient to recent gene duplications in the gymnosperm lineage, and the mapping of these duplicates on a gymnosperm genome would allow assessment of their possible translocation. Because of the incomplete nature of gene inventories in gymnosperms, such an analysis from the perspective of the angiosperm lineage is still not possible, given that nodes containing angiosperm duplicates only might not be truly angiosperm-specific. On a smaller scale, similar approaches have been applied to investigate the deep phylogenies of a few seed plant gene families completely sequenced in the conifers. They have indicated that, while some gene duplications deemed ancient predated the split between gymnosperms and angiosperms, some duplications have occurred more recently that are specific to the gymnosperm lineage (for example, [24]).

Based on such a phylogenetic approach together with gene mapping, one could also ask if the spread of gene families over the gymnosperm genome is more likely for ancient duplicates predating the GA split than for more recent duplicates postdating this split. Theoretical and empirical approaches have shown that duplicated regions should be translocated with time [9]. As such, one would expect the more recent gymnosperm-specific duplicates to be physically less spread across the genome than more ancient duplicates predating the GA split. Altogether, the relative age of gymnosperm-specific gene duplicates and their degree of translocation would allow an assessment of whether the conservation of genome macro-structure parallels the recognised archaic nature of gymnosperms in terms of morphology, the reproductive system and other phenotypic attributes [25]. Testing these hypotheses requires large catalogues of gene sequences, which have recently become available in conifers [26], and mapping of a large number of genes in a gymnosperm.

In this study, we assembled a map involving 1,801 spruce-expressed genes and examined the distribution of gene families onto the spruce genome and its level of conservation across Pinaceae and angiosperm genomes. We asked whether ancient gene duplicates shared with angiosperms are more numerous and more reshuffled than more recent duplicates occurring in the gymnosperm lineage leading to extant conifers. We also investigated how stable the genome macro-structure has been between the conifers *Picea *and *Pinus *since their divergence 120 to 140 Mya [13,14,27], a period of time corresponding to tremendous reshuffling of the angiosperm genome.

## Results

### Spruce gene map

**Figure 1:**
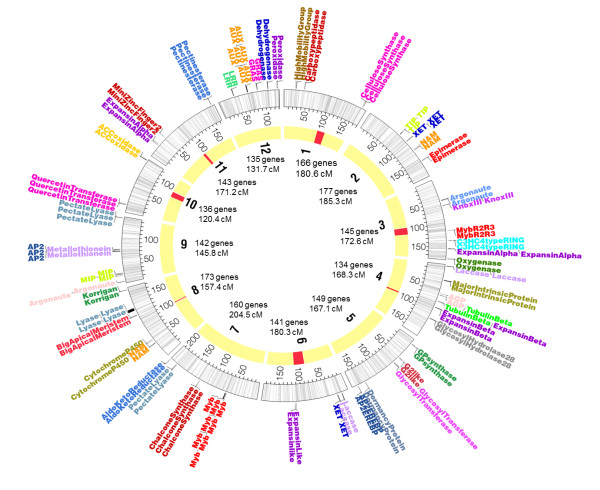
**Map of the spruce genome and tandemly arrayed genes**. The 12 spruce chromosomes were plotted with Circos [100]. From inside to outside: gene-rich regions in red; the 12 chromosomes with ticks representing the genes mapped along the spruce linkage groups, and with genetic distances in cM (Kosambi); distribution of the tandemly arrayed genes. The chromosome nomenclature and numbers of genes mapped are inside the circle. For the complete names of tandemly arrayed genes, see Additional file 4.

**Figure 2:**
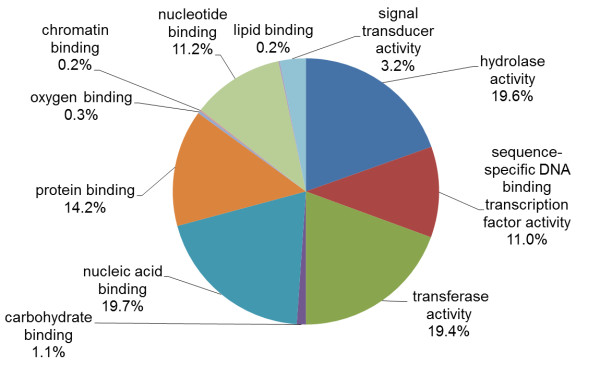
**Molecular functions of the genes incorporated in the phylogenetic analyses**. The pie chart includes the molecular functions assigned at level three of the Gene Ontology classification for the 527 sequences from the 157 gene families represented by two or more mapped genes in spruce and used in phylogenetic analyses.

We generated a spruce consensus linkage map for the white spruce (*Picea glauca *(Moench) Voss) and black spruce (*Picea mariana *(Mill.) B.S.P.) genomes (Figure 1, Additional files 1, 2, 3 and 4). This map encompassed 2,270 loci including 1,801 genes spread over the 12 linkage groups of spruce and corresponding to the haploid number of 12 chromosomes prevalent in the Pinaceae, including *Picea *(Figure 1). These genes represented a large array of molecular functions and biological processes (Figure 2 and Additional files 5 and 6, see Methods). Map length was 2,083 centiMorgan (cM) (Additional file 3). The number of mapped genes is more than twice that of the most complete spruce gene map available to date [28] and is in the same range as the map available for the loblolly pine genome, which includes 1,816 genes mapped over 1,898 cM [29]. Map length and the number of gene loci per chromosome thus appeared similar in spruce and loblolly pine.

### Gene density

**Figure 3:**
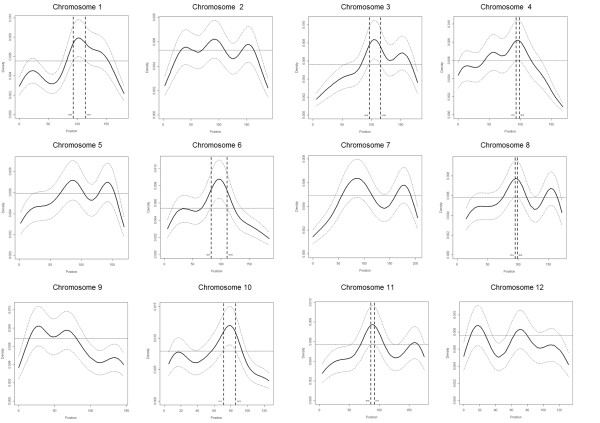
**Kernel density estimation for the spruce chromosomes**. On each plot, the curve in bold is the kernel density function and the dotted curves represent the limits of the confidence interval. The horizontal line represents the expected density of the uniform gene distribution. The vertical dotted lines represent the boundaries of the gene-rich regions: chromosomal regions for which the lower limit of the confidence interval of the density function is above the uniform function.

Our analyses revealed instances of gene clustering. Using Kolmogorov-Smirnov tests, the gene distribution deviated significantly from a uniform distribution for nine *(P *≤ 0.01) or ten (*P *≤ 0.05) of the 12 spruce chromosomes (Table 1). To localise gene-rich regions (GRRs), we conducted analyses of gene distribution relying on various bandwidths using kernel density estimation. The effect of the bandwidth upon the spread of the GRRs was weak (data not shown). At *P *≤ 0.01, only two GRRs were found on chromosomes 6 and 10; they included 1.3% of the genes (24) over 0.6% of the map length (14.7 cM). At *P *≤ 0.05, seven GRRs, including 9.2% of the mapped genes (166 out of 1,801), were found on seven chromosomes and represented 4.0% of the map length (Figures 1 and 3). In GRRs, gene density was about twice (1.78 gene/cM) that in the rest of the map (0.78 gene/cM). Tandemly arrayed genes (TAGs, see below) were not responsible for the higher gene density of the GRRs. There was no significant difference (*P *> 0.05) in the molecular functions represented by genes lying in the GRRs compared with the remainder of the map. However, regarding biological processes, the GRRs were enriched in Gene Ontology (GO) terms corresponding to metabolism (carbohydrate metabolic processes), reproduction, growth and regulation of anatomical structure (*P *≤ 0.05) (Additional file 7).

**Table 1:** Testing for gene clustering within spruce chromosomes using the Kolmogorov-Smirnov statistics (*D_n _*and D*_n_).

Chromosome	Number of mapped genes	Chromosome length (cM)	*D_n_*	D*_n_	*P*
1	166	180.6	0.0990	1.2812	≤ 0.01
2	177	185.3	0.0444	0.5948	> 0.15
3	145	172.6	0.1483	1.7946	≤ 0.01
4	134	168.3	0.1286	1.4967	≤ 0.01
5	149	167.1	0.0490	0.6013	> 0.15
6	141	180.3	0.1373	1.6451	≤ 0.01
7	160	204.5	0.0961	1.2207	≤ 0.01
8	173	157.4	0.0774	1.0226	≤ 0.02
9	142	145.8	0.0914	1.0953	≤ 0.01
10	136	120.4	0.1208	1.4219	≤ 0.01
11	143	171.2	0.0933	1.1219	< 0.01
12	135	131.7	0.1032	1.2051	≤ 0.01

### Tandemly arrayed genes

A total of 125 family members were organised into 51 TAGs (31 arrays within 1 cM and 20 arrays within 5 cM; Figure 1). Most of the arrays included two genes, but arrays were identified including up to eight genes, such as the *myb-r2r3 *array on chromosome 7 (Figure 1). Based on the GO classification, genes coding for extracellular proteins and cell wall proteins, and genes involved in DNA-binding functions and in secondary metabolism were over-represented among spruce gene arrays (Additional file 8). To test whether this distribution could be observed by chance alone, we randomly redistributed the 664 gene family members and counted the number of chromosomes represented for each family. This simulation was replicated 1,000 times. The observed and the simulated distributions were found to be significantly different (*χ*^2 ^= 35.7, degrees of freedom = 11, *P *= 0.00018). The main contribution to the *χ*^2 ^value was from the families with members mapping to a single chromosome. Seventeen gene families were found to be associated with a unique chromosome more often than would be expected by chance alone. The TAGs were the major contributors to this distribution.

### Co-localizing genes

Within 32 gene groups representing 71 genes (3.9% of the mapped genes), no recombinants were observed out of 500 white spruce progeny. These groups encompassed a variety of molecular functions with no significant deviation from the composition of the overall dataset (Additional file 9). In 20 groups, genes were related neither in sequence nor in function. By contrast, 12 groups were made of functionally related genes, including five tandem arrays and seven groups of genes from different families. These twelve groups involved three main functions: metabolism (six groups), regulation of transcription (three groups) and transport (three groups) (Additional file 9).

We obtained assessments of gene expression for co-localizing genes from 10 groups [30]. In three groups, the co-localizing genes were co-regulated across eight tissues (mature xylem, juvenile xylem, phelloderm (including phloem), young needles, vegetative buds, megagametophytes, adventitious roots and embryogenic cells). The first group included one citrate synthase involved in carbohydrate metabolism and a calcineurin B-like protein involved in transduction through calcium binding. The second group included one reductase involved in histidine catabolic process, which was co-expressed with a ribosomal 30S protein. The third group consisted of two chalcone synthases.

### Intergeneric map comparisons

**Figure 4:**
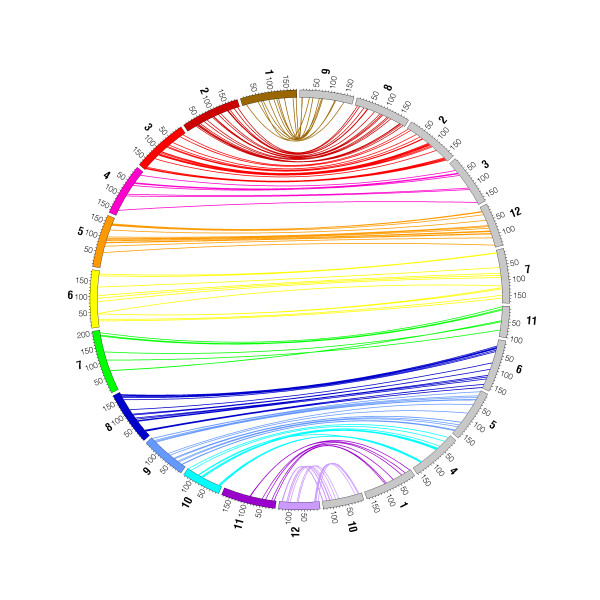
**A spruce/loblolly pine comparative map**. The syntenic positions of the 161 homologous genes mapped on both spruce and loblolly pine genomes were plotted with Circos [100] and are indicated by colour-coded lines connecting the spruce (in colour) and the loblolly pine chromosomes (in grey). The chromosome numbers are indicated outside the circle.

We compared spruce and pine gene sequences and their respective localizations on linkage maps, using that of *Pinus taeda *L. (loblolly pine) with 1,816 gene loci [31] and that of *Pinus pinaster *Ait. (maritime pine) with 292 gene loci [32]. In total, 212 gene loci were shared between spruce and pines. Out of them, 12 gene loci were syntenic between the three genomes, 51 were found between spruce and maritime pine, and 149 others were found between spruce and loblolly pine. Remarkably, the vast majority of the conserved pairs of gene sequences found among pairs of species could be mapped on homoeologous chromosomes (Additional file 10). Out of 165 genes mapped on both maps from spruce and loblolly pine, 161 (97.5%) were syntenic (Additional file 10), of which 88.8% were collinear (Figure 4 and Additional file 10).

Macro-synteny was spread all along the genomes with large conserved segments (Figure 4). The conserved positions of homologous genes allowed us to delineate the respective positions of homoeologous chromosomal regions in spruce, loblolly pine and maritime pine (Additional file 11). The conserved regions represented 82.0% and 86.5% of the lengths of the spruce and loblolly pine maps, respectively (Figure 4 and Additional file 10). The portion of 82.0% of the spruce map conserved with the loblolly pine map could be extended to 87.6% when conservation with the maritime pine map was also considered (Additional file 10). Thus, map comparison with maritime pine provided a significant enrichment in shared genes and homoeologous regions among maps. This high level of conservation enabled us to draw the first comprehensive map for a sizeable part of the gene space of the Pinaceae (Additional file 11).

### Phylogenetic analyses of 157 gene families

In total, 527 spruce genes were considered in the phylogenetic analyses. They were distributed in 157 families each containing at least two genes mapped on the spruce genome (Additional file 6). These families were distributed across diverse molecular functions, representative of the distribution of expressed genes found in white spruce (Figure 2, see Methods).

**Figure 5:**
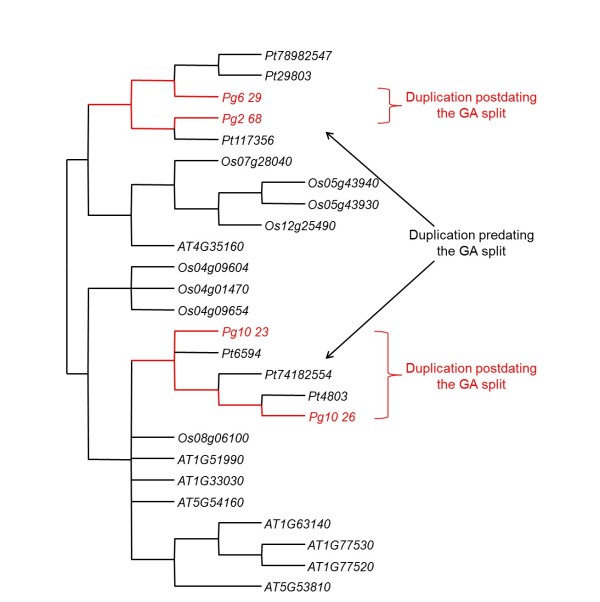
**Quercetin 3-O-methyltransferase gene family tree**. Unrooted phylogenetic tree obtained from the strict consensus of 50%-bootstrap consensus neighbour-joining and parsimony trees and indicating two spruce gene duplications post-dating the gymnosperm-angiosperm split (no intervening *Arabidopsis *or rice sequence between spruce sequences) and one spruce gene duplication predating the gymnosperm-angiosperm split (with intervening *Arabidopsis *or rice sequences between spruce sequences). Sequences are from spruce (Pg), pine (Pt), *Arabidopsis *(AT) and rice (Os). GA: gymnosperm-angiosperm split, estimated at around 300 Mya [13].

Additional file 12 provides the phylogenetic trees for all analysed gene families. Figure 5 shows the unrooted tree representative of the strict consensus between majority-rule bootstrap parsimony (MP) and majority-rule bootstrap neighbour-joining (NJ) trees obtained for the quercetin 3-O-methyltransferase family. In this example, two pairs of genes (*Pg6-29*/*Pg2-68 *and *Pg10-23*/*Pg10-26*) resulted from recent duplications after the GA split (Figure 5). One pair clustered on chromosome 10, while the two other genes were translocated on chromosomes 2 and 6 of spruce (Figure 5). Another more ancient duplication giving rise to the two gene lineages leading to *Pg2-68*/*Pg6-29 *and *Pg10-23*/*Pg10-26 *occurred before the GA split, with the two groups located on different spruce chromosomes, implying at least one translocation (Figure 5).

**Figure 6:**
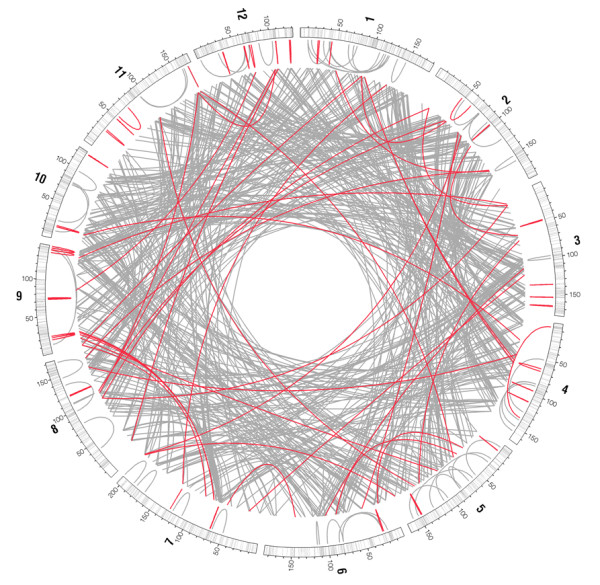
**Organization of the spruce gene space and duplications**. Genome representation with spruce chromosomes (1 to 12) showing from outside to inside: the 12 chromosomes with ticks representing the genes mapped along the spruce linkage groups, and with genetic distances in cM (Kosambi); links between genes representing duplications within chromosomes and duplications followed by inter-chromosomal translocations. Links in grey illustrate ancient and links in red illustrate recent, referring to before or after the gymnosperm-angiosperm split, around 300 Mya [13].

Using the strict consensus of majority-rule bootstrap NJ and MP phylogenetic trees for each of the 157 gene families, we evaluated, in a similar fashion, the relative age of duplications for a total of 992 gene pairs (nodes) relative to the GA split. Topological differences between NJ and MP trees affected 115 gene pairs (11.6%) whereas 877 gene pairs (88.4%) were positioned identically by the two analytical approaches, relative to the GA split. Out of these 877 congruent results, 688 pairs (78.4%) diverged before the GA split, 87 pairs (9.9%) diverged after the GA split and the divergence of 102 pairs (11.6%) could not be determined because of lack of support (polytomies). In other words, there were about eight ancient duplications for each recent one (Figure 6).

### Distribution and relative age of gene pairs

We analysed the distribution patterns found among the gene pairs on the spruce genome. Most spruce gene pairs were translocated (86.3%) and most of these translocations occurred before the GA split (94.5%). We counted the number of duplicates found on each of the 12 chromosomes, and compared the observed distribution to a theoretical distribution that would be expected by chance alone. Out of 688 gene pairs (or nodes) representing 'ancient' duplications, 56 pairs (8.1%) were located on the same chromosome and 632 pairs (91.9%) were duplicates involving a translocation to another chromosome. This difference was highly significant (*χ^2 ^*= 482.2; *P *< 2.2e^-16^), indicating that ancient gene pairs have been highly dispersed. Out of 87 pairs of genes representing 'recent' duplications, only 37 pairs (42.5%) were translocated and 50 pairs (57.5%) were located on the same chromosome. This difference was not significant (*χ*^2 ^= 1.9; *P *= 0.16). For each pair of genes, we computed the distance between the duplicates found on a same chromosome. The mean distance between duplicates arising from a recent duplication event was 4.3 cM; whereas this distance was 47.0 cM between duplicates derived from ancient duplication events. This 10-fold difference was highly significant (Welch t-test *t *= -7.8; *P *= 1.1e^-11^).

Many gene copies found on the same chromosome were forming arrays of genes tandemly duplicated within 5 cM. Within the 51 tamdemly gene arrays that incorporated 6.9% of the mapped genes, 125 gene pairs (duplications) could be classified relative to the GA split: 44 were classified as recent, only 5 were ancient, and 76 were undetermined. Overall, only four gene arrays could be accounted for by ancient duplications predating the GA split (*BAM*, *expansin-like*, *pectinesterase*, *tonoplast intrinsic protein*), whereas 29 other arrays were generated by duplications after the GA split (*χ*^2 ^= 18.9; *P *= 1.3e^-05^; Figure 6). Thus, the more recent origin of these closely-spaced duplicates has apparently resulted in less time and opportunity for them to be dispersed or translocated.

## Discussion

The completion of several genome sequencing projects in angiosperms has resulted in improved knowledge of the content and organisation of the flowering plant genomes. In gymnosperms, in the absence of a completely sequenced and ordered genome, recent efforts have been put toward improving knowledge of the gene space through several EST sequencing projects [33]; but the structural organisation of this gene space on the genome remains largely undetermined [34]. The spruce genetic map and analyses presented herein allow better comprehension of the genome macro-structure for a gymnosperm. These results combined with phylogenies reveal the relative proportion of gene duplications shared between angiosperms and gymnosperms or unique to gymnosperms, and how the seed plant genome has been reshuffled over time from a conifer perspective.

### Gene distribution and density

To localise the GRRs, we implemented a statistical approach based on the kernel density function. This represents a technical improvement compared with existing methodologies given that we used an adaptive kernel approach to avoid the use of an arbitrarily fixed bandwidth. This approach allowed us to take into account the density observed locally to compute the bandwidth size. Because the number of genes currently positioned on the spruce genome represents around 6% of the estimated total number of genes [26], we applied stringent parameters in these analyses to reduce the rate of false positives. Thus, we may have underestimated the extent of GRRs. Besides these significant peaks, a few other peaks of kernel density that do not currently reach significance (Figure 3) may do so with an increased number of mapped genes. Indeed, Kolmogorov-Smirnov tests of homogeneity of gene distribution indicated that nine chromosomes had a significantly non-uniform distribution. Even so, there does not seem to be a widespread occurrence of GRRs on the spruce genome. In addition, the seven significant GRRs were distributed among seven chromosomes. This peculiar distribution suggests that GRRs may correspond to centromeric regions where, on genetic maps, markers tend to cluster due to more limited recombination.

In angiosperms, species with small genomes tend to be made of GRRs alternating with gene-poor regions. For example, the genic space of *Arabidopsis thaliana *represents 45% of the genome while the remaining 55% is 'gene-empty' and interspersed among genes as blocks ranging in size from a few hundred base pairs to 50 kb [35]. By contrast, plant species with larger genomes do not show such a contrasted gene distribution, in line with the pattern found here for the large spruce genome. Rather, they harbour a gradient of gene density along chromosomes, such as in maize [36], soybean [37] and wheat [38,39]. In the soybean genome, a majority of the predicted genes (78%) are found in chromosome ends, whereas repeat-rich sequences are found in centromeric regions [40]. In conifers, retroelements have been reported as a large component of the genome, with some families well dispersed while others occur in centromeric or peri-centromeric regions (for example, see [41-45]). Thus, they might have participated in shaping the distribution of genes along chromosomes by reducing the occurrence of GRRs.

The type of gene distribution along the genome bears consequences for the planning of genome sequencing strategies. For instance, a gene distribution of 'island' type implies that a deeper sequencing effort is necessary to reach a majority of the genes [38]. Though genetic distance does not equate physical distance, the pattern seen here in spruce indicates that genetic maps alone that would include most of the gene complement will be insufficient to anchor a significant portion of physical scaffolds, especially if these are small. In conifers, little is known about physical gene density in genomic sequences. In spruce, two partially sequenced BAC clones had a single gene per 172 kbp and 94 kbp, respectively, which represents a density at least 10-fold lower than the average gene density of the sequenced genomes of *Arabidopsis*, rice, poplar or grapevine [46]. In addition, the sequencing of four other randomly selected BAC clones in spruce failed to report any gene [45].

### Tandemly arrayed genes and functional clusters

In the present analysis, we identified two types of gene clusters: arrays of gene duplicated in tandem and arrays of unrelated sequences sharing functional annotations. There were 51 arrays (TAGs) encompassing genes from the same family that were duplicated within 5 cM. They incorporated 6.9% (125) of the mapped genes and they could be indicative of small segmental duplications. Such TAGs were also reported in genomic sequences of model angiosperms: they involve 11.7% of the *Arabidopsis *genes and 6.7% of the rice genes [47]. Most of the spruce arrays (78.0%) included only two genes. Similar proportions were found in genome sequences of model angiosperms [47]. The largest spruce array found consisted of eight *myb-r2r3 *genes on chromosome 7 (Figure 1). Interestingly, seven of these *myb-r2r3 *belong to the same subgroup Sg4C [48]. The other genes belonging to Sg4C were not positioned on this linkage map. Spruce TAGs were significantly enriched in functions related to DNA binding, secondary metabolism and structural proteins (Figure 1). In *Arabidopsis *and rice, TAGs are under-represented among transcription factors and over-represented in enzymes [47]. GO analyses and expression data showed a strong correlation between tandem duplicates and biotic stress genes in *Arabidopsis *[49], leading the authors to suggest that 'tandem duplicates are likely important for adaptive evolution to rapidly changing environments'. In the *myb-r2r3 *gene array, the three genes named *PgMyb5*, *PgMyb10 *and *PgMyb13 *exhibited very different expression patterns [50]. The lack of co-expression of the genes mapped in arrays did not support a gene arrangement oriented by co-regulation. In these arrays, a majority of the genes were derived from duplications occurring after the GA split but were shared between spruce and pine, indicating from the perspective of geological time that expression divergence may occur quite rapidly after gene duplication [51]. Such a pattern is in accordance with the observation that if a new function is not acquired rapidly through neo-functionalisation, one duplicate tends to evolve towards a pseudogene and disappear [7]. A high frequency of pseudogenes has indeed been reported in conifer genomes [26,42,52]. However, there are exceptions to this neo-functionalisation trend among surviving duplicates, such as in the conifer *Knox-I *family. In this family, the closely located *kn1 *and *kn2 *arose from a duplication postdating the divergence between gymnosperms and angiosperms; nevertheless, neo-functionalisation has not happened yet between these duplicates in spite of the duplication occurring before the divergence between the spruce and pine lineages, more than 100 Mya [24]. Sub-functionalisation of these duplicates has been noted [24], conferring partial functional redundancy that might enhance survival and adaptation in these long-lived perennials. Several other cases of conifer-specific duplications might exist that imply partial redundancy of function instead of neo-functionalisation.

We found three clusters made of co-expressed gene sequences that were similar to operon-like structures: two cases made of non-homologous sequences and a third one made of tandemly duplicated chalcone synthases. In angiosperms, only five such structures have been described and were associated with secondary metabolism and defence mechanisms [53]. Such metabolic clusters have emerged as a new and growing theme in plant biology [54]. The three clusters found in our study were similar to these functional clusters, except that their roles were not restricted to secondary metabolism. Among our data, two other cases for which we could not obtain expression data were also good candidates for functional clusters. On chromosome 11, there were also two co-localising pectin methylesterases. Moreover, a single group of two non-homologous sequences was clearly involved in the secondary metabolism. This group encompassed one flavonol synthase and one glutathione synthase on chromosome 6 of *Picea*. Glutathione plays several important roles in the defence of plants against environmental threats. It is a substrate for glutathione *S*-transferases, enabling neutralisation of potentially toxic xenobiotics [55]. Thus, these flavonol and glutathione synthases may belong to a cluster of functionally related but non-homologous genes. Similarly, 80 co-expression clusters sharing the same GO term were described along the 3B wheat chromosome, suggesting the existence of adaptive complexes of co-functional genes [39]. In spruce, exhaustive transcriptomic resources have recently been developed [30]. Their analysis combined with the positioning on the genome of additional genes should allow us to pinpoint whether adaptation at the metabolic level has contributed to shaping the organisation of the gene space.

### Highly conserved organisation of the gene space between spruce and pine

Before conifer gene catalogues were available, the number of available orthologous markers to enable comparative studies of genome macro-structure between conifer species was highly limited [34]. A substantial conservation of the Pinaceae genome macro-structure was nevertheless suspected [34,56,57]. Estimating the extent of conservation in genome macro-structure was more exhaustive in our study, because we identified a much enlarged set of orthologous mapped gene loci (over 200) between spruce and pine. Synteny and collinearity between the spruce and loblolly pine genomes were very high. Lower collinearity was noted with the maritime pine genome, which resulted from likely lower accuracy of the gene order based on the use of a smaller mapping population for this species [32]. Therefore, it is safe to assume that the organisation of the conifer gene space has been largely maintained over a period dating back 120 to 140 Mya, since the early diversification of Pinaceae in its main lineages in the Early Cretaceous [13,14,27]. Such a high level of conservation of the genome macro-structure has also been reported among angiosperm genomes from the Rosids and Asterids clades [58,59], which diverged about 115 Mya [58-60], a time period similar to that of the pine-spruce divergence. By contrast, since the monocot-eudicot split 140 to 150 Mya [61], which slightly preceded the pine-spruce divergence, synteny has been largely disrupted between model monocots and dicots [62]. Such large discrepancies in apparent rate of evolution of genome macro-structure are largely conspicuous among angiosperm lineages, where it has been shown that the genomes of perennial species such as grape and poplar evolved slower than those of annual species such as *Arabidopsis *and rice [63]. These differences in evolutionary rates are also reminiscent of those in substitution rates between annual and perennial or woody seed plants, where various hypotheses related to mutation rate, generation time, population size and fixation rate have been proposed [64-68].

### Age and organisation of gene duplicates

The phylogenetic analysis of 157 gene families indicated a large imbalance in favour of ancient duplications predating the GA split versus more recent duplications postdating the GA split. Since the genes sampled in the present study were identified after sequencing ESTs, one could argue that the sample might be biased towards expression patterns that are possibly related to high sequence conservation (for example, [69]), hence artificially increasing the ratio of ancient versus recent gene duplications detected in the present study. First, the ratio was highly asymmetric (eight to one) and we showed that the genes and families involved in our study were representative of a large array of molecular functions and biological processes seen in the most recent spruce gene catalogue, and implicating conserved and less conserved gene families [26]. Gene annotations in conifers [26,70,71] also do not favour this hypothesis. Indeed, the most complete catalogue of expressed genes for a conifer, which was based on a large effort involving the sequencing of 23,589 full-length cDNA inserts, has recently enabled the reporting of the most exhaustive comparison of homologous genes sequenced both in angiosperms and gymnosperms [26]. The results indicated that the spruce protein families were largely overlapping with those of angiosperm model plants completely sequenced [26]. Comparing the occurrence of the Pfam domains in the spruce gene catalogue with genomes completely sequenced from model plant species showed that only 28 protein domains were statistically over-represented in spruce and most of them were involved in metabolism, stress response and retrotransposition. Moreover, the gene coding portion of the spruce genome was evaluated at around thirty thousand transcribed genes, a number in the same range as that observed for model angiosperm genomes [26]. The in-depth study of a few transcription factor families also showed that conifers lack some members in specific subfamilies while containing more genes in closely related subfamilies that were derived from duplication events postdating the GA split [24,48,72]. These various observations suggest that the conifer genes that are highly divergent from their angiosperm's homologues are rare in the sequence resources developed so far, in spite of the fact that these resources relied on the investigation of a diversity of tissues and conditions [26]. In the future, the availability of the genome sequence may allow the discovery of more conifer-specific genes that could be highly duplicated; but we would (do?) not expect to find them in abundance, as suggested by the present phylogenetic analysis.

A large majority of spruce gene pairs were translocated and most of these translocations occurred before the GA split, affecting a large majority of the 157 gene families analysed. By contrast, genes duplicated after the GA split were located overwhelmingly in close proximity on the same chromosome and often organised in tandem. These trends were consistent with the observation that the physical distances between duplicates on the *Caenorhabditis elegans *genome increase with time, due to chromosomal rearrangements and other mutational events [73]. Nevertheless, this pattern is not always clear, for instance in *Arabidopsis *gene families where the occurrence of tandem duplications and segmental duplications are negatively correlated [47,74]. In this model plant, unequal cross-over and gene loss were proposed as possible mechanisms leading to the counter-selection of tandem duplications [74].

The observed large excess of ancient duplications predating the GA split over more recent duplications postdating the GA split is consistent with the hypothesis of relative stasis in the gymnosperm lineage leading to conifers and the little evidence for a recent large expansion of the gene space. While a single whole genome duplication has been hypothesised to have affected the common ancestor of seed plants around 350 Mya [20], evidence for more recent widespread polyploidy in the gymnosperm lineage after its divergence from the angiosperm lineage was not found in the present study, in agreement with results from cytological studies reporting a rare occurrence of this phenomenon in gymnosperms [18,75]. Variation in basic chromosome number in the diploid gymnosperms would rather be the result of chromosome fusion or fission [75]. For instance, such fission would have led to the additional chromosome seen in Douglas fir, relative to other Pinaceae [56]. If so, some of the translocations hypothesised in the present study after the GA split could also be the result of ancient chromosomal fissions increasing the basic chromosome number in the lineage leading to spruce and pine.

While more recent duplications specific to the gymnosperm lineage leading to extant conifers were detected, the stasis of genome macro-structure noted in this lineage is in concordance with that observed between the spruce and pine genomes, and corresponding to a period exceeding 100 My since the last common ancestor of Pinaceae [13,14,27]. Such slow rates of genome evolution parallel the slow rates of speciation and patterns of reticulate evolution noted in Pinaceae taxa [76,77] and their archaic morphological features and life history [14,78]. Such multiple coincidences reinforce the idea that perennial nature and large historical population sizes are key factors to the slow evolution of conifers [65]. The large excess of ancient duplications detected in the present study also indicates that much gene expansion has occurred in the plant lineage before the divergence between gymnosperms and angiosperms in the Late Carboniferous. Part of this expansion in the primitive land plants might be related to the major burst of duplications noted in transcription factors and coinciding with the water-to-land transition of plants [79]. Further studies implicating other divisions of green plants are needed to better comprehend the temporal dynamic of gene family expansions and reshuffling of the plant genome before the emergence of modern seed plants.

## Perspectives

The high levels of synteny, collinearity and similar ranges of linkage distances noted between the spruce and pine genomes will provide opportunities to transfer genomic information between these genera, especially if such conservation is maintained at a finer scale. The reported genomic features are likely to extend to other genera of the Pinaceae and therefore save time and energy in the deployment of genomic resources necessary to identify orthologous regions in these ecological and economic important species. Such mutual enrichment of genetic maps across species is also significant, with respect to the complexity underlying the analysis of the large conifer genomes. Increasing the number of homologous genes mapped on both the spruce and pine genomes will increase the resolution of comparative mapping efforts, which in turn should highlight the extent of micro-arrangements since the divergence of Pinaceae taxa. Such dense gene maps and high structural correspondence between them will also help identify homoeologous quantitative trait loci responsible for adaptation [28] and other complex characters, between species and genera. While successfully applied to dissect nitrogen use efficiency in cereals [80], these efforts in comparative structural genomics should provide insight into the evolutionary trajectories of the conifer genome at the functional level.

## Methods

### Genotyping of gene SNPs

A collection of 27,720 white spruce cDNA clusters [26] was used to develop gene SNPs (Additional file 4). *In silico *SNP discovery in expressed white spruce (*Picea glauca *(Moench) Voss) genes was conducted after Sanger resequencing and according to parameters previously reported [81]. A GoldenGate (Illumina, San Diego, CA, USA) SNP array (PgLM2) specifically designed to map additional white spruce genes was constructed with 1,536 attempted SNPs dispersed through 1,509 expressed genes. The assay was conducted following procedures previously described [82]. Genotyping was done at the Genome Québec Innovation Centre (McGill University, Montreal, QC, Canada, team of A. Montpetit) and by using 250 ng of template DNA per sample (at a rate of 50 ng/μL). The results were analysed with the BeadStudio software (Illumina). We retained the 1,292 SNPs exhibiting a GenTrain quality score of 0.25 or more. Of these, 1,121 (73.0%) SNPs representative of 1,098 genes segregated among the 500 progeny of the white spruce cross D (C94-1-2516, ♀77111 × ♂2388). The average call rate per valid SNP was 99.48%. In total, 773,395 new genotypes were obtained from valid SNPs. Using replicated positive controls, the rate of reproducibility was estimated at 99.994%.

### Annotation of the mapped genes

The mapped genes were representative of a large array of molecular functions and biological processes including wood formation, growth, vascular tissues development and differentiation, responses to abiotic and biotic stresses and adaptation [83,84]. They were mostly derived from expressional and functional studies [83,84] as well as outlier detection [85] (Additional files 4, 5 and 6).

GO annotation was performed with the Blast2GO software [86]. GO terms were assigned based on the top 10 *blastx *hits found against the non redundant (nr) protein database with an E-value below 1e^-10^. GO annotation was run based on the terms from the PlantGO-Slim classification for molecular functions, biological processes and cellular components. Annotations were used to assess GO term enrichment using the Fisher exact test function implemented in Blast2GO.

In total, 6,923 GO terms including molecular functions, biological processes and cellular components were assigned to the 1,801 mapped genes, for an average of 3.9 GO terms per gene. There were 1,285 sequences associated with a molecular function. At level two of the molecular function classification, most of the terms fell in the 'binding' category (49.0%) or in the 'catalytic activity' category (40.0%). At the level three of the classification, five categories each represented about 15% to 20% of the genes; therefore, most of the terms were related either to hydrolase activity, transferase activity, nucleic acid binding, protein binding or lipid binding (Additional file 5). Also, 1,027 sequences were associated with biological processes with a large diversity of 84 terms involved in the annotations (Additional file 5).

### Spruce gene linkage maps

Two parental linkage maps for white spruce were estimated *de novo *from the 500 progeny above for cross D. Parental maps were assembled as previously described [56], using the genotyping data of the PgLM2 array, as well as data from a previous GoldenGate SNP array on the same 500 progeny [28]. Anonymous markers and gene markers from previous linkage mapping projects involving a different white spruce cross (cross P: C96-1-2516, ♀80112 × ♂80109; 260 progeny) were also considered [56,82]. The white spruce maps derived from both crosses D and P [28] were merged with the JoinMap 4.0 function 'Combine Groups for Map Integration'. Before marker ordering within each integrated linkage group, we compared the recombination frequencies estimated for homologous markers from both data sets. For this purpose, a 'heterogeneity test' was conducted with JoinMap4.0. As a result, pairs of loci showing significantly different recombination frequencies (*P *< 0.01) were eliminated to avoid erroneous marker ordering.

A black spruce (*Picea mariana *(Mill.) B.S.P.) map [82] was also used to position 58 additional genes (3% of the total mapped genes) that were not mapped in white spruce. Because the genomes of the two species are highly syntenic and collinear [82], white spruce and black spruce genetic maps were aligned based on 258 gene markers mapped in common. The white spruce composite map was taken as the reference map; then, black spruce gene loci not positioned in white spruce were transferred onto the white spruce composite map. The position of a transferred gene on the linkage group was estimated as the middle of the interval between the two closest white spruce anchor loci. Hence, the spruce composite map included 1,743 genes from white spruce and 58 genes from black spruce for a total of 1,801 positioned genes.

### Gene distribution along the chromosomes

We tested whether the genes were uniformly distributed along the chromosomes or whether they were clustered. For each chromosome, we compared the observed gene distribution with a uniform distribution by using the Kolmogorov-Smirnov test. We computed the maximum difference (noted *D_n_*) between the observed distribution *F(x) *and the empirical distribution *F_n_(x)*. With large sample sizes such as those considered here (in terms of genes), *D_n _*follows a complex distribution and the critical value depends on the sample size. D'Agostino and Stephens proposed a modified statistic named D_n_* with critical values independent of the sample size [87]. The critical values of statistical significance used for D_n_* were 0.895 (*α *= 0.05) and 1.035 (*α *= 0.01).

### Gene density analysis

To identify the location and extent of GRRs, we used the kernel density estimation, a non-parametric technique based on the kernel density function [88]. The choice of the bandwidth is arbitrary and affects the smoothness of the distribution. The function *asciker *available in the software Stata^® ^10 (College Station, TX, USA) was used to compute the non-parametric density estimator as well as the confidence intervals at two significance levels (*α *= 0.05 and 0.01) [89]. We used the adaptive kernel method [90], which is based on a varying bandwidth instead of an arbitrarily fixed one. This approach enabled us to take into account the density observed locally to compute the bandwidth size. The density and the 95% confidence intervals were calculated with the *akdensity *function in Stata^®^10. Then, we compared the position of the confidence interval with the uniform function in the R package. If the lower bound of the confidence interval was greater than the uniform distribution, the region was declared as a GRR (*P *< 0.05).

### Synteny with pine genomes

The gene-based linkage map for the loblolly pine genome [29] was downloaded from the Dendrome database [31]. Out of 1,816 genes mapped onto the loblolly pine genome, a dataset of 1,666 genes was retrieved from the Dendrome database (accession TG091). These sequences were compared with 27,720 spruce unigenes [91]) and the 1,801 spruce mapped loci by using the *blastn *program. Also, 426 mapped loci were collected from maritime pine [32]. For each pine sequence, the best hit among the spruce genes with various thresholds from 70% to 95% of identity was retrieved. All the spruce sequences matching a loblolly pine sequence with at least 95% of identity were located on a homoeologous chromosome. However, to increase the number of matches analysed, a minimum identity level of 80% was retained. Under these circumstances, 7.2% of the matches were not found on homoeologous pairs of chromosome. Moreover, we performed the reciprocal comparison involving mapped spruce genes against the pine sequence database. If one link was found between the pine and spruce chromosomes in both reciprocal analyses, we declared this link as homologous. In a few cases, one gene from one species could match several genes from the other species on the homoeologous chromosome. In such cases, a single link was retained between the two homoeologous chromosomes.

### Gene families used in phylogenetic analyses

Phylogenetic analyses were conducted by including angiosperm and gymnosperm (conifer) sequences. These involved 527 mapped spruce genes from 157 families. To assess the representativeness of this sample (Figure 2), we compared the distribution of the molecular function GO terms found across these genes and across the white spruce GCAT gene catalogue [26] with a two-tailed Fisher exact test. Out of the seven terms assigned at the level two of the molecular function's classification, only two terms were over-represented among the mapped families compared with the gene catalogue (False Discovery Rate (FDR) ≤ 0.01). These terms were 'binding' (GO:0005488) and 'transcription regulator activity' (GO:0030528). Indeed, 31 families encompassing 180 sequences were related to transcription activity or regulation. The four largest groups of mapped transcriptional regulators included 21 genes from the *myb-r2r3 *family, 17 genes from the *b-hlh *family, 14 *nam *genes and 11 *aux-iaa *genes. At the level three of this classification, only two terms were differentially represented among the mapped families and the spruce gene catalogue (FDR ≤ 0.01). Genes related to nucleic acid binding (GO:0003676) were over-represented while the ones related to transferase activity transferring glycosyl groups (GO:0016757) were under-represented among the mapped genes. Thus, there was no large molecular functional class missing in the sample of 157 families used for the phylogenomic analysis, which was quite representative of the overall relative diversity and abundance of molecular functions seen in the spruce GCAT gene catalogue [26].

### Phylogenetic analyses

The phylogenetic analyses aimed to classify duplications involving mapped spruce genes as recent or ancient by considering the GA split as a reference (see below). We translated the spruce sequences with the *getorf *program available in the EMBOSS package [92]. Among all possible open reading frames, we selected the sequence with a match with a protein sequence from another species. The *Arabidopsis *protein dataset (TAIR7 release) from TAIR [93]) and the rice protein dataset from the rice annotation database [94]) were retrieved. We also built a protein sequence dataset from pine. Although not a strict requirement for the present purpose of dating spruce gene duplications, we also retrieved two datasets of EST contigs derived from *Pinus pinaster *and *Pinus taeda *[95]. We concatenated the 12,901 sequences from *Pinus pinaster *and the 72,928 sequences from *Pinus taeda *into a single file. These sequences were translated with the *getorf *program to obtain a set of all possible protein sequences. For each spruce sequence, we conducted three *blastp *searches against the protein datasets from *Arabidopsis*, rice and pine. In the *blastp *outputs, we screened the five best hits. We aligned the spruce, pine, *Arabidopsis *and rice homologous sequences with the *kalign *program [96] and we selected manually conserved domains for further phylogenetic analysis. Because the considered angiosperm sequences were from complete genomic sequences, it was not necessary to filter out sequences based on absolute sequence similarity. However, the observed e-values in the *blastp *searches of the spruce sequences against the *Arabidopsis *proteome were in the range of e^-50 ^to e^-100^.

Phylogenetic analyses were conducted for 157 gene families (Additional file 5). To be considered, a family had to contain at least two mapped members on the spruce genome and had to be represented in *Pinus *as well as in both models and completely sequenced angiosperms *Arabidopsis *and rice, respectively an eudicot and a monocot. Both the NJ method [97], an approach based on matrices of substitution rates, and MP analysis [98] were employed using the package Phylip 3.6 [99]. We used MP and NJ methods instead of other more computer-intensive approaches such as Bayesian or maximum-likelihood algorithms given that the metric that we wanted to estimate, the proportion of ancient versus recent spruce gene duplications relative to the GA split, was based on a large sample of gene families (157), and given that our interpretation of the topologies was conservative because it was based on the strict consensus from MP and NJ trees for each gene family. Results obtained for a few gene families using other more computer-intensive phylogenetic methods resulted in essentially the same consensus trees (results not shown). To estimate NJ trees, distance matrices were calculated using the *protdist *program with the JTT amino acid substitution matrix and submitted to the program *neighbor*. Parsimony trees were estimated using *protpars*. For each family and each method, the robustness of the topologies obtained was assessed by means of 500 bootstraps using the program *seqboot*. For each method, the consensus tree derived from the bootstrap analysis was the majority-rule consensus generated with the program *consense*. Then, for each gene family phylogeny, only nodes supported minimally by 50% of bootstraps and in concordance between the two phylogenetic methods were retained, that is, the strict consensus of two majority-rule bootstrap MP and NJ trees.

For each gene family phylogeny, we used the unrooted strict consensus of the MP and NJ trees and estimated the relative age of spruce gene duplications by determining if they occurred before or after the GA split. Nodes involving spruce gene sequences but with no intervening angiosperm sequences from *Arabidopsis *or rice indicated duplications post-dating the GA split and were referred to as recent duplications. On the other hand, when spruce gene sequences were separated by intervening nodes involving angiosperm sequences from rice and/or *Arabidopsis*, these duplications predated the GA split and were referred to as ancient. In some cases, ancient gene duplicates produced before the GA split may have been lost both in the *Arabidopsis *and rice genomes, which would bias upward the number of duplications that would be declared specific to the gymnosperm lineage leading to spruce. Based on the literature, one may argue that this bias should be negligible, given that loss of gene duplicates mostly occurs quickly after duplication if processes such as sub-functionalisation or neo-functionalisation do not occur [7]. At the same time, sampling two divergent and completely sequenced angiosperm genomes (representative of the eudicot and the monocot lineages) should keep such a bias low because gene loss would have to occur independently in both angiosperm lineages after their split, or in their common ancestor during the relatively short period between the GA split (around 300 Mya) [13] and the divergence of monocots from other angiosperms (around 150 Mya) [61]. For instance, the sister lineage of the large *knox-1 *gene family in conifers has been lost in rice and other monocots but conserved in *Arabidopsis *and other eudicots [24]. Finally, because this bias would result in the scoring of a number of false-positive gymnosperm-specific duplications, it would tend to reduce the true proportion between ancient duplications predating the GA split and more recent duplications post-dating this split. If real, the bias is likely negligible, given that the observed value of this proportion was already highly skewed toward ancient duplications (ratio of eight to one, see Results).

All circular genetic maps were drawn with the Circos software [100].

## Competing interests

The authors declare that they have no competing interests.

## Authors' contributions

NP: bioinformatics and statistical analyses; BP and NI: linkage mapping; NP, JB and JL: phylogenetic analyses; JB and PR: design of the genotyping assay and data quality control; NP and JB: preparation of the manuscript. All authors read and approved the final manuscript.

## Supplementary Material

Additional file 1 **Composite spruce gene linkage map**. This composite linkage map consisted of 1,801 genes, including 1,743 genes from white spruce and 58 genes from black spruce, positioned onto the 12 linkage groups corresponding to the 12 spruce chromosomes. Genes highlighted in grey were positioned on both spruce genomes and genes written in red were positioned only onto the black spruce composite map [82]. All other genes were positioned on the white spruce composite map. Genetic distances are indicated in cM (Kosambi) at the left of each linkage group. For magnification, zoom into the figure. Click here for file

Additional file 2 **Parameters of expanded main gene linkage map for white spruce**. Click here for file

Additional file 3 **Parameters of the composite genus-level spruce gene linkage map**. Click here for file

Additional file 4 **Gene position along the spruce chromosomes, accessions, sequence and annotation**. Click here for file

Additional file 5 **Gene ontology distribution**. Gene ontology terms assigned to the 1,801 mapped spruce genes at the level 3 of the **(A) **molecular functions and **(B) **biological processes. Only categories including five genes or more are represented. Click here for file

Additional file 6 **Gene families and number of genes mapped on the spruce genome for each family**. Click here for file

Additional file 7 **Over-representation of gene ontology classes in the gene-rich regions based on Fisher exact tests**. Click here for file

Additional file 8 **Over-representation of gene ontology classes in the gene arrays based on Fisher exact tests**. Click here for file

Additional file 9 **Cluster of co-localizing genes: annotation and expression**. We collected expression data from a transcriptomic database covering eight tissues and including mature xylem, juvenile xylem, phelloderm (including phloem), young needles, vegetative buds, megagametophytes, adventitious roots and embryogenic cells [30]. A level of expression was assigned to each tissue and to each gene represented on a microarray. Correlation tests were performed based on the level of expression. Co-expression was declared if the *P*-value was lower than 0.01 (**) or 0.05 (*). Click here for file

Additional file 10 **Conservation between the chromosomes from *Picea *and *Pinus taeda *or *Pinus pinaster***. Click here for file

Additional file 11 **Coordinates and annotation of the conserved genes found on homoeologous *Picea *and *Pinus *chromosomes**. Click here for file

Additional file 12 **Unrooted majority-rule bootstrap trees obtained with the neighbour-joining (NJ) and the maximum parsimony (MP) methods for 157 gene families of seed plants**. Click here for file
