# Supplementary material for: A spruce gene map infers ancient plant genome reshuffling and subsequent slow evolution in the gymnosperm lineage leading to extant conifers
Source: BMC Biol. 2012 Oct 26;10:84. doi: 10.1186/1741-7007-10-84 (PMC3519789; doi:10.1186/1741-7007-10-84)
Supplement: Additional file 2 — Parameters of expanded main gene linkage map for white spruce. [file 1741-7007-10-84-S2.PDF]

Parameters of expanded main gene linkage map for white spruce.

Mapping parameters	Parents		Main sub-composite
	♀77111	♂2388	
Total number of available SNP gene loci	1,199	1,138	1,824
Number of loci with segregation distortion [a]	37	37	52
Total number of loci without segregation distortion	1,162	1,101	1,772
Total number of assigned SNP gene loci	1,152	1,083	1,772
Number of positioned SNP gene loci	1,150	1,077	1,710
Number of positioned accessory markers	17	14	9
Number of linkage groups (chromosomes)	12	12	12
Map length G_F, cM (Kosambi)	2,130.8	1,771.0	2,304.2
Average map density, cM (Kosambi)	1.85	1.64	2.8
Average size for major linkage groups, cM (Kosambi)	177.6	147.6	192.1
Expected map length *Ge*, cM (Kosambi)	2,680.3	2,255.5	-
Expected map coverage *Ce*	99%	99%	-

[a]Bonferroni correction: $P \leq 0.01$/number of loci.
