# Supplementary material for: A spruce gene map infers ancient plant genome reshuffling and subsequent slow evolution in the gymnosperm lineage leading to extant conifers
Source: BMC Biol. 2012 Oct 26;10:84. doi: 10.1186/1741-7007-10-84 (PMC3519789; doi:10.1186/1741-7007-10-84)
Supplement: Additional file 3 — Parameters of the composite genus-level spruce gene linkage map. [file 1741-7007-10-84-S3.PDF]

Parameters of the composite genus-level spruce gene linkage map.

Mapping parameters	Composite
Number of loci positioned from two sub-composite white spruce linkage maps	2,211
Number of AFLP[1] loci	456
Number of SSR[1] loci	12
Number of ESTP[1] gene loci	31
Number of SNP[1] gene loci	1,713
Composite white spruce map length G_F, cM[1] (Kosambi)	2,065
Average map density, per cM (Kosambi)	0.93
Gene density, per cM (Kosambi)	1.18
Number of additional SNP gene loci positioned from composite black spruce linkage map	58
Composite spruce map length G_F, cM (Kosambi)	2,083
Average map density, per cM (Kosambi)	0.92
Gene density, per cM (Kosambi)	1.16

[1] Abbreviations: AFLP: Amplified Fragment Length Polymorphism, SSR: Simple Sequence Repeats, ESTP: Expressed Sequence Tag Polymorphism (indels), SNP: Single Nucleotide Polymorphism, cM: centiMorgan.
