# Supplementary figures and images for: A spruce gene map infers ancient plant genome reshuffling and subsequent slow evolution in the gymnosperm lineage leading to extant conifers
Source: BMC Biol. 2012 Oct 26;10:84. doi: 10.1186/1741-7007-10-84 (PMC3519789; doi:10.1186/1741-7007-10-84)

A

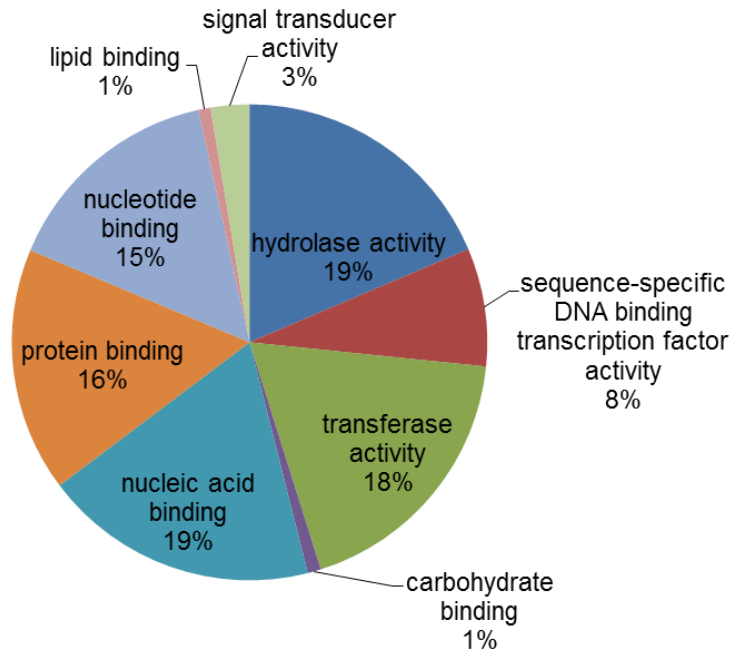

B

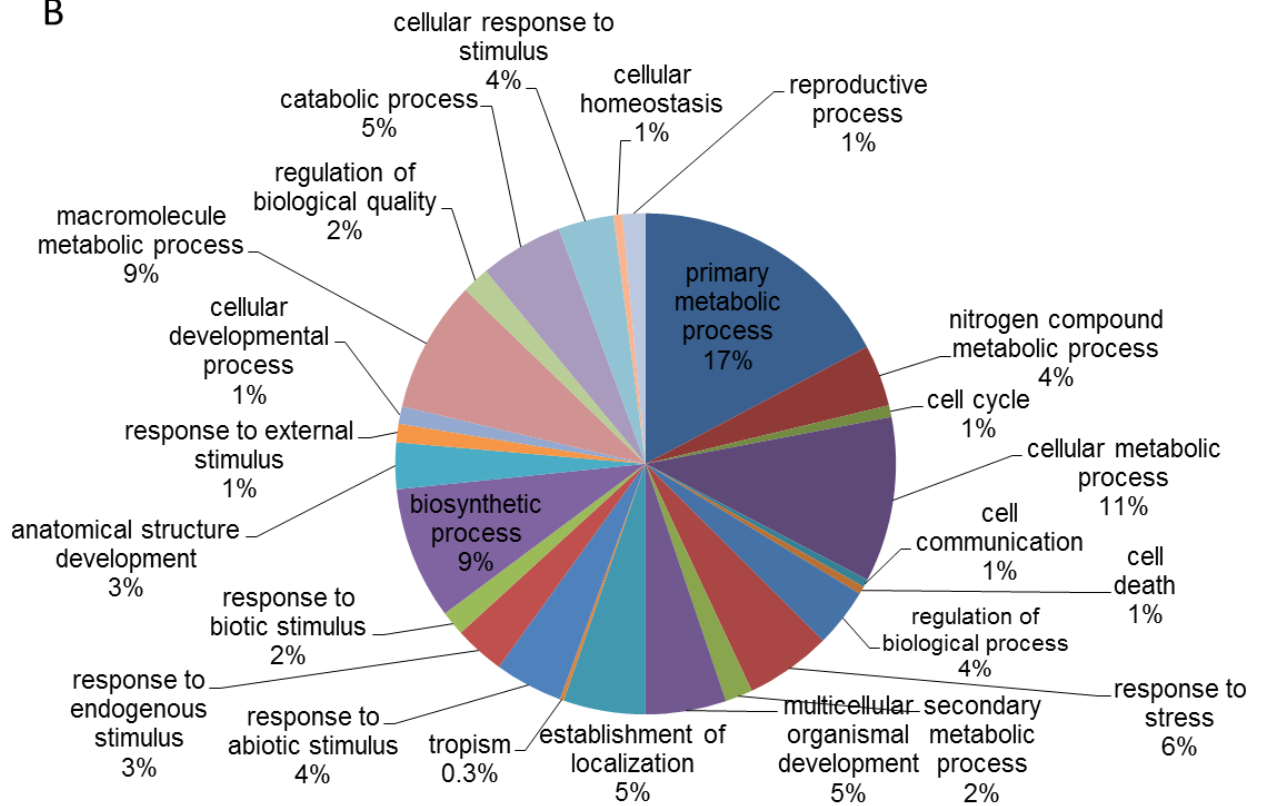

Supplement: Additional file 5 — Gene ontology distribution. Gene ontology terms assigned to the 1,801 mapped spruce genes at the level 3 of the (A) molecular functions and (B) biological processes. Only categories including five genes or more are represented. [file 1741-7007-10-84-S5.PDF]
