# Supplementary material for: A spruce gene map infers ancient plant genome reshuffling and subsequent slow evolution in the gymnosperm lineage leading to extant conifers
Source: BMC Biol. 2012 Oct 26;10:84. doi: 10.1186/1741-7007-10-84 (PMC3519789; doi:10.1186/1741-7007-10-84)
Supplement: Additional file 6 — Gene families and number of genes mapped on the spruce genome for each family. [file 1741-7007-10-84-S6.PDF]

Gene families and number of genes mapped on the spruce genome for each family.

Family	Number of mapped genes
4-coumarate:CoA ligase	2
6b interacting protein	3
ACC oxidase	2
Acid phosphatase	2
Actin related protein	2
acylcoA oxidase	2
Aldo/keto-reductase	6
Ankyrin repeat family	4
AP2 DREB	2
AP2 EREBP (Ethylene Response Factor)	7
AP2 subfamily B1	4
Auxin Response Factor (ARF)	6
Argonaute	5
Armadillo	3
Aspartylprotease	3
ATP-dependent Clp protease	3
Autophagy	3
Aux/iaa	11
Auxin responsive protein	2
Big Apical Meristem (BAM)	4
Band7 protein	2
Bas1	3
bHLH	17
bzip	8
C2C2-Co-like	7
C3H zinc finger (CCCH-type) family protein	8
C3HC4 RING subgroup1.5	2
C3HC4 RING subgroup2.1	3
C3HC4 RING subgroup2.2	4
C3HC4 RING subgroup2.3	2
C3HC4 RING subgroup2.5	4
Caffeoyl-CoA 3-O-methyltransferase	3
Calmodulin binding protein	3
MYB-related (CCA1-like)	4
CCAAT-box binding protein	2
Cellulose synthase	9
Chaperonin	3
Chlorophyll A-B binding protein	3
Cinnamoyl-coA reductase	3
Cyclin	2
Cysteine proteinase	3
Cysteine synthase	2
Cytochrome P450	4
Dehydratation protein	2
Dihydroflavonol 4-reductase	2
Dof-type zinc finger	2
Dormancy/auxin associated family	3

Embryo-abundant protein	3
Endo-1,4-beta-glucanase (KORRIGAN)	4
Endomembrane protein	2
Epimerase	8
Exostocin	4
Expansin alpha	6
Expansin beta	3
Expansin like	2
Fasciclin-like arabinogalactan protein	5
Fructose-1,6-biphosphatase	2
Galactosidase beta	5
Galactosyltransferase	11
Geranylgeranyl pyrophosphate synthase	2
Glycosyl hydrolase 1	4
Glycosyl hydrolase 17	13
Glycosyl hydrolase 18	2
Glycosyl hydrolase 9	6
Gibberellin-regulated protein	2
Glycosyltransferase 48	3
Glycosyltransferase 8	8
GRAS	6
GTP binding protein	2
Haloacid dehalogenase-like hydrolase	4
HD-ZIP 3	5
Heavy metal associated domain containing protein	2
High Mobility Group protein alpha	4
Histone H3	3
Homoeobox leucine zipper protein	2
Homeoboxleucine zipper protein family 5	2
Heat Shock Protein hsp18.1	2
Heat Shock Protein HSP70	3
Heat Shock Protein HSP81	2
Integral membrane Yip1 family	2
KH domain-containing protein	5
Kinase	3
Kinase 2	3
Kinase56720	2
Knox class III	4
Laccase	5
L-ascorbate peroxidase	2
Leunig	4
LIM transcription factor	2
LOB domain protein	2
LRR subgroup 1	6
LRR subgroup 2	10
MADS	5
Major Intrinsic Protein	3
Microtubule EB1	2
Mini Zinc Finger 2 (MIF2)	2
Mitochondrial Import Inner Protein	3
MYB R2R3	26

MYB related (TBP-like)	3
NAM	15
O-methyltransferase family	3
Oxidoreductase	6
Oxygenase	7
Pectate lyase	11
Pectinacetylesterase	2
Pectinesterase	13
Pentatricopeptide (PPR) repeat-containing protein	10
Peroxidase	5
Peroxidase PER12	2
PfkB-type carbohydrate kinase	2
Phenylalanine ammonia-lyase	2
Phosphatase 2C	5
Pinoresinol-lariciresinol reductase	3
Potassium transporter	2
Protease FTSH	2
Proton-dependent oligopeptide transport	2
Pyruvate kinase	2
Quercetin 3-O-methyltransferase	7
Rab2-like GTP-binding protein	2
Ran-binding protein 1	3
Rapid alkalinisation factor	2
Ras-related GTP-binding protein	4
Ribosomal protein	3
RNA recognition motif (RRM)- containing protein	4
sec23/sec24 transport family	2
Senescence-associated protein-related	3
Serine carboxypeptidase S10	5
Serine/threonine protein phosphatase	3
Short-chain dehydrogenase/reductase	7
Spermine synthase	2
Sucrose phosphatase	2
Superoxide dismutase (Cu-Zn)	2
Syntaxin	3
Thioredoxin	5
Tonoplast intrinsic protein	3
trans-cinnamate 4-monooxygenase	2
Transcription factor CBF/NF-Y/archaeal	3
Transducin	10
Transferase	3
TUBBY	8
TubulinAlpha	4
Ubiquitin interaction motif-containing protein / LIM domain-containing protein	2
Ubiquitin conjugating enzyme	5
Ubiquitin-protein ligase	3
UBX domain-containing protein	2
UDP-glucose 6-dehydrogenase	2
VHS domain-containing protein	2
WRKY family transcription factor group I	2

WRKY family transcription factor group IIc	2
Xyloglucan:xyloglucosyl transferase	18
Zinc finger (GATA type)	2
Zinc finger homeobox family protein	4
