# Supplementary material for: A spruce gene map infers ancient plant genome reshuffling and subsequent slow evolution in the gymnosperm lineage leading to extant conifers
Source: BMC Biol. 2012 Oct 26;10:84. doi: 10.1186/1741-7007-10-84 (PMC3519789; doi:10.1186/1741-7007-10-84)
Supplement: Additional file 7 — Over-representation of gene ontology classes in the gene-rich regions based on Fisher exact tests. [file 1741-7007-10-84-S7.PDF]

Over-representation of gene ontology classes in the gene-rich regions based on Fisher exact tests.

Gene ontology ID	Gene ontology term	p-value	Number of genes in GRRs [2]	Number of genes on the overall dataset	Number of genes not annotated in GRRs[2]	Number of genes not annotated in the overall dataset	Over/und[illegible] represente[illegible] term
GO:0048856	anatomical structure development	0.007	15	68	124	1298	over
GO:0016049	cell growth	0.008	8	25	131	1341	over
GO:0090066	regulation of anatomical structure size	0.008	8	25	131	1341	over
GO:0032535	regulation of cellular component size	0.008	8	25	131	1341	over
GO:0008361	regulation of cell size	0.008	8	25	131	1341	over
GO:0009908	flower development	0.010	8	26	131	1340	over
GO:0003006	reproductive developmental process	0.012	8	27	131	1339	over
GO:0048608	reproductive structure development	0.012	8	27	131	1339	over
GO:0005737	cytoplasm	0.013	77	617	62	749	over
GO:0040007	growth	0.019	9	36	130	1330	over
GO:0022414	reproductive process	0.030	8	33	131	1333	over
GO:0005975	carbohydrate metabolic process	0.031	22	138	117	1228	over
GO:0065008	regulation of biological quality	0.031	9	40	130	1326	over
GO:0005622	intracellular	0.035	94	811	45	555	over
GO:0005773	vacuole	0.035	11	55	128	1311	over
GO:0044424	intracellular part	0.040	90	774	49	592	over
GO:0032502	developmental process	0.048	21	137	118	1229	over
GO:0009653	anatomical structure morphogenesis	0.048	10	51	129	1315	over

[1] GRR: Gene-Rich Region
