# Supplementary material for: A spruce gene map infers ancient plant genome reshuffling and subsequent slow evolution in the gymnosperm lineage leading to extant conifers
Source: BMC Biol. 2012 Oct 26;10:84. doi: 10.1186/1741-7007-10-84 (PMC3519789; doi:10.1186/1741-7007-10-84)
Supplement: Additional file 8 — Over-representation of gene ontology classes in the gene arrays based on Fisher exact tests. [file 1741-7007-10-84-S8.PDF]

Over-representation of gene ontology classes in the gene arrays based on Fisher exact tests.

Gene Ontology ID	Gene Ontology Term	p-value	Number of genes in TAGs(2)	Number of genes on the overall dataset	Number of genes not annotated in TAGs(1)	Number of genes not annotated in the overall dataset	Over/Under-represented GO term
GO:0005576	extracellular region	3.9 E-7	18	46	97	1344	over
GO:0005618	cell wall	4.8 E-4	16	70	99	1320	over
GO:0030312	external encapsulating structure	4.8 E-4	16	70	99	1320	over
GO:0019825	oxygen binding	0.006	2	0	113	1390	over
GO:0003677	DNA binding	0.006	24	166	91	1224	over
GO:0016043	cellular component organization	0.007	20	129	95	1261	over
GO:0009719	response to endogenous stimulus	0.016	13	77	102	1313	over
GO:0003676	nucleic acid binding	0.03	29	241	86	1149	over
GO:0019748	secondary metabolic process	0.03	8	41	107	1349	over

(1) TAGs: Tandemly Arrayed Genes
