# Supplementary material for: A spruce gene map infers ancient plant genome reshuffling and subsequent slow evolution in the gymnosperm lineage leading to extant conifers
Source: BMC Biol. 2012 Oct 26;10:84. doi: 10.1186/1741-7007-10-84 (PMC3519789; doi:10.1186/1741-7007-10-84)
Supplement: Additional file 9 — Cluster of co-localizing genes: annotation and expression. We collected expression data from a transcriptomic database covering eight tissues and including mature xylem, juvenile xylem, phelloderm (including phloem), young needles, vegetative buds, megagametophytes, adventitious roots and embryogenic cells [30]. A level of expression was assigned to each tissue and to each gene represented on a microarray. Correlation tests were performed based on the level of expression. Co-expression was declared if the P-value was lower than 0.01 (**) or 0.05 (*). [file 1741-7007-10-84-S9.PDF]

Cluster of co-localizing genes: annotation and expression.

We collected expression data from a transcriptomic database covering eight tissues and including mature xylem, juvenile xylem, phelloderm (including phloem), young needles, vegetative buds, megagametophytes, adventitious roots and embryogenic cells (Raherison et al., unpublished data). A level of expression was assigned to each tissue and to each gene represented on a microarray. Correlation tests were performed based on the level of expression. Co-expression was declared if the p-value was lower than 0.01 (**) or 0.05 (*).

Cluster name	Gene nomenclature	Annotation	Cluster with 2 genes annotated	Cluster with 2 genes from the same family	Cluster with genes from distinct gene families	Cluster with genes from distinct gene families but same functional class	Cluster with no co-expression data available	Cluster of co-expressed genes	Cluster of non-co-expressed genes
Cluster1	Pg_1_4	peroxisomal biogenesis factor					1		
Cluster1	Pg_1_5	unknown function							
Cluster10	Pg_3_8	unknown function					1		
Cluster10	Pg_3_9	unknown function							
Cluster11	Pg_3_45	sodium bile acid	1		1		1		
Cluster11	Pg_3_46	integral membrane yip1 family							
Cluster12	Pg_4_82	unknown function					1		
Cluster12	Pg_4_83	peptidase							
Cluster13	Pg_5_135	rRNA processing protein	1		1		1		
Cluster13	Pg_5_136	GDP-fucose protein O-fucosyltransferase							
Cluster14	Pg_6_33	dehydrin	1		1				
Cluster14	Pg_6_34	tetratricopeptide repeat (TPR)-like superfamily fluorescent in blue light protein							1

Cluster15	Pg_6_69	flavonol synthase	1		1	metabolism	1		
Cluster15	Pg_6_70	glutathione synthase							
Cluster16	Pg_7_96	chalcone synthase	1	1				1 (**)	
Cluster16	Pg_7_97	chalcone synthase							
Cluster17	Pg_7_142	rab5 interacting family protein	1		1		1		
Cluster17	Pg_7_143	chaperone DnaJ-domain superfamily protein							
Cluster18	Pg_7_160	oxidoreductase	1	1			1		
Cluster18	Pg_7_161	oxidoreductase							
Cluster19	Pg_8_12	tonoplast intrinsic protein	1		1	transport	1		
Cluster19	Pg_8_13	cotamer subunit							
Cluster2	Pg_1_66	RING/FYVE/PHD zinc finger superfamily protein	1		1	regulation of transcription			1
Cluster2	Pg_1_67	NAC transcription factor			1				
Cluster21	Pg_8_39	SEUSS transcriptional coregulator	1		1		1		
Cluster21	Pg_8_40	alcohol dehydrogenase							
Cluster22	Pg_8_88	calcineurin b-like protein	1		1			1 : Pg_8_88 and Pg_8_89 co-expressed (*)	
Cluster22	Pg_8_89	citrate synthase							
Cluster22	Pg_8_90	phospholipd translocating ATPase							
Cluster23	Pg_8_103	30S ribosomal protein	1		1				
Cluster23	Pg_8_104	glyceraldehyde 3P dehydrogenase				Pg_8_104 and			

						Pg_8_105 : metabolism			
Cluster23	Pg_8_105	cytochrome b561/ferric reductase						1: Pg_8_103 and Pg_8_105 co-expressed (*)	
Cluster24	Pg_8_175	WRKY transcription factor	1		1		1		
Cluster24	Pg_8_176	synaptosomal associated protein							
Cluster25	Pg_9_102	transcriptional coactivator	1		1		1		
Cluster25	Pg_9_103	oxoglutarate/iron-dependent oxygenase							
Cluster26	Pg_10_4	NAC transcription factor	1		1	regulation of transcription			1
Cluster26	Pg_10_5	PHD transcription factor							
Cluster27	Pg_10_102	F box family					1		
Cluster27	Pg_10_103	unknown function							
Cluster28	Pg_11_5	NAD linked oxidoreductase-like protein	1				1		
Cluster 28	Pg_11_6	chlorophyll a b binding protein							
Cluster29	Pg_11_140	pectin methylesterase	1	1			1		
Cluster29	Pg_11_141	pectin methylesterase							
Cluster3	Pg_1_113	tubby tanscription factor							1
Cluster3	Pg_1_114	unknown function							
Cluster30	Pg_12_26	unknown function							1
Cluster30	Pg_12_27	MADS	1		1				

		transcription factor					
Cluster30	Pg_12_28	RNA recognition motif-containing protein					
Cluster31	Pg_12_36	unknown function				1	
Cluster31	Pg_12_37	protein family containing a PDZ, a K-box, and a TPR motif					
Cluster32	Pg_12_64	aux/iaa	1	1		1	
Cluster32	Pg_12_65	aux/iaa					
Cluster33	Pg_12_121	RNA-binding (RRM/RBD/RNP motifs) family protein	1		1	1	
Cluster33	Pg_12_122	protein-tyrosine phosphatase-like					
Cluster4	Pg_1_153	ubiquitin-like superfamily	1		1		1
Cluster4	Pg_1_154	homeobox protein					
Cluster5	Pg_2_8	EXORDIUM like	1		1		1
Cluster5	Pg_2_9	calreticulin					
Cluster6	Pg_2_13	twin-arginine translocation pathway	1		1	1	
Cluster6	Pg_2_14	XAP5 family protein					
Cluster7	Pg_2_36	2-oxoglutarate (2OG) and Fe(II)-dependent oxygenase	1		1	1	
Cluster7	Pg_2_37	RNA-binding KH domain-containing protein					

Cluster8	Pg_2_38	autophagy 3	1		1	1		
Cluster8	Pg_2_39	tubulin alpha-5						
Cluster9	Pg_2_73	tonoplast integral protein	1	1		1		
Cluster9	Pg_2_74	tonoplast integral protein						
Total			26	5	21	22	3	7
