# Supplementary material for: A spruce gene map infers ancient plant genome reshuffling and subsequent slow evolution in the gymnosperm lineage leading to extant conifers
Source: BMC Biol. 2012 Oct 26;10:84. doi: 10.1186/1741-7007-10-84 (PMC3519789; doi:10.1186/1741-7007-10-84)
Supplement: Additional file 10 — Conservation between the chromosomes from Picea and Pinus taeda or Pinus pinaster. [file 1741-7007-10-84-S10.PDF]

Conservation between the chromosomes from *Picea* and *Pinus taeda* or *Pinus pinaster*

Picea chromo-some	*Pinus taeda* (Pt) *or Pinus pinaster* (Pp) homeologous chromosome	Orthologous gene pairs (1)	Non orthologous gene pairs(2)	Mean length of the *Pinus*/*Picea* sequence alignment (nt)	Conserved segment in *Picea* (cM) (4)	*Picea* chromoso-me (cM) (5)	Ratio (4)/(5)	Conserved segment in *Pinus* chromoso-me (cM) (6)	*Pinus* chromoso-me (cM) (7)	Ratio (6)/(7)	Collinear homologous pairs (% of the total of orthologous pairs)
1	Pt-9	11	0	970	164.4	180.6	91.0%	137.8	168.2	81.9%	9 (81.8%)
2	Pt-8	19	0	889	149.9	188.3	79.6%	163.3	170.9	95.6%	19 (100%)
3	Pt-2	20	0	1096	165.8	178.8	92.7%	161.3	164.8	97.9%	20 (100%)
4	Pt-3	8	0	682	149.4	168.3	88.7%	156.7	161.7	96.9%	7 (87.5%)
5	Pt-12	19	0	964	143.2	169.3	84.6%	145.2	168.0	86.4%	14 (73.7%)
6	Pt-7	11	1	734	154.2	185.5	83.1%	150.5	167.7	89.7%	11 (100%)
7	Pt-11	7	0	948	129.2	204.5	63.2%	77.2	174.7	44.2%	6 (85.7%)
8	Pt-6	18	1	930	145.3	172.2	84.4%	144.7	177.7	81.4%	14 (77.8%)
9	Pt-5	15	1	1112	141.3	145.9	96.9%	169.2	172.8	97.9%	12 (80.0%)
10	Pt-4	12	1	1049	118.9	125.8	94.5%	122.3	131.3	93.1%	10 (83.3%)
11	Pt-1	6	0	1287	80.1	175.5	45.6%	92.0	100.4	91.6%	6 (100%)
12	Pt-10	15	0	891	119.6	131.7	90.8%	122.6	140.4	87.3%	15 (100%)
Total for *P. taeda*		161	4	11,552	1661.3	2026.4	82.0%	1642.8	1898.6	86.5%	143 (88.8%)
Average for *P. taeda*		13.4	0.3	963	138.4	168.9	-	136.9	158.2	-	11.9(88.8%)
1	Pp-1	1	0	121	0	180.6	0 %	0	87.3	0 %	0
2	Pp-5	5	1	674	146.6	188.3	77.8 %	66.5	133.3	49.9%	5 (100%)
3	Pp-7	5	1	628	88.8	178.8	49.7 %	58.9	93.2	63.2%	5 (100%)
4	Pp-12	5	1	697	110.1	168.3	65.4 %	68.0	145.3	46.8%	5 (100%)
5	Pp-10	4	0	1158	139.0	169.3	82.1 %	125.4	125.9	99.6%	3 (75%)
6	Pp-8	3	1	374	98.6	185.5	53.1 %	64.3	108.1	59.5%	2 (66.7%)
7	Pp-2	4	0	712	54.7	204.5	26.8 %	47.7	134.8	35.4%	4 (100%)
8	Pp-4*	6	1	913	148.1	172.2	86.0 %	144.1	154.9	93.0%	5 (83.3%)
9	Pp-6	10	1	667	123.0	145.9	84.4 %	97.4	115.4	84.4%	4 (40%)
10	Pp-11	4	0	1158	67.2	125.8	53.5 %	46.4	107.2	43.3%	3 (75%)
11	Pp-3	11	0	625	116.1	175.5	66.1 %	59.5	83.6	71.2%	6 (54.5%)
12	Pp-9	4	0	712	5.6	131.7	4.3 %	5.0	108.5	4.6%	2 (50%)
Total for *P. pinaster*		63	6	7587	1097.8	2026.4	54.2 %	783.2	1397.5	56.0%	45 (71.4%)
Average for *P. pinaster*		5.3	0.5	632.3	91.5	168.9		65.3	116.5		3.8

(1) with an identity level>80%

(2) gene pairs sharing a sequence identity >80% but hit found on another chromosome
