# Supplementary material for: A spruce gene map infers ancient plant genome reshuffling and subsequent slow evolution in the gymnosperm lineage leading to extant conifers
Source: BMC Biol. 2012 Oct 26;10:84. doi: 10.1186/1741-7007-10-84 (PMC3519789; doi:10.1186/1741-7007-10-84)
Supplement: Additional file 11 — Coordinates and annotation of the conserved genes found on homoeologous Picea and Pinus chromosomes. [file 1741-7007-10-84-S11.PDF]

Coordinates and annotation of the conserved genes found on homoeologous *Picea* and *Pinus* chromosomes.

Pinus pinaster			*Picea*			*Pinus taeda*			
Chromosome	Gene	Position (in cM)	Chromosome	Gene	Position (in cM)	Chromosome	Gene	Position (in cM)	Functional annotation
			11	pg_11_5	7.01	1	snp0-18261-01-455	96.1	NAD(P)-linked oxidoreductase superfamily
3	m685		11	pg_11_6	7.01				chlorophyll A-B binding protein
			11	pg_11_17	15.00	1	snpCL599Contig1-07-109	91.2	transcription factor CBF/NF-Y/archaeal
			11	pg_11_26	35.19	1	snp2-8585-02-472	65.8	CCAAT-HP3
			11	pg_11_30	46.56	1	snp0-1126-02-419	34.6	NAM
3	m969	0	11	pg_11_43	59.47				ribosomal 60S
			11	pg_11_61	85.82	1	snpUMN-6664-01-168	21.5	hairpin-induced protein
			11	pg_11_65	87.12	1	snp0-14961-01-327	4.1	phosphatase
3	m1339	41.3	11	pg_11_84	96.51				trans-cinnamate 4-monooxygenase / cinnamic acid 4-hydroxylase
3	PtNCS_22B8	41.4	11	pg_11_86	96.94				glycine hydroxymethyltransferase
3	m696	59.5	11	pg_11_102	125.68				fasciclin-like arabinogalactan-protein
3	m640	59	11	pg_11_111	140.53				cinnamoyl-CoA reductase-related
3	CesA3		11	pg_11_114	143.58				cellulose synthase
3	m73	37.9	11	pg_11_133	167.28				bZIP
3	m585	37.9	11	pg_11_136	167.68				unknown
3	m298	33.6	11	pg_11_138	169.23				S-adenosylmethionine synthetase
3	m376	37.9	11	pg_11_147	175.52				histone
7	m738	0 (sub-gp1)	3	pg_3_2	9.30	2	snpCL711Contig1-04-212	3.5	LIM transcription factor
			3	pg_3_10	20.21	2	snp0-9488-01-346	12.4	homeodomain protein
			3	pg_3_11	20.60	2	snpCL1259Contig1-03-442	12.4	flowering time control protein

			3	pg_3_26	51.31	2	snpCL3765Contig1-01-99	35.8	endo-1,4-beta-glucanase
			3	pg_3_38	65.11	2	snp0-7662-01-189	50.1	unknown
			3	pg_3_44	72.12	2	snpUMN-3841-01-481	59.5	calmodulin-binding protein
			3	pg_3_46	75.77	2	snp2-5858-01-255	61.6	integral membrane Yip1 family
7	m429	45,7 (sub-gp1)	3	pg_3_49	83.34				unknown
7	m108	46,3 (sub-gp1)	3	pg_3_53	91.22				chloroplast nucleoid DNA-binding protein
			3	pg_3_67	99.21	2	snp2-9790-01-129	83.7	unknown
7	PtIFG_9151	58,9 (sub-gp1)	3	pg_3_63	98.08	2	estPitaIFG-9151-a	86.9	plastocyanin-like
			3	pg_3_71	103.08	2	snpCL4668Contig1-01-281	89.5	calcium-binding EF hand family
			3	pg_3_72	103.47	2	snpUMN-3554-01-437	89.5	MYB (R2R3) subgroup8
			3	pg_3_78	108.35	2	snpCL1210Contig1-01-396	92.4	phosphoglycerate kinase
			3	pg_3_82	111.35	2	estPitaIFG-4CL-a	97.2	4-coumarate-CoA ligase
			3	pg_3_89	113.63	2	snp2-3650-01-68	101.5	AUX-IAA
			3	pg_3_101	130.38	2	snpUMN-1266-01-156	119.2	0-fucosyltransferase
			3	pg_3_145	173.20	2	snp0-13832-01-383	154.8	unknown
			3	pg_3_136	167.96	2	snp0-8538-01-252	158.1	C3HC4-type RING finger
			3	pg_3_143	172.13	2	snpCL1662Contig1-06-43	161.5	phytanoyl-CoA dioxygenase
			3	pg_3_148	175.05	2	snpCL918Contig1-04-174	161.5	bZIP
7	m694	1,7 (sub-gp3)	3	pg_3_146	174.16				structural constituent of cytoskeleton
			3	pg_3_147	174.30	2	snpCL3375Contig1-01-115	164.8	unknown
			4	pg_4_1	0	3	snpUMN-738-01-522	0	MYB (R2R3) pgmyb4
			4	pg_4_24	13.14	3	snpCL1615Contig1-02-162	29.4	unknown
12	m186	30.6	4	pg_4_25	16.99				pyrophosphate-fructose
			4	pg_4_41	43.66	3	snp0-11443-01-656	40.7	LOB domain protein
			4	pg_4_43	46.63	3	snp0-9203-03-40	50	unknown

			4	pg_4_48	54.90	3	snp0-14740-01-384	43.3	homeotic protein
			4	pg_4_81	88.70	3	snp2-1954-02-464	101.3	acid phosphatase
			4	pg_4_92	95.55	3	snp0-2097-02-460	106.8	expansin beta
12	m1039	88.3	4	pg_4_95	96.15				structural constituent of ribosome
12	m859	89.4	4	pg_4_96	96.41				thylakoid lumenal 17.4 kDa protein
12	m1495	97.9	4	pg_4_103	103.65				ABC transporter family protein
12	m721	98.6	4	pg_4_120	127.06				protein serine/threonine kinase
			4	pg_4_137	149.42	3	snp2-9454-01-416	156.7	bHLH
			10	pg_10_2	5.86	4	snpCL2055Contig1-03-78	127.1	band 7 family protein
11	m8	0 cM on F map for G2 (position of m441)	10	pg_10_6	7.62	4	snp0-11649-03-90	130.2	tubulin beta
11	m319	1.3	10	pg_10_10	8.59				
			10	pg_10_14	10.47	4	snp2-1095-01-608	129.1	fructose-1,6-bisphosphatase
			10	pg_10_16	11.69	4	snp2-6782-01-203	131.3	flower pigmentation protein
11	m732	43.9	10	pg_10_71	68.54	4	snp2-2260-01-217	63.6	endo-1,4-beta-glucanase KORRIGAN
			10	pg_10_72	69.29	4	snp2-3634-01-464	65.3	WD-40 repeat family protein
			10	pg_10_76	71.27	4	snpCL4046Contig1-02-273	61.5	splicing factor RSZ33
11	m605	47.7	10	pg_10_79	74.86	4	snpCL685Contig1-03-124	58.6	S-adenosyl-methionine-sterol-C-meth
			10	pg_10_98	84.24	4	snp2-10481-01-923	48.9	SEC14 cytosolic factor related
			10	pg_10_108	85.75	4	snpCL4023Contig1-01-244	131.3	tryptophan synthase
			10	pg_10_123	114.38	4	snp0-7479-01-104	26.4	proton-dependent oligopeptide transport
			10	pg_10_133	124.73	4	snpCL2083Contig1-01-471	9	bZIP
			9	pg_9_2	1.49	5	snp2-3236-01-224	168.3	fasciclin-like arabinogalactan-protein
			9	pg_9_10	7.17	5	snp2-6664-01-302	172.8	AP2-EREBP
6	m895	102	9	pg_9_11	7.65				phototropic-responsive NPH3 family
			9	pg_9_15	12.65	5	snp0-10955-01-469	154.2	C3HC4-type RING finger cluster 1.5

6	m723	105	9	pg_9_17	14.28	5	snpCL4011Contig1-03-102	157.4	HD-ZIP3
6	m328	100.8	9	pg_9_25	22.81				plasma membrane intrinsic protein
			9	pg_9_29	26.16	5	snp0-14003-01-677	140.9	unknown
6	m1185	96.4	9	pg_9_33	27.51				luminal binding protein
6	PtIFG_8972	83.1	9	pg_9_36	28.951				aquaporin
6	m962	95.5	9	pg_9_37	29.89				patatin
			9	pg_9_38	30.72	5	snpCL1837Contig1-01-474	132.8	ubiquitin-protein ligase
6	m104	86.4	9	pg_9_44	35.51				ABC transporter family
			9	pg_9_53	43.60	5	snp0-4517-01-461	121.7	MYB (R2R3) subgroup2
			9	pg_9_55	47.06	5	snpCL4153Contig1-03-132	122.3	nascent polypeptide associated complex
6	PAL	71	9	pg_9_56	51.78				phenylalanine ammonia-lyase
			9	pg_9_70	62.79	5	estPitaIFG-1950-a	85.1	metallothionein
			9	pg_9_85	70.86	5	estPitaIFG-0739-a	51.9	phospholipase
			9	pg_9_123	125.63	5	snp0-8844-01-456	34.6	glycosyl transferase 8
6	m153	11.6	9	pg_9_125	129.39	5	snpCL544Contig1-03-112	32.7	caffeoyl-CoA 3-O-methyltransferase
6	PtIFG_606	7.6	9	pg_9_127	130.69				zinc finger family protein
			9	pg_9_129	133.51	5	snpUMN-CL64Contig1-03-391	25.3	unknown
			9	pg_9_142	142.81	5	snp2-4617-01-196	3.6	pectate lyase
			9	pg_9_140	142.41	5	snp0-5855-01-115	19	phosphorylase
4	m314	123,2 cM on M map for G2 (position of m562)	8	pg_8_1	14.78				
			8	pg_8_10	18.39	6	snp0-18679-01-146	166.3	LEA
			8	pg_8_17	20.10	6	snp0-12515-01-612	154.2	serine carboxypeptidase S10
			8	pg_8_18	20.26	6	snp0-9989-01-389	154.2	homoserine kinase
			8	pg_8_19	20.26	6	snp2-985-01-70	166.1	peroxisomal membrane protein

			8	pg_8_22	23.10	6	snp2-3307-01-40	154.2	pyruvate dehydrogenase E1 alpha subunit
4	m667	154,9 cM on F map for G2 (position of m562)	8	pg_8_26	29.83				dirigent-like protein
			8	pg_8_50	60.23	6	snpCL2359Contig 1-03-329	135.4	betaine-aldehyde dehydrogenase
			8	pg_8_51	62.86	6	snp2-6534-01-218	122.5	unknown
			8	pg_8_56	68.76	6	snpCL4511Contig 1-02-240	122.7	unknown
			8	pg_8_60	73.30	6	snp0-9030-01-175	128.7	glycosyl transferase 8
			8	pg_8_72	85.59	6	snp0-1646-01-86	101.5	zinc finger homeobox family protein
			8	pg_8_76	89.18	6	snpUMN-7049-02-156	101.5	aspartyl protease
			8	pg_8_106	106.30	6	snp2-297-02-160	79	MYB (R2R3)
			8	pg_8_134	144.85	6	snp2-1264-01-111	40.7	glycosyl hydrolase 17
			8	pg_8_142	153.42	6	snpUMN-1037-01-401	35.7	unknown
4	m138	14.2	8	pg_8_146	154.46				unknown
			8	pg_8_151	157.01	6	estPitaIFG-8738-a	30.4	mitogen-activated protein kinase
			8	pg_8_162	160.89	6	estPitaIFG-2N7G-a	26	tubulin alpha
4	PtIFG _8898	8	8	pg_8_163	161.67				WD-40 repeat family protein
4	PtIFG _1584	8.1	8	pg_8_164	162.49				aldo/keto reductase
4	m593	9.1	8	pg_8_166	162.88				L-ascorbate peroxidase
			8	pg_8_168	163.23	6	snpCL2076Contig 1-04-211	27.6	protease FTSH
			8	pg_8_169	163.74	6	snp0-11781-01-254	21.6	epimerase/dehydratase
8	m760	20.2	6	pg_6_1	5.18				MYB transcription factor
			6	pg_6_17	20.77	7	snp2-4291-01-38	158.5	coproporphyrinogen III oxidase
			6	pg_6_23	26.20	7	snpCL756Contig1-03-84	148.9	homeobox family
8	m507	0	6	pg_6_33	43.90				dehydrin
			6	pg_6_34	43.90	7	snpCL3568Contig 1-03-235	128.2	TPR domain containing protein
			6	pg_6_35	43.90	7	snp0-5963-02-323	124.2	SNARE associated golgi protein

			6	pg_6_38	45.49	7	snpCL1188Contig 1-03-72	118.6	ubiquitin-protein ligase
			6	pg_6_63	83.79	7	snp2-6413-01-494	91.3	LEUNIG transcription factor
			6	pg_6_77	95.01	7	snp0-9182-01-120	84	membrane protein (PMP)
8	m493	64.3	6	pg_6_90	103.75	7	snpCL3539Contig 1-01-306	76.7	VHS domain-containing protein
			6	pg_6_115	132.05	7	snp2-2658-02-59	57.6	oxidoreductase
			6	pg_6_131	169.06	7	snpCL1365Contig 1-03-54	9.8	nuclear matrix constituent protein
			6	pg_6_135	174.93	7	snp0-13024-01-121	8	zinc finger (CCCH-type) family protein
5	m189	67,5 (sub-gp 2)	2	pg_2_16	17.40				
			2	pg_2_26	31.85	8	snp0-6427-02-341	158	magnesium transporter
			2	pg_2_30	36.87	8	snp2-9280-02-107	158	DNA-binding protein
			2	pg_2_33	37.14	8	snp2-4723-01-197	159.6	coatomer protein complex--famille1
5	m682	61 (sub-gp 2)	2	pg_2_34	37.77				Ras-related GTP-binding protein
			2	pg_2_35	39.35	8	snpCL560Contig1-03-130	164.1	tubulin alpha
			2	pg_2_42	42.53	8	snp2-292-01-138	164.1	nucleotidyltransferase
			2	pg_2_46	48.32	8	snp2-2936-01-185	170.9	AUX-IAA
			2	pg_2_58	62.66	8	snp2-2199-01-680	124.9	ion channel
			2	pg_2_64	72.91	8	snpCL4662Contig 1-01-215	112.1	unknown
			2	pg_2_65	92.91	8	snpUMN-CL373Contig1-07-619	116.9	acetylesterase
5	m991	27,9 (sub-gp 2)	2	pg_2_75	78.40				receptor-like kinase
5	PtIFG _893	1 (sub-gp 2)	2	pg_2_92	96.99	8	estPitaIFG-0893-a	92.1	lipid transfer protein
			2	pg_2_96	98.52	8	snp2-6080-01-266	96.3	transducin
			2	pg_2_114	114.50	8	snpCL1798Contig 1-04-56	85.8	lipid transfer protein
			2	pg_2_120	123.43	8	snp0-10054-01-402	67.1	NAM
			2	pg_2_137	147.24	8	snpCL3758Contig 1-05-475	43.5	translation initiation factor
			2	pg_2_139	147.79	8	snpCL2299Contig 1-03-323	42	ribosomal 40S

			2	pg_2_140	149.33	8	snpCL314Contig1-03-176	43.5	peroxidase PER12
			2	pg_2_151	155.71	8	snpCL3196Contig1-01-130	30.3	argonaute
5	m964	4,6 (sub-gp 1)	2	pg_2_162	163.99				thylakoid lumenal protein
			2	pg_2_165	169.96	8	snp2-7820-02-137	15.2	hydroxyproline-rich glycoprotein1
			2	pg_2_174	181.79	8	snp0-2313-02-181	7.6	phosphoribulokinase
			1	pg_1_15	13.75	9	snpCL199Contig3-02-149	144.5	AWPM-19-like membrane family
1	m495	68	1	pg_1_28	25.71				high mobility group protein alpha
			1	pg_1_44	52.63	9	snpCL3054Contig1-01-82	117.5	KH domain-containing protein
			1	pg_1_48	63.70	9	snpCL1400Contig1-02-144	30.9	ribosomal S5
			1	pg_1_52	69.75	9	snpCL1894Contig1-03-38	114.6	GDP-L-galactose-hexose-1-phosphate guanyltransferase
			1	pg_1_67	88.30	9	estPitaIFG-1576-a	70.5	lil protein -Chlorophyll A-B binding family protein
			1	pg_1_94	105.03	9	snp0-7494-02-313	73.9	calmodulin-binding protein
			1	pg_1_103	114.58	9	snp0-12156-02-134	73.9	kinase
			1	pg_1_109	120.34	9	snp0-15417-01-138	63.3	Transducin/WD repeat family
			1	pg_1_130	139.86	9	snp0-6709-02-588	42.2	WD repeat protein
			1	pg_1_158	168.01	9	snp0-13608-01-303	7.7	PPR
			1	pg_1_166	178.10	9	snp0-15011-02-197	6.7	RRM
			12	pg_12_10	3.75	10	snp0-13026-02-330	9.3	C3HC4-type RING finger
			12	pg_12_16	11.29	10	estPitaIFG-1956-a	11.8	ubiquitin-protein ligase
			12	pg_12_20	14.38	10	snpCL647Contig1-04-50	11.8	C3HC4-type RING finger
			12	pg_12_24	16.043	10	snpCL1451Contig1-02-60	22	transducin/WD repeat family
			12	pg_12_71	69.64	10	snp2-1638-01-77	77.9	transcription factor IIA
			12	pg_12_84	73.95	10	snp0-17881-01-276	87.8	exocyst subunit EXO70 family
			12	pg_12_83	73.74	10	snpCL2431Contig	87.8	unknown

							1-01-459		
			12	pg_12_97	96.15	10	snpCL2416Contig1-06-360	99.2	glutathione peroxidase
9	m807	20.7	12	pg_12_101	103.16	10	snp0-13455-01-122	106.3	MYB (R2R3) subgroup 22
9	m976	21	12	pg_12_102	103.44	10	snp2-9930-01-56	108.2	GRAS
			12	pg_12_111	107.47	10	snpCL305Contig1-05-249	116.3	dihydrolipoamide dehydrogenase
9	PtIFG_8415	16	12	pg_12_113	108.19	10	estPitaIFG-8415-a	117.6	short-chain dehydrogenase/reductase
9	CAD	17.7	12	pg_12_114	108.79				cinnamyl-alcohol dehydrogenase
			12	pg_12_120	121.72	10	snpUMN-5911-02-179	129.6	beta-ureidopropionase
			12	pg_12_125	123.31	10	snp2-1534-02-96	130.8	SNF2 domain-containing protein
			12	pg_12_118	121.17	10	snpUMN-4383-01-585	131.9	arginine N-methyltransferase
			7	pg_7_41	67.28	11	snp0-3203-01-231	48	MYB (R2R3)
2	m553	118.5	7	pg_7_23	46.09				subtilase family protein
			7	pg_7_44	67.85	11	snp0-14613-01-110	48	MYB (R2R3)
2	m1026	95.7	7	pg_7_45	68.22				MYB (R2R3)
2	PtIFG_464	82.6	7	pg_7_52	77.26				plasma membrane intrinsic protein
2	m414	70.8	7	pg_7_78	100.83				pfkB-type carbohydrate kinase
			7	pg_7_81	104.22	11	snp2-8315-02-245	78.9	serine carboxypeptidase S10
			7	pg_7_103	132.03	11	snp0-1576-01-407	51.6	Xyloglucan:xyloglucosyl transferase
			7	pg_7_143	185.13	11	snp2-1808-02-59	12.6	DNAJ heat shock
			7	pg_7_148	188.36	11	snp2-10306-01-334	9.1	unknown
			7	pg_7_150	196.47	11	snp2-945-01-77	1.7	tubulin alpha
10	m450	22,6 (sub-gp3)	5	pg_5_6	5.52				heat shock protein
			5	pg_5_9	6.91	12	snpCL4264Contig1-01-164	162.1	C3HC4-type RING finger
			5	pg_5_21	21.04	12	snp2-3140-01-95	157.6	LEUNIG transcription factor
			5	pg_5_22	21.41	12	snp2-8381-01-116	158.2	WRKY transcription factor
			5	pg_5_32	33.82	12	snp2-7803-01-212	141	polygalacturonase

			5	pg_5_38	41.99	12	snp0-9314-01-64	146.9	unknown
			5	pg_5_41	50.71	12	snp0-17893-01-332	133.2	G2-like
10	m359	0 (sub-gp2)	5	pg_5_47	57.58				60S ribosomal protein
			5	pg_5_61	72.283	12	snp2-10071-02-104	108.1	flavin reductase
	PtIFG_1643	0 (sub-gp1)	5	pg_5_68	82.50				protein phosphatase 2C
			5	pg_5_71	83.87	12	snp2-1402-02-596	94.9	chaperonin
			5	pg_5_76	85.75	12	snp2-2356-02-88	92.8	maternal effect embryo arrest protein
			5	pg_5_80	91.36	12	snp0-10113-01-119	84	unknown
			5	pg_5_82	91.73	12	snpUMN-CL306Contig1-04-261	86.6	actin-depolymerizing factor
			5	pg_5_88	95.90	12	snpCL996Contig1-03-66	74.9	xylosidase
			5	pg_5_92	99.17	12	snp2-6387-01-711	69	alpha-galactosidase
10	PpINR_AS01H04	39,3 (sub-gp1)	5	pg_5_93	100.72				ribulose bisphosphate carboxylase
			5	pg_5_96	104.34	12	snp0-7512-01-312	65.4	bZIP
			5	pg_5_112	128.10	12	snpCL64Contig1-07-134	46.7	heat shock protein 70
10	PtIFG_8580	13,5 (sub-gp1)	5	pg_5_125	144.47	12	estPitaIFG-8580-a	26.6	late embryogenesis abundant protein
			5	pg_5_128	146.04	12	snp0-5784-01-234	14.8	C3HC4-type RING finger
			5	pg_5_135	148.68	12	snpCL4330Contig1-03-120	14.7	rRNA processing protein
			5	pg_5_137	148.88	12	estPitaIFG-8580-a	26.6	ABA-responsive LEA-like protein
			5	pg_5_141	150.08	12	snp0-17253-02-86	16.9	glycosyl hydrolase 9
