# Supplementary material for: A spruce gene map infers ancient plant genome reshuffling and subsequent slow evolution in the gymnosperm lineage leading to extant conifers
Source: BMC Biol. 2012 Oct 26;10:84. doi: 10.1186/1741-7007-10-84 (PMC3519789; doi:10.1186/1741-7007-10-84)
Supplement: Additional file 12 — Unrooted majority-rule bootstrap trees obtained with the neighbour-joining (NJ) and the maximum parsimony (MP) methods for 157 gene families of seed plants. [file 1741-7007-10-84-S12.PDF]

4CL - NJ

```
                                                        +------Pt81267 41
                                                 +433.0-|
                                                 |      +------pg6 86
                                          +500.0-|
                                          |      |      +------Pt53499 23
                                          |      +472.0-|
                                   +479.0-|             +------Pt30285 16
                                   |      |
                                   |      |             +------Pt90982550
                            +272.0-|      +-------500.0-|
                            |      |                    +------pg3 82
                            |      |
                            |      |                    +------AT1G65060.
                     +491.0-|      +--------------476.0-|
                     |      |                           +------Os02g46970
                     |      |
                     |      |                           +------Os06g44620
              +303.0-|      |                    +494.0-|
              |      |      +--------------425.0-|      +------Os02g08100
              |      |                           |
       +414.0-|      |                           +-------------Os08g14760
       |      |      |
       |      |      +-----------------------------------------Os08g34790
+------|      |
|      |      +------------------------------------------------AT3G21230.
|      |
|      +-------------------------------------------------------AT3G21240.
|
+--------------------------------------------------------------AT1G51680.
```

4CL - PARS

```
                                                 +------Pt30285 16
                                          +382.8-|
                                   +330.0-|      +------Pt53499 23
                                   |      |
                            +494.0-|      +-------------Pt81267 41
                            |      |
                     +262.7-|      +--------------------pg6 86
                     |      |
                     |      |                    +------Pt90982550
                     |      +--------------489.2-|
              +350.0-|                           +------pg3 82
              |      |
              |      |                           +------AT1G65060.
              |      |             +-------406.8-|
              |      |             |             +------Os02g46970
              |      +-------279.1-|
              |                    |      +-------------Os08g14760
       +293.5-|                    +418.9-|
       |      |                           |      +------Os02g08100
       |      |                           +494.7-|
       |      |                                  +------Os06g44620
+------|      |
|      |      +-----------------------------------------Os08g34790
|      |      |
|      |      +-----------------------------------------AT1G51680.
|      |
|      +------------------------------------------------AT3G21240.
|
+-------------------------------------------------------AT3G21230.
```

6b interacting protein - NJ

```
                                   +------Pt6004 29
                            +500.0-|
                            |      +------pg5 120 27
                     +258.0-|
                     |      |      +------AT3G11100
                     |      +331.0-|
                     |             +------AT5G05550
                     |
                     +--------------------pg9 9 20
                     |
                     +--------------------Os01g52090
                     |
                     |             +------AT3G10030
                     |      +449.0-|
                     +390.0-|      +------Os09g38570
                     |      |
              +465.0-|      +-------------AT3G54390
              |      |
              |      |             +------Os04g36790
              |      +-------410.0-|
              |      |             +------Os02g35690
              |      |
              |      +--------------------AT3G58630
              |      |
       +435.0-|      |             +------Os01g48320
       |      |      +-------396.0-|
       |      |      |             +------Os05g48690
       |      |      |
       |      |      +--------------------Pt42175 20
+------|      |      |
|      |      |      +--------------------AT3G14180
|      |      |
|      |      +---------------------------AT3G24490
|      |
|      +----------------------------------Pt56876 23
|
+-----------------------------------------pg8 122 21
```

6b interacting protein - PARS

```
                                                 +------AT3G54390
                                          +337.1-|
                                   +285.0-|      +------Pt42175 20
                                   |      |
                            +337.9-|      +-------------Os09g38570
                            |      |
                     +300.5-|      +--------------------AT3G10030
                     |      |
                     |      +---------------------------Os01g52090
                     |
                     +----------------------------------pg9 9 20
                     |
                     |                           +------Pt6004 29
                     +---------------------500.0-|
                     |                           +------pg5 120 27
                     |
                     |                           +------Pt56876 23
              +450.7-|                    +409.3-|
              |      +--------------329.1-|      +------pg8 122 21
              |      |                    |
              |      |                    +-------------AT3G24490
              |      |
              |      |                           +------AT3G11100
              |      +---------------------493.6-|
       +500.0-|      |                           +------AT5G05550
       |      |      |
       |      |      |                           +------Os05g48690
       |      |      |                    +477.6-|
       |      |      +--------------296.6-|      +------Os01g48320
+------|      |                           |
|      |      |                           +-------------AT3G58630
|      |      |
|      |      +-----------------------------------------AT3G14180
|      |
|      +------------------------------------------------Os04g36790
|
+-------------------------------------------------------Os02g35690
```

ACC oxidase - NJ

```
                            +---------------------------pg 10 128
       +--------------493.0-|
       |                    |      +--------------------Pt74382551
       |                    +500.0-|
       |                           |      +-------------Pp3088 27
       |                           +258.0-|
       |                                  |      +------Pt10288254
       |                                  +290.0-|
+------|                                         +------Pt74282550
|      |
|      |      +-----------------------------------------AT2G19590
|      |      |
|      |      |                           +-------------AT1G05010
|      |      |                    +282.0-|
|      |      |                    |      |      +------AT1G62380
|      |      |                    |      +500.0-|
|      +500.0-|             +126.0-|             +------AT1G12010
|             |             |      |
|             |             |      |             +------Os02g53180
|             |             |      |      +390.0-|
|             |      +-63.0-|      +382.0-|      +------Os09g27820
|             |      |      |             |
|             |      |      |             +-------------Os09g27750
|             +274.0-|      |
|                    |      +---------------------------Pt62782556
|                    |
|                    |                           +------Os05g05680
|                    +---------------------205.0-|
|                                                +------AT1G77330
|
+-------------------------------------------------------pg10 130
```

ACC oxidase - PARS

```
+------------------------------------------------Os05g05680
|
|             +----------------------------------Os02g53180
|             |
|             |                           +------Os09g27750
|      +487.1-|      +--------------342.7-|
|      |      |      |                    +------Os09g27820
|      |      |      |
|      |      +262.3-|                    +------AT1G62380
|      |             |             +500.0-|
|      |             |      +459.0-|      +------AT1G12010
|      |             |      |      |
|      |             +339.2-|      +-------------AT1G05010
|      |                    |
|      |                    +--------------------AT2G19590
+------|
       |                                  +------Pt74282550
       |                                  |
       |                           +500.0-|------Pt10288254
       |                           |      |
       |                    +500.0-|      +------Pp3088 27
       |                    |      |
       |             +499.7-|      +-------------pg 10 128
       |             |      |
       |             |      +--------------------pg 10 130
       +-------300.8-|
                     +---------------------------Pt62782556
                     |
                     +---------------------------AT1G77330
```

Acid phosphatase - NJ

```
                                                        +------Os03g21480
                     +----------------------------500.0-|
                     |                                  +------Os07g48320
                     |
                     |                                  +------Os05g10210
                     |             +--------------500.0-|
                     |             |                    +------Os05g09704
                     |             |
                     |             |                    +------AT4G29270
              +403.0-|             |      +-------500.0-|
              |      |      +352.0-|      |             +------AT4G29260
              |      |      |      |      |
              |      |      |      |      |             +------AT5G51260
              |      |      |      |      |      +500.0-|
              |      |      |      |      +411.0-|      +------AT4G25150
              |      |      |      +293.0-|      |
              |      |      |             |      +-------------Os06g04790
              |      +302.0-|             |
       +500.0-|             |             |             +------pg4 81 18
       |      |             |             +-------500.0-|
       |      |             |             |             +------Pt74144 17
       |      |             |             |
       |      |             |             +--------------------Os06g36400
       |      |             |
+------|      |             +----------------------------------Os01g09540
|      |      |
|      |      |                                         +------AT5G44020
|      |      +-----------------------------------500.0-|
|      |                                                +------AT1G04040
|      |
|      +-------------------------------------------------------Pt74078 38
|
+--------------------------------------------------------------pg4 139 45
```

Acid phosphatase - PARS

```
                                                               +------AT5G51260
                                                        +500.0-|
              +-----------------------------------417.2-|      +------AT4G25150
              |                                         |
              |                                         +-------------Os06g04790
              |
              |                           +---------------------------Os01g09540
              |                           |
              |                           |                    +------pg4 139 45
              |                           |             +500.0-|
              |                    +280.0-|             |      +------Pt74078 38
              |                    |      |      +497.0-|
              |                    |      |      |      |      +------AT5G44020
       +499.0-|                    |      |      |      +497.2-|
       |      |                    |      +362.7-|             +------AT1G04040
       |      |             +398.8-|             |
       |      |             |      |             |             +------Os07g48320
       |      |             |      |             +-------499.7-|
       |      |             |      |                           +------Os03g21480
       |      |             |      |
       |      |      +321.8-|      |                           +------Os05g09704
+------|      |      |      |      +---------------------500.0-|
|      |      |      |      |                                  +------Os05g10210
|      |      |      |      |
|      |      +396.7-|      |                                  +------Pt74144 17
|      |             |      +----------------------------500.0-|
|      |             |                                         +------pg4 81 18
|      |             |
|      |             +------------------------------------------------Os06g36400
|      |
|      +--------------------------------------------------------------AT4G29260
|
+---------------------------------------------------------------------AT4G29270
```

AcylcoA oxidase - NJ

```
                                          +------AT4G16760
                                   +490.0-|
                            +468.0-|      +------AT2G35690
                            |      |
                     +494.0-|      +-------------Os06g01390
                     |      |
                     |      |             +------Pt37482554
              +500.0-|      +-------496.0-|
              |      |                    +------pg 2 76
              |      |
              |      |                    +------Pt20643 14
              |      +--------------491.0-|
       +482.0-|                           +------Pp7050 18
       |      |
       |      |                           +------AT1G06310
       |      |                    +343.0-|
       |      |             +494.0-|      +------AT1G06290
+------|      |             |      |
|      |      +-------500.0-|      +-------------Os06g24704
|      |                    |
|      |                    +--------------------Pt3473 25
|      |
|      |                                  +------Os11g39220
|      +----------------------------286.0-|
|                                         +------AT5G65110
|
+------------------------------------------------pg11 29
```

AcylcoA oxidase - PARS

```
                                                 +------AT1G06290
                                          +500.0-|
                                   +500.0-|      +------AT1G06310
                                   |      |
                     +-------500.0-|      +-------------Os06g24704
                     |             |
                     |             +--------------------Pt3473 25
                     |
                     |                           +------AT4G16760
              +500.0-|                    +500.0-|
              |      |             +371.0-|      +------AT2G35690
              |      |             |      |
              |      |      +499.0-|      +-------------Os06g01390
              |      |      |      |
              |      |      |      |                 +------Pt37482554
       +391.0-|      +500.0-|      +-------500.0-|
       |      |             |                    +------pg 2 76
       |      |             |
       |      |             |                    +------Pt20643 14
+------|      |             +--------------500.0-|
|      |      |                                  +------Pp7050 18
|      |      |
|      |      +-----------------------------------------pg11 29
|      |
|      +------------------------------------------------AT5G65110
|
+-------------------------------------------------------Os11g39220
```

Actin related protein - NJ

```
                                                                      +------AT5G09810
                                                               +219.0-|
                                                               |      +------Os05g36290
                                                        +262.0-|
                                                        |      |      +------Pt17002 16
                                                 +261.0-|      +408.0-|
                                                 |      |             +------Pt66098 13
                                          +390.0-|      |
                                          |      |      +--------------------Os03g61970
                                   +425.0-|      |
                                   |      |      +---------------------------AT3G53750
                            +390.0-|      |
                            |      |      +----------------------------------Pt95582554
                     +388.0-|      |
                     |      |      +-----------------------------------------AT2G42090
              +500.0-|      |
              |      |      +------------------------------------------------Pt77282549
              |      |
              |      +-------------------------------------------------------AT2G42100
       +500.0-|
       |      |                                                       +------Os08g28190
       |      |                                                +500.0-|
       |      |                                         +497.0-|      +------AT3G27000
       |      |                                         |      |
+------|      +-----------------------------------500.0-|      +-------------pg3 13
|      |                                                |
|      |                                                +--------------------Pt2223 20
|      |
|      |                                                       +-------------pg8 145
|      +-------------------------------------------------431.0-|
|                                                              |      +------Os02g38340
|                                                              +462.0-|
|                                                                     +------AT1G13180
|
+----------------------------------------------------------------------------Pt61148 18
```

Actin related protein - PARS

```
                                   +---------------------------Pt77282549
                                   |
                                   |                    +------AT2G42100
                                   |      +-------333.2-|
                            +500.0-|      |             +------AT2G42090
                            |      |      |
                            |      |      |             +------AT5G09810
                            |      |      |             |
                            |      +358.1-|             +------Pt17002 16
                            |             |             |
                            |             |      +334.4-|------Pt66098 13
                            |             |      |      |
                            |             |      |      +------Os05g36290
                            |             |      |      |
                     +500.0-|             +374.8-|      +------Os03g61970
                     |      |                    |
                     |      |                    +-------------Pt95582554
                     |      |                    |
                     |      |                    +-------------AT3G53750
                     |      |
                     |      |                           +------Os08g28190
              +267.5-|      |                    +499.8-|
              |      |      |             +479.9-|      +------AT3G27000
              |      |      |             |      |
              |      |      +-------500.0-|      +-------------pg3 13
       +493.9-|      |                    |
       |      |      |                    +--------------------Pt2223 20
       |      |      |
+------|      |      +-----------------------------------------Pt61148 18
|      |      |
|      |      +------------------------------------------------pg8 145
|      |
|      +-------------------------------------------------------AT1G13180
|
+--------------------------------------------------------------Os02g38340
```

Auxin responsive protein - NJ

```
                                                 +------pg8 105put
                                          +496.0-|
                                          |      +------Pt53830 29
                                   +389.0-|
                                   |      |      +------AT3G07570
                                   |      +500.0-|
                            +500.0-|             +------Os01g67030
                            |      |
                            |      |             +------AT3G61750
                     +347.0-|      +-------431.0-|
                     |      |                    +------Os01g48850
                     |      |
              +381.0-|      +---------------------------Pt9863 30
              |      |
              |      |                    +-------------Os03g09900
              |      +--------------348.0-|
       +500.0-|                           |      +------pg 12 82
       |      |                           +500.0-|
       |      |                                  +------Pt59582551
       |      |
+------|      |                                  +------AT3G59070
|      |      +----------------------------459.0-|
|      |                                         +------AT5G35735
|      |
|      +------------------------------------------------AT4G12980
|
+-------------------------------------------------------AT3G25290
```

Auxin responsive protein - PARS

```
                     +--------------------Os01g48850
                     |
                     |             +------Os01g67030
                     |      +498.7-|
                     |      |      +------AT3G07570
              +500.0-|-251.4-|
              |      |      |      +------pg8 105put
              |      |      +499.0-|
              |      |             +------Pt53830 29
              |      |
              |      +--------------------AT3G61750
              |
              |                    +------pg 12 82
       +500.0-|--------------500.0-|
       |      |                    +------Pt59582551
       |      |
       |      |                    +------AT3G59070
       |      +--------------383.9-|
+------|      |                    +------AT5G35735
|      |      |
|      |      +---------------------------Os03g09900
|      |      |
|      |      +---------------------------Pt9863 30
|      |
|      +----------------------------------AT4G12980
|
+-----------------------------------------AT3G25290
```

Aldo/ketoreductase - NJ

```
                                                        +------Os04g26870
                                                 +406.0-|
              +----------------------------303.0-|      +------Os04g27060
              |                                  |
              |                                  +-------------Os04g26910
              |
              |                                         +------AT2G37770
              |                                  +321.0-|
              |                           +251.0-|      +------AT3G53880
              |                           |      |
              |                    +500.0-|      +-------------Os01g62870
              |                    |      |
              |                    |      +--------------------AT2G37790
              |                    |
              |                    |                    +------Pt66382549
              |                    +--------------494.0-|
              |                    |                    +------pg8 164 29
              |                    |
              |             +500.0-|             +-------------AT1G59960
              |             |      |      +442.0-|
              |             |      |      |      |      +------Os03g13390
              |             |      +400.0-|      +394.0-|
              |             |      |      |             +------Os10g02490
              |             |      |      |
              |             |      |      +--------------------Os04g08550
              |             |      |
              |             |      +---------------------------AT5G62420
       +497.0-|      +500.0-|
       |      |      |      |                           +------Pt29916 26
       |      |      |      |                    +362.0-|
       |      |      |      |                    |      +------pg3 3 90 3
       |      |      |      |             +306.0-|
       |      |      |      |             |      +-------------AT1G06690
       |      |      |      |      +500.0-|      |
       |      |      |      |      |      |      +-------------Os07g05000
       |      |      |      |      |      |
       |      |      |      +500.0-|      +--------------------Os07g04990
       |      +500.0-|             |
       |      |      |             |                    +------AT5G53580
       |      |      |             +--------------500.0-|
       |      |      |                                  +------Os10g37330
       |      |      |
       |      |      |                                  +------pg12 55 21
       |      |      +----------------------------500.0-|
       |      |      |                                  +------Pt34367 26
+------|      |      |
|      |      |      +-----------------------------------------Os10g28320
|      |      |      |
|      |      |      +-----------------------------------------Os01g43090
|      |      |
|      |      |                                         +------pg7 160 51
|      |      |                                  +495.0-|
|      |      |                                  |      +------Pt11368255
|      |      +----------------------------500.0-|
|      |                                         |      +------pg 2 77 30
|      |                                         +470.0-|
|      |                                                +------Pt83882549
|      |
|      |                                                +------AT1G60730
|      |                                         +337.0-|
|      +-----------------------------------267.0-|      +------AT1G60690
|      |                                         |
|      |                                         +-------------AT1G60710
|      |
|      +-------------------------------------------------------AT1G60680
|
+--------------------------------------------------------------AT1G10810
```

Aldo/ketoreductase - PARS

```
                                                        +------Pt11368255
                                                 +499.3-|
                                          +453.0-|      +------pg7 160 51
                                          |      |
       +----------------------------499.2-|      +-------------Pt83882549
       |                                  |
       |                                  +--------------------pg 2 77 30
       |
       |                                                +------AT1G60730
       |                                         +256.4-|
       |                                         |      +------AT1G60690
       |                                  +276.6-|
       |                                  |      +-------------AT1G60680
       +----------------------------473.7-|      |
       |                                  |      +-------------AT1G60710
       |                                  |
       |                                  +--------------------AT1G10810
       |
       |                           +---------------------------AT5G62420
       |                           |
       |                           |                    +------Os10g02490
       |                           |             +486.3-|
       |                           |             |      +------Os03g13390
       |                           +-------276.4-|
       |                           |             |      +------AT1G59960
       |                    +299.2-|             +320.6-|
       |                    |      |                    +------Os04g08550
       |                    |      |
       |                    |      |                    +------AT2G37770
       |                    |      |             +415.1-|
       |                    |      |      +290.2-|      +------AT2G37790
       |             +500.0-|      |      |      |
       |             |      |      +500.0-|      +-------------Os01g62870
       |             |      |             |
+------|             |      |             +--------------------AT3G53880
|      |             |      |
|      |             |      |                           +------Pt66382549
|      |             |      +---------------------491.0-|
|      |      +500.0-|                                  +------pg8 164 29
|      |      |      |
|      |      |      |                                  +------Os10g37330
|      |      |      |      +---------------------500.0-|
|      |      |      |      |                           +------AT5G53580
|      |      |      |      |
|      |      |      |      |                    +-------------Os07g05000
|      |      |      +433.5-|             +326.5-|
|      |      |             |             |      |      +------Pt29916 26
|      |      |             |      +481.4-|      +493.7-|
|      |      |             |      |      |             +------pg3 3 90 3
|      +471.6-|             +500.0-|      |
|      |      |                    |      +--------------------AT1G06690
|      |      |                    |
|      |      |                    +---------------------------Os07g04990
|      |      |
|      |      |                                         +------pg12 55 21
|      |      +-----------------------------------500.0-|
|      |      |                                         +------Pt34367 26
|      |      |
|      |      +------------------------------------------------Os10g28320
|      |      |
|      |      +------------------------------------------------Os01g43090
|      |
|      +-------------------------------------------------------Os04g26870
|      |
|      +-------------------------------------------------------Os04g27060
|
+--------------------------------------------------------------Os04g26910
```

Ankyrin repeat protein - NJ

```
                                                 +---------------------------Os07g42640
                                                 |
                                                 |                    +------pg 5 79
                                          +500.0-|             +274.0-|
                                          |      |      +500.0-|      +------Pt19482550
                                          |      |      |      |
                                          |      +391.0-|      +-------------Pp5063 13
                                   +317.0-|             |
                                   |      |             +--------------------AT2G26210
                                   |      |
                                   |      |                           +------AT5G07840
                                   |      +---------------------500.0-|
                                   |                                  +------AT5G61230
                                   |
                                   |                                  +------Os03g63480
                                   |                           +463.0-|
                                   |                           |      +------Os09g33810
                                   |                    +328.0-|
                                   |                    |      |      +------AT2G17390
                                   |                    |      +500.0-|
                                   |             +383.0-|             +------AT4G35450
                            +351.0-|             |      |
                            |      |             |      |             +------pg 6 6
                            |      |             |      |             |
                            |      |      +401.0-|      +-------497.0-|------Pp7113516
                            |      |      |      |                    |
                            |      |      |      |                    +------Pt75382554
                            |      +332.0-|      |
                            |      |      |      +---------------------------Pt74882553
                     +264.0-|      |      |
                     |      |      |      +----------------------------------AT5G66055
                     |      |      |
                     |      |      +-----------------------------------------AT3G04710
                     |      |      |
              +383.0-|      |      |                                  +------AT4G18950
              |      |      |      +----------------------------422.0-|
              |      |      |                                         +------AT2G31800
              |      |      |
       +500.0-|      |      +------------------------------------------------AT4G19150
       |      |      |
       |      |      +-------------------------------------------------------Os03g55110
+------|      |
|      |      +--------------------------------------------------------------AT2G03430
|      |
|      +---------------------------------------------------------------------Pt34789 22
|
+----------------------------------------------------------------------------pg 10 106
```

Ankyrin repeat protein - PARS

```
                                          +-------------AT2G26210
                                          |
                                   +311.7-|      +------Pt19482550
                                   |      |      |
                                   |      +500.0-|------Pp5063 13
                     +-------489.9-|             |
                     |             |             +------pg 5 79
                     |             |
                     |             +--------------------Os07g42640
                     |
                     |             +--------------------Pt74882553
                     |             |
                     |             |             +------Pp7113516
                     |             |             |
                     |      +463.5-|      +500.0-|------pg 6 6
                     |      |      |      |      |
                     |      |      |      |      +------Pt75382554
                     |      |      |      |
                     |      |      +267.6-|      +------Os03g63480
                     |      |             +481.1-|
                     +250.8-|             |      +------Os09g33810
              +384.4-|      |             |
              |      |      |             |      +------AT2G17390
              |      |      |             +497.7-|
              |      |      |                    +------AT4G35450
              |      |      |
              |      |      +---------------------------AT5G66055
              |      |
              |      +----------------------------------AT3G04710
              |      |
              |      +----------------------------------AT4G19150
       +288.0-|      |
       |      |      +----------------------------------AT4G18950
       |      |      |
       |      |      |                           +------AT5G07840
       |      |      +---------------------499.6-|
       |      |      |                           +------AT5G61230
+------|      |      |
|      |      |      +----------------------------------AT2G31800
|      |      |
|      |      |                                  +------pg 10 106
|      |      +----------------------------500.0-|
|      |                                         +------Pt34789 22
|      |
|      +------------------------------------------------AT2G03430
|
+-------------------------------------------------------Os03g55110
```

AP2-EREBP //DREB - NJ

```
                                          +------AT4G39780
                                   +296.0-|
                            +345.0-|      +------Os02g51670
                            |      |
                            |      +-------------Os06g11860
                            |
                            |             +------Os03g09170
                            |             |
                            |             +------pg5 25 12
                            |             |
                            |             +------Pt3573 53
                            |             |
                            |             +------Os08g31580
                     +499.0-|             |
                     |      +-------494.0-|------AT1G22190
                     |      |             |
                     |      |             +------Pt38986 48
                     |      |             |
                     |      |             +------pg11 80 46
                     |      |             |
                     |      |             +------Os09g20350
              +483.0-|      |             |
              |      |      |             +------AT1G78080
              |      |      |
              |      |      |             +------AT1G64380
              |      |      +-------298.0-|
              |      |                    +------Os05g49700
       +268.0-|      |
       |      |      |                    +------Os04g46410
       |      |      |             +296.0-|
       |      |      +-------499.0-|      +------AT4G16750
       |      |                    |
       |      |                    +-------------Os04g46400
       |      |
+------|      +----------------------------------AT5G21960
|      |
|      |                    +--------------------Os08g35240
|      |                    |
|      |             +499.0-|             +------Pt27569 12
|      |             |      |      +493.0-|
|      |             |      +500.0-|      +------Pp414 6
|      |             |             |
|      +-------260.0-|             +-------------pg5 111 13
|                    |
|                    +---------------------------AT1G74930
|                    |
|                    |                    +------AT1G19210
|                    +--------------261.0-|
|                                         +------Os02g54050
|
+------------------------------------------------Os06g09717
```

AP2-EREBP //DREB - PARS

```
                                                 +--------------------Os06g11860
                                                 |
                                                 |      +-------------AT1G78080
                                                 |      |
                                                 |      |      +------Os05g49700
                                                 |      +345.0-|
                                                 |      |      +------AT1G64380
                                          +480.3-|      |
                                          |      |      +-------------Os03g09170
                                          |      |      |
                                          |      |      +-------------AT1G22190
                                          |      |      |
                                          |      |      +-------------Pt38986 48
                                          |      |      |
                                          |      +299.8-|-------------pg5 25 12
                                          |             |
                                          |             +-------------Os08g31580
                                          |             |
                                   +494.0-|             +-------------Os02g51670
                                   |      |             |
                                   |      |             +-------------AT4G39780
                                   |      |             |
                                   |      |             +-------------pg11 80 46
                                   |      |             |
                                   |      |             +-------------Pt3573 53
                                   |      |             |
                            +284.4-|      |             +-------------Os09g20350
                            |      |      |
                            |      |      |                    +------Os04g46400
                            |      |      |             +426.3-|
                            |      |      +-------500.0-|      +------Os04g46410
                            |      |                    |
                     +348.9-|      |                    +-------------AT4G16750
                     |      |      |
                     |      |      +----------------------------------Os06g09717
                     |      |
                     |      |                           +-------------Os08g35240
                     |      |                           |
              +378.0-|      +---------------------498.3-|      +------Pp414 6
              |      |                                  |      |
              |      |                                  +500.0-|------Pt27569 12
              |      |                                         |
       +262.9-|      |                                         +------pg5 111 13
       |      |      |
       |      |      +------------------------------------------------Os02g54050
+------|      |
|      |      +-------------------------------------------------------AT1G74930
|      |
|      +--------------------------------------------------------------AT5G21960
|
+---------------------------------------------------------------------AT1G19210
```

AP2 subfamilyb1 - NJ

```
                                    +------pg 9 89 30
                             +369.0-|
                             |      +------Pt20142 16
                      +298.0-|
                      |      |      +------Pt66101 17
                      |      +299.0-|
                      |             +------Pt58582550
                      |
                      |             +------pg 9 90 10
                      +-------469.0-|
               +486.0-|             +------Pt6317 7
               |      |
               |      |             +------pg 9 88 34
               |      +-------277.0-|
               |      |             +------Pp12793 12
               |      |
               |      |             +------Pp7549 9
               |      +-------499.0-|
               |                    +------Pt2882555
        +500.0-|
        |      |                    +------Os01g12440
        |      |      +-------262.0-|
        |      |      |             +------Os02g32140
        |      |      |
        |      |      +--------------------Pt18561 16
        |      |      |
        |      |      |             +------AT3G15210
        |      |      |      +500.0-|
        |      |      +410.0-|      +------AT1G53170
 +------|      +464.0-|      |
 |      |             |      +-------------AT5G44210
 |      |             |
 |      |             |             +------Os05g41780
 |      |             +-------438.0-|
 |      |             |             +------Os01g58420
 |      |             |
 |      |             +--------------------Os01g46870
 |      |             |
 |      |             +--------------------Os04g52090
 |      |
 |      +----------------------------------AT1G50640
 |
 +-----------------------------------------AT3G20310
```

AP2 subfamilyb1 - PARS

```
              +----------------------------------Pt66101 17
              |
              |                           +------AT3G15210
              |                    +500.0-|
              |                    |      +------AT1G53170
              |             +353.5-|
              |             |      |      +------AT5G44210
              |             |      +378.8-|
              |             |             +------Pt18561 16
              |             |
              |             +--------------------Os01g12440
              |      +252.2-|
              |      |      +--------------------Os02g32140
              |      |      |
              |      |      +--------------------Os04g52090
              +396.2-|      |
              |      |      +--------------------Os01g58420
       +317.6-|      |      |
       |      |      |      +--------------------Os05g41780
       |      |      |
       |      |      +---------------------------Os01g46870
       |      |
       |      +----------------------------------pg 9 89 30
       |      |
       |      +----------------------------------Pt20142 16
       |      |
       |      |                           +------pg 9 88 34
       |      +---------------------297.0-|
+------|      |                           +------Pp12793 12
|      |      |
|      |      |                           +------Pp7549 9
|      |      +---------------------493.4-|
|      |      |                           +------Pt2882555
|      |      |
|      |      +----------------------------------Pt58582550
|      |
|      |                                  +------AT1G50640
|      +----------------------------500.0-|
|      |                                  +------AT3G20310
|      |
|      +-----------------------------------------Pt6317 7
|
+------------------------------------------------pg 9 90 10
```

AP2-EREBP //ERF genes on chromosome 5 - NJ

```
                          +-------------Os02g54160
                    +394.0-|
                    |      |      +------Os06g09390
                    |      +274.0-|
              +284.0-|             +------Os09g26420
              |      |
              |      +--------------------AT3G14230
              |      |
              |      +--------------------AT1G53910
              |
        +340.0-|                   +------pg5 132 28
        |     +--------------386.0-|
        |     |                    +------pg5 133 82
        |     |
  +------|     +--------------------------Pt52682550
  |      |     |
  |      |     +--------------------------Pp5971 22
  |      |
  |      +---------------------------------Pt29791 33
  |
  +----------------------------------------pg5 127 66
```

AP2-EREBP //ERF genes on chromosome 5 -PARS

```
                                                 +------Pt29791 33
                                          +490.6-|
                                   +494.6-|      +------pg5 132 28
                                   |      |
                            +483.8-|      +-------------pg5 127 66
                            |      |
                            |      +--------------------pg5 133 82
                     +494.2-|
                     |      +---------------------------Pp5971 22
                     |      |
                     |      +---------------------------Pt52682550
              +339.7-|
              |      +----------------------------------AT3G14230
              |      |
       +------|      +----------------------------------AT1G53910
       |      |      |
       |      |      +----------------------------------Os09g26420
       |      |
       |      +-----------------------------------------Os02g54160
       |
       +------------------------------------------------Os06g09390
```

AP2-EREBP //ERF - NJ

```
                     +------------------------------------------------AT2G44840
                     |
                     |             +----------------------------------Os05g45954
                     |             |
                     |             |                           +------Os02g29550
                     |             |                    +271.0-|
                     |             |             +278.0-|      +------pg2 126 20
                     |      +409.0-|             |      |
                     |      |      |             |      +-------------Pt54827 21
                     |      |      |             |
                     |      |      |      +413.0-|--------------------Os11g03540
                     |      |      |      |      |
                     +265.0-|      |      |      +--------------------AT4G13040
                     |      |      +329.0-|      |
                     |      |             |      +--------------------AT3G54320
                     |      |             |
                     |      |             +---------------------------Os08g34360
                     |      |
                     |      +-----------------------------------------Os06g05340
                     |
              +354.0-|------------------------------------------------Os02g43790
              |      |
              |      +------------------------------------------------AT5G51190
              |      |
              |      |                                         +------Pt76314 23
              |      |                                  +354.0-|
              |      +----------------------------275.0-|      +------pg9 10 18
              |      |                                  |
              |      |                                  +-------------Pt12736 19
              |      |
              |      +------------------------------------------------AT5G47220
       +329.0-|      |
       |      |      +------------------------------------------------AT4G17500
       |      |      |
       |      |      +------------------------------------------------Os04g46220
       |      |      |
       |      |      +------------------------------------------------Os01g54890
       |      |      |
       |      |      +------------------------------------------------AT5G61600
       |      |      |
       |      |      +------------------------------------------------Os04g46250
       |      |
       |      +-------------------------------------------------------Os03g08470
       |
+------|                                                       +------pg5 133 82
|      |                                                       |
|      |                                                       +------Pp5971 22
|      |                                                +420.0-|
|      |                                                |      +------Pt52682550
|      +------------------------------------------309.0-|      |
|      |                                                |      +------Pt29791 33
|      |                                                |
|      |                                                +-------------AT1G53910
|      |
|      |                                                       +------Os06g09390
|      +-------------------------------------------------315.0-|
|      |                                                       +------Os02g54160
|      |
|      +--------------------------------------------------------------Os09g26420
|
+---------------------------------------------------------------------AT3G14230
```

AP2-EREBP //ERF - PARS

```
                                +------Pt76314 23
                          +428.0-|
          +--------------479.1-|      +------pg9 10 18
          |                    |
          |                    +-------------Pt12736 19
          |
          +---------------------------------AT2G44840
          |
          |             +--------------------AT4G13040
          |             |
          |      +288.2-|             +------pg2 126 20
          |      |      |      +496.6-|
          |      |      +453.6-|      +------Pt54827 21
          |      |             |
          |      |             +-------------Os02g29550
          |      |
          |      |                    +------Os08g34360
          +415.1-|             +302.7-|
          |      +-------463.4-|      +------AT3G54320
          |      |             |
          |      |             +-------------Os11g03540
   +487.1-|      |
   |      |      +---------------------------Os06g05340
   |      |      |
   |      |      +---------------------------Os05g45954
   |      |
   |      |                           +------AT4G17500
   |      +--------------------395.2-|
   |      |                           +------AT5G47220
   |      |
   |      |                           +------AT5G51190
   |      |                    +454.5-|
   |      +--------------349.2-|      +------AT5G61600
   |      |                    |
   |      |                    +-------------Os04g46250
+------|      |
|      |      +---------------------------------Os01g54890
|      |      |
|      |      +---------------------------------Os04g46220
|      |      |
|      |      +---------------------------------Os02g43790
|      |
|      |             +---------------------------AT3G14230
|      |             |
|      |             +---------------------------Os09g26420
|      |             |
|      |             |                    +------Os02g54160
|      |             +--------------253.2-|
|      |             |                    +------Os06g09390
|      |             |
|      +-------274.4-|             +-------------Pp5971 22
|                    |      +395.7-|
|                    |      |      |      +------pg5 133 82
|                    +416.2-|      +397.0-|
|                    |      |             +------Pt29791 33
|                    |      |
|                    |      +--------------------Pt52682550
|                    |
|                    +---------------------------AT1G53910
|
+------------------------------------------------Os03g08470
```

Argonaute - NJ

```
                            +-------------------------------------------------------Os03g47830
                            |
                            |                                                +------AT1G69440
                            |             +----------------------------498.0-|
                            |             |                                  +------Pt15631
                            |             |
                            |             |                                  +------Os01g16850
                            |             |                                  |
                            |             |                    +-------354.0-|------Os04g06770
                            |             |                    |             |
                            |             |                    |             +------Os01g16870
                            |             |             +475.0-|
                            |             |             |      |             +------AT2G27040
                            |             |             |      |      +296.0-|
                            |             |             |      +339.0-|      +------AT5G21030
                            |             |      +324.0-|             |
                            |             |      |      |             +-------------AT5G21150
                            |             |      |      |
                            |      +436.0-|      |      +---------------------------AT2G32940
                     +292.0-|      |      |      |      |
                     |      |      |      +476.0-|      +---------------------------Os07g16224
                     |      |      |      |      |
                     |      |      |      |      |                    +-------------pg3 25
                     |      |      |      |      |             +466.0-|
                     |      |      |      |      |             |      |      +------pg3 24
                     |      |      |      |      +-------496.0-|      +444.0-|
                     |      |      |      |                    |             +------Pt49182547
                     |      +257.0-|      |                    |
                     |      |      |      |                    +--------------------Pt60282548
              +271.0-|      |      |      |
              |      |      |      |      |                                  +------AT1G31290
              |      |      |      |      +----------------------------500.0-|
              |      |      |      |      |                                  +------AT1G31280
              |      |      |      |      |
              |      |      |      |      +-----------------------------------------Pt24844
              |      |      |      |
       +382.0-|      |      |      +------------------------------------------------Os07g09020
       |      |      |      |
       |      |      |      +-------------------------------------------------------AT2G27880
       |      |      |
       |      |      +--------------------------------------------------------------Os03g58600
       |      |
       |      |                                                              +------pg2 151
       |      +--------------------------------------------------------500.0-|
       |                                                                     +------Pt19192
       |
       |                                                              +-------------Os02g58490
       |                                                              |
       +--------------------------------------------------------303.0-|-------------Os06g51310
       |                                                              |
+------|                                                              |      +------Pt9825
|      |                                                              +468.0-|
|      |                                                                     +------Pt81778
|      |
|      |                                                                     +------Os04g47870
|      |                                                              +257.0-|
|      +--------------------------------------------------------315.0-|      +------pg9 7
|      |                                                              |
|      |                                                              +-------------Os02g45070
|      |
|      +----------------------------------------------------------------------------AT5G43810
|      |
|      +----------------------------------------------------------------------------Os06g39640
|      |
|      +----------------------------------------------------------------------------Pt18682551
|
+-----------------------------------------------------------------------------------AT1G48410
```

Argonaute - PARS

```
                                                                             +------pg3 24
                                                                      +316.2-|
                                                               +252.5-|      +------Pt49182547
                                                               |      |
                                                        +487.5-|      +-------------pg3 25
                                                        |      |
                                                        |      +--------------------Pt60282548
                                                        |
                                                        |                    +------Os01g16870
                                                        |                    |
                                                 +314.1-|--------------263.5-|------Os01g16850
                                                 |      |                    |
                                                 |      |                    +------Os04g06770
                                                 |      |
                                                 |      +---------------------------AT5G21150
                                          +289.2-|      |
                                          |      |      |                    +------AT5G21030
                                          |      |      +--------------259.6-|
                                   +478.0-|      |                           +------AT2G27040
                                   |      |      |
                                   |      |      +----------------------------------AT2G32940
                                   |      |
                                   |      +-----------------------------------------Os07g16224
                                   |
                            +421.4-|                                         +------AT1G69440
                            |      +-----------------------------------442.6-|
                            |      |                                         +------Pt15631
                            |      |
                            |      +------------------------------------------------Pt24844
                     +365.4-|      |
                     |      |      |                                         +------AT1G31280
                     |      |      +-----------------------------------500.0-|
                     |      |                                                +------AT1G31290
                     |      |
              +391.5-|      +-------------------------------------------------------Os07g09020
              |      |
              |      +--------------------------------------------------------------AT2G27880
              |      |
              |      +--------------------------------------------------------------Os03g47830
              |      |
              |      +--------------------------------------------------------------Os03g58600
              |
              |                                                              +------Pt81778
              +--------------------------------------------------------387.0-|
              |                                                              +------Pt9825
              |
              +---------------------------------------------------------------------Os04g47870
              |
       +500.0-|---------------------------------------------------------------------Pt18682551
       |      |
       |      +---------------------------------------------------------------------Os02g58490
       |      |
       |      +---------------------------------------------------------------------AT1G48410
       |      |
       |      +---------------------------------------------------------------------AT5G43810
       |      |
+------|      +---------------------------------------------------------------------pg9 7
|      |      |
|      |      +---------------------------------------------------------------------Os02g45070
|      |      |
|      |      +---------------------------------------------------------------------Os06g39640
|      |      |
|      |      +---------------------------------------------------------------------Os06g51310
|      |
|      +----------------------------------------------------------------------------Pt19192
|
+-----------------------------------------------------------------------------------pg2 151
```

Armadillo - NJ

```
                             +------AT1G23030
                      +499.0-|
      +-------262.0-|       +------AT1G71020
      |             |
      |             +-------------AT2G28830
      |
      |                      +------AT3G46510
      |             +279.0-|
      |             |       +------AT3G54850
      |      +414.0-|
      |      |      |       +------pg4 104
      +274.0-|      +496.0-|
      |      |              +------Pt80980 26
      |      |
      |      +-------------------Os06g01304
      |
+------|                     +------pg8 178
|      |             +500.0-|
|      +-------398.0-|      +------Pt47225 22
|      |             |
|      |             +-------------AT5G42340
|      |
|      +---------------------------Pt27806 29
|      |
|      |                     +------AT3G03440
|      +--------------287.0-|
|      |                     +------pg10 29
|      |
|      +---------------------------Os12g38210
|      |
|      +---------------------------Os03g16824
|
+----------------------------------Pt5796 32
```

Armadillo - PARS

```
                            +------pg4 104
              +-------460.6-|
              |             +------Pt80980 26
              |
              +--------------------Pt5796 32
              |
              |             +------Os12g38210
              +-------310.7-|
              |             +------Os03g16824
              |
              |             +------AT1G23030
              +-------500.0-|
              |             +------AT1G71020
              |
       +456.8-|--------------------AT3G54850
       |      |
       |      +--------------------Pt27806 29
       |      |
       |      +--------------------Os06g01304
       |      |
       |      |      +-------------AT5G42340
       |      +400.3-|
+------|      |      |      +------Pt47225 22
|      |      |      +500.0-|
|      |      |             +------pg8 178
|      |      |
|      |      +--------------------AT3G46510
|      |      |
|      |      +--------------------AT2G28830
|      |
|      +---------------------------AT3G03440
|
+----------------------------------pg10 29
```

Aspartyl protease - NJ

```
                                          +------Pt13482548
                                          |
                     +--------------500.0-|------Pp11969 37
                     |                    |
                     |                    +------pg5 11
                     |
                     |                    +------Os05g51220
                     |      +-------493.0-|
                     |      |             +------Os01g41550
                     |      |
                     +350.0-|             +------AT1G01300
                     |      |      +500.0-|
                     |      |      |      +------AT3G61820
                     |      +274.0-|
              +255.0-|             |      +------Pt73913 37
              |      |             +375.0-|
              |      |                    +------pg8 76
              |      |
              |      +---------------------------AT3G20015
              |      |
              |      |                    +------Os01g52190
              |      +--------------436.0-|
       +500.0-|      |                    +------Pt28202 28
       |      |      |
       |      |      +---------------------------AT3G18490
       |      |      |
       |      |      |                    +------AT1G25510
       |      |      +--------------352.0-|
       |      |                           +------Os01g04710
       |      |
       |      +----------------------------------Os04g58070
       |
       +-----------------------------------------Os05g04630
       |
       |                                  +------Pt40940 33
       +----------------------------295.0-|
+------|                                  +------Pp6113518
|      |
|      |                           +-------------Pt39372 9
|      |                    +256.0-|
|      |                    |      |      +------AT4G04460
|      +--------------265.0-|      +480.0-|
|      |                    |             +------pg 4 72
|      |                    |
|      |                    +--------------------Os05g49200
|      |
|      |                                  +------Pt43682554
|      +----------------------------394.0-|
|      |                                  +------Pp2701 16
|      |
|      +-----------------------------------------AT1G62290
|
+------------------------------------------------AT1G11910
```

Aspartyl protease - PARS

```
              +-------------pg8 76
              |
              +-------------Pt73913 37
              |
              |      +------AT1G01300
              +497.8-|
              |      +------AT3G61820
              |
              |      +------Pt28202 28
              +274.3-|
              |      +------Os01g52190
              |
              +-------------AT3G20015
              |
              |      +------Os05g51220
       +500.0-|-498.0-|
       |      |      +------Os01g41550
       |      |
       |      +-------------Os01g04710
       |      |
       |      +-------------AT3G18490
       |      |
       |      +-------------AT1G25510
       |      |
       |      |      +------Pt13482548
       |      |      |
       |      +499.2-|------pg5 11
       |      |      |
       |      |      +------Pp11969 37
       |      |
       |      +-------------Os04g58070
       |
+------|--------------------AT4G04460
|      |
|      |             +------Pt39372 9
|      +-------252.5-|
|      |             +------pg 4 72
|      |
|      |      +-------------Os05g04630
|      |      |
|      |      |      +------AT1G11910
|      +262.5-|-295.5-|
|      |      |      +------AT1G62290
|      |      |
|      |      |      +------Pp2701 16
|      |      +469.5-|
|      |             +------Pt43682554
|      |
|      |             +------Pp6113518
|      +-------440.7-|
|                    +------Pt40940 33
|
+---------------------------Os05g49200
```

ATP-dependent Clp protease - NJ

```
                                                        +---------------------------Os10g43050
                                                        |
                                                        |                    +------Pp1472 13
                                                 +269.0-|             +498.0-|
                                                 |      |      +500.0-|      +------Pt4282551
                                                 |      |      |      |
                                                 |      +436.0-|      +-------------pg 6 102
                                                 |             |
                                                 |             +--------------------AT5G45390
                                          +485.0-|
                                          |      |             +--------------------Os01g32350
                                          |      |             |
                                          |      |      +480.0-|             +------Pp13489 25
                                          |      |      |      |      +256.0-|
                                          |      |      |      +500.0-|      +------Pt51070 30
                                   +276.0-|      +494.0-|             |
                                   |      |             |             +-------------pg 5 107
                                   |      |             |
                                   |      |             +---------------------------AT1G66670
                                   |      |
                            +422.0-|      |                                  +------AT1G02560
                            |      |      |                           +394.0-|
                            |      |      +---------------------500.0-|      +------Os03g19510
                            |      |                                  |
                     +500.0-|      |                                  +-------------Pt90782552
                     |      |      |
                     |      |      +------------------------------------------------AT1G11750
                     |      |
                     |      |                                                +------AT5G23140
                     |      +------------------------------------------496.0-|
              +477.0-|                                                       +------Os02g42290
              |      |
              |      |                                                       +------AT1G49970
              |      +-------------------------------------------------429.0-|
       +445.0-|      |                                                       +------Pt57172 27
       |      |      |
       |      |      +--------------------------------------------------------------Os05g51450
       |      |
       |      |                                                       +-------------Pt32786 24
       |      +-------------------------------------------------493.0-|
+------|                                                              |      +------AT4G17040
|      |                                                              +498.0-|
|      |                                                                     +------Os01g16530
|      |
|      |                                                                     +------Pt5074 17
|      |                                                              +500.0-|
|      +--------------------------------------------------------397.0-|      +------pg 2 130
|                                                                     |
|                                                                     +-------------AT1G09130
|
+-----------------------------------------------------------------------------------Os03g22430
```

ATP-dependent Clp protease - PARS

```
                                                                      +------AT4G17040
                                                               +500.0-|
                     +-----------------------------------493.4-|      +------Os01g16530
                     |                                         |
                     |                                         +-------------Pt32786 24
                     |
                     |                                                +------Pp13489 25
                     |                                                |
                     |                                         +500.0-|------pg 5 107
                     |                                         |      |
                     |                    +--------------482.0-|      +------Pt51070 30
                     |                    |                    |
                     |                    |                    |      +------Os01g32350
                     |                    |                    +331.2-|
                     |                    |                           +------AT1G66670
                     |             +432.0-|
                     |             |      |             +--------------------AT5G45390
              +458.2-|             |      |             |
              |      |             |      |      +313.9-|             +------Pp1472 13
              |      |             |      |      |      |      +498.0-|
              |      |             |      |      |      +500.0-|      +------Pt4282551
              |      |             |      +340.2-|             |
              |      |             |             |             +-------------pg 6 102
              |      |             |             |
              |      |             |             +---------------------------Os10g43050
              |      |      +500.0-|
              |      |      |      |                                  +------AT1G02560
              |      |      |      |                           +283.4-|
              |      |      |      +---------------------499.6-|      +------Os03g19510
              |      |      |      |                           |
              |      |      |      |                           +-------------Pt90782552
       +341.4-|      |      |      |
       |      |      +346.5-|      |                                  +------AT5G23140
       |      |             |      +----------------------------477.3-|
       |      |             |      |                                  +------Os02g42290
       |      |             |      |
       |      |             |      +-----------------------------------------AT1G11750
       |      |             |
       |      |             |                                         +------AT1G49970
+------|      |             |                                  +389.3-|
|      |      |             +----------------------------334.3-|      +------Pt57172 27
|      |      |                                                |
|      |      |                                                +-------------Os05g51450
|      |      |
|      |      |                                                       +------pg 2 130
|      |      +-------------------------------------------------500.0-|
|      |                                                              +------Pt5074 17
|      |
|      +---------------------------------------------------------------------AT1G09130
|
+----------------------------------------------------------------------------Os03g22430
```

Autophagy - NJ

```
                                   +-------------Os03g27350
                            +268.0-|
                            |      |      +------pg3 77
                     +484.0-|      +471.0-|
                     |      |             +------Pt26365 19
              +500.0-|      |
              |      |      +--------------------AT2G44140
       +399.0-|      |
       |      |      +---------------------------AT3G59950
       |      |
       |      +----------------------------------pg 2 38
       |
       |                                  +------AT3G06420
       |                           +349.0-|
       |                    +491.0-|      +------AT3G15580
       |                    |      |
       +--------------368.0-|      +-------------Os11g01010
+------|                    |
|      |                    |             +------pg 6 45
|      |                    +-------409.0-|
|      |                                  +------Pp2466 16
|      |
|      +-----------------------------------------AT2G05630
|      |
|      +-----------------------------------------Pt66980 5
|      |
|      |                           +-------------AT4G16520
|      +---------------------322.0-|
|                                  |      +------Pt47701 12
|                                  +433.0-|
|                                         +------Pt47700 10
|
+------------------------------------------------Os07g32800
```

Autophagy - PARS

```
                                                        +------Pt47700 10
                                                 +487.4-|
                                          +486.6-|      +------Pt47701 12
                                          |      |
                                   +454.2-|      +-------------AT4G16520
                                   |      |
                                   |      |             +------AT2G05630
                            +466.8-|      +-------459.8-|
                            |      |                    +------Os07g32800
                            |      |
                     +470.9-|      +---------------------------Pt66980 5
                     |      |
                     |      |                           +------pg 6 45
                     |      +---------------------283.2-|
              +500.0-|                                  +------Pp2466 16
              |      |
              |      |                                  +------AT3G06420
              |      |                                  |
       +500.0-|      +----------------------------337.7-|------AT3G15580
       |      |                                         |
       |      |                                         +------Os11g01010
       |      |
+------|      +------------------------------------------------pg 2 38
|      |
|      |                                                +------pg3 77
|      |                                         +500.0-|
|      |                                         |      +------Pt26365 19
|      +-----------------------------------291.8-|
|                                                |      +------AT2G44140
|                                                +306.8-|
|                                                       +------AT3G59950
|
+--------------------------------------------------------------Os03g27350
```

ARF - NJ

```
                                                                                                         +------Os06g09660
                                                                                                  +272.0-|
                                                                                           +416.0-|      +------AT5G20730
                                                                                           |      |
                                                                                    +310.0-|      +-------------AT1G19220
                                                                                    |      |
                                                               +--------------321.0-|      +--------------------Os08g40900
                                                               |                    |
                                                               |                    |                    +------Os06g48950
                                                               |                    +--------------455.0-|
                                                               |                                         +------Os02g04810
                                                               |
                                                               |                                  +-------------AT1G30330
                                                               |                           +498.0-|
                                                        +371.0-|                           |      |      +------Os06g46410
                                                        |      |                    +251.0-|      +477.0-|
                                                        |      |                    |      |             +------Os02g06910
                                                        |      |             +381.0-|      |
                                                        |      |             |      |      +--------------------pg 9 52 12
                                                        |      |             |      |
                                                        |      |      +451.0-|      +---------------------------Pt3182554
                                                        |      |      |      |
                                                 +369.0-|      |      |      |                           +------AT5G37020
                                                 |      |      +495.0-|      +---------------------410.0-|
                                                 |      |             |                                  +------Os04g57610
                                                 |      |             |
                                                 |      |             |                                  +------Pt70482549
                                                 |      |             +----------------------------336.0-|
                                          +348.0-|      |                                                +------pg 1 96 11
                                          |      |      |
                                          |      |      |                                                +------Os04g56850
                                          |      |      +------------------------------------------476.0-|
                                          |      |                                                       +------AT1G19850
                                          |      |
                                          |      +--------------------------------------------------------------AT5G60450
                                          |
                                          |                                                       +-------------Os01g70270
                                          |                                                       |
                                          |                                                       |      +------Pt40182547
                                   +416.0-|                                                       +382.0-|
                                   |      |                                                       |      +------Pt69482549
                                   |      |                                                       |
                                   |      +-------------------------------------------------263.0-|      +------Os11g32110
                                   |      |                                                       +357.0-|
                                   |      |                                                       |      +------Os12g29520
                                   |      |                                                       |
                            +333.0-|      |                                                       +-------------AT5G62000
                            |      |      |                                                       |
                            |      |      |                                                       +-------------Pt82281 17
                            |      |      |
                            |      |      +---------------------------------------------------------------------pg1 53 49
                            |      |
                     +500.0-|      |                                                              +-------------AT1G59750
                     |      |      +--------------------------------------------------------449.0-|
                     |      |                                                                     |      +------Os02g35140
                     |      |                                                                     +499.0-|
                     |      |                                                                            +------Os04g36054
                     |      |
              +261.0-|      |                                                                     +-------------AT2G46530
              |      |      +---------------------------------------------------------------257.0-|
              |      |                                                                            |      +------AT4G23980
              |      |                                                                            +257.0-|
              |      |                                                                                   +------AT3G61830
              |      |
       +432.0-|      +------------------------------------------------------------------------------------------AT1G35520
       |      |
       |      +-------------------------------------------------------------------------------------------------AT1G34410
       |      |
+------|      +-------------------------------------------------------------------------------------------------AT1G34310
|      |      |
|      |      +-------------------------------------------------------------------------------------------------AT1G35240
|      |
|      +--------------------------------------------------------------------------------------------------------AT1G34390
|
+---------------------------------------------------------------------------------------------------------------AT1G35540
```

ARF - PARS

```
                                                               +------AT1G35520
                                                               |
                                                               +------AT1G35540
                                                               |
                                                               +------AT1G35240
       +-------------------------------------------------498.9-|
       |                                                       +------AT1G34390
       |                                                       |
       |                                                       +------AT1G34310
       |                                                       |
       |                                                       +------AT1G34410
       |
       |                    +-----------------------------------------Pt70482549
       |                    |
       |                    |                                  +------AT5G37020
       |                    |                    +-------499.9-|
       |                    |                    |             +------Os04g57610
       |             +442.2-|             +494.9-|
       |             |      |             |      |             +------Os02g06910
       |             |      |             |      |      +447.7-|
       |             |      |      +251.4-|      +492.6-|      +------Os06g46410
       |             |      |      |      |             |
       |      +458.6-|      |      |      |             +-------------AT1G30330
       |      |      |      +498.2-|      |
       |      |      |             |      +---------------------------Pt3182554
       |      |      |             |
       |      |      |             +----------------------------------pg 9 52 12
       |      |      |
       |      |      +------------------------------------------------pg 1 96 11
       |      |
       |      |                                                +------Os06g48950
       |      +------------------------------------------489.9-|
       +428.9-|                                                +------Os02g04810
       |      |
       |      +-------------------------------------------------------Os08g40900
       |      |
       |      |                                                +------Os04g56850
+------|      +------------------------------------------452.6-|
|      |      |                                                +------AT1G19850
|      |      |
|      |      +-------------------------------------------------------AT1G19220
|      |      |
|      |      |                                                +------Os06g09660
|      |      +------------------------------------------276.5-|
|      |                                                       +------AT5G20730
|      |
|      +--------------------------------------------------------------Os01g70270
|      |
|      +--------------------------------------------------------------AT3G61830
|      |
|      +--------------------------------------------------------------Pt40182547
|      |
|      +--------------------------------------------------------------Os12g29520
|      |
|      |                                                       +------AT5G62000
|      +-------------------------------------------------284.6-|
|      |                                                       +------AT5G60450
|      |
|      +--------------------------------------------------------------AT2G46530
|      |
|      +--------------------------------------------------------------Pt69482549
|      |
|      +--------------------------------------------------------------Os11g32110
|      |
|      +--------------------------------------------------------------Pt82281 17
|      |
|      +--------------------------------------------------------------pg1 53 49
|      |
|      |                                                       +------Os04g36054
|      |                                                +483.3-|
|      +------------------------------------------375.6-|      +------Os02g35140
|                                                       |
|                                                       +-------------AT1G59750
|
+---------------------------------------------------------------------AT4G23980
```

Auxin Responsive Protein - NJ

```
                                                 +------pg8 105put
                                          +496.0-|
                                          |      +------Pt53830 29
                                   +389.0-|
                                   |      |      +------AT3G07570
                                   |      +500.0-|
                            +500.0-|             +------Os01g67030
                            |      |
                            |      |             +------AT3G61750
                     +347.0-|      +-------431.0-|
                     |      |                    +------Os01g48850
                     |      |
              +381.0-|      +---------------------------Pt9863 30
              |      |
              |      |                    +-------------Os03g09900
              |      +--------------348.0-|
       +500.0-|                           |      +------pg 12 82
       |      |                           +500.0-|
       |      |                                  +------Pt59582551
       |      |
+------|      |                                  +------AT3G59070
|      |      +----------------------------459.0-|
|      |                                         +------AT5G35735
|      |
|      +------------------------------------------------AT4G12980
|
+-------------------------------------------------------AT3G25290
```

Auxin Responsive Protein - PARS

```
                     +--------------------Os01g48850
                     |
                     |             +------Os01g67030
                     |      +498.7-|
                     |      |      +------AT3G07570
              +500.0-|-251.4-|
              |      |      |      +------pg8 105put
              |      |      +499.0-|
              |      |             +------Pt53830 29
              |      |
              |      +--------------------AT3G61750
              |
              |                    +------pg 12 82
       +500.0-|--------------500.0-|
       |      |                    +------Pt59582551
       |      |
       |      |                    +------AT3G59070
       |      +--------------383.9-|
+------|      |                    +------AT5G35735
|      |      |
|      |      +---------------------------Os03g09900
|      |      |
|      |      +---------------------------Pt9863 30
|      |
|      +----------------------------------AT4G12980
|
+-----------------------------------------AT3G25290
```

AUX - NJ

```
+----------------------------------------------------------------------Pt45234 23
 |
 |                                                       +-------------AT4G28640
 |                                                       |
 |                                                       |      +------pg3 89 26
 |                                  +--------------327.0-|-500.0-|
 |                                  |                    |      +------Pt37682553
 |                                  |                    |
 |                                  |                    |      +------AT2G33310
 |                                  |                    +500.0-|
 |                                  |                           +------AT1G04550
 |                                  |
 |                           +252.0-|                           +------Pt63131 21
 |                           |      |                    +500.0-|
 |                           |      |             +499.0-|      +------Pp9453 14
 |                           |      |             |      |
 |                           |      |      +396.0-|      +-------------pg 2 46 53
 |                           |      |      |      |
 |                           |      |      |      +--------------------Os02g57250
 |                           |      |      |
 |                    +325.0-|      +437.0-|                    +------Os01g53880
 |                    |      |             +--------------287.0-|
 |                    |      |             |                    +------AT3G16500
 |                    |      |             |
 |                    |      |             +---------------------------Os02g13520
 |                    |      |
 |                    |      +-----------------------------------------pg12 64 17
 |                    |      |
 |                    |      +-----------------------------------------Os05g08570
 |                    |
 |                    |                                  +-------------AT4G29080
 |                    |                           +300.0-|
 |                    |                           |      |      +------Os01g13030
 |                    +---------------------326.0-|      +389.0-|
 |                    |                           |             +------Os05g14180
 |                    |                           |
 |                    |                           +--------------------Os06g22870
 |                    |
 |                    +------------------------------------------------Os03g53150
 |             +258.0-|
 |             |      |                                         +------Os05g48590
 |             |      +-----------------------------------497.0-|
 |             |      |                                         +------Os01g48444
 |             |      |
 |             |      |                                         +------AT1G04250
 |             |      |                                  +325.0-|
 |             |      +----------------------------438.0-|      +------AT4G14550
 |             |      |                                  |
 |             |      |                                  +-------------AT3G23050
 |             |      |
 |      +469.0-|      +------------------------------------------------AT2G22670
 |      |      |      |
 |      |      |      +------------------------------------------------Os12g40890
 |      |      |      |
 |      |      |      +------------------------------------------------Os03g43400
 |      |      |      |
 |      |      |      +------------------------------------------------AT3G04730
 |      |      |      |
 |      |      |      +------------------------------------------------AT5G65670
 +------|      |
        |      |                                  +--------------------Pt18949 57
        |      |                                  |
        |      +----------------------------500.0-|             +------pg12 62 17
        |                                         |      +492.0-|
        |                                         +460.0-|      +------Pt76261 19
        |                                                |
        |                                                +-------------Pt64486 14
        |
        |                                                       +------pg12 61 18
        +-------------------------------------------------460.0-|
                                                                +------Pp1055 23
```

AUX - PARS

```
+----------------------------------------Os01g53880
|
|                    +------pg3 89 26
|            +--------------500.0-|
|            |                    +------Pt37682553
|            |
|            +---------------------------pg12 64 17
|            |
|            |                    +------AT2G22670
|            |      +-------279.8-|
|            |      |             +------AT5G65670
|            +282.9-|
|            |      |             +------AT4G14550
|            |      |      +314.4-|
|            |      +307.3-|      +------AT1G04250
|            |             |
|            |             +-------------AT3G23050
|            |
|            |                    +------Os01g48444
|            +--------------458.3-|
|            |                    +------Os05g48590
|            |
|            +---------------------------Os03g43400
|            |
|            +---------------------------Os12g40890
|            |
|            +---------------------------Os03g53150
|            |
|            +---------------------------Os06g22870
|            |
|      +266.4-|---------------------------Os05g08570
|      |      |
|      |      +---------------------------AT4G29080
|      |      |
|      |      |             +-------------Pt64486 14
|      |      |      +444.7-|
|      |      |      |      |      +------Pt76261 19
|      |      +322.3-|      +259.2-|
|      |      |      |             +------pg12 62 17
|      |      |      |
|      |      |      +--------------------Pt18949 57
|      |      |
|      |      +---------------------------AT4G28640
|      |      |
|      |      |                    +------Pt45234 23
|      |      |                    |
|      |      +--------------469.9-|------Pp1055 23
|      |      |                    |
|      |      |                    +------pg12 61 18
|      |      |
+------|      |                    +------AT2G33310
       |      +--------------491.3-|
       |      |                    +------AT1G04550
       |      |
       |      +---------------------------AT3G04730
       |      |
       |      |                    +------Os05g14180
       |      +--------------282.4-|
       |                           +------Os01g13030
       |
       |             +--------------------Os02g57250
       +-------314.1-|
       |             |      +-------------pg 2 46 53
       |             +499.0-|
       |                    |      +------Pt63131 21
       |                    +499.4-|
       |                           +------Pp9453 14
       |
       +----------------------------------AT3G16500
       |
       +----------------------------------Os02g13520
```

6b interacting protein - NJ

```
                                   +------Pt6004 29
                            +500.0-|
                            |      +------pg5 120 27
                     +258.0-|
                     |      |      +------AT3G11100
                     |      +331.0-|
                     |             +------AT5G05550
                     |
                     +--------------------pg9 9 20
                     |
                     +--------------------Os01g52090
                     |
                     |             +------AT3G10030
                     |      +449.0-|
                     +390.0-|      +------Os09g38570
                     |      |
              +465.0-|      +-------------AT3G54390
              |      |
              |      |             +------Os04g36790
              |      +-------410.0-|
              |      |             +------Os02g35690
              |      |
              |      +--------------------AT3G58630
              |      |
       +435.0-|      |             +------Os01g48320
       |      |      +-------396.0-|
       |      |      |             +------Os05g48690
       |      |      |
       |      |      +--------------------Pt42175 20
+------|      |      |
|      |      |      +--------------------AT3G14180
|      |      |
|      |      +---------------------------AT3G24490
|      |
|      +----------------------------------Pt56876 23
|
+-----------------------------------------pg8 122 21
```

6b interacting protein - PARS

```
                                                  +------AT3G54390
                                           +337.1-|
                                    +285.0-|      +------Pt42175 20
                                    |      |
                             +337.9-|      +-------------Os09g38570
                             |      |
                      +300.5-|      +--------------------AT3G10030
                      |      |
                      |      +---------------------------Os01g52090
                      |
                      +----------------------------------pg9 9 20
                      |
                      |                           +------Pt6004 29
                      +---------------------500.0-|
                      |                           +------pg5 120 27
                      |
                      |                           +------Pt56876 23
               +450.7-|                    +409.3-|
               |      +--------------329.1-|      +------pg8 122 21
               |      |                    |
               |      |                    +-------------AT3G24490
               |      |
               |      |                           +------AT3G11100
               |      +---------------------493.6-|
        +500.0-|      |                           +------AT5G05550
        |      |      |
        |      |      |                           +------Os05g48690
        |      |      |                    +477.6-|
        |      |      +--------------296.6-|      +------Os01g48320
+-------|      |                           |
|       |      |                           +-------------AT3G58630
|       |      |
|       |      +-----------------------------------------AT3G14180
|       |
|       +------------------------------------------------Os04g36790
|
+--------------------------------------------------------Os02g35690
```

Band 7 family protein - NJ

```
                                          +------AT5G51570
                                   +489.0-|
                                   |      +------Os06g04460
                     +-------488.0-|
                     |             |      +------Pt282 32
                     |             +500.0-|
                     |                    +------pg 4 53
              +500.0-|
              |      |                    +------AT1G69840
              |      |             +447.0-|
              |      |      +418.0-|      +------Os08g30790
              |      |      |      |
       +497.0-|      +425.0-|      +-------------AT3G01290
       |      |             |
       |      |             |             +------Pt6982556
       |      |             +-------396.0-|
+------|      |                           +------Pt26956 35
|      |      |
|      |      +----------------------------------AT4G27585
|      |
|      +-----------------------------------------pg10 2
|
+------------------------------------------------Pt27399 40
```

Band 7 family protein - PARS

```
                                   +------AT1G69840
                            +460.9-|
                            |      +------Os08g30790
                            |
                     +498.0-|      +------Pt26956 35
                     |      +457.4-|
                     |      |      +------Pt6982556
                     |      |
              +500.0-|      +-------------AT3G01290
              |      |
              |      |             +------Os06g04460
              |      |      +264.8-|
              |      |      |      +------AT5G51570
       +500.0-|      +416.0-|
       |      |             |      +------Pt282 32
       |      |             +497.6-|
+------|      |                    +------pg 4 53
|      |      |
|      |      +---------------------------AT4G27585
|      |
|      +----------------------------------Pt27399 40
|
+-----------------------------------------pg10 2
```

BAM - NJ

```
                                          +------AT5G49660
              +---------------------465.0-|
              |                           +------Os12g43660
              |
              +----------------------------------AT1G09970
              |
              |                           +------Os11g12530
              |                    +401.0-|
              +--------------475.0-|      +------Os01g65650
       +344.0-|                    |
       |      |                    +-------------AT1G72180
       |      |
       |      |                           +------Pt43676
       |      |             +-------450.0-|
       |      |             |             +------Pt72937
       |      |      +342.0-|
       |      |      |      |             +------Pt39682556
       |      |      |      |      +374.0-|
       |      +374.0-|      +420.0-|      +------Pt81728
       |             |             |
       |             |             +-------------pg811033
       |             |
       |             +---------------------------Pt56748
       |
       |                                  +------Os03g05140
       |                           +341.0-|
       |                           |      +------Os02g02140
       |                    +425.0-|
       |                    |      |      +------AT1G08590
       +--------------341.0-|      +418.0-|
+------|                    |             +------AT4G28650
|      |                    |
|      |                    +--------------------AT5G61480
|      |
|      +-----------------------------------------AT4G28490
|      |
|      +-----------------------------------------Os01g13800
|      |
|      +-----------------------------------------Pt68237
|      |
|      |                                  +------Os03g56270
|      +----------------------------321.0-|
|      |                                  +------AT5G65700
|      |
|      |                                  +------Pt42073
|      +----------------------------272.0-|
|      |                                  +------pg14134
|      |
|      +-----------------------------------------pg81079
|      |
|      +-----------------------------------------Pt63256
|      |
|      +-----------------------------------------AT1G28440
|
+------------------------------------------------Os08g28870
```

BAM - PARS

```
                            +--------------------AT5G61480
                            |
                     +281.5-|             +------Os02g02140
                     |      |      +320.6-|
                     |      |      |      +------Os03g05140
                     |      +437.3-|
                     |             |      +------AT4G28650
                     |             +386.9-|
                     |                    +------AT1G08590
                     |
                     |             +-------------pg81079
                     +-------255.6-|
                     |             |      +------AT5G65700
                     |             +500.0-|
                     |                    +------Os03g56270
                     |
                     |                    +------AT5G49660
                     +--------------447.7-|
              +251.2-|                    +------Os12g43660
              |      |
              |      +---------------------------Pt56748
              |      |
              |      |                    +------Pt43676
              |      +--------------418.9-|
              |      |                    +------Pt72937
              |      |
              |      +---------------------------AT1G09970
              |      |
              |      |                    +------Os11g12530
              |      |             +369.1-|
              |      +-------479.7-|      +------Os01g65650
              |      |             |
       +422.7-|      |             +-------------AT1G72180
       |      |      |
       |      |      |             +-------------Pt81728
       |      |      +-------432.4-|
       |      |                    |      +------pg811033
       |      |                    +402.1-|
       |      |                           +------Pt39682556
       |      |
       |      +----------------------------------Pt68237
+------|      |
|      |      +----------------------------------Pt63256
|      |      |
|      |      +----------------------------------Pt42073
|      |      |
|      |      +----------------------------------pg14134
|      |
|      |                                  +------Os01g13800
|      |                                  |
|      +----------------------------251.3-|------AT4G28490
|                                         |
|                                         +------AT1G28440
|
+------------------------------------------------Os08g28870
```

BAS1 cytochromeP450 protein - NJ

```
                                                                        +------Pt47982547
                                     +----------------------------481.0-|
                                     |                                  +------pg8 36
|
                                     +-----------------------------------------Os03g25480
                                     |
                                     |                                  +------Os07g45290
                                     |                           +320.0-|
                                     |                    +484.0-|      +------Os06g39880
                                     |                    |      |
                                     |             +387.0-|      +-------------Os02g11020
                              +340.0-|             |      |
                              |      |      +489.0-|      +--------------------Os01g29150
                              |      |      |      |
                              |      +364.0-|      +---------------------------AT2G26710
                              |      |      |
                              |      |      +----------------------------------Pt12038255
                              |      |
                              |      +-----------------------------------------pg 8 35
                              |      |
                              |      +-----------------------------------------pg 1 95
                              |      |
                              |      +-----------------------------------------Pt8682552
                              |
                       +477.0-|------------------------------------------------Pt72580 19
                       |      |
                       |      |                                         +------Os01g43851
                       |      +-----------------------------------333.0-|
                       |      |                                         +------AT1G17060
                       |      |
                       |      |                                         +------Os01g43710
                       |      |                                  +349.0-|
                +405.0-|      +----------------------------359.0-|      +------Os01g43844
                |      |      |                                  |
                |      |      |                                  +-------------Os01g41810
                |      |      |
                |      |      +------------------------------------------------AT3G14620
         +498.0-|      |      |
         |      |      |      +------------------------------------------------Os01g43740
         |      |      |
         |      |      +-------------------------------------------------------AT3G14610
  +------|      |
  |      |      +--------------------------------------------------------------AT3G14630
  |      |
  |      |                                                              +------AT3G14690
  |      +--------------------------------------------------------295.0-|
  |                                                                     +------AT3G14650
  |
  +----------------------------------------------------------------------------AT3G14680
```

BAS1 cytochromeP450 protein - PARS

```
                                                                             +------Os01g43710
                                                                             |
                                   +-----------------------------------263.3-|------Os01g41810
                                   |                                         |
                                   |                                         +------Os01g43844
                                   |
                                   |                                         +------Os01g29150
                                   |                                  +288.3-|
                                   |                                  |      +------Os02g11020
                                   |                           +381.6-|
                                   |                           |      |      +------Os07g45290
                                   |                    +410.3-|      +316.4-|
                                   |                    |      |             +------Os06g39880
                                   |                    |      |
                                   |                    |      +--------------------AT2G26710
                                   |                    |
                                   |                    +---------------------------Pt8682552
                                   |                    |
                                   |             +254.1-|---------------------------Os03g25480
                                   |             |      |
                            +426.4-|             |      |                    +------pg8 36
                            |      |             |      +--------------480.9-|
                            |      |             |      |                    +------Pt47982547
                            |      |      +316.6-|      |
                            |      |      |      |      +---------------------------Pt12038255
                            |      |      |      |      |
                            |      |      |      |      +---------------------------pg 8 35
                            |      +280.8-|      |
                            |      |      |      +----------------------------------pg 1 95
                            |      |      |
                     +277.8-|      |      +-----------------------------------------Os01g43740
                     |      |      |      |
                     |      |      |      +-----------------------------------------AT3G14620
                     |      |      |
                     |      |      +------------------------------------------------Pt72580 19
                     |      |      |
              +445.9-|      |      +------------------------------------------------Os01g43851
              |      |      |      |
              |      |      |      +------------------------------------------------AT1G17060
              |      |      |
       +441.5-|      |      +-------------------------------------------------------AT3G14610
       |      |      |
       |      |      +--------------------------------------------------------------AT3G14630
+------|      |
|      |      +---------------------------------------------------------------------AT3G14680
|      |
|      +----------------------------------------------------------------------------AT3G14690
|
+-----------------------------------------------------------------------------------AT3G14650
```

bHLH - NJ

```
                                                               +------pg4 137 21
                                                        +411.0-|
       +------------------------------------------256.0-|      +------Pt39056 34
       |                                                |
       |                                                +-------------AT2G27230
       |
       |                                         +--------------------Os07g35870
       |                                         |
       |                    +--------------500.0-|             +------Pp12156 20
       |                    |                    |      +500.0-|
       |                    |                    |      |      +------pg4 130 66
       |                    |                    +341.0-|
       |                    |                           +-------------AT5G54680
       |                    |                           |
       |                    |                           +-------------AT1G51070
       |                    |
       |                    |                                  +------Pt63658 42
       |             +435.0-|             +--------------489.0-|
       |             |      |             |                    +------pg9 137 66
       |             |      |             |
       |             |      |             |                    +------Os01g70310
       |             |      |             |                    |
+------|             |      |      +296.0-|             +443.0-|------AT1G12860
|      |             |      |      |      |             |      |
|      |             |      |      |      |      +488.0-|      +------Os11g32100
|      |             |      |      |      |      |      |
|      |             |      |      |      |      |      |      +------Pt38442 14
|      |             |      +464.0-|      +452.0-|      +500.0-|
|      |      +499.0-|             |             |             +------pg4 78 22
|      |      |      |             |             |
|      |      |      |             |             +--------------------AT2G16910
|      |      |      |             |
|      |      |      |             +----------------------------------AT2G28160
|      |      |      |
|      |      |      |                                         +------Pt77971 34
|      |      |      |                                  +491.0-|
|      |      |      |                                  |      +------pg3 91 13
|      |      |      |                           +271.0-|
|      |      |      |                           |      |      +------AT1G68810
|      |      |      |                           |      +491.0-|
|      |      |      +---------------------470.0-|             +------AT3G25710
|      |      |                                  |
|      +500.0-|                                  |             +------Os03g15440
|             |                                  +-------465.0-|
|             |                                                +------Os08g33590
|             |
|             |                                                +------Pt71807 22
|             |                                         +350.0-|
|             |                           +-------388.0-|      +------pg7 134 25
|             |                           |             |
|             |                           |             +-------------AT5G58010
|             |                    +436.0-|
|             |                    |      |                    +------pg2 115 60
|             |                    |      |      +-------299.0-|
|             |                    |      |      |             +------Pt69504 45
|             |                    |      +373.0-|
|             +--------------439.0-|             |             +------Os09g25040
|                                  |             |      +493.0-|
|                                  |             +466.0-|      +------Os02g55250
|                                  |                    |
|                                  |                    +-------------AT2G24260
|                                  |
|                                  +----------------------------------Os06g09370
|
+---------------------------------------------------------------------Os01g64560
```

bHLH - PARS

```
                                                               +------pg4 130 66
                                                        +374.8-|
                                                        |      +------Pp12156 20
                                                        |
                            +---------------------500.0-|-------------AT1G51070
                            |                           |
                            |                           |      +------AT5G54680
                            |                           +334.7-|
                            |                                  +------Os07g35870
                            |
                            |                                  +------Os01g70310
                            |                           +279.9-|
                            |                    +494.3-|      +------AT1G12860
                            |                    |      |
                            |             +490.9-|      +-------------Os11g32100
                            |             |      |
                            |             |      |             +------Pt38442 14
                            |      +477.8-|      +-------500.0-|
                     +389.0-|      |      |                    +------pg4 78 22
                     |      |      |      |
                     |      +479.0-|      +---------------------------AT2G16910
                     |      |      |
                     |      |      |                    +-------------AT2G28160
                     |      |      +--------------417.9-|
                     |      |                           |      +------pg9 137 66
                     |      |                           +479.3-|
                     |      |                                  +------Pt63658 42
                     |      |
                     |      |                                  +------Os03g15440
                     |      |                    +-------492.6-|
                     |      |                    |             +------Os08g33590
                     |      |                    |
              +500.0-|      +--------------498.0-|      +-------------pg3 91 13
              |      |                           |      |
              |      |                           |      |      +------AT3G25710
              |      |                           +493.2-|-497.1-|
              |      |                                  |      +------AT1G68810
              |      |                                  |
              |      |                                  +-------------Pt77971 34
              |      |
              |      |                                         +------Os09g25040
              |      |                           +-------337.7-|
              |      |                           |             +------Os02g55250
              |      |                           |
              |      |                           +--------------------AT2G24260
       +499.4-|      |                           |
       |      |      +---------------------497.9-|             +------pg7 134 25
       |      |                                  |             |
       |      |                                  |      +328.8-|------Pt71807 22
       |      |                                  |      |      |
       |      |                                  |      |      +------AT5G58010
       |      |                                  |      |
       |      |                                  +385.2-|-------------pg2 115 60
+------|      |                                         |
|      |      |                                         +-------------Os06g09370
|      |      |                                         |
|      |      |                                         +-------------Pt69504 45
|      |      |
|      |      |                                                +------AT2G27230
|      |      +------------------------------------------441.7-|
|      |                                                       +------Os01g64560
|      |
|      +--------------------------------------------------------------pg4 137 21
|
+---------------------------------------------------------------------Pt39056 34
```

bHLH – NJ – genes on chromosome 9

```
                                  +------Pt71807 22
                              +491.0-|
                        +334.0-|      +------Pt69504 45
                        |      |
                        |      +-------------AT2G46510
                        |
                        |             +------pg 9 137 6
                        +-------378.0-|
                  +289.0-|             +------pg 9 133 2
                  |      |
                  |      |             +------Os04g23440
                  |      +-------395.0-|
                  |      |             +------Os04g23550
                  |      |
                  |      +------------------AT5G57150
            +282.0-|
            |      |      +-------------Os01g70310
            |      +-------301.0-|
            |      |      |      +------Pt39056 34
      +447.0-|      |      +465.0-|
      |      |      |             +------Pp12156 20
      |      |      |
+------|      |      +-------------------------AT1G12860
|      |      |
|      |      +-------------------------------Os11g32100
|      |
|      +-------------------------------------AT2G16910
|
+-------------------------------------------Os02g02820
```

bHLH – PARS – genes on chromosome 9

```
+-------------------------------------------------------Os02g02820
|
|                                                +------Os04g23550
|                                         +398.0-|
|                    +--------------250.1-|      +------Os04g23440
|                    |                    |
|                    |                    +-------------AT2G46510
|                    |
|                    |                           +------Pt69504 45
|                    |                    +500.0-|
|                    |                    |      +------Pt71807 22
|                    |             +394.3-|
|             +283.6-|             |      |      +------Pp12156 20
|             |      |      +267.0-|      +473.3-|
|             |      |      |      |             +------Pt39056 34
|             |      +347.2-|      |
|             |      |      |      +--------------------pg 9 133 2
|      +426.6-|      |      |
|      |      |      |      +---------------------------pg 9 137 6
|      |      |      |
|      |      |      +----------------------------------AT5G57150
|      |      |
+------|      |                           +-------------Os01g70310
       |      +---------------------253.9-|
       |                                  |      +------Os11g32100
       |                                  +378.2-|
       |                                         +------AT1G12860
       |
       +------------------------------------------------AT2G16910
```

bZIP - NJ

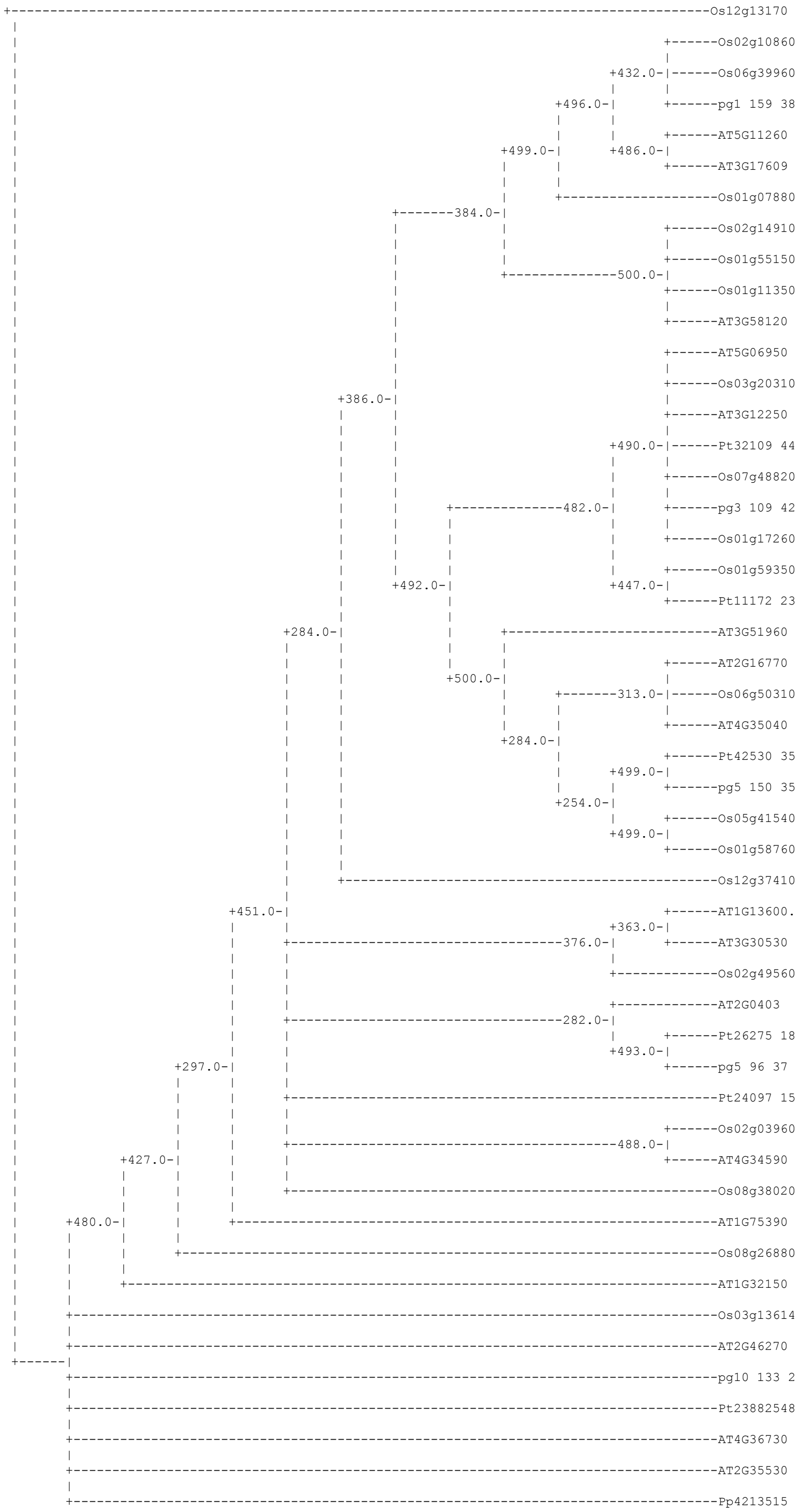
Os12g13170
Os02g10860
+432.0-
Os06g39960
+496.0-
pg1 159 38
AT5G11260
+499.0-
+486.0-
AT3G17609
Os01g07880
384.0-
Os02g14910
Os01g55150
500.0-
Os01g11350
AT3G58120
AT5G06950
Os03g20310
+386.0-
AT3G12250
+490.0-
Pt32109 44
Os07g48820
482.0-
pg3 109 42
Os01g17260
Os01g59350
+492.0-
+447.0-
Pt11172 23
+284.0-
AT3G51960
AT2G16770
+500.0-
313.0-
Os06g50310
AT4G35040
+284.0-
Pt42530 35
+499.0-
pg5 150 35
+254.0-
Os05g41540
+499.0-
Os01g58760
Os12g37410
AT1G13600.
+451.0-
+363.0-
376.0-
AT3G30530
Os02g49560
AT2G0403
282.0-
Pt26275 18
+493.0-
+297.0-
pg5 96 37
Pt24097 15
Os02g03960
488.0-
+427.0-
AT4G34590
Os08g38020
+480.0-
AT1G75390
Os08g26880
AT1G32150
Os03g13614
AT2G46270
pg10 133 2
Pt23882548
AT4G36730
AT2G35530
Pp4213515

bZIP - PARS

```
                                                        +------Pt26275 18
              +-----------------------------------398.9-|
              |                                         +------pg5 96 37
              |
              |                                         +------AT3G58120
              |                                         |
              |                                         +------Os02g14910
              |                           +-------500.0-|
              |                           |             +------Os01g55150
              |                           |             |
              |                           |             +------Os01g11350
              |      +--------------336.0-|
              |      |                    |      +-------------Os01g07880
              |      |                    |      |
              |      |                    |      |      +------Os02g10860
              |      |                    +500.0-|      |
              |      |                           |      +------pg1 159 38
              |      |                           |      |
              |      |                           +470.1-|------AT3G17609
              |      |                                  |
              |      |                                  +------Os06g39960
              |      |                                  |
              |      |                                  +------AT5G11260
              |      |
              |      |                           +-------------Os05g41540
              |      |                           |
              +355.2-|                           |      +------AT2G16770
              |      |                           |      |
              |      |                    +399.5-|-323.9-|------AT4G35040
              |      |                    |      |      |
              |      |                    |      |      +------Os06g50310
              |      |             +270.4-|      |
              |      |             |      |      +-------------Os01g58760
              |      |             |      |
              |      |      +444.0-|      |             +------pg5 150 35
              |      |      |      |      +-------496.0-|
              |      |      |      |                    +------Pt42530 35
              |      |      |      |
              |      |      |      +---------------------------AT3G51960
              |      |      |
              |      |      |                           +------Pt32109 44
              |      +460.0-|                           |
              |             |                           +------Os03g20310
              |             |                           |
       +351.7-|             |                           +------AT5G06950
       |      |             |                           |
       |      |             |                           +------Os01g17260
       |      |             |                           |
       |      |             +---------------------494.0-|------Os07g48820
       |      |                                         |
       |      |                                         +------Pt11172 23
       |      |                                         |
       |      |                                         +------Os01g59350
       |      |                                         |
       |      |                                         +------AT3G12250
       |      |                                         |
       |      |                                         +------pg3 109 42
       |      |
       |      |                                         +------Pt23882548
       |      |                                         |
       |      |                                         +------AT2G46270
       |      |                                         |
       |      |                                         +------AT4G36730
       |      |                                         |
       |      |                                         +------Os12g13170
       |      |                                         |
       |      |                                  +442.7-|------AT1G32150
       |      |                                  |      |
       |      |                                  |      +------Os03g13614
+------|      |                                  |      |
|      |      |                                  |      +------AT2G35530
|      |      |                           +458.9-|      |
|      |      |                           |      |      +------pg10 133 2
|      |      |                           |      |      |
|      |      +---------------------383.0-|      |      +------Pp4213515
|      |      |                           |      |
|      |      |                           |      +-------------Os08g26880
|      |      |                           |
|      |      |                           +--------------------AT1G75390
|      |      |
|      |      +------------------------------------------------Os12g37410
|      |      |
|      |      +------------------------------------------------AT2G0403
|      |      |
|      |      |                           +--------------------AT3G30530
|      |      |                           |
|      |      |                           |      +-------------Os02g49560
|      |      |                           +367.0-|
|      |      +---------------------306.7-|      |      +------Os08g38020
|      |                                  |      +349.6-|
|      |                                  |             +------Pt24097 15
|      |                                  |
|      |                                  +--------------------AT1G13600.
|      |
|      +-------------------------------------------------------Os02g03960
|
+--------------------------------------------------------------AT4G34590
```

C2C2CO-like - NJ

```
                                                                      +------Os04g41560
                                                                      |
                                          +---------------------376.0-|------Os02g39360
                                          |                           |
                                          |                           +------pg1 10 19
                                          |
                                          |                           +------Os01g10580
                                          |                    +454.0-|
                                          |                    |      +------Os06g05890
                                          |             +315.0-|
                                          |             |      |      +------Os12g10660
                                          |             |      +472.0-|
                                          |             |             +------AT4G10240
                                          |      +358.0-|
                                          |      |      |             +------Pt27804 16
                                          |      |      |             |
                                          |      |      |      +498.0-|------Pt9977 21
                                          +393.0-|      |      |      |
                                          |      |      +442.0-|      +------pg8 74 14
                                   +253.0-|      |             |
                                   |      |      |             +-------------AT1G78600
                                   |      |      |
                                   |      |      +---------------------------Os05g11510
                                   |      |
                                   |      |                           +------Os02g43170
                                   |      |                    +499.0-|
                                   |      |                    |      +------Os04g45690
                                   |      +--------------378.0-|
                                   |      |                    |      +------AT1G75540
                                   |      |                    +412.0-|
                                   |      |                           +------AT4G39070
                                   |      |
                                   |      |                           +------Pt73033 20
                                   |      +---------------------453.0-|
                            +425.0-|      |                           +------Pt60782548
                            |      |      |
                            |      |      |                           +------AT1G06040
                            |      |      +---------------------355.0-|
                            |      |                                  +------AT2G31380
                            |      |
                            |      |                                  +------Pt13340 14
                            |      |                           +334.0-|
                            |      |                    +461.0-|      +------pg1 37 41
                     +293.0-|      |                    |      |
                     |      |      +--------------261.0-|      +-------------Pt13342 14
                     |      |      |                    |
                     |      |      |                    |             +------AT2G21320
                     |      |      |                    +-------376.0-|
                     |      |      |                                  +------AT4G38960
                     |      |      |
                     |      |      +-----------------------------------------Os09g35880
                     |      |
                     |      +------------------------------------------------Os08g08120
              +401.0-|
              |      |                                                +------AT3G21880
              |      |                                         +360.0-|
              |      |                                         |      +------Pp11384 10
              |      |                                         |
              |      |                                         +-------------Pp9878 15
              |      |                                         |
              |      |                                         |      +------AT3G07650
              |      |                                         +251.0-|
              |      |                                         |      +------AT5G48250
              |      |                                         |
              |      +-----------------------------------311.0-|-------------pg1 111 12
       +483.0-|                                                |
       |      |                                                +-------------Pt20371 18
       |      |                                                |
       |      |                                                +-------------Os02g49230
       |      |                                                |
       |      |                                                |      +------AT2G33500
       |      |                                                +376.0-|
       |      |                                                |      +------AT1G28050
+------|      |                                                |
|      |      |                                                +-------------Os06g19444
|      |      |
|      |      |                                                       +------AT4G27310
|      |      +-------------------------------------------------417.0-|
|      |                                                              +------AT5G54470
|      |
|      +---------------------------------------------------------------------Pp13171 13
|      |
|      +---------------------------------------------------------------------pg9 135 17
|
+----------------------------------------------------------------------------Pt20182553
```

C2C2CO-like - PARS

```
                                                               +------Pt9977 21
                                                        +272.3-|
                                                 +492.0-|      +------pg8 74 14
                                                 |      |
                                                 |      +-------------Pt27804 16
                                          +396.6-|
                                          |      |             +------Os12g10660
                                          |      |      +476.5-|
                                          |      +304.6-|      +------AT4G10240
                                          |             |
              +---------------------382.0-|             +-------------AT1G78600
              |                           |
              |                           |                    +------Os01g10580
              |                           +--------------267.5-|
              |                           |                    +------Os06g05890
              |                           |
              |                           +---------------------------Os05g11510
              |
              |                                                +------AT5G48250
              |                                         +448.6-|
              |                                         |      +------AT3G07650
              |                    +--------------499.3-|
              |                    |                    +-------------Os06g19444
              |                    |                    |
              |                    |                    +-------------Os02g49230
              |                    |
              |                    |                           +------AT2G33500
              |             +399.0-|             +-------495.2-|
              |             |      |             |             +------AT1G28050
              |             |      |      +339.5-|
              |             |      |      |      |      +-------------pg1 111 12
              |             |      |      |      +425.8-|
              |             |      |      |             |      +------Pt20371 18
              |             |      +255.1-|             +355.6-|
              |             |             |                    +------Pp9878 15
              |      +292.0-|             |
              |      |      |             |                    +------Pp11384 10
              |      |      |             +--------------434.3-|
              |      |      |                                  +------AT3G21880
              |      |      |
              |      |      |                                  +------AT5G54470
              |      |      |                    +-------500.0-|
              +285.0-|      |                    |             +------AT4G27310
              |      |      +--------------371.9-|
       +278.6-|      |                           |             +------pg9 135 17
       |      |      |                           |      +276.1-|
       |      |      |                           +493.2-|      +------Pt20182553
       |      |      |                                  |
       |      |      |                                  +-------------Pp13171 13
       |      |      |
       |      |      +------------------------------------------------Os08g08120
       |      |
       |      |                                                +------Pt60782548
       |      +------------------------------------------415.7-|
       |      |                                                +------Pt73033 20
       |      |
       |      +-------------------------------------------------------Os09g35880
       |      |
       |      +-------------------------------------------------------Os04g45690
       |      |
       |      +-------------------------------------------------------Os02g39360
       |      |
+------|      |                                                +------AT2G31380
|      |      +------------------------------------------428.6-|
|      |      |                                                +------AT1G06040
|      |      |
|      |      +-------------------------------------------------------pg1 10 19
|      |      |
|      |      |                                                +------AT2G21320
|      |      +------------------------------------------267.6-|
|      |      |                                                +------AT4G38960
|      |      |
|      |      +-------------------------------------------------------AT1G75540
|      |      |
|      |      +-------------------------------------------------------AT4G39070
|      |      |
|      |      +-------------------------------------------------------Os02g43170
|      |      |
|      |      +-------------------------------------------------------Os04g41560
|      |
|      |                                                       +------pg1 37 41
|      +-------------------------------------------------496.2-|
|                                                              +------Pt13340 14
|
+---------------------------------------------------------------------Pt13342 14
```

C3H - NJ

```
                     +-----------------------------------------pg1 58
                     |
                     +-----------------------------------------Pt22351 34
                     |
                     |                    +--------------------pg 7 21
                     |                    |
                     |             +302.0-|             +------AT4G29190
                     |             |      |      +314.0-|
                     |             |      +309.0-|      +------AT2G19810
                     |             |             |
                     |             |             +-------------Os01g09620
                     |      +303.0-|
                     |      |      |                    +------AT5G44260
                     |      |      +--------------320.0-|
              +296.0-|      |      |                    +------AT1G03790
              |      +374.0-|      |
              |      |      |      |                    +------Pt13982547
              |      |      |      +--------------487.0-|
              |      |      |                           +------pg 6 135
              |      |      |
              |      |      +----------------------------------Pt6426 33
              |      |
              |      +-----------------------------------------AT5G19390
       +401.0-|      |
       |      |      |                                  +------AT1G26410
       |      |      |                           +448.0-|
       |      |      |                    +274.0-|      +------Pt45152 14
       |      |      |                    |      |
+------|      |      +--------------279.0-|      +-------------pg4 47
|      |      |                           |
|      |      |                           +--------------------Pt41168 14
|      |      |
|      |      +------------------------------------------------Os03g49170
|      |
|      +-------------------------------------------------------AT2G41900
|
+--------------------------------------------------------------AT5G12850
```

C3H - PARS

```
                           +------Pt13982547
       +--------------500.0-|
       |                    +------pg 6 135
       |
       +---------------------------AT1G03790
       |
       +---------------------------AT5G44260
       |
       |             +-------------Os03g49170
       +-------299.1-|
       |             |      +------AT5G12850
       |             +479.8-|
       |                    +------AT2G41900
       |
       +---------------------------AT5G19390
       |
       |      +--------------------pg 7 21
+------|      |
|      +304.9-|             +------AT2G19810
|      |      |      +493.7-|
|      |      +376.9-|      +------AT4G29190
|      |             |
|      |             +-------------Os01g09620
|      |
|      |                    +------Pt45152 14
|      +--------------353.5-|
|      |                    +------pg4 47
|      |
|      +---------------------------Pt6426 33
|      |
|      +---------------------------AT1G26410
|      |
|      |                    +------Pt41168 14
|      +--------------356.8-|
|                           +------pg1 58
|
+----------------------------------Pt22351 34
```

C3HC4 Ring cluster 1.5 - NJ

```
                            +-------------Os05g33000
                     +476.0-|
                     |      |      +------AT5G19080
              +427.0-|      +500.0-|
              |      |             +------AT3G06140
              |      |
              |      +--------------------Os01g27720
              |
              |                    +------Os03g15000
       +405.0-|--------------499.0-|
       |      |                    +------Os10g13670
       |      |
       |      |                    +------pg9 15 19
       |      |             +500.0-|
       |      |             |      +------Pt19996 22
+------|      +-------497.0-|
|      |                    |      +------pg5 85 21
|      |                    +500.0-|
|      |                           +------Pt45282553
|      |
|      |                           +------AT3G09770
|      +---------------------338.0-|
|                                  +------AT3G53410
|
+-----------------------------------------AT5G03200
```

C3HC4 Ring cluster 1.5 - PARS

```
                                                   +------pg5 85 21
                                            +500.0-|
                                            |      +------Pt45282553
                              +-------486.5-|
                              |             |      +------pg9 15 19
                              |             +500.0-|
                       +462.7-|                    +------Pt19996 22
                       |      |
                       |      |      +--------------------Os01g27720
                       |      +488.3-|
                       |             |      +-------------Os05g33000
                +500.0-|             +482.3-|
                |      |                    |      +------AT5G19080
                |      |                    +500.0-|
                |      |                           +------AT3G06140
                |      |
         +------|      |                    +-------------AT3G53410
         |      |      +--------------474.7-|
         |      |                           |      +------AT3G09770
         |      |                           +450.7-|
         |      |                                  +------AT5G03200
         |      |
         |      +-----------------------------------------Os03g15000
         |
         +------------------------------------------------Os10g13670
```

C3HC4 Ring cluster 2.1 - NJ

```
                                                               +------Os02g33720
                                                        +483.0-|
                                                 +257.0-|      +------AT1G49230
                                                 |      |
                                          +498.0-|      +-------------pg10 12 32
                                          |      |
                                   +344.0-|      +--------------------Pt52173 26
                                   |      |
                            +500.0-|      +---------------------------Os01g60730
                            |      |
                     +309.0-|      +----------------------------------AT3G10910
                     |      |
                     |      |                                  +------Os04g37740
              +344.0-|      +----------------------------500.0-|
              |      |                                         +------Os02g36330
              |      |
       +475.0-|      |                                         +------Pt30485 8
       |      |      +-----------------------------------494.0-|
       |      |                                                +------pg3 136 19
+------|      |
|      |      +-------------------------------------------------------AT5G17600
|      |
|      +--------------------------------------------------------------AT4G33565
|
+---------------------------------------------------------------------Os04g50100
```

C3HC4 Ring cluster 2.1 - PARS

```
                                                        +------Pt52173 26
                                                 +306.8-|
                                                 |      +------pg10 12 32
                                          +411.3-|
                                          |      |      +------Os02g33720
                                   +411.5-|      +295.6-|
                                   |      |             +------AT1G49230
                            +491.0-|      |
                            |      |      +--------------------Os01g60730
                            |      |
                     +301.6-|      +---------------------------AT3G10910
                     |      |
                     |      |                           +------Os02g36330
              +274.2-|      +---------------------500.0-|
              |      |                                  +------Os04g37740
              |      |
       +498.9-|      |                                  +------AT4G33565
       |      |      +----------------------------293.4-|
       |      |                                         +------Os04g50100
+------|      |
|      |      +------------------------------------------------AT5G17600
|      |
|      +-------------------------------------------------------Pt30485 8
|
+--------------------------------------------------------------pg3 136 19
```

C3HC4 Ring cluster 2.2 - NJ

```
                                                 +-------------Os03g20870
                                   +-------500.0-|
                                   |             |      +------AT5G56340
                                   |             +484.0-|
                                   |                    +------AT1G55530
                            +393.0-|
                            |      |                    +------Os01g58780
                            |      |             +481.0-|
                            |      |      +281.0-|      +------Os05g41520
                            |      |      |      |
                            |      +496.0-|      +-------------Os03g22830
                            |             |
                     +312.0-|             |             +------AT3G56580
                     |      |             +-------500.0-|
                     |      |                           +------AT2G40830
                     |      |
                     |      |                           +------Pt32507 28
                     |      |                    +500.0-|
              +474.0-|      |             +500.0-|      +------Pt32506 19
              |      |      |             |      |
              |      |      +-------499.0-|      +-------------pg3 104 53
              |      |                    |
       +271.0-|      |                    +--------------------pg3 103 20
       |      |      |
       |      |      +-----------------------------------------Os10g34590
+------|      |
|      |      +------------------------------------------------Os05g01940
|      |
|      +-------------------------------------------------------AT3G46620
|
+--------------------------------------------------------------AT5G59550
```

C3HC4 Ring cluster 2.2 - PARS

```
                            +--------------------Os03g22830
                            |
                     +387.3-|             +------Os05g41520
                     |      |      +474.3-|
                     |      |      |      +------Os01g58780
                     |      +433.8-|
              +441.5-|             |      +------AT2G40830
              |      |             +500.0-|
              |      |                    +------AT3G56580
              |      |
              |      |             +-------------Os03g20870
              |      +-------488.8-|
              |                    |      +------AT5G56340
              |                    +488.5-|
       +485.5-|                           +------AT1G55530
       |      |
       |      +----------------------------------Os10g34590
       |      |
       |      +----------------------------------Os05g01940
       |      |
+------|      +----------------------------------AT3G46620
|      |      |
|      |      +----------------------------------AT5G59550
|      |
|      |                                  +------Pt32507 28
|      |                           +500.0-|
|      +---------------------492.2-|      +------Pt32506 19
|                                  |
|                                  +-------------pg3 104 53
|
+------------------------------------------------pg3 103 20
```

C3HC4 Ring cluster 2.3 - NJ

```
                                                               +------pg1 60 18
                                                 +-------498.0-|
                                                 |             +------Pt67811 19
                                                 |
                                                 |      +-------------AT1G45180
                                                 +353.0-|
                                                 |      |      +------AT4G34040
                                          +489.0-|      +500.0-|
                                          |      |             +------AT2G15530
                                          |      |
                                          |      |             +------AT5G42940
                                          |      +-------374.0-|
                                   +406.0-|      |             +------Os01g47740
                                   |      |      |
                                   |      |      +--------------------Os04g55510
                                   |      |
                            +495.0-|      |                    +------Os01g49770
                            |      |      +--------------500.0-|
                            |      |                           +------Os05g47670
                     +461.0-|      |
                     |      |      +----------------------------------Pt63636 14
                     |      |
                     |      +-----------------------------------------AT4G31450
              +332.0-|
              |      |                                         +------Pt58009 14
              |      +-----------------------------------500.0-|
       +426.0-|      |                                         +------pg6 61 47
       |      |      |
       |      |      +------------------------------------------------Os02g05692
+------|      |
|      |      +-------------------------------------------------------Os06g48040
|      |
|      +--------------------------------------------------------------AT5G24870
|
+---------------------------------------------------------------------AT5G10650
```

C3HC4 Ring cluster 2.3 - PARS

```
                     +----------------------------------Pt63636 14
                     |
                     |                    +-------------Os04g55510
                     |                    |
                     |                    |      +------pg1 60 18
                     |                    +500.0-|
                     |                    |      +------Pt67811 19
                     |             +274.7-|
                     |             |      |      +------AT2G15530
              +384.2-|             |      +500.0-|
              |      |      +446.4-|      |      +------AT4G34040
              |      |      |      |      |
              |      |      |      |      +-------------AT1G45180
              |      +442.6-|      |
              |      |      |      +--------------------AT5G42940
       +453.9-|      |      |
       |      |      |      +---------------------------Os01g47740
       |      |      |
       |      |      |                           +------Os05g47670
       |      |      +---------------------500.0-|
       |      |                                  +------Os01g49770
       |      |
       |      +-----------------------------------------AT4G31450
+------|
|      |                                         +------pg6 61 47
|      +-----------------------------------500.0-|
|      |                                         +------Pt58009 14
|      |
|      |                                         +------AT5G10650
|      +-----------------------------------364.0-|
|      |                                         +------AT5G24870
|      |
|      +------------------------------------------------Os02g05692
|
+-------------------------------------------------------Os06g48040
```

C3HC4 Ring cluster 2.5 - NJ

```
                      +------pg10 22 32
       +-------332.0-|
       |              +------AT1G72200
       |
+------|       +-------------Os08g38460
|      |       |
|      |       +-------------AT1G15100
|      |       |
|      +274.0-|-------------Os02g55520
|              |
|              +-------------AT2G04240
|              |
|              |       +------pg11 18 33
|              +285.0-|
|                      +------Pt29079 20
|
+---------------------------Pt11029 18
```

C3HC4 Ring cluster 2.5 - PARS

```
                                         +-------------AT1G15100
                                  +362.6-|
                                  |      |      +------Os08g38460
                                  |      +283.7-|
                           +318.7-|             +------pg10 22 32
                           |      |
                           |      |             +------Pt29079 20
                    +477.5-|      +-------500.0-|
                    |      |                    +------pg11 18 33
             +441.9-|      |
             |      |      +---------------------------Pt11029 18
      +------|      |
      |      |      +----------------------------------AT2G04240
      |      |
      |      +-----------------------------------------AT1G72200
      |
      +------------------------------------------------Os02g55520
```

Calmodulin - NJ

```
                                          +-------------pg8 24
                            +-------499.0-|
                            |             |      +------Pt97182549
                            |             +500.0-|
                            |                    +------Pp2980 17
                            |
                     +500.0-|                    +------Pt25482547
                     |      |                    |
                     |      |                    +------Pt25382556
                     |      |      +-------500.0-|
                     |      |      |             +------Pt25682552
                     |      |      |             |
                     |      +500.0-|             +------Pp13367 11
                     |             |
              +470.0-|             |             +------AT1G62820
              |      |             |      +500.0-|
              |      |             +498.0-|      +------AT1G12310
              |      |                    |
              |      |                    +-------------Os08g02420
              |      |
              |      |                           +------Pt25682547
              |      |                           |
              |      |                           +------Pt95882549
              |      +---------------------498.0-|
              |                                  +------Pt95882550
              |                                  |
              |                                  +------pg 6 8 19
       +327.0-|
       |      |                                  +------AT3G43810
       |      |                                  |
       |      |                                  +------AT2G27030
       |      |                                  |
       |      +----------------------------432.0-|------AT2G41110
       |      |                                  |
       |      |                                  +------AT5G21274
       |      |                                  |
       |      |                                  +------AT3G56800
+------|      |
|      |      |                                  +------Os07g48780
|      |      |                                  |
|      |      |                                  +------Os01g17190
|      |      +----------------------------300.0-|
|      |                                         +------Os03g20370
|      |                                         |
|      |                                         +------Os01g16240
|      |
|      |                                         +------AT1G66410
|      +-----------------------------------486.0-|
|                                                +------AT5G37780
|
+-------------------------------------------------------Os05g41210
```

Calmodulin - PARS

```
                                                 +------Pp2980 17
                                          +500.0-|
                     +--------------475.9-|      +------Pt97182549
                     |                    |
                     |                    +-------------pg8 24
                     |
                     |                           +------AT2G41110
                     |                           |
                     |                           +------AT3G56800
                     |                           |
                     |                    +293.8-|------AT5G21274
                     |                    |      |
                     |                    |      +------AT2G27030
                     |             +252.2-|      |
              +500.0-|             |      |      +------AT3G43810
              |      |             |      |
              |      |             |      |      +------AT5G37780
              |      |             |      +329.7-|
              |      |             |             +------AT1G66410
              |      |             |
              |      |      +286.3-|--------------------Os07g48780
              |      |      |      |
              |      |      |      +--------------------Os01g16240
              |      |      |      |
              |      |      |      +--------------------Os01g17190
              |      |      |      |
              |      +498.0-|      +--------------------Os03g20370
       +499.0-|             |      |
       |      |             |      +--------------------Os05g41210
       |      |             |
       |      |             |                    +------Pt25682547
       |      |             |                    |
       |      |             |                    +------Pt95882550
       |      |             +--------------303.7-|
       |      |                                  +------Pt95882549
       |      |                                  |
       |      |                                  +------pg 6 8 19
+------|      |
|      |      |                                  +------Pp13367 11
|      |      |                                  |
|      |      |                                  +------Pt25682552
|      |      +----------------------------499.0-|
|      |                                         +------Pt25382556
|      |                                         |
|      |                                         +------Pt25482547
|      |
|      |                                         +------AT1G62820
|      +-----------------------------------500.0-|
|                                                +------AT1G12310
|
+-------------------------------------------------------Os08g02420
```

CAOMT - NJ

```
                     +----------------------------------Os06g06980
                     |
                     |                           +------Os08g38920
                     |                    +373.0-|
                     |                    |      +------Os09g30360
                     |             +295.0-|
                     |             |      |      +------Os08g38910
                     |      +302.0-|      +415.0-|
                     |      |      |             +------Os08g38900
                     |      |      |
                     |      |      +--------------------AT4G26220
                     +360.0-|
                     |      |                    +------AT1G67980
                     |      |      +-------500.0-|
              +500.0-|      |      |             +------AT1G67990
              |      |      +309.0-|
              |      |             |      +-------------pg2 129 32
              |      |             +500.0-|
              |      |                    |      +------Pt19982549
              |      |                    +500.0-|
              |      |                           +------Pp6003 13
              |      |
       +500.0-|      +----------------------------------Pt74682547
       |      |      |
       |      |      +----------------------------------pg9 125 43
       |      |      |
       |      |      +----------------------------------Pp6404 24
+------|      |      |
|      |      |      +----------------------------------AT4G34050
|      |      |
|      |      +-----------------------------------------Pp3037 11
|      |
|      +------------------------------------------------Pt18200 16
|
+-------------------------------------------------------pg2 82 51
```

CAOMT - PARS

```
       +--------------------pg9 125 43
       |
       |             +------AT1G67980
       +-------500.0-|
       |             +------AT1G67990
       |
       |      +-------------Pp3037 11
       +500.0-|
       |      |      +------pg2 82 51
       |      +464.8-|
       |             +------Pt18200 16
       |
       |      +-------------Os08g38910
       |      |
       |      |      +------Os09g30360
       +380.7-|-395.9-|
+------|      |      +------Os08g38920
|      |      |
|      |      +-------------Os08g38900
|      |
|      |             +------Pt19982549
|      |      +498.1-|
|      +500.0-|      +------Pp6003 13
|      |      |
|      |      +-------------pg2 129 32
|      |
|      +--------------------AT4G26220
|      |
|      +--------------------Pp6404 24
|      |
|      +--------------------Pt74682547
|      |
|      +--------------------AT4G34050
|
+---------------------------Os06g06980
```

CCAAT-box binding - NJ

```
              +----------------------------------Os03g14669
              |
              +----------------------------------Pt15100 17
              |
              |                           +------Pt57094 20
              |             +-------448.0-|
              |             |             +------Os06g45640
              |      +257.0-|
              |      |      |             +------AT1G08970
       +492.0-|      |      |      +497.0-|
       |      +499.0-|      +493.0-|      +------AT1G54830
       |      |      |             |
       |      |      |             +-------------Os02g07450
       |      |      |
+------|      |      +---------------------------AT1G56170
|      |      |
|      |      +----------------------------------pg 12 60 3
|      |      |
|      |      +----------------------------------pg3 3 96 1
|      |
|      +-----------------------------------------AT3G48590
|
+------------------------------------------------AT5G63470
```

CCAAT-box binding - PARS

```
                            +------Pt57094 20
                            |
              +-------331.1-|------Os06g45640
              |             |
              |             +------AT1G56170
       +494.3-|
       |      |             +------AT1G08970
       |      |      +491.6-|
       |      +264.4-|      +------AT1G54830
       |             |
       |             +-------------Os02g07450
       |
+------|                    +------AT3G48590
|      +--------------493.3-|
|      |                    +------AT5G63470
|      |
|      +---------------------------pg3 3 96 1
|      |
|      +---------------------------Pt15100 17
|      |
|      +---------------------------pg 12 60 3
|
+----------------------------------Os03g14669
```

CCA1 like - NJ

```
+-----------------------------------------Os01g09280
|
|                    +--------------------pg2 81 87
|                    |
|             +488.0-|             +------Pt34782549
|             |      |      +269.0-|
|             |      +276.0-|      +------Pt31654 18
|             |             |
|             |             +-------------pg5 66 20
|             |
|             +---------------------------AT5G61620
|             |
|             +---------------------------Os05g51160
|             |
|             |                    +------Os10g41200
|             +--------------279.0-|
|             |                    +------AT5G56840
|             |
|             |             +-------------Os01g41900
|             +-------268.0-|
|      +469.0-|             |      +------Os01g09640
|      |      |             +264.0-|
|      |      |                    +------Os05g10690
|      |      |
|      |      +---------------------------AT3G16350
|      |      |
|      |      +---------------------------Os08g04840
|      |      |
|      |      |             +-------------Pt33049 32
+------|      +-------500.0-|
       |      |             |      +------Pt33098 17
       |      |             +362.0-|
       |      |                    +------pg2 19 19
       |      |
       |      +---------------------------Os08g05510
       |      |
       |      +---------------------------AT1G70000
       |
       +----------------------------------AT5G47390
```

CCA1 like - PARS

```
                     +------pg2 81 87
                     |
                     +------Os05g51160
                     |
                     +------Pt31654 18
                     |
                     +------AT5G56840
                     |
                     +------Os01g41900
                     |
                     +------Os08g04840
              +258.0-|
              |      +------pg5 66 20
              |      |
              |      +------Os01g09640
              |      |
              |      +------AT5G61620
              |      |
              |      +------Pt34782549
              |      |
              |      +------Os08g05510
              |      |
       +372.2-|      +------Os05g10690
       |      |
       |      |      +------Pt33098 17
       |      |      |
       |      +495.7-|------pg2 19 19
       |      |      |
       |      |      +------Pt33049 32
+------|      |
|      |      +-------------Os10g41200
|      |      |
|      |      +-------------AT3G16350
|      |      |
|      |      +-------------AT1G70000
|      |
|      +--------------------AT5G47390
|
+---------------------------Os01g09280
```

CCR - NJ

```
+----------------------------------------------------------------------------Os03g60380
|
|                                                                     +------AT1G76470
|                           +-----------------------------------351.0-|
|                           |                                         +------Os01g61230
|                           |
|                           |      +-----------------------------------------AT1G51410
|                           |      |
|                           |      |                           +-------------Pt57482553
|                           |      |                    +497.0-|
|                           |      |                    |      |      +------Pt59448 19
|                           |      |                    |      +324.0-|
|                           |      |             +402.0-|             +------Pt32642 21
|                           +447.0-|             |      |
|                           |      |             |      |             +------Os09g04050
|                           |      |             |      +-------404.0-|
|                           |      |      +432.0-|                    +------Os02g08420
|                    +358.0-|      |      |      |
|                    |      |      |      |      |                    +------Pt13691 26
|                    |      |      |      |      |             +496.0-|
|                    |      |      +500.0-|      +-------261.0-|      +------pg3 23put
|                    |      |             |                    |
|                    |      |             |                    +-------------AT1G80820
|                    |      |             |
|                    |      |             +----------------------------------Os09g25150
|                    |      |
|             +350.0-|      |                                         +------Os01g74660
|             |      |      +-----------------------------------286.0-|
|             |      |      |                                         +------AT2G02400
|             |      |      |
|             |      |      |                                         +------Pt40813 21
|             |      |      +-----------------------------------500.0-|
|      +356.0-|      |                                                +------pg11 111
|      |      |      |
|      |      |      |                                                +------pg11 143
|      |      |      +------------------------------------------500.0-|
+------|      |                                                       +------Pt63095 30
       |      |
       |      +--------------------------------------------------------------Pt16803 15
       |
       +---------------------------------------------------------------------AT5G58490
```

CCR - PARS

```
                                   +-------------Pt57482553
                            +489.8-|
                            |      |      +------Pt32642 21
                            |      +313.9-|
                            |             +------Pt59448 19
                            |
                            +--------------------AT1G80820
                     +500.0-|
                     |      |             +------Os09g04050
                     |      +-------432.1-|
                     |      |             +------Os02g08420
                     |      |
              +349.9-|      |      +-------------Os09g25150
              |      |      +388.1-|
              |      |             |      +------pg3 23put
              |      |             +481.8-|
              |      |                    +------Pt13691 26
              |      |
              |      +---------------------------AT1G51410
              |
       +500.0-|                    +-------------Pt16803 15
       |      |                    |
       |      |                    |      +------Os01g61230
       |      |                    +422.6-|
       |      |                    |      +------AT1G76470
       |      |                    |
       |      |                    |      +------Pt63095 30
       |      |                    +500.0-|
       |      +--------------264.2-|      +------pg11 143
+------|                           |
|      |                           +-------------Os03g60380
|      |                           |
|      |                           |      +------AT2G02400
|      |                           +417.8-|
|      |                           |      +------Os01g74660
|      |                           |
|      |                           +-------------AT5G58490
|      |
|      +-----------------------------------------Pt40813 21
|
+------------------------------------------------pg11 111
```

CESA - NJ

```
                                                                             +------AT4G18780
                                                        +--------------463.0-|
                                                        |                    +------Os01g54620
                                                        |
                                                 +222.0-|                    +------pg 9 39
                                                 |      |             +500.0-|
                                                 |      |      +225.0-|      +------Pt49300 59
                                                 |      |      |      |
                                          +431.0-|      +208.0-|      +-------------AT5G44030
                                          |      |             |
                                          |      |             +--------------------Os10g32980
                                          |      |
                                          |      |                           +------Pt38482553
                                          |      +---------------------499.0-|
                                   +390.0-|                                  +------pg11 114pu
                                   |      |
                                   |      |                                  +------Os07g24190
                                   |      |                           +392.0-|
                                   |      |             +-------500.0-|      +------Os07g14850
                                   |      |             |             |
                                   |      |             |             +-------------Os03g62090
                                   |      +-------367.0-|
                                   |                    |      +--------------------AT2G21770
                            +271.0-|                    |      |
                            |      |                    +493.0-|             +------AT5G64740
                            |      |                           |      +343.0-|
                            |      |                           +361.0-|      +------AT5G09870
                            |      |                                  |
                            |      |                                  +-------------AT4G39350
                     +277.0-|      |
                     |      |      |                                         +------AT5G17420
                     |      |      |                                  +498.0-|
                     |      |      +----------------------------228.0-|      +------Os09g25490
                     |      |                                         |
              +459.0-|      |                                         +-------------Pt44831 70
              |      |      |
              |      |      +-------------------------------------------------------pg4 51
              |      |
              |      |                                                       +------Os03g59340
       +351.0-|      |                                                +490.0-|
       |      |      +------------------------------------------494.0-|      +------AT5G05170
       |      |                                                       |
       |      |                                                       +-------------Os07g10770
       |      |
       |      |                                                       +-------------Pp4713522
+------|      +-------------------------------------------------500.0-|
|      |                                                              |      +------pg1 163put
|      |                                                              +303.0-|
|      |                                                                     +------Pt482549 3
|      |
|      |                                                                     +------AT2G25540
|      +---------------------------------------------------------------474.0-|
|                                                                            +------AT4G32410
|
+-----------------------------------------------------------------------------------Os05g08370
```

CESA - PARS

```
       +--------------------------------------------------------------AT4G32410
       |
       |                                                       +------Os03g62090
       |                                                       |
       |                                         +-------494.4-|------Os07g14850
       |                                         |             |
       |                                         |             +------Os07g24190
       |                                  +373.0-|
       |                                  |      |             +------AT5G09870
       |                                  |      |      +260.9-|
       |                                  |      |      |      +------AT5G64740
       |                                  |      +496.2-|
       |                                  |             +-------------AT4G39350
       |                                  |             |
       |                                  |             +-------------AT2G21770
       |                           +353.4-|
       |                           |      |                    +------Pt49300 59
       |                           |      |             +494.7-|
       |                           |      |             |      +------pg 9 39
       |                           |      |      +271.7-|
       |                           |      |      |      |      +------Os10g32980
       |                           |      |      |      +300.9-|
       |                           |      |      |             +------AT5G44030
+------|                           |      |      |
|      |                           |      +437.8-|             +------Pt38482553
|      |                           |             +-------421.4-|
|      |                    +355.2-|             |             +------pg11 114pu
|      |                    |      |             |
|      |                    |      |             |             +------Os01g54620
|      |                    |      |             +-------456.8-|
|      |                    |      |                           +------AT4G18780
|      |                    |      |
|      |                    |      +----------------------------------Pt44831 70
|      |                    |      |
|      |             +494.2-|      +----------------------------------pg4 51
|      |             |      |      |
|      |             |      |      |                           +------Os09g25490
|      |             |      |      +---------------------485.8-|
|      |             |      |                                  +------AT5G17420
|      |             |      |
|      |             |      |                                  +------AT5G05170
|      |      +490.9-|      |                           +500.0-|
|      |      |      |      +---------------------497.9-|      +------Os03g59340
|      |      |      |                                  |
|      |      |      |                                  +-------------Os07g10770
|      |      |      |
|      +498.4-|      |                                         +------pg1 163put
|             |      |                                         |
|             |      +-----------------------------------490.6-|------Pp4713522
|             |                                                |
|             |                                                +------Pt482549 3
|             |
|             +-------------------------------------------------------Os05g08370
|
+---------------------------------------------------------------------AT2G25540
```

Chalcone synthase - NJ

```
              +--------------------------------------------------------------AT5G13930
              |
              |                                                       +------AT4G00040
              |                                                +497.0-|
              |                    +---------------------500.0-|      +------AT1G02050
              |                    |                           |
              |                    |                           +-------------AT4G34850
              |                    |
              |                    |             +---------------------------Os07g17010
       +500.0-|             +370.0-|             |
       |      |             |      |             |                    +------Os10g07040
       |      |             |      |      +433.0-|             +497.0-|
       |      |             |      |      |      |      +500.0-|      +------Os10g08620
       |      |             |      |      |      |      |      |
       |      |      +304.0-|      +496.0-|      +439.0-|      +-------------Os10g09860
       |      |      |      |             |             |
       |      |      |      |             |             +--------------------Os07g34140
       |      |      |      |             |
       |      +489.0-|      |             +----------------------------------Os07g31770
       |             |      |
       |             |      +------------------------------------------------Os07g11440
       |             |
       |             +-------------------------------------------------------Os11g32650
       |
+------|                                                       +-------------Pp5313518
|      |                                                       |
|      |                                                       +-------------Pt7982551
|      |                                                +487.0-|
|      |                                                |      +-------------Pt44382548
|      |                                                |      |
|      |                                                |      |      +------pg7 95 57
|      |                                                |      +259.0-|
|      |                                                |             +------Pt48269 22
|      |                                         +460.0-|
|      |                                         |      |             +------Pt11578254
|      |                                         |      |             |
|      |                                         |      |             +------Pp9506 12
|      |                                         |      |             |
|      |                                         |      |             +------Pt11061 20
|      |                                         |      +-------487.0-|
|      +-----------------------------------263.0-|                    +------pg7 96 28
|                                                |                    |
|                                                |                    +------Pt29282548
|                                                |                    |
|                                                |                    +------Pp9779 20
|                                                |
|                                                |                    +------pg7 97 8
|                                                +--------------316.0-|
|                                                                     +------Pp9505 18
|
+----------------------------------------------------------------------------Pt36482553
```

Chalcone synthase - PARS

```
                                                                      +------AT1G02050
                                                               +500.0-|
                                   +---------------------500.0-|      +------AT4G00040
                                   |                           |
                                   |                           +-------------AT4G34850
                                   |
                                   |             +---------------------------Os07g17010
                            +415.9-|             |
                            |      |             |                    +------Os10g07040
                            |      |      +425.9-|             +500.0-|
                            |      |      |      |      +500.0-|      +------Os10g08620
                            |      |      |      |      |      |
                            |      +467.7-|      +276.3-|      +-------------Os10g09860
                     +445.1-|             |             |
                     |      |             |             +--------------------Os07g34140
                     |      |             |
                     |      |             +----------------------------------Os07g31770
              +500.0-|      |
              |      |      |                                         +------Os11g32650
              |      |      +-----------------------------------363.0-|
              |      |                                                +------Os07g11440
              |      |
              |      +-------------------------------------------------------AT5G13930
              |
              |                                                       +------Pt48269 22
              |                                                       |
              |                                                       +------Pt7982551
              |                                                       |
       +301.4-|-------------------------------------------------270.0-|------pg7 95 57
       |      |                                                       |
       |      |                                                       +------Pp5313518
       |      |                                                       |
       |      |                                                       +------Pt44382548
       |      |
       |      |                                                       +------pg7 96 28
       |      |                                                       |
       |      |                                                       +------Pt11061 20
       |      |                                                       |
+------|      |                                                       +------Pp9779 20
|      |      +-------------------------------------------------267.6-|
|      |                                                              +------Pt29282548
|      |                                                              |
|      |                                                              +------Pt11578254
|      |                                                              |
|      |                                                              +------Pp9506 12
|      |
|      +---------------------------------------------------------------------Pt36482553
|      |
|      +---------------------------------------------------------------------pg7 97 8
|
+----------------------------------------------------------------------------Pp9505 18
```

Chaperonin - NJ

```
                                                                      +-------------Pt64982553
                     +------------------------------------------500.0-|
                     |                                                |      +------Pp13518 27
                     |                                                +251.0-|
                     |                                                       +------pg 1 128
                     |
                     |                                                       +------Os06g36700
                     |                                                +489.0-|
                     |                    +---------------------500.0-|      +------AT1G24510
                     |                    |                           |
                     |                    |                           +-------------Pt29652 20
                     |                    |
                     |                    |                    +--------------------Os06g34690
                     |                    |                    |
                     |             +115.0-|             +358.0-|             +------pg 5 29 26
              +392.0-|             |      |             |      |      +406.0-|
              |      |             |      |             |      +487.0-|      +------Pt26982553
              |      |             |      |      +500.0-|             |
              |      |             |      |      |      |             +-------------AT5G26360
              |      |             |      |      |      |
              |      |             |      +113.0-|      +---------------------------Os02g14929
              |      |             |             |
              |      |      +465.0-|             |                           +------Pp11703 46
              |      |      |      |             +---------------------500.0-|
              |      |      |      |                                         +------AT3G18190
              |      |      |      |
              |      |      |      |                                  +-------------AT3G11830
              |      |      |      |                           +500.0-|
              |      |      |      |                           |      |      +------Os06g47320
       +370.0-|      |      |      |                           |      +488.0-|
       |      |      +498.0-|      +---------------------257.0-|             +------Pt56120 37
       |      |             |                                  |
       |      |             |                                  |             +------AT3G20050
       |      |             |                                  +-------500.0-|
       |      |             |                                                +------Os04g46620
       |      |             |
       |      |             |                                                +------Os05g48290
+------|      |             |                                         +344.0-|
|      |      |             |                                         |      +------Os03g42220
|      |      |             +-----------------------------------500.0-|
|      |      |                                                       |      +------AT5G20890
|      |      |                                                       +378.0-|
|      |      |                                                              +------pg 5 71
|      |      |
|      |      +---------------------------------------------------------------------Os05g05470
|      |
|      +----------------------------------------------------------------------------AT3G02530
|
+-----------------------------------------------------------------------------------AT5G16070
```

Chaperonin - PARS

```
                                                 +--------------------AT5G20890
                                                 |
                     +---------------------500.0-|             +------Os03g42220
                     |                           |      +406.8-|
                     |                           +370.0-|      +------Os05g48290
                     |                                  |
                     |                                  +-------------pg 5 71
                     |
                     |                                         +------Os04g46620
                     |                    +--------------500.0-|
                     |                    |                    +------AT3G20050
                     |                    |
              +500.0-|             +375.9-|                    +------Os02g14929
              |      |             |      |             +446.2-|
              |      |             |      |      +396.9-|      +------Os06g34690
              |      |             |      |      |      |
              |      |             |      +500.0-|      +-------------AT5G26360
              |      |      +346.4-|             |
              |      |      |      |             |             +------Pt26982553
              |      |      |      |             +-------484.0-|
              |      |      |      |                           +------pg 5 29 26
              |      |      |      |
              |      |      |      |                           +------Pp11703 46
              |      +489.1-|      +---------------------500.0-|
              |             |                                  +------AT3G18190
              |             |
       +379.0-|             |                                  +------Os06g36700
       |      |             |                           +487.3-|
       |      |             |                    +500.0-|      +------AT1G24510
       |      |             |                    |      |
       |      |             +--------------356.6-|      +-------------Pt29652 20
       |      |                                  |
       |      |                                  |      +-------------AT3G11830
       |      |                                  +500.0-|
       |      |                                         |      +------Os06g47320
+------|      |                                         +494.1-|
|      |      |                                                +------Pt56120 37
|      |      |
|      |      |                                                +------Pp13518 27
|      |      |                                                |
|      |      +------------------------------------------500.0-|------pg 1 128
|      |                                                       |
|      |                                                       +------Pt64982553
|      |
|      |                                                       +------AT3G02530
|      +-------------------------------------------------454.4-|
|                                                              +------AT5G16070
|
+---------------------------------------------------------------------Os05g05470
```

Chlorophyll A-B binding protein - NJ

```
                                                                +--------------Pt81771 17
                                                        +223.0-|
                                                        |       |       +------pg 11 6
                                                        |       +-88.0-|
                                                +392.0-|               +------Os01g41710
                                                |       |
                                                |       |               +------Pt99782547
                                        +221.0-|       +-------489.0-|
                                        |       |                       +------Os01g52240
                                +338.0-|       |
                                |       |       +------------------------------Os03g39610
                        +500.0-|       |
                        |       |       +--------------------------------------AT2G34430
                +500.0-|       |
                |       |       +----------------------------------------------AT1G29930
        +500.0-|       |
        |       |       +------------------------------------------------------AT3G54890
+------|       |
|       |       +--------------------------------------------------------------pg 7 46
|       |
|       +----------------------------------------------------------------------AT5G01530
|
+------------------------------------------------------------------------------Os07g37240
```

Chlorophyll A-B binding protein - PARS

```
                            +-------------pg 11 6
                            |
                            +-------------Pt81771 17
                            |
              +-------250.3-|      +------Os01g52240
              |             +365.0-|
              |             |      +------Pt99782547
       +258.3-|             |
       |      |             +-------------Os01g41710
       |      |
       |      |      +--------------------AT3G54890
       |      +500.0-|
       |             |      +-------------pg 7 46
+------|             +500.0-|
|      |                    |      +------Os07g37240
|      |                    +500.0-|
|      |                           +------AT5G01530
|      |
|      +----------------------------------AT2G34430
|      |
|      +----------------------------------AT1G29930
|
+-----------------------------------------Os03g39610
```

4CL - NJ

```
                                                        +------Pt81267 41
                                                 +433.0-|
                                                 |      +------pg6 86
                                          +500.0-|
                                          |      |      +------Pt53499 23
                                          |      +472.0-|
                                   +479.0-|             +------Pt30285 16
                                   |      |
                                   |      |             +------Pt90982550
                            +272.0-|      +-------500.0-|
                            |      |                    +------pg3 82
                            |      |
                            |      |                    +------AT1G65060.
                     +491.0-|      +--------------476.0-|
                     |      |                           +------Os02g46970
                     |      |
                     |      |                           +------Os06g44620
              +303.0-|      |                    +494.0-|
              |      |      +--------------425.0-|      +------Os02g08100
              |      |                           |
       +414.0-|      |                           +-------------Os08g14760
       |      |      |
       |      |      +-----------------------------------------Os08g34790
+------|      |
|      |      +------------------------------------------------AT3G21230.
|      |
|      +-------------------------------------------------------AT3G21240.
|
+--------------------------------------------------------------AT1G51680.
```

4CL - PARS

```
                                                 +------Pt30285 16
                                          +382.8-|
                                   +330.0-|      +------Pt53499 23
                                   |      |
                            +494.0-|      +-------------Pt81267 41
                            |      |
                     +262.7-|      +--------------------pg6 86
                     |      |
                     |      |                    +------Pt90982550
                     |      +--------------489.2-|
              +350.0-|                           +------pg3 82
              |      |
              |      |                           +------AT1G65060.
              |      |             +-------406.8-|
              |      |             |             +------Os02g46970
              |      +-------279.1-|
              |                    |      +-------------Os08g14760
       +293.5-|                    +418.9-|
       |      |                           |      +------Os02g08100
       |      |                           +494.7-|
       |      |                                  +------Os06g44620
+------|      |
|      |      +-----------------------------------------Os08g34790
|      |      |
|      |      +-----------------------------------------AT1G51680.
|      |
|      +------------------------------------------------AT3G21240.
|
+-------------------------------------------------------AT3G21230.
```

Cyclin - NJ

```
                                                                                    +------AT1G16330
                                                                             +245.0-|
                                                                             |      +------pg4 33
                                          +-----------------------------------318.0-|
                                          |                                  |      +------Pt13782554
                                          |                                  +500.0-|
                                          |                                         +------pg10 21
                                          |
                                          |                                  +-------------AT4G35620
                                          |                           +296.0-|
                                   +320.0-|                           |      |      +------AT1G76310
                                   |      |                    +303.0-|      +500.0-|
                                   |      |                    |      |             +------AT1G20610
                                   |      |             +402.0-|      |
                                   |      |             |      |      +--------------------Os06g51110
                                   |      |      +498.0-|      |
                            +343.0-|      |      |      |      +---------------------------Os04g47580
                            |      |      +307.0-|      |
                            |      |             |      +----------------------------------Pt28241 13
                            |      |             |
                            |      |             +-----------------------------------------Pt35186 24
                     +485.0-|      |
                     |      |      |                                                +------Os01g59120
                     |      |      +------------------------------------------465.0-|
              +494.0-|      |                                                       +------Os01g17402
              |      |      |
              |      |      +--------------------------------------------------------------Os05g41390
       +500.0-|      |
       |      |      +---------------------------------------------------------------------AT2G26760
+------|      |
|      |      +----------------------------------------------------------------------------AT4G37490
|      |
|      +-----------------------------------------------------------------------------------AT3G11520
|
+------------------------------------------------------------------------------------------AT5G06150
```

Cyclin - PARS

```
+----------------------------------------------------------------------------AT4G37490
|
|                                                       +--------------------Os04g47580
|                                                       |
|                                                +335.1-|--------------------Os06g51110
|                                                |      |
|                                                |      |      +-------------AT4G35620
|                                                |      +367.3-|
|                                         +463.5-|             |      +------AT1G20610
|                                         |      |             +488.4-|
|                                         |      |                    +------AT1G76310
|                                  +415.4-|      |
|                                  |      |      +---------------------------Pt28241 13
|                                  |      |
|                                  |      +----------------------------------Pt35186 24
|                           +393.5-|
|                           |      +-----------------------------------------AT1G16330
|                           |      |
|                           |      |                                  +------pg10 21
|                           |      |                           +500.0-|
|                    +377.4-|      +---------------------287.4-|      +------Pt13782554
|                    |      |                                  |
|                    |      |                                  +-------------pg4 33
|                    |      |
|             +445.0-|      |                                         +------Os01g17402
|             |      |      +-----------------------------------309.5-|
|             |      |                                                +------Os01g59120
|      +321.6-|      |
|      |      |      +-------------------------------------------------------Os05g41390
|      |      |
+------|      +--------------------------------------------------------------AT2G26760
       |
       |                                                              +------AT5G06150
       +--------------------------------------------------------500.0-|
                                                                      +------AT3G11520
```

Cysteine proteinase - NJ

```
                                                                             +------AT4G39090
                                                                      +500.0-|
              +-------------------------------------------------494.0-|      +------AT2G21430
              |                                                       |
              |                                                       +-------------AT4G16190
              |
              |                                                              +------AT4G35350
              |                                                {+-------500.0-|
              |                                                |             +------Os01g73980
              |                                  +-------273.0-|
              |                                  |             |      +-------------Pt9782547
              |                                  |             +500.0-|
              |                                  |                    |      +------Pt42197 29
              |                                  |                    +316.0-|
              |                                  |                           +------pg3 3 35 8
              |                                  |
              |                                  |                           +------Os11g14900
              |                                  |      +--------------500.0-|
              |                                  |      |                    +------AT5G50260
              |                                  |      |
              |                                  +368.0-|                    +------Pt4025 25
              |                                  |      |             +380.0-|
              |                                  |      |      +499.0-|      +------pg8 140
              |                           +444.0-|      |      |      |
       +500.0-|                           |      |      +500.0-|      +-------------Pt49182549
       |      |                           |      |             |
       |      |                           |      |             +--------------------Pt94782554
       |      |                           |      |
       |      |                           |      |                           +------Os04g57490
       |      |                           |      |                    +500.0-|
       |      |                           |      |                    |      +------Os04g57440
       |      |                    +500.0-|      +-------------459.0-|
       |      |                    |      |      |                    |      +------AT3G19390
       |      |                    |      |      |                    +324.0-|
       |      |                    |      |      |                           +------AT4G36880
       |      |                    |      |      |
       |      |             +300.0-|      |      |                           +------AT1G09850
       |      |             |      |      |      +---------------------462.0-|
       |      |             |      |      |                                  +------Os05g43230
+------|      |             |      |      |
|      |      |             |      |      +-----------------------------------------AT5G60360
|      |      |      +416.0-|      |
|      |      |      |      |      +------------------------------------------------Os07g29760
|      |      |      |      |
|      |      |      |      |                                                +------Pt63982549
|      |      |      |      |                                         +500.0-|
|      |      +400.0-|      +-----------------------------------284.0-|      +------Pp8674 22
|      |             |                                                |
|      |             |                                                +-------------AT3G54940
|      |             |
|      |             |                                                       +------Pt63989 22
|      |             +-------------------------------------------------500.0-|
|      |                                                                     +------pg1 82
|      |
|      +----------------------------------------------------------------------------Os04g24600
|
+-----------------------------------------------------------------------------------Os02g27030
```

Cysteine proteinase - PARS

```
                                                 +------pg8 140
                                                 |
                                          +479.0-|------Pt4025 25
                                          |      |
                                   +500.0-|      +------Pt49182549
                                   |      |
                            +292.5-|      +-------------Pt94782554
                            |      |
                            |      |             +------Os11g14900
                            |      +-------500.0-|
                            |                    +------AT5G50260
                            |
                            |                    +------Os04g57490
                            +--------------500.0-|
                            |                    +------Os04g57440
                            |
                            |                    +------pg3 3 35 8
                            |             +370.1-|
                            +-------500.0-|      +------Pt42197 29
                     +459.3-|             |
                     |      |             +-------------Pt9782547
                     |      |
                     |      |                    +------Os05g43230
                     |      +--------------466.6-|
                     |      |                    +------AT1G09850
                     |      |
              +500.0-|      +---------------------------AT4G35350
              |      |      |
              |      |      +---------------------------Os01g73980
              |      |      |
              |      |      +---------------------------AT3G19390
              |      |      |
              |      |      +---------------------------AT4G36880
              |      |
              |      +----------------------------------AT5G60360
              |
              |                                  +------Pt63989 22
              +----------------------------500.0-|
       +463.5-|                                  +------pg1 82
       |      |
       |      |                                  +------Pp8674 22
       |      +----------------------------500.0-|
       |      |                                  +------Pt63982549
       |      |
       |      |                           +-------------AT4G16190
       |      +---------------------382.9-|
+------|      |                           |      +------AT4G39090
|      |      |                           +499.2-|
|      |      |                                  +------AT2G21430
|      |      |
|      |      |                                  +------AT3G54940
|      |      +----------------------------292.1-|
|      |                                         +------Os07g29760
|      |
|      +------------------------------------------------Os02g27030
|
+-------------------------------------------------------Os04g24600
```

Cysteine synthase - NJ

```
                                   +-------------Os12g42980
                            +379.0-|
                            |      |      +------Pt20299 21
                            |      +263.0-|
                     +498.0-|             +------Pt11338255
                     |      |
                     |      |             +------Pt84982556
                     |      |      +500.0-|
              +324.0-|      +500.0-|      +------Pp7183 23
              |      |             |
              |      |             +-------------pg 8 129 6
       +500.0-|      |
       |      |      +---------------------------Os01g74650
       |      |
+------|      |                           +------AT3G59760
|      |      +---------------------189.0-|
|      |                                  +------AT2G43750
|      |
|      +-----------------------------------------Pt46782548
|
+------------------------------------------------pg1 21
```

Cysteine synthase - PARS

```
                            +-------------Os12g42980
                     +322.4-|
                     |      |      +------Pt20299 21
                     |      +319.4-|
              +487.0-|             +------Pt11338255
              |      |
              |      |             +------Pt84982556
              |      |      +500.0-|
              |      +500.0-|      +------Pp7183 23
       +500.0-|             |
       |      |             +-------------pg 8 129 6
       |      |
       |      |                    +------Os01g74650
+------|      |                    |
|      |      +--------------272.5-|------AT3G59760
|      |                           |
|      |                           +------AT2G43750
|      |
|      +----------------------------------Pt46782548
|
+-----------------------------------------pg1 21
```

Cytochrome P450 - NJ

```
                                                                                    +------AT3G48310
                                                                             +450.0-|
                                                                      +499.0-|      +------AT3G48320
                                                                      |      |
                                                               +500.0-|      +-------------AT3G48290
                                                               |      |
                                                        +500.0-|      +--------------------AT3G48270
                                                        |      |
                                                 +344.0-|      +---------------------------AT3G48280
                                                 |      |
                                                 |      +----------------------------------AT3G26310
                                                 |
                                                 |                                  +------Pt85582551
                                                 |                           +494.0-|
                                                 |                    +259.0-|      +------pg3 57put
                                                 |                    |      |
                                          +271.0-|             +449.0-|      +-------------Os05g41440
                                          |      |             |      |
                                          |      +-------498.0-|      +--------------------AT2G40890
                                          |      |             |
                                          |      |             |                    +------AT1G74540
                                          |      |             +--------------496.0-|
                                          |      |                                  +------AT1G74550
                                          |      |
                                          |      |                                  +------Pt67730 35
                                          |      +----------------------------477.0-|
                                          |                                         +------pg9 124
                                          |
                                          |                           +--------------------Os09g26980
                                   +367.0-|                           |
                                   |      |                           |             +------Os08g35510
                                   |      +---------------------499.0-|             |
                                   |      |                           |      +296.0-|------Os09g08990
                                   |      |                           |      |      |
                                   |      |                           +314.0-|      +------Os09g26960
                                   |      |                                  |
                                   |      |                                  +-------------Os09g26940
                                   |      |
                                   |      |                                         +------AT4G22690
                            +498.0-|      |                                  +500.0-|
                            |      |      |                           +393.0-|      +------AT4G22710
                            |      |      |                           |      |
                            |      |      |                           |      +-------------Os06g41070
                            |      |      +---------------------259.0-|
                            |      |                                  |             +------Pt49640 23
                     +320.0-|      |                                  |      +483.0-|
                     |      |      |                                  +498.0-|      +------Pt58482554
                     |      |      |                                         |
                     |      |      |                                         +-------------AT5G07990
              +459.0-|      |      |
              |      |      |      +-------------------------------------------------------Os08g03682
              |      |      |
              |      |      +--------------------------------------------------------------Os01g08810
              |      |
              |      +---------------------------------------------------------------------AT2G27690
              |
       +360.0-|----------------------------------------------------------------------------Os05g31740
       |      |
       |      |                                                                     +------AT1G34540
       |      |                                                              +373.0-|
       |      +--------------------------------------------------------370.0-|      +------AT3G56630
+------|      |                                                              |
|      |      |                                                              +-------------Os01g58990
|      |      |
|      |      +----------------------------------------------------------------------------Os05g37250
|      |
|      |                                                                            +------AT5G63450
|      +----------------------------------------------------------------------277.0-|
|                                                                                   +------AT3G48520
|
+------------------------------------------------------------------------------------------Os12g25660
```

Cytochrome P450 - PARS

```
                                          +----------------------------------Os06g41070
                                          |
                                          |                           +------AT4G22690
                                          |      +--------------500.0-|
                                          |      |                    +------AT4G22710
                                          |      |
                                          |      |                    +------Os09g08990
                                          |      |                    |
                                          |      |                    +------Os09g26940
                                          |      |                    |
                                          |      +--------------489.4-|------Os08g35510
                                   +257.5-|      |                    |
                                   |      |      |                    +------Os09g26980
                                   |      |      |                    |
                                   |      |      |                    +------Os09g26960
                                   |      |      |
                                   |      |      |             +-------------Os05g41440
                                   |      |      |      +370.9-|
                                   |      |      |      |      |      +------Pt85582551
                                   |      |      |      |      +499.2-|
                                   |      |      +499.5-|             +------pg3 57put
                                   |      |      |      |
                                   |      +307.3-|      |             +------AT1G74550
                                   |             |      |      +498.0-|
                                   |             |      +305.4-|      +------AT1G74540
                                   |             |             |
                                   |             |             +-------------AT2G40890
                            +264.3-|             |
                            |      |             |                    +------Pt67730 35
                            |      |             +--------------337.1-|
                            |      |             |                    +------pg9 124
                            |      |             |
                            |      |             |             +-------------AT3G48320
                            |      |             |             |
                            |      |             |      +445.0-|      +------AT3G48270
                            |      |             |      |      |      |
                     +500.0-|      |             |      |      +335.4-|------AT3G48310
                     |      |      |             +496.0-|             |
                     |      |      |             |      |             +------AT3G48290
                     |      |      |             |      |
                     |      |      |             |      +--------------------AT3G48280
                     |      |      |             |
                     |      |      |             +---------------------------AT3G26310
                     |      |      |
                     |      |      +-----------------------------------------AT5G07990
                     |      |
                     |      +------------------------------------------------Os08g03682
                     |
              +318.7-|                                                +------AT1G34540
              |      |                                         +500.0-|
              |      +-----------------------------------268.0-|      +------AT3G56630
              |      |                                         |
              |      |                                         +-------------Os01g58990
              |      |
              |      |                                                +------Os05g37250
       +492.0-|      +------------------------------------------500.0-|
       |      |      |                                                +------AT2G27690
       |      |      |
       |      |      +-------------------------------------------------------Os05g31740
+------|      |      |
|      |      |      +-------------------------------------------------------Os01g08810
|      |      |
|      |      +--------------------------------------------------------------Os12g25660
|      |
|      +---------------------------------------------------------------------AT5G63450
|
+----------------------------------------------------------------------------AT3G48520
```

Dehydration protein - NJ

```
                                                        +------Pt14135 7
              +-----------------------------------478.0-|
              |                                         +------Pt37059 29
              |
              |                                         +------Os04g14150
              |                                  +337.0-|
              |                           +491.0-|      +------AT4G19120
              |                           |      |
              |                    +484.0-|      +-------------AT1G31850
              |                    |      |
              |                    |      |      +-------------Pp8253 14
              |                    |      +493.0-|
              |             +482.0-|             |      +------pg6 144 7
              |             |      |             +345.0-|
              |             |      |                    +------Pt9929 31
              |      +414.0-|      |
       +251.0-|      |      |      +---------------------------Os06g03750
       |      +454.0-|      |
       |      |      |      +----------------------------------Os09g24900
       |      |      |
       |      |      +-----------------------------------------Os10g41970
       |      |
       |      +------------------------------------------------AT3G51070
       |      |
       |      |                                  +-------------AT2G34300
+------|      +----------------------------322.0-|
|      |      |                                  |      +------Os01g61250
|      |      |                                  +490.0-|
|      |      |                                         +------pg11 128 1
|      |      |
|      |      +------------------------------------------------Os11g08314
|      |      |
|      |      +------------------------------------------------AT1G29470
|      |
|      +-------------------------------------------------------Os01g66110
|
+--------------------------------------------------------------Os12g07840
```

Dehydration protein - PARS

```
              +----------------------------------Os10g41970
              |
              |      +--------------------------Os09g24900
       +435.7-|      |
       |      |      |                    +------Os04g14150
       |      |      |                    |
       |      |      |             +490.5-|------AT4G19120
       |      +317.3-|             |      |
       |             |             |      +------AT1G31850
       |             |      +496.9-|
       |             |      |      |      +------Pt9929 31
       |             |      |      |      |
       |             +489.6-|      +493.2-|------Pp8253 14
       |                    |             |
       |                    |             +------pg6 144 7
       |                    |
       |                    +--------------------Os06g03750
       |
+------|-----------------------------------------Os11g08314
|      |
|      +-----------------------------------------AT3G51070
|      |
|      +-----------------------------------------Pt14135 7
|      |
|      |                                  +------Os01g61250
|      |                           +497.8-|
|      +---------------------486.5-|      +------pg11 128 1
|      |                           |
|      |                           +-------------AT2G34300
|      |
|      +-----------------------------------------AT1G29470
|      |
|      +-----------------------------------------Os01g66110
|      |
|      +-----------------------------------------Pt37059 29
|
+------------------------------------------------Os12g07840
```

Dihydroflavonol 4-reductase - NJ

```
                                                        +------Pt19582551
                                                 +500.0-|
                                                 |      +------Pt18965 25
                                          +353.0-|
                                          |      |      +------Pt76182553
                                          |      +494.0-|
                            +-------304.0-|             +------Pp11556 25
                            |             |
                            |             |             +------Os01g44260
                            |             |      +322.0-|
                            |             +254.0-|      +------AT5G42800
                            |                    |
                            |                    +-------------pg 6 37
                            |
                            |                           +------Os04g53850
                            |                           |
                            |                           +------Os04g53780
                     +307.0-|                           |
                     |      |      +--------------337.0-|------Os04g53920
                     |      |      |                    |
                     |      |      |                    +------Os04g53810
                     |      |      |                    |
                     |      |      |                    +------Os04g53800
                     |      |      |
                     |      |      +---------------------------pg 9 126 2
                     |      |      |
                     |      |      |                    +------Pp8488 21
                     |      |      |             +349.0-|
              +365.0-|      +278.0-|             |      +------Pt37855 17
              |      |             |      +312.0-|
              |      |             |      |      +-------------Pt1783 16
              |      |      +266.0-|      |
              |      |             |      |      +-------------Pt66392 20
              |      |             |      |
              |      |             |      +--------------------Pt72342 21
       +500.0-|      |             |
       |      |      |             +---------------------------AT4G27250
       |      |      |             |
       |      |      |             +---------------------------AT1G61720
+------|      |      |
|      |      |      +-----------------------------------------AT2G45400
|      |      |
|      |      +------------------------------------------------AT4G35420
|      |
|      +-------------------------------------------------------AT1G51410
|
+--------------------------------------------------------------AT1G15950
```

Dihydroflavonol 4-reductase - PARS

```
                            +------AT1G51410
              +-------500.0-|
              |             +------AT1G15950
              |
              |      +-------------AT4G27250
              |      |
              |      +-------------Pt72342 21
              |      |
              |      +-------------AT1G61720
              |      |
              |      |      +------Os04g53800
              |      +252.6-|
              |      |      +------Os04g53780
              |      |
              |      +-------------Pt37855 17
              +250.5-|
              |      +-------------pg 9 126 2
              |      |
              |      |      +------Os04g53920
              |      +297.0-|
              |      |      +------Os04g53850
       +359.7-|      |
       |      |      +-------------Os04g53810
       |      |      |
       |      |      +-------------Pp8488 21
       |      |      |
       |      |      |      +------Pt1783 16
       |      |      +330.2-|
       |      |             +------Pt66392 20
       |      |
       |      +--------------------pg 6 37
       |      |
+------|      |             +------Pt18965 25
|      |      +-------398.9-|
|      |      |             +------Pt19582551
|      |      |
|      |      +--------------------AT4G35420
|      |      |
|      |      |             +------Pt76182553
|      |      +-------499.0-|
|      |      |             +------Pp11556 25
|      |      |
|      |      +--------------------AT2G45400
|      |
|      +---------------------------AT5G42800
|
+----------------------------------Os01g44260
```

DOF family - NJ

```
                                          +--------------------pg4 105
                                          |
              +---------------------471.0-|             +------Pt38028 21
              |                           |      +497.0-|
              |                           +314.0-|      +------Pt38059 5
              |                                  |
              |                                  +-------------Pp11615 12
              |
              |                                         +------AT2G37590
              |                                  +490.0-|
              |                           +284.0-|      +------AT5G02460
              |                           |      |
              |                    +314.0-|      +-------------Os03g16850
              |                    |      |
              |                    |      |             +------AT4G38000
       +500.0-|                    |      +-------500.0-|
       |      |                    |                    +------AT5G60200
       |      |             +452.0-|
       |      |             |      |                    +------AT3G21270
       |      |             |      |             +338.0-|
       |      |             |      |      +483.0-|      +------Os03g38870
       |      |             |      |      |      |
       |      |             |      +307.0-|      +-------------AT1G28310
       |      |      +366.0-|             |
       |      |      |      |             +--------------------Pt58151 20
+------|      |      |      |
|      |      |      |      |                           +------Pp713516 2
|      |      |      |      +---------------------500.0-|
|      |      +297.0-|      |                           +------Pt49007 32
|      |             |      |
|      |             |      +----------------------------------pg3 114
|      |             |
|      |             |                                  +------AT4G24060
|      |             +----------------------------500.0-|
|      |                                                +------Os04g47990
|      |
|      +-------------------------------------------------------AT5G62940
|
+--------------------------------------------------------------Os02g47810
```

DOF family - PARS

```
                                          +------Pt38059 5
                                   +447.4-|
                                   |      +------Pt38028 21
              +--------------450.0-|
              |                    +-------------pg4 105
              |                    |
              |                    +-------------Pp11615 12
              |
              |                           +------Pt49007 32
              |             +-------460.1-|
              |             |             +------Pp713516 2
              |             |
       +500.0-|             |      +-------------Pt58151 20
       |      |             |      |
       |      |             +401.6-|      +------Os03g38870
       |      |             |      |      |
       |      |      +293.2-|      +342.7-|------AT1G28310
       |      |      |      |             |
       |      |      |      |             +------AT4G38000
       |      |      |      |
       |      |      |      |             +------AT5G02460
+------|      |      |      |      +426.3-|
|      |      +344.7-|      |      |      +------Os03g16850
|      |             |      +289.0-|
|      |             |             |      +------Os04g47990
|      |             |             +500.0-|
|      |             |                    +------AT4G24060
|      |             |
|      |             +---------------------------pg3 114
|      |
|      +-----------------------------------------AT5G62940
|
+------------------------------------------------Os02g47810
```

Dormancy/auxin associated family - NJ

```
+-------------------------------------------------------pg5 122
|
|                           +---------------------------Pt58782551
|                           |
|                           |                    +------AT1G56220
|                    +169.0-|             +361.0-|
|                    |      |      +210.0-|      +------Pt24937 12
|                    |      |      |      |
|             +317.0-|      +210.0-|      +-------------Pp5213517
|             |      |             |
|             |      |             +--------------------Pt70044 27
|             |      |
|             |      +----------------------------------Pt69533 11
|             |
|             |                                  +------pg5 121
|      +373.0-|                           +119.0-|
|      |      |                    +-46.0-|      +------Pt73256 14
|      |      |                    |      |
|      |      |             +-42.0-|      +-------------Pt73253 13
|      |      |             |      |
|      |      |      +-82.0-|      +--------------------Pt73251 12
|      |      |      |      |
+------|      +369.0-|      +---------------------------Pt73255 12
       |             |
       |             +----------------------------------Pt73252 13
       |
       |                                  +-------------Pt30182555
       |                           +401.0-|
       |                           |      |      +------pg5 115put
       |                           |      +421.0-|
       +---------------------368.0-|             +------Pp7314 11
                                   |
                                   |             +------AT1G54070
                                   +-------334.0-|
                                                 +------AT1G28330
```

Dormancy/auxin associated family - PARS

```
                                   +---------------------------Pt70044 27
                                   |
                                   |                    +------Pt73256 14
                            +311.7-|             +261.7-|
                            |      |      +425.5-|      +------Pt73253 13
                            |      |      |      |
                     +328.4-|      +325.6-|      +-------------pg5 122 18
                     |      |             |
                     |      |             +--------------------pg5 121 16
              +303.5-|      |
              |      |      +----------------------------------Pt24937 12
              |      |
       +499.1-|      +-----------------------------------------pg5 115 13
       |      |
       |      |                                         +------AT1G56220
+------|      +-----------------------------------473.5-|
|      |                                                +------AT1G54070
|      |
|      +-------------------------------------------------------Pt17479 11
|
+--------------------------------------------------------------Pp4768 13
```

Embryo-abundant protein-related - NJ

```
                                                 +------Pt19460 18
                                          +132.0-|
                                   +270.0-|      +------Pt95382549
                                   |      |
                            +500.0-|      +-------------pg2 168put
                            |      |
                     +293.0-|      +--------------------Pt35976 16
                     |      |
                     |      |                    +------Pt39882556
              +319.0-|      +--------------500.0-|
              |      |                           +------pg4 11put
              |      |
              |      +----------------------------------AT2G41380
              |
              |                                  +------AT1G55450
              |                           +444.0-|
       +500.0-|                    +500.0-|      +------AT3G54150
       |      |                    |      |
       |      |             +284.0-|      +-------------AT3G61210
       |      |             |      |
       |      |             |      |             +------AT4G22530
       |      |      +154.0-|      +-------460.0-|
+------|      |      |      |                    +------AT5G10830
|      |      +279.0-|      |
|      |             |      +---------------------------Os01g40660
|      |             |
|      |             +----------------------------------Os01g51870
|      |
|      +------------------------------------------------Pt73067 15
|
+-------------------------------------------------------Pt74719 16
```

Embryo-abundant protein-related - PARS

```
+------------------------------------------------Pt35976 16
 |
 |                                         +------Pt39882556
 |                    +--------------500.0-|
 |                    |                    +------pg4 11put
 |                    |
 |                    |      +--------------------Os01g40660
 |                    |      |
 |                    |      |      +-------------AT3G54150
 |                    |      +497.0-|
 |                    +296.4-|      |      +------AT3G61210
 |                    |      |      +329.3-|
 |             +287.3-|      |             +------AT1G55450
 |             |      |      |
 |             |      |      |      +-------------Os01g51870
 |             |      |      +251.5-|
 |             |      |             |      +------AT5G10830
 |             |      |             +253.3-|
 |      +491.5-|      |                    +------AT4G22530
 |      |      |      |
 |      |      |      |                    +------Pt73067 15
 |      |      |      +--------------497.5-|
 +------|      |                           +------Pt74719 16
        |      |
        |      +----------------------------------AT2G41380
        |
        |                           +-------------Pt95382549
        +---------------------366.6-|
                                    |      +------pg2 168put
                                    +314.7-|
                                           +------Pt19460 18
```

Endo-1,4-beta-glucanase KORRIGAN - NJ

```
                                                                                    +------AT2G32990
                                                                      +-------500.0-|
                                                                      |             +------Os02g50490
                                                               +352.0-|
                                                               |      |             +------Os01g12030
                                                               |      |      +500.0-|
                                                               |      +492.0-|      +------Os01g12070
                                                        +351.0-|             |
                                                        |      |             +-------------Os05g12150
                                                        |      |
                                                 +480.0-|      |                    +------AT1G64390
                                                 |      |      +--------------496.0-|
                                                 |      |                           +------AT4G11050
                                                 |      |
                                          +494.0-|      +----------------------------------Pt9380 32
                                          |      |
                                          |      |                                  +------Os04g57860
                                          |      |                           +322.0-|
                                   +446.0-|      +---------------------304.0-|      +------AT1G48930
                                   |      |                                  |
                                   |      |                                  +-------------AT2G44550
                                   |      |
                                   |      |                                         +------AT4G23560
                                   |      +-----------------------------------500.0-|
                            +362.0-|                                                +------AT4G09740
                            |      |
                            |      |                                                +------AT1G19940
                            |      |                                         +480.0-|
                            |      |                                  +500.0-|      +------AT1G75680
                            |      |                                  |      |
                            |      +----------------------------492.0-|      +-------------Os06g50140
                            |                                         |
                            |                                         +--------------------Pt53582556
                            |
                            |                                                       +------Os08g02220
                            |                                  +--------------300.0-|
                     +500.0-|                                  |                    +------Os09g36060
                     |      |                                  |
                     |      |                           +309.0-|                    +------Pt91982554
                     |      |                           |      |             +500.0-|
                     |      |                           |      |      +500.0-|      +------Pp2910 22
                     |      |                           |      |      |      |
                     |      |                           |      +308.0-|      +-------------pg8 85
                     |      |                           |             |
                     |      |                           |             +--------------------AT1G71380
                     |      |                           |
                     |      |                           +----------------------------------Os01g21070
                     |      |                           |
                     |      +---------------------472.0-|                           +------AT1G70710
              +478.0-|                                  +---------------------500.0-|
              |      |                                  |                           +------AT1G23210
              |      |                                  |
              |      |                                  +----------------------------------Pt11267 63
              |      |                                  |
              |      |                                  |                           +------AT1G02800
              |      |                                  +---------------------497.0-|
              |      |                                  |                           +------AT4G02290
              |      |                                  |
              |      |                                  |                           +------Os02g50040
       +421.0-|      |                                  +---------------------500.0-|
       |      |      |                                                              +------Os06g14540
       |      |      |
       |      |      |                                                              +------AT1G65610
       |      |      +--------------------------------------------------------497.0-|
       |      |                                                                     +------Os04g41970
       |      |
       |      |                                                +---------------------------Os03g21210
+------|      |                                                |
|      |      |                                                |                    +------Pt40328 51
|      |      +------------------------------------------367.0-|             +500.0-|
|      |                                                       |      +494.0-|      +------Pp6413523
|      |                                                       |      |      |
|      |                                                       +271.0-|      +-------------pg10 71
|      |                                                              |
|      |                                                              +--------------------Os03g52630
|      |
|      +-----------------------------------------------------------------------------------AT5G49720
|
+------------------------------------------------------------------------------------------AT4G24260
```

Endo-1,4-beta-glucanase KORRIGAN - PARS

```
                                   +------Pt9380 32
                            +499.3-|
                            |      +------Pp12338 21
                            |
                            +-------------Os03g52630
                            |
                            |      +------pg3 26put
                            +500.0-|
                            |      +------Pt47947 17
                            |
                            +-------------AT2G32990
                            |
                            +-------------Os06g50140
                            |
                            |      +------pg8 85
                            |      |
                            +328.3-|------Pt91982554
                            |      |
                            |      +------Pp2910 22
                            |
                            +-------------Os02g50040
                            |
                            +-------------Pt53582556
                            |
                            +-------------AT5G49720
                            |
                            |      +------Os01g12030
                            +285.8-|
                            |      +------Os02g50490
                            |
                            +-------------Os04g41970
                            |
                            +-------------Os01g12070
                            |
                            |      +------AT4G02290
                            +393.5-|
                            |      +------AT1G02800
                            |
                            +-------------AT4G24260
       +--------------294.7-|
       |                    +-------------AT1G64390
       |                    |
       |                    |      +------AT4G09740
       |                    +311.6-|
       |                    |      +------AT4G23560
       |                    |
       |                    |      +------AT1G75680
       |                    +394.6-|
       |                    |      +------AT1G48930
       |                    |
       |                    +-------------Os05g12150
       |                    |
       |                    +-------------Pt11267 63
       |                    |
       |                    +-------------Os01g21070
       |                    |
       |                    +-------------Os06g14540
       |                    |
       |                    +-------------Os04g57860
       |                    |
       |                    +-------------Os08g02220
       |                    |
+------|                    +-------------AT1G23210
|      |                    |
|      |                    +-------------AT1G71380
|      |                    |
|      |                    +-------------AT2G44550
|      |                    |
|      |                    +-------------Os09g36060
|      |                    |
|      |                    +-------------AT1G65610
|      |                    |
|      |                    +-------------AT1G19940
|      |                    |
|      |                    +-------------AT4G11050
|      |                    |
|      |                    +-------------AT1G70710
|      |                    |
|      |                    +-------------Pt39488 12
|      |
|      |                           +------pg10 71
|      |                    +250.3-|
|      |             +422.1-|      +------Pt40328 51
|      |             |      |
|      |      +427.0-|      +-------------Pt40419 9
|      |      |      |
|      +374.4-|      +--------------------Pp6413523
|             |
|             |                    +------pg8 172
|             +--------------418.1-|
|                                  +------Pt26100 33
|
+-----------------------------------------Os03g21210
```

Endomembrane protein - NJ

```
                     +---------------------------Os05g07650
                     |
              +252.0-|                    +------pg4 125
              |      |      +-------500.0-|
              |      |      |             +------Pt80582549
              |      +500.0-|
              |             |             +------AT3G13772
       +303.0-|             |      +500.0-|
       |      |             +498.0-|      +------AT1G55130
       |      |                    |
       |      |                    +-------------AT5G25100
       |      |
+------|      |                           +------Os02g49050
|      |      +---------------------497.0-|
|      |                                  +------AT4G12650
|      |
|      |                                  +------Os02g55440
|      |                           +405.0-|
|      +---------------------475.0-|      +------pg12 25
|                                  |
|                                  +-------------AT5G35160
|
+------------------------------------------------Pt44629 19
```

Endomembrane protein - PARS

```
                                                        +------AT1G55130
                                                 +500.0-|
                                          +499.0-|      +------AT3G13772
                                          |      |
                                   +500.0-|      +-------------AT5G25100
                                   |      |
                                   |      |             +------Pt80582549
                            +424.5-|      +-------500.0-|
                            |      |                    +------pg4 125
                            |      |
                     +482.0-|      +---------------------------Os05g07650
                     |      |
                     |      |                           +------Os02g49050
              +500.0-|      +---------------------345.8-|
              |      |                                  +------AT4G12650
       +369.6-|      |
       |      |      +-----------------------------------------Pt44629 19
+------|      |
|      |      +------------------------------------------------AT5G35160
|      |
|      +-------------------------------------------------------Os02g55440
|
+--------------------------------------------------------------pg12 25
```

Epimerase/dehydratase - NJ

```
                                                               +------Os01g21320
                     +-----------------------------------278.0-|
                     |                                         +------Os01g62020
                     |
                     |             +----------------------------------AT3G23820
                     |             |
                     |             |                           +------Os09g32670
                     |             |                    +500.0-|
                     |             |             +400.0-|      +------Os08g41440
                     |             |             |      |
                     |      +500.0-|      +340.0-|      +-------------Os03g14540
                     |      |      |      |      |
                     |      |      |      |      |             +------Os06g08810
                     |      |      |      |      +-------383.0-|
                     |      |      |      |                    +------Os02g54890
                     |      |      |      |
                     |      |      |      |                    +------Pt21817 33
                     |      |      +367.0-|             +500.0-|
                     |      |             |             |      +------pg8 169
                     |      |             |      +297.0-|
                     |      |             |      |      |      +------Pt69882556
                     |      |             |      |      +492.0-|
                     |      |             |      |             +------pg2 145
                     |      |             |      |
                     |      |             +312.0-|--------------------AT4G30440
                     +500.0-|                    |
                     |      |                    |             +------AT1G02000
              +493.0-|      |                    |             |
              |      |      |                    |      +262.0-|------AT4G12250
              |      |      |                    |      |      |
              |      |      |                    +284.0-|      +------AT4G00110
              |      |      |                           |
              |      |      |                           +-------------AT2G45310
              |      |      |
              |      |      |                                  +------Pt72982549
              |      |      |             +--------------411.0-|
              |      |      |             |                    +------Pp5713516
              |      |      |             |
              |      |      |             |                    +------pg2 154put
              |      |      |             |             +489.0-|
              |      |      +-------500.0-|      +254.0-|      +------Pt57848 21
              |      |                    |      |      |
              |      |                    |      |      +-------------AT1G63000
              |      |                    |      |
              |      |                    |      |             +------AT3G14790
              |      |                    |      +-------454.0-|
              |      |                    +488.0-|             +------AT1G53500
       +269.0-|      |                           |
       |      |      |                           +--------------------Os02g45540
       |      |      |                           |
       |      |      |                           +--------------------Os03g17000
       |      |      |                           |
       |      |      |                           +--------------------AT1G78570
       |      |      |
       |      |      +------------------------------------------------Pp5413519
       |      |
       |      |                                                +------Pt87382554
       |      |                                         +473.0-|
+------|      |                                         |      +------pg7 100
|      |      +-----------------------------------254.0-|
|      |      |                                         |      +------Pp7838 28
|      |      |                                         |      |
|      |      |                                         +479.0-|------pg1 124
|      |      |                                                |
|      |      |                                                +------Pt11818255
|      |      |
|      |      +-------------------------------------------------------AT3G46440
|      |      |
|      |      +-------------------------------------------------------AT5G59290
|      |
|      +--------------------------------------------------------------AT2G28760
|
+---------------------------------------------------------------------Os03g16980
```

Epimerase/dehydratase - PARS

```
                                                        +------Os01g62020
                                                 +402.2-|
                     +---------------------397.6-|      +------Os01g21320
                     |                           |
                     |                           +-------------Pp5413519
                     |
                     |                    +--------------------AT2G45310
                     |                    |
                     |                    |             +------pg2 145
                     |                    |      +499.9-|
                     |                    |      |      +------Pt69882556
                     |                    +265.5-|
                     |                    |      |      +------pg8 169
                     |                    |      +499.9-|
                     |                    |             +------Pt21817 33
                     |                    |
                     |             +284.6-|--------------------AT4G30440
                     |             |      |
                     |             |      +--------------------AT1G02000
              +499.9-|             |      |
              |      |             |      |             +------Os06g08810
              |      |             |      +-------435.8-|
              |      |             |      |             +------Os02g54890
              |      |             |      |
              |      |             |      +--------------------AT4G12250
              |      |      +499.9-|      |
              |      |      |      |      +--------------------AT4G00110
              |      |      |      |
              |      |      |      |                    +------Os08g41440
              |      |      |      +--------------499.9-|
              |      |      |      |                    +------Os09g32670
              |      |      |      |
              |      |      |      +---------------------------Os03g14540
              |      |      |      |
              |      |      |      +---------------------------AT3G23820
              |      |      |
              |      +499.9-|             +--------------------Os03g17000
              |             |             |
              |             |             |      +-------------AT1G63000
              |             |             +254.0-|
              |             |             |      |      +------pg2 154put
              |             |             |      +497.3-|
              |             |             |             +------Pt57848 21
       +298.2-|             |      +294.4-|
       |      |             |      |      |             +------AT3G14790
       |      |             |      |      |      +468.8-|
       |      |             |      |      +299.8-|      +------AT1G53500
       |      |             |      |      |      |
       |      |             +499.9-|      |      +-------------AT1G78570
       |      |                    |      |
       |      |                    |      +--------------------Os02g45540
       |      |                    |
       |      |                    |                    +------Pp5713516
       |      |                    +--------------472.2-|
       |      |                                         +------Pt72982549
       |      |
+------|      +------------------------------------------------AT5G59290
|      |      |
|      |      |                                         +------Pp7838 28
|      |      |                                  +288.8-|
|      |      |                           +497.0-|      +------pg1 124
|      |      |                           |      |
|      |      |                           |      +-------------Pt11818255
|      |      +---------------------413.0-|
|      |      |                           +--------------------pg7 100
|      |      |                           |
|      |      |                           +--------------------Pt87382554
|      |      |
|      |      +------------------------------------------------AT3G46440
|      |
|      +-------------------------------------------------------AT2G28760
|
+--------------------------------------------------------------Os03g16980
```

Exostosin - NJ

```
                                                                                                                       +------Os03g05070
                                                        +-------------------------------------------------------216.0-|
                                                        |                                                             +------Os10g32110
                                                        |
                                                        |                                                             +------AT2G35100
                                                        |                                                      +380.0-|
                                                        |                                               +431.0-|      +------AT5G44930
                                                        |                                               |      |
                                                        |                                               |      +-------------Os07g37960
                                                        |                                        +212.0-|
                                                        |                                        |      |             +------Os01g69220
                                                        |                                        |      |      +443.0-|
                                                        |                                        |      +246.0-|      +------AT1G67410
                                                        |             +---------------------197.0-|             |
                                                        |             |                          |             +-------------Pt29295 11
                                                        |             |                          |
                                                 +111.0-|             |                          |                    +------AT1G34270
                                                 |      |             |                          |             +137.0-|
                                                 |      |             |                          +-------215.0-|      +------pg 1 71 10
                                                 |      |             |                                        |
                                                 |      |             |                                        +-------------Os04g57510
                                                 |      |             |
                                                 |      |      +500.0-|             +-----------------------------------------AT3G07620
                                                 |      |      |      |             |
                                                 |      |      |      |             |                             +------Pt6816 40
                                                 |      |      |      |             |                      +500.0-|
                                                 |      |      |      |             |               +403.0-|      +------Os01g01780
                                                 |      |      |      |      +160.0-|                      |      |
                                                 |      |      |      |      |      |        +298.0-|      +-------------pg5 70
                                                 |      |      |      |      |      |        |      |
                                                 |      |      |      |      |      +349.0-|      +--------------------AT5G03795
                                          +138.0-|      |      |      |      |      |      |
                                          |      +447.0-|      +500.0-|      +137.0-|      +---------------------------AT5G25310
                                          |      |      |      |      |      |      |
                                          |      |      |      |      |      |      +----------------------------------Os07g09050
                                          |      |      |      |      |      |
                                          |      |      |      |      |      |                                          +------Os06g07887
                                          |      |      |      |      |      |                                   +404.0-|
                                          |      |      |      |      |      +---------------------------167.0-|      +------Os04g01930
                                          |      |      |      |      |                                        |
                                   +-56.0-|      |      |      |      |                                        +-------------AT5G20260
                                   |      |      |      |      |
                                   |      |      |      |      |                                                      +------Pt29640 36
                                   |      |      |      |      +------------------------------------------------453.0-|
                                   |      |      |      |                                                             +------AT4G22580
                                   |      |      |      |
                                   |      |      |      +----------------------------------------------------------------------------AT2G32740
                            +208.0-|      |      |
                            |      |      |      |                                                                    +------AT2G32750
                            |      |      |      +---------------------------------------------------------------------136.0-|
                            |      |      |                                                                           +------AT2G29040
                            |      |      |
                     +177.0-|      |      |                                                                           +------Pt67332 23
                     |      |      |      |                                                                    +249.0-|
                     |      |      +---------------------------------------------------------------------307.0-|      +------AT5G62220
                     |      |                                                                             |
              +440.0-|      |                                                                             +-------------AT1G63450
              |      |      |
              |      |      +-------------------------------------------------------------------------------------------------Os03g05060
       +483.0-|      |
       |      |      +--------------------------------------------------------------------------------------------------------Os10g32170
+------|      |
|      |      +---------------------------------------------------------------------------------------------------------------Pt76618 21
|      |
|      +----------------------------------------------------------------------------------------------------------------------AT2G20370
|
+-----------------------------------------------------------------------------------------------------------------------------Os03g05110
```

Exostosin - PARS

```
+--------------------------------------------------------------Os03g05110
|
|             +------------------------------------------------Os03g05070
|             |
|             |                                         +------AT5G44930
|             |                                  +427.0-|
|             |                           +459.3-|      +------AT2G35100
|             |                           |      |
|             |                           |      +-------------Os07g37960
|             |                           |
|             |                    +422.7-|             +------AT1G67410
|             |                    |      +-------469.6-|
|             |                    |      |             +------Os01g69220
|             |                    |      |
|             |                    |      +--------------------Pt29295 11
|             |             +421.9-|
|             |             |      +---------------------------Os04g57510
|             |             |      |
|             |             |      +---------------------------AT1G34270
|             |             |      |
|             |             |      +---------------------------pg 1 71 10
|             |             |
|             |             |                    +-------------pg5 70
|             |             |                    |
|             |      +440.4-|                    +-------------Os07g09050
|             |      |      |                    |
|             |      |      |                    |      +------Os04g01930
|             |      |      |                    +445.3-|
|             |      |      |                    |      +------Os06g07887
|             |      |      |             +288.7-|
|             |      |      |             |      +-------------AT3G07620
|             |      |      |             |      |
|             |      |      |             |      +-------------AT5G20260
|      +308.4-|-426.7-|      |             |      |
|      |      |      |      +-------500.0-|      +-------------AT5G25310
|      |      |      |                    |      |
|      |      |      |                    |      +-------------AT5G03795
|      |      |      |                    |
|      |      |      |                    |             +------Os01g01780
|      |      |      |                    +-------500.0-|
|      |      |      |                                  +------Pt6816 40
|      |      |      |
|      |      |      |                                  +------AT4G22580
|      |      |      +----------------------------495.2-|
|      |      |                                         +------Pt29640 36
|      |      |
|      |      +------------------------------------------------Os10g32110
|      |      |
|      |      +------------------------------------------------Pt76618 21
+------|      |
       |      |                                         +------AT5G62220
       |      +-----------------------------------277.5-|
       |      |                                         +------Pt67332 23
       |      |
       |      +------------------------------------------------AT2G29040
       |      |
       |      +------------------------------------------------AT2G32750
       |      |
       |      +------------------------------------------------Os10g32170
       |      |
       |      +------------------------------------------------AT2G32740
       |      |
       |      +------------------------------------------------Os03g05060
       |      |
       |      +------------------------------------------------AT1G63450
       |
       +-------------------------------------------------------AT2G20370
```

Expansin alpha - NJ

```
                                          +------Pt11972 13
                                   +500.0-|
                                   |      +------pg3 125 42
                     +-------499.0-|
                     |             |      +------Pt71597 13
                     |             +385.0-|
                     |                    +------pg3 124 42
                     |
                     |                    +------pg11 21 33
                     |             +488.0-|
                     |             |      +------Pt55810 15
                     |      +457.0-|
                     |      |      |      +------Pt28971 11
                     |      |      +494.0-|
                     +470.0-|             +------pg11 23 12
                     |      |
                     |      |             +------AT2G03090
              +407.0-|      |             |
              |      |      +-------440.0-|------AT1G69530
              |      |                    |
              |      |                    +------AT1G26770
              |      |
              |      |                    +------AT2G40610
              |      +--------------275.0-|
              |      |                    +------AT5G05290
              |      |
              |      |                    +------Os01g60770
       +332.0-|      +--------------394.0-|
       |      |      |                    +------Os05g39990
       |      |      |
       |      |      +---------------------------Os02g51040
       |      |      |
       |      |      +---------------------------Os03g21820
       |      |
+------|      +----------------------------------Os03g60720
|      |      |
|      |      |                           +------AT2G37640
|      |      |                    +463.0-|
|      |      +--------------415.0-|      +------AT5G02260
|      |                           |
|      |                           +-------------AT2G28950
|      |
|      +-----------------------------------------AT2G39700
|
+------------------------------------------------AT3G55500
```

Expansin alpha - PARS

```
+----------------------------------AT2G03090
|
|             +--------------------Os02g51040
|             |
|      +277.0-|             +------pg3 125 42
|      |      |      +499.3-|
|      |      |      |      +------Pt11972 13
|      |      +492.4-|
|      |             |      +------Pt71597 13
|      |             +381.3-|
|      |                    +------pg3 124 42
|      |
|      |                    +------AT3G55500
|      |      +-------312.8-|
|      |      |             +------AT2G39700
|      |      |
|      +297.1-|--------------------Os03g60720
|      |      |
|      |      |      +-------------AT2G28950
|      |      +290.5-|
|      |             |      +------AT5G02260
|      |             +368.2-|
|      |                    +------AT2G37640
|      |
+------|---------------------------AT1G69530
       |
       |                    +------Pt28971 11
       |             +300.2-|
       |             |      +------pg11 23 12
       +-------493.7-|
       |             |      +------pg11 21 33
       |             +468.6-|
       |                    +------Pt55810 15
       |
       |                    +------Os03g21820
       |                    |
       +-------------275.1-|------AT5G05290
       |                    |
       |                    +------AT2G40610
       |
       +---------------------------Os01g60770
       |
       +---------------------------AT1G26770
       |
       +---------------------------Os05g39990
```

Expansin beta - NJ

```
+--------------------------------------------------------------Os02g42650
|
|                                  +---------------------------pg 4 94 14
|                                  |
|                                  |                    +------Pt5264 8
|                    +-------493.0-|             +420.0-|
|                    |             |             |      +------Pt64799 13
|                    |             |      +485.0-|
|                    |             |      |      |      +------pg 4 93 20
|                    |             +346.0-|      +493.0-|
|                    |                    |             +------pg 4 92 19
|                    |                    |
|                    |                    +--------------------Pt14322 9
|                    |
|             +432.0-|                           +-------------Os04g46650
|             |      |                    +435.0-|
|             |      |                    |      |      +------AT2G45110
|             |      |                    |      +482.0-|
|             |      |             +294.0-|             +------AT3G60570
|             |      |             |      |
|             |      |             |      |             +------Os04g46630
|      +448.0-|      |      +431.0-|      +-------434.0-|
|      |      |      |      |      |                    +------AT1G65680
|      |      |      +499.0-|      |
|      |      |             |      +---------------------------Os02g44106
+------|      |             |
       |      |             +----------------------------------Os02g44108
       |      |
       |      +------------------------------------------------AT2G20750
       |
       +-------------------------------------------------------AT4G28250
```

Expansin beta - PARS

```
                                          +------AT2G45110
                                   +487.5-|
                                   |      +------AT3G60570
                                   |
                                   |      +------AT1G65680
                                   +328.4-|
                     +-------500.0-|      +------Os04g46630
                     |             |
                     |             +-------------Os04g46650
                     |             |
                     |             +-------------Os02g44108
                     |             |
                     |             +-------------Os02g44106
              +359.5-|
              |      |                    +------pg 4 93 20
              |      |             +494.6-|
              |      |             |      +------pg 4 92 19
              |      |      +494.6-|
              |      |      |      +-------------Pt64799 13
       +362.2-|      |      |      |
       |      |      +347.2-|      +-------------Pt5264 8
       |      |             |
       |      |             +--------------------pg 4 94 14
+------|      |             |
|      |      |             +--------------------Pt14322 9
|      |      |
|      |      +----------------------------------AT2G20750
|      |
|      +-----------------------------------------AT4G28250
|
+------------------------------------------------Os02g42650
```

Expansin like - NJ

```
                                          +-------------AT4G38400
                                   +500.0-|
                                   |      |      +------AT3G45960
                                   |      +500.0-|
                            +346.0-|             +------AT3G45970
                            |      |
                            |      |             +------Os10g39640
                            |      |      +455.0-|
                     +500.0-|      +345.0-|      +------Os03g04020
                     |      |             |
                     |      |             +-------------Os07g29290
                     |      |
                     |      |             +-------------Os06g50960
              +500.0-|      +-------355.0-|
              |      |                    |      +------Pt55276 21
              |      |                    +500.0-|
       +389.0-|      |                           +------pg6 107 18
       |      |      |
       |      |      +----------------------------------Os07g31390
+------|      |
|      |      +-----------------------------------------AT4G17030
|      |
|      +------------------------------------------------pg6 105 15
|
+-------------------------------------------------------Pt67969 17
```

Expansin like - PARS

```
                                                 +------AT3G45960
                                          +500.0-|
                                   +500.0-|      +------AT3G45970
                                   |      |
                                   |      +-------------AT4G38400
                                   |
                            +410.7-|             +------Os10g39640
                            |      +-------441.2-|
                            |      |             +------Os03g04020
                            |      |
                     +407.0-|      +--------------------Os07g29290
                     |      |
                     |      |                    +------Pt55276 21
                     |      |             +500.0-|
              +500.0-|      +-------283.2-|      +------pg6 107 18
              |      |                    |
              |      |                    +-------------Os06g50960
       +498.5-|      |
       |      |      +----------------------------------Os07g31390
+------|      |
|      |      +-----------------------------------------AT4G17030
|      |
|      +------------------------------------------------Pt67969 17
|
+-------------------------------------------------------pg6 105 15
```

Fasciclin-like arabinogalactan-protein - NJ

```
                                                                                           +------Os03g03600
                                                                                    +497.0-|
                                                                                    |      +------Os04g48490
                                                                      +-------462.0-|
                                                                      |             |      +------AT2G45470
                                                                      |             +500.0-|
                                                                      |                    +------AT3G60900
                                                               +248.0-|
                                                               |      |                    +------Pt89782551
                                                               |      |             +496.0-|
                                                               |      |      +496.0-|      +------Pt89782550
                                                               |      |      |      |
                                                               |      +496.0-|      +-------------pg 4 64
                                                               |             |
                                                        +499.0-|             |             +------Pt51082554
                                                        |      |             +-------500.0-|
                                                        |      |                           +------Pp8113520
                                                        |      |
                                                        |      |                           +------Os09g07350
                                                        |      |                    +500.0-|
                                          +-------434.0-|      |             +411.0-|      +------Os08g23180
                                          |             |      |             |      |
                                          |             |      +-------424.0-|      +-------------AT5G55730
                                          |             |                    |
                                          |             |                    +--------------------AT4G12730
                                          |             |
                                          |             |                                  +------Os01g62380
                                          |             +----------------------------500.0-|
                                          |                                                +------AT3G46550
                                          |
                                          |                                         +-------------Pp7413522
                                          |                                  +500.0-|
                                   +208.0-|                                  |      |      +------Pt11575 15
                                   |      |                           +325.0-|      +494.0-|
                                   |      |                           |      |             +------Pt16182553
                                   |      |                    +498.0-|      |
                                   |      |                    |      |      +--------------------pg7 62
                                   |      |             +492.0-|      |
                                   |      |             |      |      +---------------------------Pt54615 18
                                   |      |             |      |
                                   |      |      +293.0-|      +----------------------------------AT2G04780
                                   |      |      |      |
                            +153.0-|      |      |      |                                  +------Pt63878 13
                            |      |      |      |      +----------------------------500.0-|
                            |      |      +338.0-|                                         +------pg9 2
                            |      |             |
                            |      |             |                                         +------Pt11608255
                            |      |             |                                  +498.0-|
                     +126.0-|      |             +----------------------------500.0-|      +------Pp75 19
                     |      |      |                                                |
                     |      |      |                                                +-------------pg11 102
                     |      |      |
              +490.0-|      |      +--------------------------------------------------------------Os05g07060
              |      |      |
              |      |      +---------------------------------------------------------------------Os01g06580
              |      |
              |      |                                                              +-------------Os02g20540
              |      +--------------------------------------------------------240.0-|
       +496.0-|                                                                     |      +------Os05g48890
       |      |                                                                     +500.0-|
       |      |                                                                            +------Os01g47780
       |      |
       |      |                                                                            +------AT1G03870
+------|      |                                                                     +268.0-|
|      |      +---------------------------------------------------------------410.0-|      +------AT5G44130
|      |                                                                            |
|      |                                                                            +-------------AT2G20520
|      |
|      +------------------------------------------------------------------------------------------AT5G60490
|
+-------------------------------------------------------------------------------------------------AT5G03170
```

Fasciclin-like arabinogalactan-protein - PARS

```
                                   +------Os08g23180
                            +500.0-|
                     +375.3-|      +------Os09g07350
                     |      |
              +334.1-|      +-------------AT5G55730
              |      |
              |      +--------------------AT4G12730
              |
              |                    +------Os04g48490
              |             +409.8-|
              |             |      +------Os03g03600
              +-------418.2-|
       +462.0-|             |      +------AT2G45470
       |      |             +500.0-|
       |      |                    +------AT3G60900
       |      |
       |      |                    +------Pt89782550
       |      |             +498.0-|
       |      |      +466.4-|      +------Pt89782551
       |      |      |      |
       |      +474.5-|      +-------------pg 4 64
       |             |
       |             |             +------Pp8113520
       |             +-------397.1-|
       |                           +------Pt51082554
       |
       |                    +-------------Pp7413522
       |             +500.0-|
       |             |      |      +------Pt16182553
       |             |      +493.0-|
       |      +499.8-|             +------Pt11575 15
       |      |      |
       |      |      |             +------Pt54615 18
       +403.2-|      +-------493.7-|
       |      |                    +------pg7 62
       |      |
       |      +---------------------------AT2G04780
       |
       |                    +-------------pg11 102
+------|--------------489.5-|
|      |                    |      +------Pp75 19
|      |                    +492.6-|
|      |                           +------Pt11608255
|      |
|      +----------------------------------Os01g06580
|      |
|      |                           +------pg9 2
|      +---------------------500.0-|
|      |                           +------Pt63878 13
|      |
|      |                           +------Os01g47780
|      |                    +498.0-|
|      +--------------434.8-|      +------Os05g48890
|      |                    |
|      |                    +-------------Os02g20540
|      |
|      |                    +-------------AT2G20520
|      |                    |
|      |                    |      +------AT1G03870
|      +--------------453.6-|-268.0-|
|      |                    |      +------AT5G44130
|      |                    |
|      |                    |      +------AT5G60490
|      |                    +460.0-|
|      |                           +------AT5G03170
|      |
|      |                           +------Os01g62380
|      +---------------------499.0-|
|                                  +------AT3G46550
|
+-----------------------------------------Os05g07060
```

Fructose-1,6-bisphosphatase - NJ

```
                                                  +------pg4 55
                                          +498.0-|
                                          |      +------Pt12259 28
                            +-------435.0-|
                            |             |      +------AT3G54050
                            |             +400.0-|
                            |                    +------Os03g16050
                            |
                            |      +--------------------Pt11868255
                            |      |
                            |      |      +-------------pg10 89
                            |      |      |
                            |      |      +-------------AT2G01140
                            +500.0-|      |
                            |      |      +-------------Os01g02880
                            |      |      |
                     +493.0-|      |      +-------------Os11g07020
                     |      |      |      |
                     |      |      +500.0-|-------------AT2G21330
                     |      |             |
                     |      |             |      +------Pt10718254
                     |      |             +252.0-|
                     |      |             |      +------Pp6410 24
                     |      |             |
              +292.0-|      |             +-------------AT4G38970
              |      |      |             |
              |      |      |             +-------------Pt53482556
              |      |      |
              |      |      +---------------------------AT5G64380
              |      |      |
              |      |      +---------------------------AT3G55800
       +486.0-|      |
       |      |      +----------------------------------Os05g36270
       |      |
       |      +-----------------------------------------AT1G43670
+------|      |
|      |      +-----------------------------------------Pt54379 29
|      |      |
|      |      +-----------------------------------------Os01g64660
|      |
|      +------------------------------------------------Pt44177 62
|
+-------------------------------------------------------pg10 14
```

Fructose-1,6-bisphosphatase - PARS

```
                                                                      +------Os01g02880
                                                               +309.3-|
                                                               |      +------AT2G01140
                                                               |
                                                               +-------------Os11g07020
                                                               |
                                                               |      +------AT4G38970
                                                               +352.5-|
                                                        +500.0-|      +------AT2G21330
                                                        |      |
                                                        |      +-------------pg10 89
                                                        |      |
                                                        |      |      +------Pp6410 24
                                                 +500.0-|      +490.8-|
                                                 |      |      |      +------Pt10718254
                                                 |      |      |
                                          +412.1-|      |      +-------------Pt53482556
                                          |      |      |
                                          |      |      +--------------------Pt11868255
                                   +492.1-|      |
                                   |      |      +---------------------------AT3G55800
                                   |      |
                                   |      +----------------------------------AT5G64380
                            +500.0-|
                            |      |                                  +------AT3G54050
                            |      |                           +311.6-|
                            |      |                           |      +------Os03g16050
                     +352.1-|      +---------------------499.3-|
                     |      |                                  |      +------Pt12259 28
                     |      |                                  +460.6-|
              +280.2-|      |                                         +------pg4 55
              |      |      |
              |      |      +------------------------------------------------Pt54379 29
       +370.2-|      |
       |      |      +-------------------------------------------------------AT1G43670
       |      |
+------|      |                                                       +------pg10 14
|      |      +-------------------------------------------------347.0-|
|      |                                                              +------Pt44177 62
|      |
|      +---------------------------------------------------------------------Os01g64660
|
+----------------------------------------------------------------------------Os05g36270
```

Galactosidase beta - NJ

```
                                                                      +------AT4G36360
                                          +---------------------146.0-|
                                          |                           +------AT1G45130
                                          |
                                          |                           +------Os06g37560
                                          |                    +500.0-|
                                   +464.0-|             +443.0-|      +------Os02g12730
                                   |      |             |      |
                                   |      |      +281.0-|      +-------------AT4G26140
                                   |      |      |      |
                                   |      |      |      +--------------------AT3G13750
                                   |      |      |
                                   |      +144.0-|                    +------pg 7 112
                                   |             |             +500.0-|
                            +225.0-|             |      +406.0-|      +------Pt65345 25
                            |      |             |      |      |
                            |      |             +114.0-|      +-------------Os03g06940
                            |      |                    |
                            |      |                    +--------------------Os01g39830
                            |      |
                     +313.0-|      |                                  +------AT2G32810
                     |      |      |                           +471.0-|
                     |      |      +---------------------261.0-|      +------Pt29292 19
                     |      |                                  |
              +279.0-|      |                                  +-------------AT5G63810
              |      |      |
              |      |      +------------------------------------------------AT1G77410
              |      |
       +438.0-|      |                                                +------pg6 113
       |      |      +------------------------------------------500.0-|
       |      |                                                       +------Pt20917 25
+------|      |
|      |      +--------------------------------------------------------------Pt10182555
|      |
|      +---------------------------------------------------------------------AT2G28470
|
+----------------------------------------------------------------------------Os03g15020
```

Galactosidase beta – PARS

```
                                                               +------pg6 113
              +------------------------------------------500.0-|
              |                                                +------Pt20917 25
              |
              |                                                +------Pt29292 19
              |                    +---------------------428.0-|
              |                    |                           +------AT2G32810
              |                    |
              |                    |             +--------------------Os01g39830
              |                    |             |
              |                    |      +347.3-|             +------Os06g37560
              |             +273.7-|      |      |      +500.0-|
              |             |      |      |      +422.6-|      +------Os02g12730
              |             |      |      |             |
              |             |      |      |             +-------------AT4G26140
              |             |      |      |
       +423.5-|             |      |      |                    +------pg 7 112
       |      |             |      |      |             +498.7-|
       |      |             |      +458.3-|-------268.7-|      +------Pt65345 25
       |      |      +254.7-|             |             |
       |      |      |      |             |             +-------------Os03g06940
       |      |      |      |             |
       |      |      |      |             +---------------------------AT1G45130
       |      |      |      |             |
       |      +289.1-|      |             +---------------------------AT4G36360
+------|      |      |      |             |
|      |      |      |      |             +---------------------------AT3G13750
|      |      |      |      |
|      |      |      |      +-----------------------------------------AT5G63810
|      |      |      |
|      |      |      +------------------------------------------------AT1G77410
|      |      |
|      |      +-------------------------------------------------------Pt10182555
|      |
|      +--------------------------------------------------------------AT2G28470
|
+---------------------------------------------------------------------Os03g15020
```

Galactosidase beta - NJ

```
                                                        +-------------AT4G36360
                                                 +456.0-|
                                                 |      |      +------Os03g06940
                                                 |      +463.0-|
                                          +443.0-|             +------AT1G45130
                                          |      |
                                          |      |             +------Os01g39830
                                   +462.0-|      +-------296.0-|
                                   |      |                    +------AT3G13750
                                   |      |
                                   |      |             +-------------Os02g12730
                            +449.0-|      +-------113.0-|
                            |      |                    |      +------AT4G26140
                            |      |                    +271.0-|
                            |      |                           +------Os06g37560
                     +166.0-|      |
                     |      |      +----------------------------------pg 7 112
                     |      |
                     |      |                                  +------AT2G32810
                     |      |                           +460.0-|
              +358.0-|      +---------------------187.0-|      +------AT1G77410
              |      |                                  |
              |      |                                  +-------------AT5G63810
              |      |
       +482.0-|      |                                         +------Pt20917 25
       |      |      +-----------------------------------499.0-|
       |      |                                                +------pg5 116put
+------|      |
|      |      +-------------------------------------------------------Pt10182555
|      |
|      +--------------------------------------------------------------AT2G28470
|
+---------------------------------------------------------------------Os03g15020
```

Galactosidase beta – PARS

```
+---------------------------------------------------------------------Os03g15020
|
|                           +-----------------------------------------pg 7 112
|                           |
|                           |                                  +------Os06g37560
|                           |      +---------------------337.8-|
|                    +488.0-|      |                           +------AT4G26140
|                    |      |      |
|                    |      |      |             +--------------------Os01g39830
|                    |      |      |      +478.4-|
|                    |      |      |      |      |      +-------------AT4G36360
|                    |      +482.8-|      |      +484.2-|
|                    |             +468.5-|             |      +------Os03g06940
|             +265.9-|             |      |             +484.2-|
|             |      |             |      |                    +------AT1G45130
|             |      |             |      |
|             |      |             |      +---------------------------AT3G13750
|             |      |             |
|             |      |             +----------------------------------Os02g12730
|             |      |
|             |      +------------------------------------------------pg5 116
|      +451.3-|
|      |      |                                         +-------------AT2G32810
|      |      +-----------------------------------351.2-|
|      |      |                                         |      +------AT1G77410
|      |      |                                         +314.8-|
+------|      |                                                +------AT5G63810
       |      |
       |      +-------------------------------------------------------Pt20917 25
       |      |
       |      +-------------------------------------------------------Pt10182555
       |
       +--------------------------------------------------------------AT2G28470
```

Galactosidase - NJ

```
                                                                      +------AT4G36360
                                                               +421.0-|
                                          +--------------463.0-|      +------AT1G45130
                                          |                    |
                                          |                    +-------------Os03g06940
                                          |
                                   +383.0-|                    +-------------Os01g39830
                                   |      |             +485.0-|
                                   |      |             |      |      +------Os02g12730
                                   |      |      +338.0-|      +500.0-|
                                   |      |      |      |             +------Os06g37560
                            +448.0-|      +338.0-|      |
                            |      |             |      +--------------------AT3G13750
                            |      |             |
                     +246.0-|      |             +---------------------------AT4G26140
                     |      |      |
                     |      |      +-----------------------------------------Pt65345 25
                     |      |
              +338.0-|      +------------------------------------------------AT2G32810
              |      |
              |      |                                                +------Pt20917 25
              |      |                                         +500.0-|
              |      |                                         |      +------pg6 113
       +447.0-|      +-----------------------------------346.0-|
       |      |                                                |      +------Pt64344 21
       |      |                                                +252.0-|
       |      |                                                       +------AT5G63810
+------|      |
|      |      |                                                       +------Pt10182555
|      |      +-------------------------------------------------500.0-|
|      |                                                              +------pg9 33
|      |
|      +---------------------------------------------------------------------AT2G28470
|
+----------------------------------------------------------------------------Os03g15020
```

Galactosidase - PARS

```
+---------------------------------------------------------------------AT2G32810
|
|                                  +----------------------------------Pt64344 21
|                                  |
|                                  |      +---------------------------Pt65345 25
|                           +316.9-|      |
|                           |      |      |      +--------------------Os01g39830
|                           |      |      |      |
|                           |      +499.0-|      |      +-------------Os03g06940
|                           |             |      +423.4-|
|                           |             |      |      |      +------AT4G36360
|                           |             |      |      +301.4-|
|                           |             +402.6-|             +------AT1G45130
|                           |                    |
|                           |                    +--------------------AT4G26140
|                    +322.7-|                    |
|                    |      |                    +--------------------AT3G13750
|                    |      |                    |
|                    |      |                    |             +------Os02g12730
|                    |      |                    +-------500.0-|
|                    |      |                                  +------Os06g37560
|                    |      |
|             +454.0-|      |                                  +------pg6 113
|             |      |      +----------------------------499.7-|
|             |      |      |                                  +------Pt20917 25
|             |      |      |
|             |      |      +-----------------------------------------AT5G63810
|      +495.2-|      |
|      |      |      |                                         +------pg9 33
|      |      |      +-----------------------------------499.0-|
+------|      |                                                +------Pt10182555
       |      |
       |      +-------------------------------------------------------Os03g15020
       |
       +--------------------------------------------------------------AT2G28470
```

Galactosyltransferase - NJ

```
                                                                                           +-------------Os12g41956
                                          +------------------------------------------265.0-|
                                          |                                                |      +------AT4G21060
                                          |                                                +192.0-|
                                          |                                                       +------Os08g03670
                                          |
                                          |                                                       +------Os01g65590
                                          |                           +---------------------500.0-|
                                          |                           |                           +------Os05g35266
                                          |                           |
                                          |                    +319.0-|                           +------Pt40969 15
                                          |                    |      |      +--------------500.0-|
                                   +275.0-|                    |      |      |                    +------pg1 42put
                                   |      |                    |      |      |
                                   |      |                    |      +356.0-|                    +------Os06g07905
                                   |      |                    |             |             +498.0-|
                                   |      |             +382.0-|             |      +303.0-|      +------Os06g08005
                                   |      |             |      |             |      |      |
                                   |      |             |      |             +396.0-|      +-------------AT4G32120
                                   |      |             |      |                    |
                            +422.0-|      |      +500.0-|      |                    +--------------------AT2G25300
                            |      |      |      |      |      |
                            |      |      |      |      |      +-----------------------------------------Os08g29710
                            |      |      +419.0-|      |
                            |      |             |      +------------------------------------------------AT5G53340
                            |      |             |
                     +366.0-|      |             +-------------------------------------------------------AT3G06440
                     |      |      |
                     |      |      |                                                              +------Pt71196 38
                     |      |      +--------------------------------------------------------457.0-|
                     |      |                                                                     +------pg9 51
              +314.0-|      |
              |      |      |                                                                     +------Os03g58900
              |      |      +---------------------------------------------------------------401.0-|
       +254.0-|      |                                                                            +------Os03g58920
       |      |      |
       |      |      +-----------------------------------------------------------------------------------Os07g09690
+------|      |
|      |      +------------------------------------------------------------------------------------------AT5G62620
|      |
|      +-------------------------------------------------------------------------------------------------AT1G27120
|
+--------------------------------------------------------------------------------------------------------AT1G74800
```

Galactosyltransferase - PARS

```
                                                               +------Pt40969 15
                                                        +500.0-|
                                                 +257.0-|      +------pg1 42
                                                 |      |
                                                 |      +-------------AT5G53340
                                                 |
                                                 |             +------Os06g07905
                                                 |      +356.1-|
                                          +361.6-|      |      +------Os06g08005
                                          |      +339.9-|
                                          |      |      |      +------AT2G25300
                                          |      |      +491.3-|
                                   +500.0-|      |             +------AT4G32120
                                   |      |      |
                                   |      |      +--------------------Os08g29710
                                   |      |
                            +499.0-|      |                    +------Os05g35266
                            |      |      +--------------488.7-|
                            |      |                           +------Os01g65590
                     +273.5-|      |
                     |      |      +----------------------------------AT3G06440
                     |      |
              +391.5-|      +-----------------------------------------Os12g41956
              |      |
              |      +------------------------------------------------Os08g03670
              |      |
              |      +------------------------------------------------AT4G21060
              |
       +424.9-|                                                +------AT5G62620
       |      |                                                |
       |      |                                         +251.6-|------AT1G27120
       |      |                                         |      |
       |      |                                         |      +------AT1G74800
       |      |                                         |
+------|      +-----------------------------------404.5-|      +------Os03g58900
|      |                                                +261.3-|
|      |                                                |      +------Os03g58920
|      |                                                |
|      |                                                +-------------Os07g09690
|      |
|      +--------------------------------------------------------------Pt71196 38
|
+---------------------------------------------------------------------pg9 51
```

Galactosyltransferase NJ

```
                     +---------------------------Os08g29710
                     |
                     |             +-------------pg9 51
                     |             |
                     |             |      +------AT4G21060
                     |             +291.0-|
                     |             |      +------Os08g03670
                     |             |
                     |             +-------------Os03g58920
              +254.0-|             |
              |      |             +-------------Os03g58900
              |      |             |
              |      |      +499.0-|-------------AT5G62620
              |      |      |      |
              |      |      |      +-------------Os07g09690
              |      |      |      |
              |      |      |      +-------------Os12g41956
              |      |      |      |
              |      +500.0-|      +-------------AT1G27120
              |             |      |
              |             |      +-------------AT1G74800
              |             |      |
       +291.0-|             |      +-------------Pt71196 38
       |      |             |
       |      |             +--------------------AT3G06440
       |      |
       |      |                           +------Os06g07905
       |      |                    +500.0-|
       |      |                    |      +------Os06g08005
       |      +--------------500.0-|
       |      |                    |      +------AT2G25300
       |      |                    +500.0-|
       |      |                           +------AT4G32120
       |      |
       |      |                           +------Pt40969 15
       |      +---------------------287.0-|
       |                                  +------AT5G53340
       |
       +-----------------------------------------AT1G77810
       |
       |                           +-------------Os02g06840
       |                    +362.0-|
       |                    |      |      +------AT1G05170
       |                    |      +446.0-|
+------|             +312.0-|             +------AT2G32430
|      |             |      |
|      |             |      +--------------------AT4G26940
|      +-------254.0-|      |
|      |             |      +--------------------Os03g38050
|      |             |
|      |             +---------------------------Pt67708 23
|      |
|      +-----------------------------------------Pp4455 16
|      |
|      |                                  +------Os09g27950
|      +----------------------------469.0-|
|      |                                  +------Os02g35870
|      |
|      |                                  +------Os05g35266
|      +----------------------------497.0-|
|      |                                  +------Os01g65590
|      |
|      +-----------------------------------------pg10 31
|      |
|      +-----------------------------------------AT1G33430
|      |
|      +-----------------------------------------AT1G32930
|
+------------------------------------------------Pt13006 33
```

Galactosyltransferase - PARS

```
                                          +-----------------------------------------AT3G06440
                                          |
                                          |                                  +------AT4G21060
                                          |                           +461.9-|
                                          |                    +441.0-|      +------Os12g41956
                                          |                    |      |
                                          |             +388.4-|      +-------------Os08g03670
                                   +500.0-|             |      |
                                   |      |      +280.0-|      +--------------------pg9 51
                                   |      |      |      |
                                   |      |      |      +---------------------------Pt71196 38
                                   |      |      |
                                   |      |      +----------------------------------AT1G74800
                                   |      |      |
                                   |      |      +----------------------------------AT5G62620
                                   |      +476.8-|
                                   |             |                           +------Os03g58920
                                   |             +---------------------312.3-|
                                   |             |                           +------Os07g09690
                                   |             |
                                   |             +----------------------------------AT1G27120
                            +445.0-|             |
                            |      |             +----------------------------------Os03g58900
                            |      |
                            |      |                                         +------Os06g08005
                            |      |                                  +500.0-|
                            |      |                                  |      +------Os06g07905
                            |      +----------------------------498.0-|
                            |      |                                  |      +------AT4G32120
                            |      |                                  +496.7-|
                            |      |                                         +------AT2G25300
                            |      |
                            |      +------------------------------------------------Os08g29710
                            |      |
                     +325.5-|      +------------------------------------------------AT5G53340
                     |      |      |
                     |      |      +------------------------------------------------Pt40969 15
                     |      |
                     |      +-------------------------------------------------------Os03g38050
                     |      |
                     |      |                                                +------AT2G32430
                     |      +------------------------------------------433.6-|
                     |      |                                                +------AT1G05170
                     |      |
                     |      +-------------------------------------------------------Pt67708 23
                     |      |
                     |      +-------------------------------------------------------AT4G26940
                     |      |
              +273.0-|      +-------------------------------------------------------Os02g06840
              |      |
              |      |                                                       +------Os02g35870
              |      +-------------------------------------------------337.0-|
              |      |                                                       +------Os09g27950
              |      |
              |      +--------------------------------------------------------------pg10 31
              |      |
       +496.9-|      +--------------------------------------------------------------AT1G77810
       |      |      |
       |      |      +--------------------------------------------------------------Pp4455 16
       |      |      |
       |      |      +--------------------------------------------------------------Pt13006 33
+------|      |      |
|      |      |      +--------------------------------------------------------------AT1G33430
|      |      |
|      |      +---------------------------------------------------------------------AT1G32930
|      |
|      +----------------------------------------------------------------------------Os05g35266
|
+-----------------------------------------------------------------------------------Os01g65590
```

Geranylgeranyl pyrophosphate synthase - NJ

```
                                                +------pg5 14
                                         +429.0-|
                                         |      +------Pt63482550
                                  +359.0-|
                                  |      |      +------pg 5 15
                           +250.0-|      +497.0-|
                           |      |             +------Pt71308 18
                    +437.0-|      |
                    |      |      +--------------------Pt31282548
                    |      |
             +433.0-|      +---------------------------Pt57286 26
             |      |
             |      |                           +------Os02g44780
             |      +---------------------439.0-|
      +385.0-|                                  +------Os01g14630
      |      |
      |      |                                  +------Os07g39270
      |      |                    +-------346.0-|
      |      |                    |             +------AT2G18640
      |      +--------------257.0-|
+-----|                           |      +-------------AT3G29430
|     |                           +500.0-|
|     |                                  |      +------AT3G14550
|     |                                  +218.0-|
|     |                                         +------AT3G14530
|     |
|     +------------------------------------------------AT4G36810
|
+------------------------------------------------------AT2G18620
```

Geranylgeranyl pyrophosphate synthase - PARS

```
                                          +------AT3G14530
                                   +466.8-|
                            +500.0-|      +------AT3G14550
                            |      |
                            |      +-------------AT3G29430
                            |
                            |             +------Os07g39270
              +-------257.3-|-------282.1-|
              |             |             +------AT2G18640
              |             |
              |             +--------------------AT4G36810
              |             |
              |             +--------------------AT2G18620
              |
       +429.3-|                           +------Pt63482550
       |      |                    +357.8-|
       |      |                    |      +------pg5 14
       |      |             +360.3-|
       |      |             |      +-------------Pt71308 18
       |      |      +346.5-|      |
+------|      |      |      |      +-------------pg 5 15
|      |      +452.8-|      |
|      |             |      +--------------------Pt57286 26
|      |             |
|      |             +---------------------------Pt31282548
|      |
|      +-----------------------------------------Os01g14630
|
+------------------------------------------------Os02g44780
```

Gibberellin-regulated family protein NJ

```
                                                               +-------------Pt11282549
                                                        +216.0-|
                                                        |      |      +------Pt8411 14
                                                 +350.0-|      +179.0-|
                                                 |      |             +------Pt79800 7
                                          +222.0-|      |
                                          |      |      +--------------------Pt11282548
                                   +348.0-|      |
                                   |      |      +---------------------------Pt13082549
                            +132.0-|      |
                            |      |      +----------------------------------pg12 108
                     +140.0-|      |
                     |      |      +-----------------------------------------AT1G75750
              +274.0-|      |
              |      |      +------------------------------------------------AT1G74670
              |      |
              |      +-------------------------------------------------------AT1G22690
       +499.0-|
       |      |                           +----------------------------------Pt14896 7
       |      |                           |
       |      |                           |                    +-------------Pt63213 10
       |      +---------------------472.0-|             +436.0-|
       |                                  |             |      |      +------pg7 12
+------|                                  |      +336.0-|      +419.0-|
|      |                                  |      |      |             +------Pt28670 26
|      |                           +333.0-|             |
|      |                                  |             +--------------------Pt27443 7
|      |                                  |
|      |                                  +---------------------------Pp3987 9
|      |
|      +---------------------------------------------------------------------AT4G09610
|
+----------------------------------------------------------------------------AT4G09600
```

Gibberellin-regulated family protein - PARS

```
                                                 +------AT4G09610
                     +---------------------499.9-|
                     |                           +------AT4G09600
                     |
                     |                           +------Pt8411 14
                     |                           |
                     |                           +------Pt79800 7
                     |                           |
                     |                    +445.9-|------Pt11282549
              +499.8-|                    |      |
              |      |                    |      +------Pt11282548
              |      |             +499.9-|      |
              |      |             |      |      +------Pt13082549
              |      |             |      |
              |      |      +342.9-|      +-------------pg12 108
       +357.3-|      |      |      |
       |      |      |      |      |             +------AT1G22690
       |      |      +352.9-|      +-------266.3-|
       |      |             |                    +------AT1G74670
       |      |             |
       |      |             +---------------------------AT1G75750
+------|      |
|      |      +-----------------------------------------Pt63213 10
|      |
|      |                                         +------pg7 12
|      |                                  +391.6-|
|      |                           +396.2-|      +------Pt28670 26
|      |                           |      |
|      +---------------------284.3-|      +-------------Pt27443 7
|                                  |
|                                  +--------------------Pp3987 9
|
+-------------------------------------------------------Pt14896 7
```

Glycosyl hydrolase 1 - NJ

```
                                                                      +-------------Os08g39870
                                                                      |
                                   +----------------------------282.0-|      +------AT5G44640
                                   |                                  |      |
                                   |                                  |      +------AT2G44450
                                   |                                  +500.0-|
                                   |                                         +------AT2G25630
                                   |                                         |
                                   |                                         +------AT5G42260
                                   |
                                   |                                         +------AT3G18080
                                   |                                  +458.0-|
                                   |                           +500.0-|      +------AT3G18070
                                   |                           |      |
                                   |                    +264.0-|      +-------------Os01g32364
                                   |                    |      |
                                   |                    |      |             +------pg5 83 27
                                   |             +269.0-|      +-------500.0-|
                                   |             |      |                    +------Pt50282553
                                   |             |      |
                                   |      +376.0-|      +---------------------------Os07g46280
                                   |      |      |
                                   |      |      |                           +------Os03g49610
                                   +408.0-|      +---------------------499.0-|
                                   |      |                                  +------Os03g49600
                                   |      |
                                   |      +-----------------------------------------AT5G54570
                                   |
                                   +------------------------------------------------Os09g31430
                                   |
                                   |                                         +------Pt37490 20
                                   |                                  +334.0-|
                            +448.0-|                                  |      +------Pt56082548
                            |      |                                  |
                            |      |                                  +-------------Pt62749 19
                            |      +----------------------------465.0-|
                            |      |                                  |      +------Pt46682548
                            |      |                                  +500.0-|
                            |      |                                  |      +------Pt12616 40
                            |      |                                  |
                            |      |                                  +-------------pg4 75 49
                            |      |
                            |      |                                         +------Pt70347 18
                            |      +-----------------------------------500.0-|
                            |      |                                         +------pg 4 90 43
                            |      |
                            |      |                                  +-------------Os06g21570
                            |      |                           +471.0-|
                            |      |                           |      |      +------Os04g39880
                            |      +---------------------265.0-|      +492.0-|
                     +330.0-|      |                           |             +------Os04g39900
                     |      |      |                           |
                     |      |      |                           +--------------------AT2G44480
                     |      |      |
                     |      |      +------------------------------------------------Os08g39860
                     |      |      |
                     |      |      |                                  +-------------AT3G60140
                     |      |      +----------------------------500.0-|
                     |      |      |                                  |      +------AT5G24550
                     |      |      |                                  +487.0-|
              +492.0-|      |      |                                         +------AT5G24540
              |      |      |      |
              |      |      |      +------------------------------------------------AT1G47600
              |      |      |
              |      |      +-------------------------------------------------------AT1G26560
              |      |      |
              |      |      +-------------------------------------------------------Pt20849 24
       +471.0-|      |      |
       |      |      |      +-------------------------------------------------------Pt45282552
       |      |      |
       |      |      |                                                       +------Os03g11420
       |      |      +-------------------------------------------------499.0-|
+------|      |                                                              +------Os10g17650
|      |      |
|      |      |                                                              +------pg 12 115
|      |      +--------------------------------------------------------500.0-|
|      |                                                                     +------Pt2655 35
|      |
|      +----------------------------------------------------------------------------AT5G36890
|
+-----------------------------------------------------------------------------------Os01g67220
```

Glycosyl hydrolase 1 - PARS

```
                                                               +------AT5G24540
                                                        +490.6-|
                            +---------------------500.0-|      +------AT5G24550
                            |                           |
                            |                           +-------------AT3G60140
                            |
                            |                                  +------Os03g49610
                            |                    +-------471.4-|
                            |                    |             +------Os03g49600
                            |             +365.5-|
                            |             |      |             +------AT3G18080
                            |             |      |      +498.0-|
                            |             |      +459.0-|      +------AT3G18070
                            |             |             |
                            |      +292.7-|             +-------------Os01g32364
                            |      |      |
                            |      |      |                    +------Pt50282553
                            |      |      +--------------500.0-|
                            +292.2-|      |                    +------pg5 83 27
                            |      |      |
                            |      |      +---------------------------Os07g46280
                            |      |
                            |      +----------------------------------AT5G54570
                            |
                            |                                  +------Pt46682548
                            |             +--------------500.0-|
                            |             |                    +------Pt12616 40
                            +-------417.2-|
                            |             |      +--------------------Pt62749 19
                            |             |      |
                            |             +408.8-|             +------Pt37490 20
                            |                    |      +392.1-|
                     +370.4-|                    +383.7-|      +------pg4 75 49
                     |      |                           |
                     |      |                           +-------------Pt56082548
                     |      |
                     |      |                                  +------pg 4 90 43
                     |      +----------------------------500.0-|
                     |      |                                  +------Pt70347 18
                     |      |
                     |      +-----------------------------------------AT2G44480
                     |      |
                     |      |                                  +------AT5G44640
                     |      |                                  |
                     |      |                                  +------AT2G44450
                     |      +----------------------------500.0-|
                     |      |                                  +------AT5G42260
                     |      |                                  |
                     |      |                                  +------AT2G25630
                     |      |
                     |      +-----------------------------------------Os09g31430
              +445.9-|      |
              |      |      +-----------------------------------------Os08g39860
              |      |      |
              |      |      |                                  +------Os04g39880
              |      |      |                           +351.6-|
              |      |      +---------------------443.4-|      +------Os04g39900
              |      |      |                           |
              |      |      |                           +-------------Os06g21570
              |      |      |
              |      |      +-----------------------------------------Os08g39870
              |      |      |
              |      |      +-----------------------------------------AT1G47600
       +460.6-|      |
       |      |      |                                         +------Pt45282552
       |      |      |                                  +283.0-|
       |      |      +----------------------------269.0-|      +------Pt20849 24
       |      |      |                                  |
       |      |      |                                  +-------------AT1G26560
       |      |      |
+------|      |      |                                         +------Os10g17650
|      |      |      +-----------------------------------456.6-|
|      |      |                                                +------Os03g11420
|      |      |
|      |      |                                                +------pg 12 115
|      |      +------------------------------------------437.4-|
|      |                                                       +------Pt2655 35
|      |
|      +--------------------------------------------------------------AT5G36890
|
+---------------------------------------------------------------------Os01g67220
```

Glycosyl hydrolase 9 NJ

```
+--------------------------------------------------------------AT1G19940
|
|                                                       +------AT1G64390
|                                                +500.0-|
|                                         +446.0-|      +------AT4G11050
|                                         |      |
|                                  +483.0-|      +-------------Os02g50490
|                                  |      |
|                           +458.0-|      +--------------------Os04g57860
|                           |      |
|                    +500.0-|      +---------------------------AT2G44540
|                    |      |
|                    |      |                           +------Pt91982554
|             +458.0-|      +---------------------500.0-|
|             |      |                                  +------pg 5 141
|             |      |
|             |      |                                  +------pg1 76
|      +495.0-|      +----------------------------500.0-|
|      |      |                                         +------Pt53582556
|      |      |
+------|      |                                         +------Os09g36350
       |      +-----------------------------------396.0-|
       |                                                +------Os06g50140
       |
       +-------------------------------------------------------AT1G75680
```

Glycosyl hydrolase 9 - PARS

```
                                   +-------------Os04g57860
                                   |
                                   |      +------AT1G64390
                            +305.7-|-499.5-|
                            |      |      +------AT4G11050
                     +310.9-|      |
                     |      |      +-------------Os02g50490
                     |      |
              +500.0-|      +--------------------AT2G44540
              |      |
              |      |                    +------Pt91982554
              |      +--------------499.0-|
       +309.5-|                           +------pg 5 141
       |      |
       |      +----------------------------------Os06g50140
       |      |
+------|      |                           +------pg1 76
|      |      +---------------------500.0-|
|      |                                  +------Pt53582556
|      |
|      |                                  +------AT1G19940
|      +----------------------------338.4-|
|                                         +------AT1G75680
|
+------------------------------------------------Os09g36350  |
```

Glycosyl hydrolase 17 - NJ

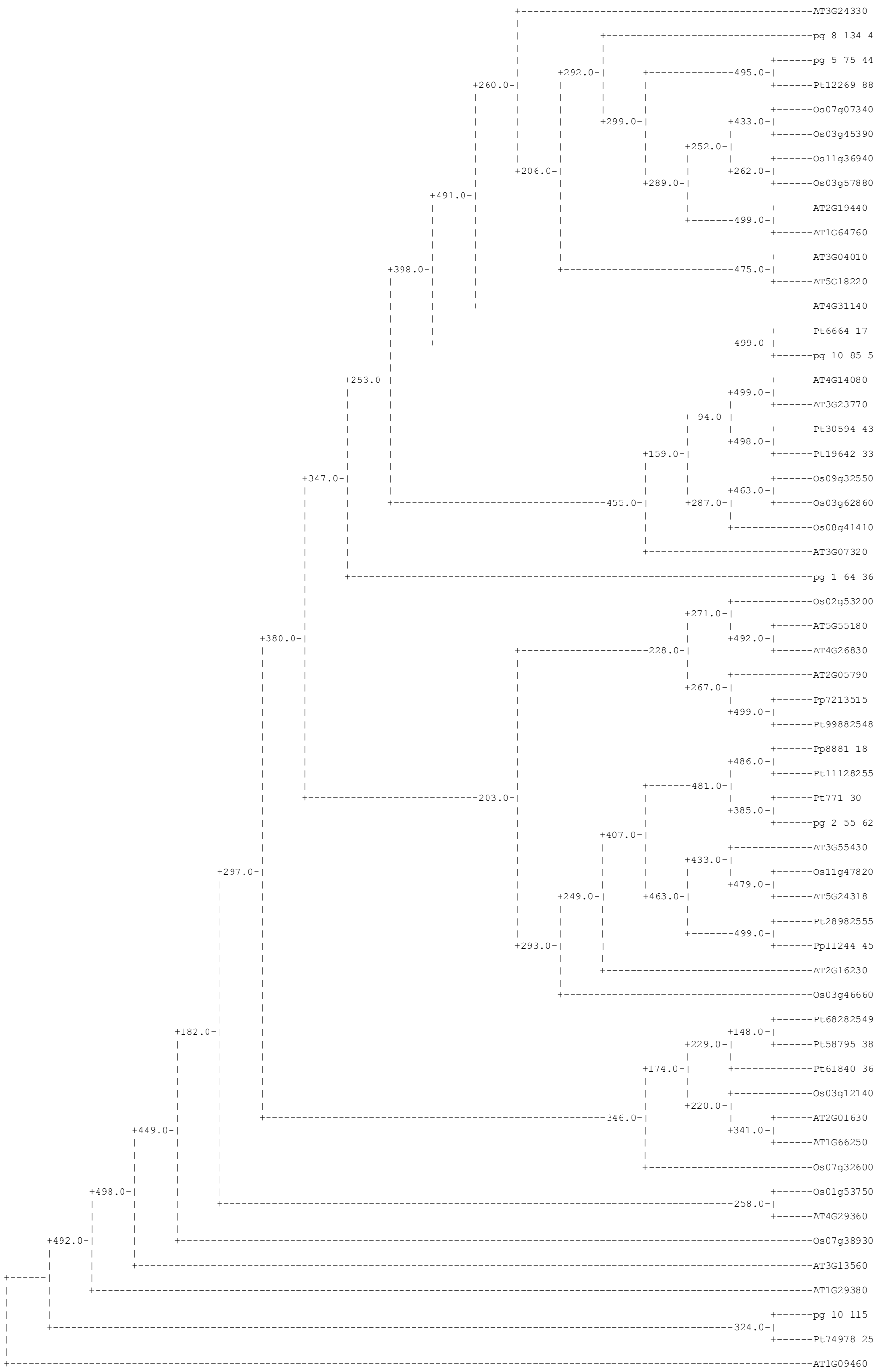

Glycosyl hydrolase 17 - PARS

```
                                                                      +------AT3G13560
                                   +----------------------------256.3-|
                                   |                                  +------Os07g38930
                                   |
                                   |                                  +------AT5G55180
                                   +----------------------------464.3-|
                                   |                                  +------AT4G26830
                                   |
                                   |                                  +------AT5G24318
                                   |                           +321.8-|
                                   |                    +413.6-|      +------AT3G55430
                                   |                    |      |
                                   |             +322.6-|      +-------------Os11g47820
                                   |             |      |
                                   |             |      |             +------Pt28982555
                                   |             |      +-------500.0-|
                                   |      +361.8-|                    +------Pp11244 45
                                   |      |      |
                                   |      |      |                    +------pg 2 55 62
                                   |      |      |             +465.5-|
                                   |      |      |             |      +------Pt771 30
                                   |      |      +-------456.9-|
                                   +273.5-|                    |      +------Pt11128255
                                   |      |                    +480.6-|
                                   |      |                           +------Pp8881 18
                                   |      |
                                   |      +----------------------------------Os03g46660
                                   |      |
                                   |      +----------------------------------AT2G16230
                                   |
                                   |                                  +------Pt6664 17
                                   +----------------------------500.0-|
                                   |                                  +------pg 10 85 5
                                   |
                                   |                                  +------Pt19642 33
                                   +----------------------------487.3-|
                                   |                                  +------Pt30594 43
                                   |
                            +287.5-|                                  +------AT2G01630
                            |      |                           +364.9-|
                            |      |                           |      +------AT1G66250
                            |      |                           |
                            |      |                           +-------------Os03g12140
                            |      |                           |
                            |      +---------------------274.5-|      +------Os07g32600
                            |      |                           +305.7-|
                            |      |                           |      +------Pt61840 36
                            |      |                           |
                            |      |                           +-------------Pt68282549
                            |      |                           |
                            |      |                           +-------------Pt58795 38
                            |      |
                            |      |                                  +------AT4G14080
                            |      +----------------------------487.3-|
                            |      |                                  +------AT3G23770
                            |      |
                            |      |                                  +------AT4G29360
                            |      +----------------------------465.8-|
                            |      |                                  +------Os01g53750
                            |      |
                            |      +-----------------------------------------AT2G05790
                            |      |
                            |      +-----------------------------------------AT3G07320
                            |      |
                            |      +-----------------------------------------Os03g62860
                     +500.0-|      |
                     |      |      +-----------------------------------------Os08g41410
                     |      |      |
                     |      |      +-----------------------------------------pg 1 64 36
                     |      |      |
                     |      |      |                                  +------Pt99882548
                     |      |      +----------------------------500.0-|
                     |      |      |                                  +------Pp7213515
                     |      |      |
                     |      |      +-----------------------------------------Os02g53200
                     |      |      |
                     |      |      +-----------------------------------------Os09g32550
                     |      |
                     |      |                           +--------------------Os11g36940
                     |      |                           |
                     |      |                           |             +------AT1G64760
                     |      |                           |      +500.0-|
                     |      |                           |      |      +------AT2G19440
                     |      |                    +315.8-|-372.4-|
              +296.4-|      |                    |      |      |      +------Os03g45390
              |      |      |                    |      |      +452.8-|
              |      |      |                    |      |             +------Os07g07340
              |      |      |                    |      |
              |      |      |                    |      +--------------------Os03g57880
              |      |      |                    |
              |      |      |                    |                    +------AT3G04010
              |      |      |                    +--------------383.2-|
              |      |      +--------------495.3-|                    +------AT5G18220
              |      |                           |
              |      |                           +---------------------------pg 8 134 4
       +500.0-|      |                           |
       |      |      |                           |                    +------Pt12269 88
       |      |      |                           +--------------491.6-|
       |      |      |                           |                    +------pg 5 75 44
       |      |      |                           |
       |      |      |                           +---------------------------AT4G31140
+------|      |      |                           |
|      |      |      |                           +---------------------------AT3G24330
|      |      |      |
|      |      |      +-------------------------------------------------------AT1G09460
|      |      |
|      |      +--------------------------------------------------------------AT1G29380
|      |
|      +---------------------------------------------------------------------pg 10 115
|
+----------------------------------------------------------------------------Pt74978 25
```

Glycosyl hydrolase 18 - NJ

```
                                                                      +------Pt77774 15
                                                               +193.0-|
                                                        +473.0-|      +------Pt77778 4
                                                        |      |
                                                 +488.0-|      +-------------Pt12082555
                                                 |      |
                                          +495.0-|      +--------------------pg 12 85
                                          |      |
                                   +493.0-|      +---------------------------Pt48789 19
                                   |      |
                            +500.0-|      +----------------------------------pg 12 76
                            |      |
                     +500.0-|      +-----------------------------------------Pt42603 23
                     |      |
                     |      |                                         +------Os04g30770
              +434.0-|      +-----------------------------------462.0-|
              |      |                                                +------Os11g27400
              |      |
       +441.0-|      |                                                +------AT4G19770
       |      |      +------------------------------------------487.0-|
       |      |                                                       +------AT4G19800
+------|      |
|      |      +--------------------------------------------------------------AT4G19750
|      |
|      +---------------------------------------------------------------------AT4G19810
|
+----------------------------------------------------------------------------AT4G19820
```

Glycosyl hydrolase 18 - PARS

```
                                                  +------AT4G19800
              +----------------------------500.1-|
              |                                  +------AT4G19770
              |
              |                                  +------pg 12 85
              |                                  |
              |                                  +------Pt12082555
              |                           +480.5-|
              |                           |      +------Pt77774 15
       +272.8-|                    +479.5-|      |
       |      |                    |      |      +------Pt77778 4
       |      |                    |      |
       |      |             +500.1-|      +-------------Pt48789 19
       |      |             |      |
       |      |             |      +--------------------Pt42603 23
       |      |      +337.8-|      |
+------|      |      |      |      +--------------------pg 12 76
|      |      +495.1-|      |
|      |             |      +---------------------------Os11g27400
|      |             |
|      |             +----------------------------------Os04g30770
|      |
|      |                                         +------AT4G19820
|      +-----------------------------------497.9-|
|                                                +------AT4G19810
|
+-------------------------------------------------------AT4G19750
```

Glycosyl hydrolase 28 - NJ

```
                            +-------------Pt48482550
                     +499.0-|
                     |      |      +------Pt32482556
                     |      +323.0-|
              +492.0-|             +------Os07g14160
              |      |
              |      |             +------pg4 109
              |      |      +482.0-|
       +352.0-|      +500.0-|      +------pg4 110
       |      |             |
       |      |             +-------------Pt29416 28
       |      |
+------|      +---------------------------Os03g03350
|      |
|      |                           +------AT3G48950
|      |                    +481.0-|
|      +--------------456.0-|      +------AT2G23900
|                           |
|                           +-------------AT4G23500
|
+-----------------------------------------AT3G61490
```

Glycosyl hydrolase 28 - PARS

```
                                          +------pg4 109
                                   +497.3-|
                            +500.0-|      +------pg4 110
                            |      |
                     +446.2-|      +-------------Pt29416 28
                     |      |
                     |      |      +-------------Pt48482550
                     |      +500.0-|
              +374.3-|             |      +------Pt32482556
              |      |             +465.7-|
              |      |                    +------Os07g14160
       +445.5-|      |
       |      |      +---------------------------Os03g03350
       |      |
+------|      |                           +------AT3G61490
|      |      +---------------------328.6-|
|      |                                  +------AT4G23500
|      |
|      +-----------------------------------------AT3G48950
|
+------------------------------------------------AT2G23900
```

Glycosyl transferase family 8 - NJ

```
                                                               +------Os03g47530
                                                        +445.0-|
                                                 +260.0-|      +------Os07g45260
                                                 |      |
                                                 |      +-------------Os04g44850
                                                 |
                                                 |             +------AT1G02720
                                                 |      +444.0-|
                                                 +291.0-|      +------AT4G02130
                                                 |      |
                                                 |      +-------------AT3G62660
                                                 |
                                                 +--------------------pg 5 67
                                                 |
                                                 +--------------------AT1G13250
                                                 |
                                          +367.0-|--------------------AT3G06260
                                          |      |
                                          |      +--------------------pg8 60
                                          |      |
                                          |      +--------------------Pt48807 20
                                          |      |
                                          |      +--------------------Os03g18890
                                          |      |
                                   +433.0-|      |             +------Os06g13760
                                   |      |      +-------309.0-|
                                   |      |      |             +------Os02g50600
                                   |      |      |
                                   |      |      +--------------------AT1G70090
                            +496.0-|      |      |
                            |      |      |      +--------------------Os03g24510
                            |      |      |
                     +339.0-|      |      +---------------------------AT1G19300
                     |      |      |
                     |      |      +----------------------------------pg3 69
                     |      |
                     |      +-----------------------------------------Os08g38740
                     |
                     |                                         +------pg3 60
                     |                                         |
                     |                                         +------Pp8611 52
                     |                           +-------500.0-|
                     |                           |             +------Pt24582549
                     |                           |             |
                     |                    +414.0-|             +------Pt15666 13
                     |                    |      |
                     |                    |      |      +-------------AT3G02350
                     |                    |      +289.0-|
                     |             +402.0-|             |      +------AT3G25140
                     |             |      |             +481.0-|
                     |             |      |                    +------Os02g29530
                     |             |      |
                     |             |      +---------------------------Os06g12280
                     |             |
                     |      +281.0-|                           +------pg5 103
                     |      |      |                    +500.0-|
                     |      |      |             +383.0-|      +------Pt41071 27
                     |      |      |             |      |
                     |      |      |      +405.0-|      +-------------Pt9016 18
                     |      |      |      |      |
                     +279.0-|      |      |      |             +------AT2G20810
                     |      |      +481.0-|      +-------456.0-|
                     |      |             |                    +------Os04g54360
              +470.0-|      |             |
              |      |      |             +---------------------------Os03g30000
              |      |      |
              |      |      |                                  +------Pt5765 14
              |      |      +----------------------------500.0-|
              |      |                                         +------pg 11 98
              |      |
              |      |                                         +------AT5G15470
              |      |                                  +500.0-|
              |      |                           +351.0-|      +------AT3G01040
              |      |                           |      |
              |      |                           |      +-------------Os03g11330
              |      |                           |
              |      |                           |             +------Os01g52710
              |      |                           +-------454.0-|
              |      |                           |             +------AT3G58790
       +498.0-|      |                    +428.0-|
       |      |      |                    |      |             +------pg 9 123
       |      |      |                    |      |      +497.0-|
       |      |      |                    |      +491.0-|      +------Pp1213521
       |      |      +--------------362.0-|      |      |
       |      |      |                    |      |      +-------------Pt6618 14
       |      |      |                    |      |
       |      |      |                    |      +--------------------Os12g38930
       |      |      |                    |
       |      |      |                    +---------------------------AT5G54690
+------|      |      |
|      |      |      +------------------------------------------------AT4G38270
|      |      |      |
|      |      |      +------------------------------------------------Os09g30280
|      |      |      |
|      |      |      +------------------------------------------------AT5G47780
|      |      |
|      |      +-------------------------------------------------------Os06g49810
|      |
|      +--------------------------------------------------------------AT3G61130
|      |
|      +--------------------------------------------------------------Pt2080 29
|
+---------------------------------------------------------------------Os09g36190
```

Glycosyl transferase family 8 - PARS

```
+---------------------------------------------------------------Os09g36190
 |
 |                                          +--------------------Os12g38930
 |                                          |
 |                                          |      +-------------Pt6618 14
 |                                          +466.1-|
 |                                          |      |      +------pg 9 123
 |                                          |      +496.1-|
 |                                          |             +------Pp1213521
 |                                   +322.3-|
 |                                   |      |             +------AT3G58790
 |                                   |      +-------335.8-|
 |                                   |      |             +------Os01g52710
 |                                   |      |
 |                            +365.2-|      |             +------AT5G15470
 |                            |      |      |      +495.4-|
 |                            |      |      +347.7-|      +------AT3G01040
 |                            |      |             |
 |                            |      |             +-------------Os03g11330
 |                            |      |
 |                            |      +---------------------------AT5G54690
 |                            |
 |                            |                    +-------------Os03g24510
 |                            |                    |
 |                            |                    |      +------Os04g44850
 |                            |                    |      |
 |                            |                    |      +------Os06g13760
 |                            |                    +310.8-|
 |                            |                    |      +------AT3G06260
 |                            |                    |      |
 |                            |                    |      +------Os02g50600
 |                            |                    |
 |                            |                    |      +------AT3G62660
 |                            |                    |      |
 |                            |                    |      +------AT1G02720
 |                            |                    +298.2-|
 |              +-------307.8-|                    |      +------Os03g18890
 |              |             |             +275.7-|      |
 |              |             |             |      |      +------AT4G02130
 |              |             |             |      |
 |              |             |             |      +-------------AT1G13250
 |              |             |             |      |
 |              |             |             |      +-------------Pt48807 20
 |              |             |             |      |
 |              |             |             |      +-------------Os07g45260
 |              |             |      +497.9-|      |
 |              |             |      |      |      +-------------pg 5 67
 |              |             |      |      |      |
 |              |             |      |      |      +-------------pg8 60
 |              |             |      |      |      |
 |              |             +485.6-|      |      +-------------Os03g47530
 |              |             |      |      |      |
 |              |             |      |      |      +-------------AT1G70090
 |              |             |      |      |
 |              |             |      |      +--------------------AT1G19300
 |              |             |      |
 |              |             |      +---------------------------pg3 69
 |              |             |
 |              |             |                           +------pg 11 98
 |              |             +--------------------------499.9-|
 |              |                                         +------Pt5765 14
 |              |
 |              |                                         +------Pt15666 13
 |              |                                         |
 |              |                                         +------Pp8611 52
 |              |                                  +491.7-|
 |      +462.0-|                                  |      +------pg3 60
 |      |      |                                  |      |
 |      |      |                           +278.9-|      +------Pt24582549
 |      |      |                           |      |
 |      |      |                           |      +-------------Os02g29530
 |      |      |                    +356.0-|      |
 |      |      |                    |      |      +-------------AT3G25140
 |      |      |             +458.3-|      |
 |      |      |             |      |      +--------------------AT3G02350
 |      |      |             |      |
 |      |      |      +429.3-|      +---------------------------Os06g12280
 |      |      |      |      |
 |      |      |      |      |      +---------------------------Os03g30000
 |      |      |      |      |      |
 |      |      |      |      +473.1-|                    +------Os04g54360
 |      |      |      |             |      +-------465.9-|
 |      |      |      |             |      |             +------AT2G20810
 |      |      +345.9-|             +451.9-|
 +------|      |      |                    |             +------Pt41071 27
        |      |      |                    |      +493.9-|
        |      |      |                    +295.7-|      +------pg5 103
        |      |      |                           |
        |      |      |                           +-------------Pt9016 18
        |      |      |
        |      |      +-----------------------------------------AT4G38270
        |      |
        |      +------------------------------------------------AT5G47780
        |      |
        |      +------------------------------------------------Os08g38740
        |      |
        |      +------------------------------------------------Os09g30280
        |
        +-------------------------------------------------------Os06g49810
        |
        +-------------------------------------------------------Pt2080 29
        |
        +-------------------------------------------------------AT3G61130
```

Glycosyl transferase family 48 - NJ

```
                     +-------------AT3G59100
              +500.0-|
              |      |      +------AT1G06490
              |      +397.0-|
       +262.0-|             +------Os01g34880
       |      |
       |      |      +-------------pg3 100 15
       |      +348.0-|
       |             |      +------Pt70271 18
       |             +496.0-|
       |                    +------pg5 54 20
       |
       |                    +------AT1G05570
+------|--------------500.0-|
|      |                    +------AT2G31960
|      |
|      |                    +------Os06g08380
|      |             +500.0-|
|      +-------269.0-|      +------AT2G13680
|      |             |
|      |             +-------------Os03g03610
|      |
|      +---------------------------Os06g51270
|
+----------------------------------Os02g58560
```

Glycosyl transferase family 48 - PARS

```
                                   +-------------AT1G06490
                            +500.0-|
                            |      |      +------Os01g34880
                     +287.7-|      +317.6-|
                     |      |             +------AT3G59100
                     |      |
                     |      +--------------------Os03g03610
                     |
              +327.9-|                    +------AT2G13680
              |      +-------------500.0-|
              |      |                    +------Os06g08380
              |      |
              |      +---------------------------Os06g51270
       +500.0-|      |
       |      |      +---------------------------Os02g58560
       |      |
       |      +----------------------------------pg3 100 15
+------|      |
|      |      |                           +------pg5 54 20
|      |      +--------------------490.4-|
|      |                                  +------Pt70271 18
|      |
|      +-----------------------------------------AT1G05570
|
+------------------------------------------------AT2G31960
```

GRAS - NJ

```
                                                  +------AT3G03450
                                   +-------499.0-|
                                   |             +------AT5G17490
                                   |
                                   |      +-------------Os03g49990
                            +397.0-|-404.0-|
                            |      |      |      +------AT1G14920
                            |      |      +499.0-|
                            |      |             +------AT2G01570
                            |      |
              +-------482.0-|      +--------------------AT1G66350
              |             |
              |             |                    +------Os05g49930
              |             |             +487.0-|
              |             |             |      +------Os01g45860
              |             +-------443.0-|
              |                           |      +------Pt33263 16
              |                           +499.0-|
              |                                  +------pg3 3 107
              |
              +-----------------------------------------Os11g31100
       +304.0-|
       |      |                                  +------AT1G12970
       |      +----------------------------500.0-|
       |      |                                  +------AT3G26500
       |      |
       |      |             +---------------------------Pt77182549
       |      |             |
       |      |      +375.0-|             +-------------Os07g01240
       |      |      |      |      +261.0-|
       |      |      |      |      |      |      +------Pt62554 39
       |      |      |      +364.0-|      +483.0-|
       |      +500.0-|             |             +------pg12 104 4
       |             |             |
       |             |             +--------------------AT2G30700
       |             |
       |             +----------------------------------AT1G61900
       |
       |                                         +------AT1G21450
+------|                                  +334.0-|
|      +----------------------------389.0-|      +------Os01g65900
|      |                                  |
|      |                                  +-------------Pt25436 67
|      |
|      |                                         +------Pt66682 24
|      |                                  +474.0-|
|      |                                  |      +------AT1G50600
|      |                           +310.0-|
|      |                           |      |      +------pg 12 92 4
|      +---------------------274.0-|      +436.0-|
|      |                           |             +------Pt52969 21
|      |                           |
|      |                           +--------------------Os07g39470
|      |
|      +------------------------------------------------AT2G04890
|      |
|      |                                         +------Os10g22430
|      +-----------------------------------484.0-|
|                                                +------Os03g09280
|
+-------------------------------------------------------AT5G48150
```

GRAS - PARS

```
                                                               +------Os01g45860
                                                        +500.0-|
                                                        |      +------Os05g49930
                                                 +293.4-|
                                                 |      |      +------Pt33263 16
                                                 |      +457.3-|
                                                 |             +------pg3 3 107
                                                 |
                                                 +--------------------Os03g49990
                                                 |
                            +--------------500.0-|--------------------AT1G66350
                            |                    |
                            |                    |             +------AT5G17490
                            |                    +-------429.0-|
                            |                    |             +------AT3G03450
                            |                    |
                            |                    |             +------AT1G14920
                            |                    +-------499.5-|
                            |                                  +------AT2G01570
                            |
                            |                           +-------------AT2G30700
                     +425.3-|                    +498.6-|
                     |      |                    |      |      +------Pt62554 39
                     |      |                    |      +500.0-|
                     |      |             +500.0-|             +------pg12 104 4
                     |      |             |      |
                     |      |             |      |             +------Os07g01240
                     |      |             |      |      +319.2-|
                     |      |      +499.1-|      +280.3-|      +------AT1G61900
                     |      |      |      |             |
                     |      |      |      |             +-------------Pt77182549
                     |      |      |      |
                     |      +453.4-|      |                    +------AT1G12970
                     |             |      +--------------500.0-|
                     |             |                           +------AT3G26500
              +256.5-|             |
              |      |             +----------------------------------Os11g31100
              |      |
              |      |                                         +------Os01g65900
              |      +-----------------------------------275.8-|
              |      |                                         +------AT1G21450
              |      |
              |      +------------------------------------------------AT5G48150
              |      |
              |      |                                         +------Os03g09280
       +442.0-|      +-----------------------------------500.0-|
       |      |      |                                         +------Os10g22430
       |      |      |
       |      |      +------------------------------------------------AT2G04890
       |      |      |
       |      |      +------------------------------------------------Pt25436 67
+------|      |
|      |      |                                                +------pg 12 92 4
|      |      +------------------------------------------498.2-|
|      |      |                                                +------Pt52969 21
|      |      |
|      |      +-------------------------------------------------------Os07g39470
|      |
|      +--------------------------------------------------------------AT1G50600
|
+---------------------------------------------------------------------Pt66682 24
```

GTP-binding family protein - NJ

```
                                                               +------Os11g38020
                                                        +410.0-|
       +------------------------------------------366.0-|      +------pg 12 43 3
       |                                                |
       |                                                +-------------AT5G57960
       |
       |             +------------------------------------------------AT5G18570
       |             |
       |             |                                         +------pg 12 75 2
       |             |                                  +500.0-|
+------|             |                           +343.0-|      +------Pt49027 17
|      |             |                           |      |
|      |      +350.0-|                    +500.0-|      +-------------Os05g35540
|      |      |      |                    |      |
|      |      |      |             +425.0-|      +--------------------AT4G02790
|      |      |      |             |      |
|      |      |      |             |      |                    +------AT2G27200
|      |      |      |      +335.0-|      +--------------390.0-|
|      +497.0-|      |      |      |                           +------AT1G52980
|             |      +497.0-|      |
|             |             |      +----------------------------------AT3G07050
|             |             |
|             |             +-----------------------------------------AT2G41670
|             |
|             +-------------------------------------------------------AT3G49730
|
+---------------------------------------------------------------------AT5G52210
```

GTP-binding family protein - PARS

```
                                                        +------pg 12 75 2
                                                 +452.0-|
                                                 |      +------Pt49027 17
                                          +499.3-|
                                          |      |      +------Os05g35540
                                          |      +255.6-|
                                   +355.7-|             +------AT4G02790
                                   |      |
                                   |      |             +------AT2G41670
                                   |      |      +475.3-|
                            +472.7-|      +321.0-|      +------AT3G07050
                            |      |             |
                            |      |             +-------------AT1G52980
                     +334.4-|      |
                     |      |      +---------------------------AT2G27200
              +400.9-|      |
              |      |      +----------------------------------AT5G18570
       +500.0-|      |
       |      |      +-----------------------------------------AT3G49730
       |      |
+------|      +------------------------------------------------AT5G52210
|      |
|      +-------------------------------------------------------Os11g38020
|      |
|      +-------------------------------------------------------AT5G57960
|
+--------------------------------------------------------------pg 12 43 3
```

Haloacid dehalogenase-like hydrolase - NJ

```
       +------------------------------------------------pg 4 127 2
       |
       |                           +--------------------pg 8 90 20
       |                           |
       |                           |             +------Pt5769 92
       |                           +-------444.0-|
       |                           |             +------Os10g27220
       |                           |
       |                           +--------------------Os05g01030
       |                           |
       |                           +--------------------AT3G27870
       |                    +251.0-|
       |                    |      |      +-------------AT1G26130
       |                    |      |      |
       |                    |      |      |      +------AT3G25610
       |                    |      +421.0-|-275.0-|
       |             +329.0-|      |      |      +------AT1G13210
       |             |      |      |      |
       |             |      |      |      +-------------AT1G68710
       |             |      |      |
       |      +490.0-|      |      +--------------------Os06g29380
       |      |      |      |
+------|      |      |      +---------------------------Os06g36990
|      |      |      |
|      |      |      +----------------------------------Os08g29150
|      |      |
|      |      |                                  +------pg 11 1 21
|      +385.0-|                           +286.0-|
|      |      |                    +423.0-|      +------Pt24430 47
|      |      |                    |      |
|      |      |             +452.0-|      +-------------AT2G41250
|      |      |             |      |
|      |      |             |      +--------------------Os07g46520
|      |      |             |
|      |      +-------267.0-|                    +------Os03g36750
|      |                    +--------------360.0-|
|      |                    |                    +------Os08g37940
|      |                    |
|      |                    +---------------------------pg 6 104 2
|      |
|      |                                         +------AT3G10970
|      +-----------------------------------422.0-|
|      |                                         +------Os04g47020
|      |
|      +------------------------------------------------AT4G11570
|
+-------------------------------------------------------Os09g39560
```

Haloacid dehalogenase-like hydrolase - PARS

```
                                          +------Os07g46520
                                   +425.9-|
                                   |      +------AT2G41250
                            +500.0-|
                            |      |      +------Pt24430 47
                            |      +500.0-|
                            |             +------pg 11 1 21
              +-------445.5-|
              |             |             +------Os03g36750
              |             +-------344.1-|
              |             |             +------Os08g37940
              |             |
              |             +--------------------pg 6 104 2
              |
              +----------------------------------Os04g47020
              |
              |                           +------AT1G26130
              |                           |
              |                           +------AT3G25610
              |                    +496.9-|
              |                    |      +------AT1G68710
       +362.8-|                    |      |
       |      |                    |      +------AT1G13210
       |      |                    |
       |      |                    |      +------Os06g36990
       |      |                    +366.6-|
       |      |             +394.5-|      +------pg 8 90 20
       |      |             |      |
       |      |             |      +-------------AT3G27870
       |      |             |      |
       |      |             |      |      +------Os05g01030
       |      |      +500.0-|      +320.6-|
+------|      |      |      |      |      +------Os06g29380
|      |      |      |      |      |
|      |      |      |      |      +-------------Os08g29150
|      |      +258.5-|      |
|      |             |      |             +------Os10g27220
|      |             |      +-------486.7-|
|      |             |                    +------Pt5769 92
|      |             |
|      |             +---------------------------AT3G10970
|      |
|      +-----------------------------------------Os09g39560
|      |
|      +-----------------------------------------AT4G11570
|
+------------------------------------------------pg 4 127 2
```

Heat shock protein 18.1 kDa class I - NJ

```
                                                               +------Os01g04370
                     +-----------------------------------376.0-|
                     |                                         +------Os01g04380
                     |
                     |                                         +------AT4G10250
                     |                                  +500.0-|
                     |                                  |      +------Os04g36750
                     |                           +296.0-|
                     |                           |      |      +------Os02g03570
                     |                           |      +350.0-|
                     |                    +411.0-|             +------Os01g04340
                     |                    |      |
                     |                    |      |             +------AT1G07400
              +464.0-|                    |      |      +395.0-|
              |      |                    |      +496.0-|      +------AT2G29500
              |      |             +372.0-|             |
              |      |             |      |             +-------------AT1G59860
              |      |             |      |
              |      |             |      |                    +------AT1G53540
              |      |             |      |             +483.0-|
              |      |      +376.0-|      +-------482.0-|      +------AT3G46230
              |      |      |      |                    |
              |      |      |      |                    +-------------AT5G59720
              |      |      |      |
       +485.0-|      |      |      |             +--------------------pg3 3 40 3
       |      |      |      |      |             |
       |      |      +279.0-|      +-------495.0-|             +------Pt40082550
       |      |             |                    |      +500.0-|
       |      |             |                    +398.0-|      +------pg 5 110 2
       |      |             |                           |
       |      |             |                           +-------------Pt11338255
+------|      |             |
|      |      |             +-----------------------------------------Os01g04360
|      |      |
|      |      |                                                +------Os03g15960
|      |      +------------------------------------------498.0-|
|      |                                                       +------Os03g16030
|      |
|      +--------------------------------------------------------------Os03g16040
|
+---------------------------------------------------------------------Os03g16020
```

Heat shock protein 18.1 kDa class I - PARS

```
+--------------------------------------------------------------AT1G53540
|
|                                                       +------pg 5 110 2
|                                                +500.0-|
|                                                |      +------Pt40082550
|                           +--------------421.6-|
|                           |                    |      +------pg3 3 40 3
|                           |                    +487.0-|
|                           |                           +------Pt11338255
|                           |
|                    +286.5-|                           +------AT1G07400
|                    |      |                    +348.5-|
|                    |      |      +-------500.0-|      +------AT2G29500
|                    |      |      |             |
|                    |      |      |             +-------------AT1G59860
|                    |      |      |
|                    |      +265.8-|                    +------AT4G10250
|                    |             |             +487.2-|
|                    |             |      +258.0-|      +------Os04g36750
|             +495.5-|             |      |      |
|             |      |             +420.7-|      +-------------Os02g03570
|             |      |                    |
|             |      |                    +--------------------Os01g04340
|             |      |
|             |      |                                  +------Os01g04380
|             |      |                                  |
|             |      |                    +-------278.3-|------Os01g04370
|             |      |                    |             |
|      +498.3-|      |                    |             +------Os01g04360
|      |      |      +--------------470.5-|
|      |      |                           |             +------Os03g16040
|      |      |                           |      +328.6-|
|      |      |                           |      |      +------Os03g16020
|      |      |                           +428.8-|
+------|      |                                  |      +------Os03g16030
       |      |                                  +498.4-|
       |      |                                         +------Os03g15960
       |      |
       |      +------------------------------------------------AT5G59720
       |
       +-------------------------------------------------------AT3G46230
```

Heat shock protein 81 - NJ

```
                                                               +------Os01g04370
                     +-----------------------------------376.0-|
                     |                                         +------Os01g04380
                     |
                     |                                         +------AT4G10250
                     |                                  +500.0-|
                     |                                  |      +------Os04g36750
                     |                           +296.0-|
                     |                           |      |      +------Os02g03570
                     |                           |      +350.0-|
                     |                    +411.0-|             +------Os01g04340
                     |                    |      |
                     |                    |      |             +------AT1G07400
              +464.0-|                    |      |      +395.0-|
              |      |                    |      +496.0-|      +------AT2G29500
              |      |             +372.0-|             |
              |      |             |      |             +-------------AT1G59860
              |      |             |      |
              |      |             |      |                    +------AT1G53540
              |      |             |      |             +483.0-|
              |      |      +376.0-|      +-------482.0-|      +------AT3G46230
              |      |      |      |                    |
              |      |      |      |                    +-------------AT5G59720
              |      |      |      |
       +485.0-|      |      |      |             +--------------------pg3 3 40 3
       |      |      |      |      |             |
       |      |      +279.0-|      +-------495.0-|             +------Pt40082550
       |      |             |                    |      +500.0-|
       |      |             |                    +398.0-|      +------pg 5 110 2
       |      |             |                           |
       |      |             |                           +-------------Pt11338255
+------|      |             |
|      |      |             +-----------------------------------------Os01g04360
|      |      |
|      |      |                                                +------Os03g15960
|      |      +------------------------------------------498.0-|
|      |                                                       +------Os03g16030
|      |
|      +--------------------------------------------------------------Os03g16040
|
+---------------------------------------------------------------------Os03g16020
```

Heat shock protein 81 - PARS

```
+--------------------------------------------------------------AT1G53540
|
|                                                       +------pg 5 110 2
|                                                +500.0-|
|                                                |      +------Pt40082550
|                           +--------------421.6-|
|                           |                    |      +------pg3 3 40 3
|                           |                    +487.0-|
|                           |                           +------Pt11338255
|                           |
|                    +286.5-|                           +------AT1G07400
|                    |      |                    +348.5-|
|                    |      |      +-------500.0-|      +------AT2G29500
|                    |      |      |             |
|                    |      |      |             +-------------AT1G59860
|                    |      |      |
|                    |      +265.8-|                    +------AT4G10250
|                    |             |             +487.2-|
|                    |             |      +258.0-|      +------Os04g36750
|             +495.5-|             |      |      |
|             |      |             +420.7-|      +-------------Os02g03570
|             |      |                    |
|             |      |                    +--------------------Os01g04340
|             |      |
|             |      |                                  +------Os01g04380
|             |      |                                  |
|             |      |                    +-------278.3-|------Os01g04370
|             |      |                    |             |
|      +498.3-|      |                    |             +------Os01g04360
|      |      |      +--------------470.5-|
|      |      |                           |             +------Os03g16040
|      |      |                           |      +328.6-|
|      |      |                           |      |      +------Os03g16020
|      |      |                           +428.8-|
+------|      |                                  |      +------Os03g16030
       |      |                                  +498.4-|
       |      |                                         +------Os03g15960
       |      |
       |      +------------------------------------------------AT5G59720
       |
       +-------------------------------------------------------AT3G46230
```

Heavy-metal-associated domain-containing protein NJ

```
              +--------------------------------------------------------------AT4G39700
              |
              |                                                +-------------Pt33075 9
              |                           +--------------286.0-|
              |                           |                    |      +------Pt57907 13
              |                           |                    +497.0-|
              |                           |                           +------pg 5 87 13
              |                           |
              |                    +286.0-|                           +------Os03g06080
              |                    |      |                    +498.0-|
              |                    |      |      +-------259.0-|      +------Os04g17100
              |                    |      |      |             |
              |                    |      |      |             +-------------AT4G38580
              |                    |      |      |
       +493.0-|                    |      +213.0-|                    +------AT1G71050
       |      |                    |             |             +500.0-|
       |      |             +260.0-|             |      +497.0-|      +------AT1G22990
       |      |             |      |             |      |      |
       |      |             |      |             +380.0-|      +-------------AT5G17450
       |      |             |      |                    |
       |      |             |      |                    +--------------------Pt11558255
       |      |             |      |
       |      |             |      |                           +-------------Pp8042 14
       |      |      +369.0-|      |                    +500.0-|
       |      |      |      |      |                    |      |      +------Pt56228 15
       |      |      |      |      +--------------424.0-|      +499.0-|
+------|      |      |      |                           |             +------Pt56229 43
|      |      |      |      |                           |
|      |      +409.0-|      |                           +--------------------pg 11 52 1
|      |             |      |
|      |             |      +------------------------------------------------Os03g26650
|      |             |
|      |             |                                                +------Pt95482556
|      |             |                                         +480.0-|
|      |             +-----------------------------------366.0-|      +------Pt17516 11
|      |                                                       |
|      |                                                       +-------------AT4G08570
|      |
|      +---------------------------------------------------------------------Os09g20000
|
+----------------------------------------------------------------------------Os08g31140
```

Heavy-metal-associated domain-containing protein
PARS

```
                            +---------------------------pg 11 52 1
                            |
                            |                    +------AT1G22990
                            |             +500.0-|
                            |      +495.1-|      +------AT1G71050
                            |      |      |
                            +270.4-|      +-------------AT5G17450
                            |      |
                            |      +--------------------Pt11558255
                            |
                            |             +-------------Os03g26650
                            +-------263.5-|
                            |             |      +------pg 5 87 13
                     +462.6-|             +436.7-|
                     |      |                    +------Pt57907 13
                     |      |
                     |      +---------------------------AT4G38580
                     |      |
                     |      |                    +------Pp8042 14
                     |      |                    |
                     |      +--------------500.0-|------Pt56229 43
                     |      |                    |
              +391.4-|      |                    +------Pt56228 15
              |      |      |
              |      |      +---------------------------Pt33075 9
              |      |      |
              |      |      |                    +------Os03g06080
              |      |      +--------------500.0-|
              |      |                           +------Os04g17100
       +484.1-|      |
       |      |      |                           +------Pt95482556
       |      |      |                    +464.2-|
       |      |      +--------------415.1-|      +------Pt17516 11
+------|      |                           |
|      |      |                           +-------------AT4G08570
|      |      |
|      |      +-----------------------------------------AT4G39700
|      |
|      +------------------------------------------------Os09g20000
|
+-------------------------------------------------------Os08g31140
```

HD-ZIP3 - NJ

```
                     +-------------Pt73277
              +386.0-|
              |      |      +------pg7 128
              |      +500.0-|
       +253.0-|             +------Pt52171
       |      |
       |      |             +------Pt72482549
       |      +-------495.0-|
       |                    +------pg3 37
       |
       +---------------------------AT3G60390
       |
       |                    +------AT2G22800
       +--------------478.0-|
       |                    +------AT4G37790
       |
       +---------------------------AT4G16780
+------|
|      |                    +------Os03g12860
|      |                    |
|      +--------------491.0-|------Os08g36220
|      |                    |
|      |                    +------Os10g01470
|      |
|      +---------------------------AT2G44910
|      |
|      +---------------------------AT5G06710
|      |
|      +---------------------------Os04g46350
|      |
|      +---------------------------Pt13898254
|
+----------------------------------Os09g27450
```

HD-ZIP3 - PARS

```
                     +-------------Pt13898254
                     |
              +367.2-|-------------Pt73277
              |      |
              |      |      +------Pt52171
       +250.2-|      +496.6-|
       |      |             +------pg7 128
       |      |
       |      +--------------------AT5G06710
       |
       +---------------------------AT3G60390
       |
       +---------------------------Os03g12860
       |
       |                    +------Pt72482549
       +--------------453.5-|
       |                    +------pg3 37
+------|
|      |                    +------AT2G22800
|      +--------------494.1-|
|      |                    +------AT4G37790
|      |
|      +---------------------------Os09g27450
|      |
|      |                    +------Os04g46350
|      +--------------337.0-|
|      |                    +------AT4G16780
|      |
|      +---------------------------Os10g01470
|      |
|      +---------------------------AT2G44910
|
+----------------------------------Os08g36220
```

HD-ZIP5 - NJ

```
                                                 +------pg12 80 77
                                          +499.0-|
                                          |      +------Pt34899 38
                                   +498.0-|
                                   |      |      +------Pt56782550
                                   |      +500.0-|
                                   |             +------pg9 96 30
                            +308.0-|
                            |      |             +------Os02g49700
                            |      |      +500.0-|
                            |      |      |      +------Os08g32080
                     +421.0-|      +475.0-|
                     |      |             |      +------Os08g37580
                     |      |             +475.0-|
                     |      |                    +------Os09g29460
              +327.0-|      |
              |      |      +---------------------------AT3G01470
              |      |
              |      |                           +------Pt56193 17
       +500.0-|      +---------------------310.0-|
       |      |                                  +------AT5G65310
       |      |
+------|      |                                  +------Pt3682 37
|      |      +----------------------------499.0-|
|      |                                         +------Pt72682550
|      |
|      +------------------------------------------------AT4G40060
|
+-------------------------------------------------------AT2G22430
```

HD-ZIP5 - PARS

```
                                   +------pg9 96 30
                     +-------500.0-|
                     |             +------Pt56782550
                     |
                     |             +------Pt34899 38
                     +-------492.7-|
              +368.6-|             +------pg12 80 77
              |      |
              |      |             +------Os08g37580
              |      |      +463.4-|
              |      |      |      +------Os09g29460
       +281.0-|      +267.1-|
       |      |             |      +------Os08g32080
       |      |             +496.0-|
       |      |                    +------Os02g49700
       |      |
       |      +---------------------------AT3G01470
       |
+------|                           +------Pt72682550
|      |                    +444.0-|
|      |                    |      +------Pt3682 37
|      +--------------301.0-|
|      |                    |      +------AT2G22430
|      |                    +414.3-|
|      |                           +------AT4G40060
|      |
|      +----------------------------------AT5G65310
|
+-----------------------------------------Pt56193 17
```

HMG alpha - NJ

```
                                          +------pg4 68 35
                                          |
                                   +416.0-|------Pt64082547
                                   |      |
                            +253.0-|      +------Pp3454 15
                            |      |
                            |      |      +------AT3G51880
                     +432.0-|      +267.0-|
                     |      |             +------Os06g51220
                     |      |
              +432.0-|      +--------------------Os09g37910
              |      |
              |      |                    +------AT1G20696
       +494.0-|      +--------------413.0-|
       |      |                           +------AT1G20693
       |      |
+------|      |                           +------Pp1113515
|      |      +---------------------491.0-|
|      |                                  +------Pt11928255
|      |
|      +-----------------------------------------pg1 25 28
|
+------------------------------------------------pg1 28 19
```

HMG alpha - PARS

```
                                 +------Os06g51220
                                 |
                          +373.5-|------Pt11928255
                          |      |
                          |      +------Pp1113515
                   +373.5-|
                   |      +-------------pg1 28 19
                   |      |
            +390.4-|      +-------------pg1 25 28
            |      |
            |      |             +------AT1G20693
     +416.2-|      +-------494.4-|
     |      |                    +------AT1G20696
     |      |
     |      +---------------------------Os09g37910
+------|
|      +----------------------------------pg4 68 35
|      |
|      +----------------------------------Pt64082547
|      |
|      +----------------------------------AT3G51880
|
+-----------------------------------------Pp3454 15
```

Histone H3 - NJ

```
              +------AT5G10390
              |
              +------AT5G10400
              |
              +------Pt75489 8
              |
              +------AT5G65360
              |
              +------Pt99 25
              |
              +------Os06g06510
              |
              +------Os05g36280
              |
              +------Os04g34240
       +489.0-|
       |      +------Pt98 8
       |      |
       |      +------AT3G27360
       |      |
       |      +------pg9 76 9
       |      |
       |      +------Pt74310 8
       |      |
       |      +------Os11g05730
       |      |
       |      +------Os06g06460
       |      |
       |      +------AT1G09200
+------|      |
|      |      +------pg6 91 41
|      |
|      +-------------AT4G40030
|      |
|      +-------------AT5G10980
|      |
|      +-------------pg2 122 38
|      |
|      +-------------Pt70541 12
|      |
|      +-------------Os06g04030
|      |
|      +-------------AT4G40040
|      |
|      +-------------Os03g27310
|
+--------------------Pp713515 1
```

Histone H3 - PARS

```
                  +------Os06g06460
                  |
                  +------AT5G10390
                  |
                  +------pg6 91 41
                  |
                  +------Os06g06510
                  |
                  +------Os11g05730
                  |
                  +------Pt99 25
                  |
                  +------Pt74310 8
                  |
                  +------Pt75489 8
           +489.0-|
           |      +------AT5G65360
           |      |
           |      +------Os04g34240
           |      |
           |      +------Pt98 8
           |      |
           |      +------Os05g36280
           |      |
           |      +------AT1G09200
           |      |
           |      +------AT3G27360
           |      |
           |      +------pg9 76 9
    +------|      |
    |      |      +------AT5G10400
    |      |
    |      +-------------Os06g04030
    |      |
    |      +-------------Os03g27310
    |      |
    |      +-------------AT4G40030
    |      |
    |      +-------------pg2 122 38
    |      |
    |      +-------------Pt70541 12
    |      |
    |      +-------------Pp713515 1
    |      |
    |      +-------------AT4G40040
    |
    +--------------------AT5G10980
```

Hsp70 - NJ

```
                                                 +------AT5G02500.
                                          +482.0-|
                     +--------------435.0-|      +------AT5G02490.
                     |                    |
                     |                    +-------------AT3G09440.
                     |
                     |                           +------pg3 314037
                     |                    +478.0-|
                     |             +500.0-|      +------Pt10928255
                     |             |      |
                     |      +431.0-|      +-------------Pp5695 35
                     |      |      |
                     +296.0-|      +--------------------AT1G16030.
                     |      |
              +279.0-|      +---------------------------Os03g16920
              |      |
              |      |                           +------Pp6513517
              |      |                    +496.0-|
              |      +--------------500.0-|      +------Pt81082556
              |      |                    |
              |      |                    +-------------pg 5 112 104
              |      |
       +282.0-|      +----------------------------------Pt97582550
       |      |      |
       |      |      +----------------------------------AT3G12580.
       |      |      |
       |      |      +----------------------------------pg 7 121 41
       |      |
+------|      +-----------------------------------------Os03g16860
|      |      |
|      |      |                                  +------Os01g62290
|      |      +----------------------------482.0-|
|      |                                         +------Os05g38530
|      |
|      +------------------------------------------------Os03g60620
|
+-------------------------------------------------------Os11g47760
```

Hsp70 - PARS

```
              +-----------------------------------------Os03g16860
              |
              |                                  +------Os01g62290
              +----------------------------397.3-|
              |                                  +------Os05g38530
              |
              |                                  +------pg3 314037
              |                           +500.0-|
              |                    +500.0-|      +------pg7 12141
              |                    |      |
              |             +415.1-|      +-------------Pp5695 35
              |             |      |
              |      +417.4-|      +--------------------Pt10928255
              |      |      |
       +335.7-|-263.7-|      +---------------------------AT1G16030.
       |      |      |
       |      |      +----------------------------------Os03g16920
       |      |
       |      +-----------------------------------------Os11g47760
       |      |
       |      +-----------------------------------------Os03g60620
       |      |
+------|      |                                  +------AT5G02490.
|      |      |                           +369.2-|
|      |      +---------------------471.0-|      +------AT5G02500.
|      |      |                           |
|      |      |                           +-------------AT3G09440.
|      |      |
|      |      +-----------------------------------------AT3G12580.
|      |
|      |                                  +-------------pg5 112104
|      +----------------------------500.0-|
|                                         |      +------Pt81082556
|                                         +299.6-|
|                                                +------Pp6513517
|
+-------------------------------------------------------Pt97582550
```

Integral membrane Yip1 family - NJ

```
                                          +------AT4G30260
       +----------------------------500.0-|
       |                                  +------AT2G18840
       |
       |                                  +------AT3G52760
       |                           +304.0-|
       |                           |      +------AT2G36300
       |                    +301.0-|
+------|                    |      |      +------AT5G38320
|      |                    |      +223.0-|
|      |             +500.0-|             +------Os03g17990
|      |             |      |
|      |             |      |             +------Pt69982549
|      |      +363.0-|      +-------477.0-|
|      |      |      |                    +------pg10 51 29
|      |      |      |
|      +492.0-|      +---------------------------Os05g36150
|             |
|             |                           +------pg3 46 32
|             +---------------------308.0-|
|                                         +------Pt9782552
|
+------------------------------------------------Os01g64760
```

Integral membrane Yip1 family - PARS

```
                                                 +------AT4G30260
                     +---------------------500.0-|
                     |                           +------AT2G18840
                     |
                     |                           +------Pt69982549
                     |                    +500.0-|
              +435.5-|             +361.8-|      +------pg10 51 29
              |      |             |      |
              |      |      +480.8-|      +-------------Os03g17990
              |      |      |      |
              |      |      |      |             +------AT3G52760
       +372.8-|      +500.0-|      +-------483.0-|
       |      |             |                    +------AT2G36300
       |      |             |
       |      |             +---------------------------AT5G38320
+------|      |
|      |      |                                  +------pg3 46 32
|      |      +----------------------------499.0-|
|      |                                         +------Pt9782552
|      |
|      +------------------------------------------------Os01g64760
|
+-------------------------------------------------------Os05g36150
```

KH domain-containing protein - NJ

```
                                          +--------------------Os02g10080
                                          |
                                          |             +------Pp11173 9
                                          |      +438.0-|
                                   +362.0-|      |      +------Pt27884 28
                                   |      |      |
                                   |      |      |      +------AT3G12130
                                   |      |      +263.0-|
                                   |      |      |      +------Os04g57010
                                   |      +398.0-|
                                   |             +-------------Os06g41384
                            +490.0-|             |
                            |      |             +-------------Pt24697 27
                            |      |             |
                            |      |             +-------------Pt21092 28
                            |      |             |
                            |      |             +-------------pg 4 54 64
                            |      |
                            |      +---------------------------AT5G06770
                            |
                            |             +--------------------Pt17996 7
                     +499.0-|             |
                     |      |      +498.0-|             +------pg9 65 14
                     |      |      |      |      +273.0-|
                     |      |      |      |      |      +------Pp8013516
                     |      |      |      +354.0-|
                     |      |      |             +-------------Pt44182547
                     |      |      |             |
                     |      |      |             +-------------Pt43582553
                     |      |      |
                     |      |      |                    +------Pp4809 14
                     |      +470.0-|--------------450.0-|
                     |             |                    +------Pt73755 31
              +297.0-|             |
              |      |             |                    +------AT1G51580
              |      |             +--------------315.0-|
              |      |             |                    +------Os09g32300
              |      |             |
              |      |             +---------------------------Pt5082555
              |      |             |
              |      |             +---------------------------pg2 37 14
       +305.0-|      |
       |      |      +-----------------------------------------AT5G56140
       |      |      |
       |      |      +-----------------------------------------AT4G26480
       |      |      |
       |      |      +-----------------------------------------Os02g49080
       |      |
       |      |                                         +------Pt34582556
       |      |                                         |
       |      +-----------------------------------498.0-|------Pt34582555
       |                                                |
       |                                                +------Pp12805 15
       |
       |                                                +------Pt48782547
       +------------------------------------------500.0-|
       |                                                +------Pp6389 27
+------|
|      |                                                +------Os01g60260
|      |                                         +497.0-|
|      +-----------------------------------465.0-|      +------Os05g40290
|      |                                         |
|      |                                         +-------------Os07g12490
|      |
|      |                                                +------AT3G08620
|      +------------------------------------------458.0-|
|      |                                                +------Os03g60110
|      |
|      |                                                +------Os01g66300
|      +------------------------------------------286.0-|
|      |                                                +------Os05g34670
|      |
|      +-------------------------------------------------------pg 1 44 14
|      |
|      +-------------------------------------------------------AT1G09660
|
+--------------------------------------------------------------AT2G38610
```

KH domain-containing protein - PARS

```
                                                                             +------Os01g60260
                                                                      +483.4-|
                                                               +455.5-|      +------Os05g40290
                                                               |      |
                                                        +319.0-|      +-------------Os07g12490
                                                        |      |
                                                 +257.3-|      +--------------------Os03g60110
                                                 |      |
                                                 |      +---------------------------AT3G08620
                                                 |
                                                 |                           +------Os02g49080
                                                 |                           |
                                                 +---------------------311.2-|------AT4G26480
                                                 |                           |
                                                 |                           +------AT5G56140
                                                 |
                                                 +----------------------------------Os01g66300
                                                 |
                                                 |                           +------Pt34582556
                                          +261.6-|                    +347.2-|
                                          |      +--------------448.0-|      +------Pp12805 15
                                          |      |                    |
                                          |      |                    +-------------Pt34582555
                                          |      |
                                          |      +----------------------------------AT1G09660
                                          |      |
                                   +497.9-|      |                           +------Pt48782547
                                   |      |      +---------------------499.7-|
                                   |      |      |                           +------Pp6389 27
                                   |      |      |
                                   |      |      +----------------------------------pg 1 44 14
                                   |      |      |
                                   |      |      +----------------------------------Os05g34670
                                   |      |
                                   |      +-----------------------------------------AT2G38610
                                   |
                            +488.9-|                                         +------AT1G51580
                            |      |                           +-------307.8-|
                            |      |                           |             +------Os09g32300
                            |      |                           |
                            |      |                           |             +------Pp8013516
                            |      |                           |             |
                            |      |                           |             +------Pt43582553
                            |      |                           |      +448.9-|
                            |      |                           |      |      +------Pt44182547
                            |      |                           +490.9-|      |
                            |      +---------------------334.9-|      |      +------Pt17996 7
                     +340.9-|                                  |      |
                     |      |                                  |      +-------------pg9 65 14
                     |      |                                  |
                     |      |                                  +--------------------Pt5082555
                     |      |                                  |
                     |      |                                  +--------------------pg2 37 14
              +413.1-|      |                                  |
              |      |      |                                  |             +------Pt73755 31
              |      |      |                                  +-------499.9-|
              |      |      |                                                +------Pp4809 14
              |      |      |
              |      |      +-------------------------------------------------------Pt21092 28
              |      |
              |      +--------------------------------------------------------------Os06g41384
              |
       +307.6-|                                                              +------AT5G06770
       |      +--------------------------------------------------------490.9-|
       |      |                                                              +------AT3G12130
       |      |
       |      +---------------------------------------------------------------------Pt24697 27
       |      |
+------|      +---------------------------------------------------------------------pg 4 54 64
|      |      |
|      |      |                                                              +------Pt27884 28
|      |      +--------------------------------------------------------487.1-|
|      |                                                                     +------Pp11173 9
|      |
|      +----------------------------------------------------------------------------Os02g10080
|
+-----------------------------------------------------------------------------------Os04g57010
```

Kinase - NJ

```
                                   +------Os01g21960
                            +397.0-|
                            |      +------Os12g01740
                            |
                            |      +------Pt26667 24
                            +500.0-|
                     +355.0-|      +------Pt29068 20
                     |      |
                     |      |      +------Pt4286 25
                     |      +500.0-|
                     |      |      +------pg 6 56 21
                     |      |
              +455.0-|      +-------------pg 10 113
              |      |
              |      |             +------AT1G09440
              |      +-------500.0-|
              |      |             +------AT1G56720
       +------|      |
       |      |      |             +------Os01g44110
       |      |      +-------480.0-|
       |      |                    +------Os10g35450
       |      |
       |      +-------------------------AT3G59110
       |
       +--------------------------------AT2G42960
```

kinase - PARS

```
                                          +------Os10g35450
                                   +403.9-|
                                   |      +------Os01g44110
                            +379.6-|
                            |      |      +------pg 6 56 21
                            |      +500.0-|
                     +372.4-|             +------Pt4286 25
                     |      |
                     |      |             +------Pt26667 24
                     |      |      +500.0-|
              +413.4-|      +467.9-|      +------Pt29068 20
              |      |             |
              |      |             +-------------pg 10 113
              |      |
       +499.2-|      |                    +------Os12g01740
       |      |      +--------------488.0-|
       |      |                           +------Os01g21960
       |      |
+------|      |                           +------AT3G59110
|      |      +---------------------491.5-|
|      |                                  +------AT2G42960
|      |
|      +-----------------------------------------AT1G56720
|
+------------------------------------------------AT1G09440
```

Kinase - NJ

```
                             +--------------------AT5G59700
                            |
              +-------383.0-|             +------Os05g06990
              |             |      +410.0-|
              |             +361.0-|      +------Os06g22810
              |                    |
              |                    +-------------AT3G46290
              |
              |                           +------Pt13240 22
              |                    +440.0-|
              |             +290.0-|      +------Pp7668 9
              |             |      |
              |      +347.0-|      +-------------AT5G61350
              |      |      |
              +382.0-|      +--------------------pg7 93
              |      |
              |      +---------------------------AT2G21480
              |
              |                           +------pg7 83 16
              |                    +500.0-|
              |                    |      +------Pt4792 25
              |                    |
              |                    |      +------Os06g41980
              |                    +304.0-|
              |                    |      +------Os02g09960
              |                    |
              |                    +-------------AT2G33580
       +359.0-|             +266.0-|
       |      |             |      |      +------Os01g53840
       |      |             |      +499.0-|
       |      |             |      |      +------AT1G51940
       |      |             |      |
       |      +-------479.0-|      +-------------Os03g13080
       |      |             |      |
       |      |             |      +-------------AT3G21630
       |      |             |      |
       |      |             |      +-------------Os02g45750
       |      |             |
       |      |             +--------------------AT4G02010
       |      |
+------|      |                           +------Os01g56330
|      |      +---------------------500.0-|
|      |      |                           +------Os03g21540
|      |      |
|      |      +----------------------------------AT5G28680
|      |      |
|      |      |                           +------pg2 75 8
|      |      +---------------------488.0-|
|      |      |                           +------Pt26467 32
|      |      |
|      |      +----------------------------------AT3G51550
|      |      |
|      |      +----------------------------------AT5G54380
|      |
|      +-----------------------------------------Os05g25450
|
+------------------------------------------------Os05g20150
```

Kinase - PARS

```
                     +--------------------Os03g13080
                     |
                     |             +------Os06g41980
                     +-------423.5-|
                     |             +------Os02g09960
                     |
                     |             +------pg7 83 16
                     |      +500.0-|
                     |      |      +------Pt4792 25
                     +276.0-|
              +477.4-|      |      +------Os01g53840
              |      |      +497.9-|
              |      |             +------AT1G51940
              |      |
              |      +--------------------AT3G21630
              |      |
              |      +--------------------Os02g45750
              |      |
              |      +--------------------AT4G02010
              |      |
              |      +--------------------AT2G33580
              |
              |                    +------Pp7668 9
              +--------------493.8-|
              |                    +------Pt13240 22
              |
              +---------------------------Pt26467 32
              |
              |                    +------AT5G54380
              +--------------270.6-|
              |                    +------AT5G28680
       +294.9-|
       |      |                    +------AT3G46290
       |      +--------------306.0-|
       |      |                    +------AT5G59700
       |      |
       |      +---------------------------AT5G61350
       |      |
       |      |                    +------Os01g56330
       |      +--------------419.9-|
       |      |                    +------Os03g21540
       |      |
+------|      +---------------------------AT3G51550
|      |      |
|      |      +---------------------------pg2 75 8
|      |      |
|      |      +---------------------------Os06g22810
|      |      |
|      |      +---------------------------pg7 93
|      |      |
|      |      +---------------------------Os05g06990
|      |      |
|      |      +---------------------------AT2G21480
|      |
|      +----------------------------------Os05g25450
|
+-----------------------------------------Os05g20150
```

Kinase - NJ

```
                                                                             +------Os05g30870
                                                                      +500.0-|
                                                                      |      +------Os01g71000
                                                                      |
                                                                      |      +------Pt2162 42
                                                               +293.0-|-437.0-|
                                                               |      |      +------AT5G13160
                                                               |      |
                                                               |      |      +------Os07g47270
                                                               |      +401.0-|
                                          +--------------461.0-|             +------AT3G20530
                                          |                    |
                                          |                    +--------------------AT5G02800
                                          |                    |
                                          |                    +--------------------Os02g30900
                                          |                    |
                                          |                    +--------------------AT1G20650
                                          |
                                          |                                  +------Os12g44090
                                          |                    +-------500.0-|
                                   +500.0-|                    |             +------AT5G62710
                                   |      |                    |
                                   |      |             +410.0-|             +------AT2G31880
                                   |      |             |      |      +500.0-|
                                   |      |             |      |      |      +------Os06g18000
                                   |      |             |      +485.0-|
                                   |      |      +500.0-|             |      +------Pt22544 25
                                   |      |      |      |             +500.0-|
                                   |      |      |      |                    +------pg1 103 23
                            +385.0-|      |      |      |
                            |      |      +498.0-|      +---------------------------AT5G63930
                            |      |             |
                            |      |             |                           +------AT1G24030
                            |      |             |                    +500.0-|
                            |      |             +--------------497.0-|      +------Os09g20880
                     +479.0-|      |                                  |
                     |      |      |                                  +-------------pg3 6 35
                     |      |      |
                     |      |      +------------------------------------------------AT5G15080
                     |      |
              +341.0-|      |                                                +------AT2G07180
              |      |      +------------------------------------------500.0-|
              |      |                                                       +------Os06g45280
              |      |
       +497.0-|      |                                                       +------AT5G56460
       |      |      +-------------------------------------------------500.0-|
       |      |                                                              +------Os03g06330
+------|      |
|      |      +---------------------------------------------------------------------AT2G05940
|      |
|      |                                                              +-------------AT5G01020
|      +--------------------------------------------------------500.0-|
|                                                                     |      +------Os11g02830
|                                                                     +500.0-|
|                                                                            +------Os12g02810
|
+-----------------------------------------------------------------------------------pg11 90 59
```

Kinase - PARS

```
                                                                             +------Os05g30870
                                                                      +445.2-|
                                                                      |      +------Os01g71000
                                                                      |
                                                                      +-------------AT5G02800
                                                                      |
                                                                      +-------------AT3G20530
                                                                      |
                                                               +417.1-|-------------Os02g30900
                                                               |      |
                                                               |      +-------------AT1G20650
                                                               |      |
                                                               |      +-------------Pt2162 42
                                                 +-------316.9-|      |
                                                 |             |      +-------------AT5G13160
                                                 |             |      |
                                                 |             |      +-------------Os07g47270
                                                 |             |
                                                 |             |      +-------------pg3 6 35
                                                 |             +372.3-|
                                                 |                    |      +------AT1G24030
                                                 |                    +452.5-|
                                                 |                           +------Os09g20880
                                          +500.0-|
                                          |      |                           +------AT2G31880
                                          |      |                    +500.0-|
                                          |      |                    |      +------Os06g18000
                                          |      |             +452.0-|
                                          |      |             |      |      +------Pt22544 25
                                          |      |             |      +500.0-|
                                          |      |      +320.9-|             +------pg1 103 23
                                          |      |      |      |
                                   +382.4-|      |      |      |             +------Os12g44090
                                   |      |      +500.0-|      +-------500.0-|
                                   |      |             |                    +------AT5G62710
                                   |      |             |
                                   |      |             +---------------------------AT5G63930
                                   |      |
                            +307.5-|      +-----------------------------------------AT5G15080
                            |      |      |
                            |      |      |                                  +------AT2G07180
                            |      |      +----------------------------500.0-|
                            |      |                                         +------Os06g45280
                     +478.6-|      |
                     |      |      |                                         +------Os03g06330
                     |      |      +-----------------------------------500.0-|
              +477.1-|      |                                                +------AT5G56460
              |      |      |
              |      |      +-------------------------------------------------------AT2G05940
       +500.0-|      |
       |      |      +--------------------------------------------------------------pg11 90 59
+------|      |
|      |      +---------------------------------------------------------------------AT5G01020
|      |
|      +----------------------------------------------------------------------------Os11g02830
|
+-----------------------------------------------------------------------------------Os12g02810
```

Knox - NJ

```
                                                 +------AT4G08150
              +----------------------------258.0-|
              |                                  +------Os03g51690
              |
              |                                  +------Pt76992 38
              |                           +347.0-|
              |                    +500.0-|      +------pg3 33
              |                    |      |
              |             +303.0-|      +-------------pg3 32
              |             |      |
       +258.0-|             |      |             +------pg2 103
       |      |             |      +-------500.0-|
       |      |             |                    +------Pt58750 56
       |      |      +468.0-|
       |      |      |      |                    +------Pt25366 46
       |      |      |      |      +-------500.0-|
       |      |      |      |      |             +------pg6 30
       |      |      |      |      |
       |      |      |      +424.0-|             +------Os01g19694
+------|      +460.0-|             |      +500.0-|
|      |             |             |      |      +------Os05g03884
|      |             |             +493.0-|
|      |             |                    |      +------AT1G70510
|      |             |                    +500.0-|
|      |             |                           +------AT1G23380
|      |             |
|      |             +----------------------------------AT1G62360
|      |
|      +------------------------------------------------Os07g03770
|
+-------------------------------------------------------Os03g51710
```

Knox - PARS

```
+--------------------------------------------------------------Os03g51690
|
|                                                       +------Pt76992 38
|                                                +326.9-|
|                                         +499.0-|      +------pg3 33
|                                         |      |
|                                  +337.8-|      +-------------pg3 32
|                                  |      |
|                                  |      |             +------pg2 103
|                                  |      +-------500.0-|
|                                  |                    +------Pt58750 56
|                           +352.4-|
|                           |      |                    +------Os01g19694
|                           |      |      +-------500.0-|
|                           |      |      |             +------Os05g03884
|                           |      |      |
|                           |      +405.0-|             +------AT1G70510
|                    +340.0-|             |      +500.0-|
|                    |      |             |      |      +------AT1G23380
|                    |      |             +253.3-|
|                    |      |                    |      +------pg6 30
|             +396.8-|      |                    +500.0-|
|             |      |      |                           +------Pt25366 46
|             |      |      |
|      +325.3-|      |      +----------------------------------AT1G62360
|      |      |      |
|      |      |      +-----------------------------------------Os03g51710
+------|      |
       |      +------------------------------------------------Os07g03770
       |
       +-------------------------------------------------------AT4G08150
```

Laccase - NJ

```
                                                                             +------Os05g30870
                                                                      +445.2-|
                                                                      |      +------Os01g71000
                                                                      |
                                                                      +-------------AT5G02800
                                                                      |
                                                                      +-------------AT3G20530
                                                                      |
                                                               +417.1-|-------------Os02g30900
                                                               |      |
                                                               |      +-------------AT1G20650
                                                               |      |
                                                               |      +-------------Pt2162 42
                                                 +-------316.9-|      |
                                                 |             |      +-------------AT5G13160
                                                 |             |      |
                                                 |             |      +-------------Os07g47270
                                                 |             |
                                                 |             |      +-------------pg3 6 35
                                                 |             +372.3-|
                                                 |                    |      +------AT1G24030
                                                 |                    +452.5-|
                                                 |                           +------Os09g20880
                                          +500.0-|
                                          |      |                           +------AT2G31880
                                          |      |                    +500.0-|
                                          |      |                    |      +------Os06g18000
                                          |      |             +452.0-|
                                          |      |             |      |      +------Pt22544 25
                                          |      |             |      +500.0-|
                                          |      |      +320.9-|             +------pg1 103 23
                                          |      |      |      |
                                   +382.4-|      |      |      |             +------Os12g44090
                                   |      |      +500.0-|      +-------500.0-|
                                   |      |             |                    +------AT5G62710
                                   |      |             |
                                   |      |             +---------------------------AT5G63930
                                   |      |
                            +307.5-|      +-----------------------------------------AT5G15080
                            |      |      |
                            |      |      |                                  +------AT2G07180
                            |      |      +---------------------------500.0-|
                            |      |                                         +------Os06g45280
                     +478.6-|      |
                     |      |      |                                         +------Os03g06330
                     |      |      +----------------------------------500.0-|
              +477.1-|      |                                                +------AT5G56460
              |      |      |
              |      |      +-------------------------------------------------------AT2G05940
       +500.0-|      |
       |      |      +--------------------------------------------------------------pg11 90 59
+------|      |
|      |      +---------------------------------------------------------------------AT5G01020
|      |
|      +----------------------------------------------------------------------------Os11g02830
|
+-----------------------------------------------------------------------------------Os12g02810
```

Laccase - PARS

```
                                                        +-------------Os01g62480
                                                 +351.6-|
                                                 |      |      +------Os05g38420
                                                 |      +457.0-|
                                          +255.2-|             +------Os01g62490
                                          |      |
                                          |      +--------------------AT2G29130
                                   +300.0-|      |
                                   |      |      +--------------------Os03g16610
                                   |      |
                                   |      +---------------------------AT5G60020
                                   |
                            +500.0-|                    +-------------pg 4 2 39
                            |      |             +500.0-|
                            |      |             |      |      +------Pt11718254
                            |      |             |      +375.9-|
                            |      |             |             +------Pt46110 38
                            |      +-------372.6-|
                     +376.2-|                    +--------------------Pt45182551
                     |      |                    |
                     |      |                    |             +------Pt7682547
                     |      |                    +-------413.5-|
                     |      |                                  +------Pt79882556
                     |      |
                     |      +-----------------------------------------Os01g44330
              +406.0-|
              |      |                                         +------Pt1019 33
              |      |                                  +392.0-|
              |      +----------------------------294.4-|      +------pg6 5put 2
              |      |                                  |
       +355.3-|      |                                  +-------------Pt39552 38
       |      |      |
       |      |      +------------------------------------------------AT5G03260
       |      |
+------|      +-------------------------------------------------------Os11g48060
|      |      |
|      |      +-------------------------------------------------------AT5G58910
|      |
|      +--------------------------------------------------------------AT2G38080
|
+---------------------------------------------------------------------AT5G01190
```

L-ascorbate peroxidase - NJ

```
                                                               +------Pt66312 19
              +------------------------------------------285.0-|
              |                                                +------AT3G09640
              |
              |                                                +------Pt82982547
              |                                         +500.0-|
              |      +----------------------------418.0-|      +------Pp2440 19
       +500.0-|      |                                  |
       |      |      |                                  +-------------AT1G07890
       |      |      |
       |      |      |                                         +------Pp417 23
       |      |      |                                  +271.0-|
       |      |      |                           +500.0-|      +------Pt81722 29
       |      +322.0-|                           |      |
       |             |                    +247.0-|      +-------------pg7 38
       |             |                    |      |
       |             |             +209.0-|      +--------------------AT4G35970
       |             |             |      |
+------|             |      +500.0-|      +---------------------------AT4G35000
|      |             |      |      |
|      |             |      |      |                           +------Os08g43560
|      |             +251.0-|      +---------------------381.0-|
|      |                    |                                  +------Os04g14680
|      |                    |
|      |                    |                                  +------Os03g17690
|      |                    +----------------------------464.0-|
|      |                                                       +------Os07g49400
|      |
|      |                                                       +------Pp3280 16
|      +-------------------------------------------------483.0-|
|                                                              +------Pt95182555
|
+---------------------------------------------------------------------pg8 166
```

L-ascorbate peroxidase - PARS

```
+-------------------------------------------------------pg8 166
|
|                                                +------pg7 38
|                                                |
|                                         +498.6-|------Pt81722 29
|                                         |      |
|                                         |      +------Pp417 23
|                                         |
|                                  +500.0-|-------------AT4G35970
|                                  |      |
|                                  |      +-------------AT4G35000
|                                  |      |
|                           +392.8-|      |      +------Os04g14680
|                           |      |      +350.7-|
|                           |      |             +------Os08g43560
|                           |      |
|                    +349.8-|      |             +------Os03g17690
|                    |      |      +-------495.4-|
|                    |      |                    +------Os07g49400
|             +329.3-|      |
|             |      |      +---------------------------Pt66312 19
|             |      |
|      +494.0-|      +----------------------------------AT3G09640
|      |      |
|      |      |                                  +------Pp2440 19
|      |      |                           +500.0-|
+------|      +---------------------444.0-|      +------Pt82982547
       |                                  |
       |                                  +-------------AT1G07890
       |
       |                                         +------Pp3280 16
       +-----------------------------------280.0-|
                                                 +------Pt95182555
```

LEUNIG - NJ

```
                                                         +------pg5 44 29
                                          +-------500.0-|
                                          |             +------Pt37829 14
                                   +285.0-|
                                   |      |      +-------------pg12 68 31
                                   |      +498.0-|
                                   |             |      +------Pt57126 33
                            +444.0-|             +300.0-|
                            |      |                    +------Pp1382 12
                            |      |
                            |      |                    +------Pt1982555
                     +363.0-|      +-------------376.0-|
                     |      |                           +------pg6 63 11
                     |      |
                     |      |                           +------Os01g08190
              +500.0-|      +--------------------500.0-|
              |      |                                  +------Os01g42260
              |      |
       +500.0-|      |                                  +------Pt80511 16
       |      |      +---------------------------495.0-|
       |      |                                         +------pg5 21 21
+------|      |
|      |      +------------------------------------------------AT2G32700.
|      |
|      +-------------------------------------------------------Os04g43130
|
+--------------------------------------------------------------Os02g56880
```

LEUNIG - PARS

```
                                          +------pg5 44 29
                            +-------500.1-|
                            |             +------Pt37829 14
                            |
                            |             +------Os01g42260
                            |      +497.5-|
                            |      |      +------Os01g08190
                            +354.3-|
                     +421.2-|      |      +------pg12 68 31
                     |      |      |      |
                     |      |      +328.6-|------Pp1382 12
                     |      |             |
                     |      |             +------Pt57126 33
              +473.6-|      |
              |      |      +--------------------Pt1982555
              |      |      |
              |      |      +--------------------pg6 63 11
       +420.1-|      |
       |      |      |                    +------pg5 21 21
       |      |      +--------------458.8-|
+------|      |                           +------Pt80511 16
|      |      |
|      |      +----------------------------------AT2G32700.
|      |
|      +-----------------------------------------Os02g56880
|
+------------------------------------------------Os04g43130
```

LIM - NJ

```
                                                         +------------Os03g15940
                                          +-------285.0-|
                                          |             |      +------AT2G39900
                                          |             +500.0-|
                                          |                    +------AT3G55770
                                   +271.0-|
                                   |      |             +-------------AT1G01780
                                   |      |      +500.0-|
                                   |      |      |      |      +------AT2G45800
                                   |      |      |      +265.0-|
                                   |      +484.0-|             +------AT3G61230
                            +362.0-|             |
                            |      |             |      +-------------Os10g35930
                            |      |             +495.0-|
                            |      |                    |      +------Os04g45010
                            |      |                    +497.0-|
                     +427.0-|      |                           +------Os02g42820
                     |      |      |
                     |      |      +----------------------------------Os06g13030
                     |      |
              +500.0-|      |                                  +------Os12g32620
              |      |      +----------------------------500.0-|
              |      |                                         +------AT1G10200
              |      |
              |      |                                  +-------------pg3 2 52
       +294.0-|      +----------------------------429.0-|
       |      |                                         |      +------Pt43982552
       |      |                                         +495.0-|
+------|      |                                                +------Pp11159 14
|      |      |
|      |      +-------------------------------------------------------Pt76369 12
|      |
|      +--------------------------------------------------------------Pp2204 10
|
+---------------------------------------------------------------------pg9 131 43
```

LIM - PARS

```
                                                        +------Pp2204 10
                                                        |
                     +----------------------------498.3-|------pg9 131 43
                     |                                  |
                     |                                  +------Pt76369 12
                     |
              +266.3-|      +----------------------------------Os06g13030
              |      |      |
              |      |      |                           +------AT2G45800
              |      |      |                           |
              |      |      |             +-------489.6-|------AT1G01780
              |      +352.4-|             |             |
              |             |      +493.7-|             +------AT3G61230
              |             |      |      |
              |             |      |      |      +-------------Os10g35930
              |             |      |      +492.5-|
              |             |      |             |      +------Os04g45010
       +491.5-|             +290.3-|             +499.5-|
       |      |                    |                    +------Os02g42820
       |      |                    |
       |      |                    |                    +------AT3G55770
       |      |                    |             +476.7-|
       |      |                    +-------364.3-|      +------AT2G39900
       |      |                                  |
+------|      |                                  +-------------Os03g15940
|      |      |
|      |      +------------------------------------------------pg3 2 52
|      |      |
|      |      |                                         +------Pt43982552
|      |      +-----------------------------------283.1-|
|      |                                                +------Pp11159 14
|      |
|      +-------------------------------------------------------AT1G10200
|
+--------------------------------------------------------------Os12g32620
```

LOB domain protein - NJ

```
                                   +--------------------Pt96082554
                                   |
                            +500.0-|             +------Pt34310 12
                            |      |      +353.0-|
                            |      +498.0-|      +------pg 4 41 33
                     +306.0-|             |
                     |      |             +-------------Pt27489 11
                     |      |
              +368.0-|      |                    +------AT2G28500
              |      |      +--------------500.0-|
              |      |                           +------AT1G07900
              |      |
              |      +----------------------------------AT2G30130
              |
              |                                  +------Pt7277 16
              |                           +270.0-|
              |             +-------492.0-|      +------Pt16032 32
       +293.0-|             |             |
       |      |             |             +-------------pg 11 126
       |      |             |
       |      |      +496.0-|                    +------Pt24088 25
       |      |      |      |             +499.0-|
       |      |      |      |      +209.0-|      +------Pp4027 11
       |      |      |      |      |      |
       |      |      |      |      |      +-------------Os05g07270
       |      |      |      +-92.0-|
+------|      +442.0-|             |             +------AT2G30340
|      |             |             |      +408.0-|
|      |             |             +176.0-|      +------AT2G40470
|      |             |                    |
|      |             |                    +-------------Os01g60960
|      |             |
|      |             +----------------------------------AT1G31320
|      |
|      |                                         +------AT1G65620
|      +-----------------------------------500.0-|
|                                                +------Os01g07480
|
+-------------------------------------------------------Os03g45750
```

LOB domain protein - PARS

```
       +--------------------------------------------------------------pg 11 126
       |
       |                                                       +------pg 4 41 33
       |                                                +333.1-|
       |                                         +295.2-|      +------Pt34310 12
       |                                         |      |
       |                                  +500.0-|      +-------------Pt27489 11
       |                                  |      |
       |                           +420.0-|      +--------------------Pt96082554
       |                           |      |
       |                           |      |                    +------AT2G28500
       |                    +420.0-|      +--------------500.0-|
       |                    |      |                           +------AT1G07900
       |             +391.0-|      |
       |             |      |      +----------------------------------AT2G30130
       |      +360.4-|      |
       |      |      |      +-----------------------------------------Os03g45750
       |      |      |
+------|-486.9-|      +------------------------------------------------AT1G31320
|      |      |
|      |      |                                                +------Os01g07480
|      |      +------------------------------------------498.5-|
|      |                                                       +------AT1G65620
|      |
|      +--------------------------------------------------------------Pt16032 32
|      |
|      |                                                       +------Pt24088 25
|      +-------------------------------------------------479.7-|
|      |                                                       +------Pp4027 11
|      |
|      +--------------------------------------------------------------Pt7277 16
|      |
|      +--------------------------------------------------------------AT2G30340
|      |
|      +--------------------------------------------------------------AT2G40470
|      |
|      +--------------------------------------------------------------Os05g07270
|
+---------------------------------------------------------------------Os01g60960
```

LRR group1 - NJ

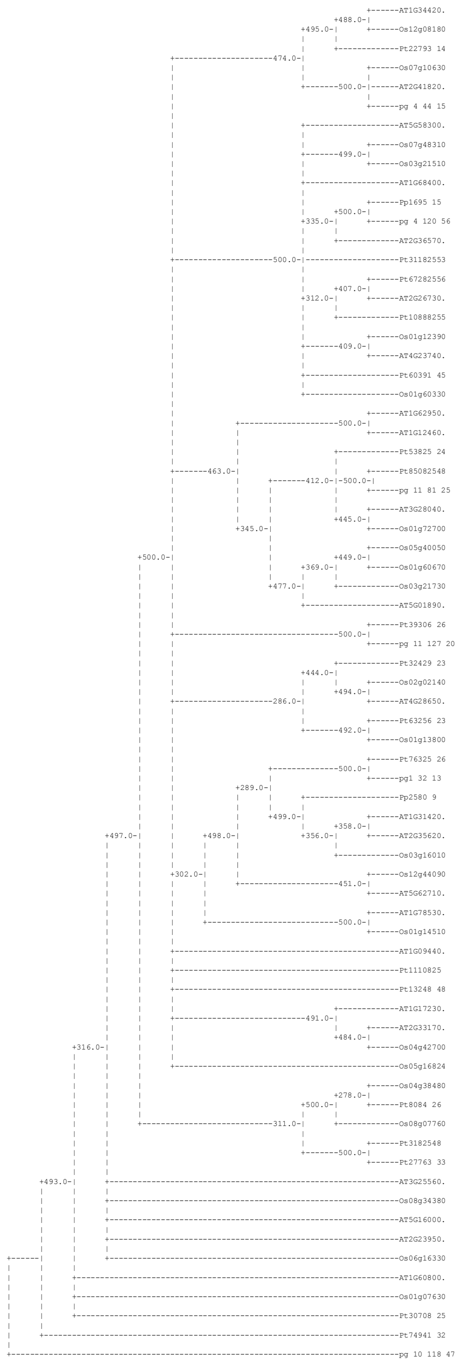

LRR group1 - PARS

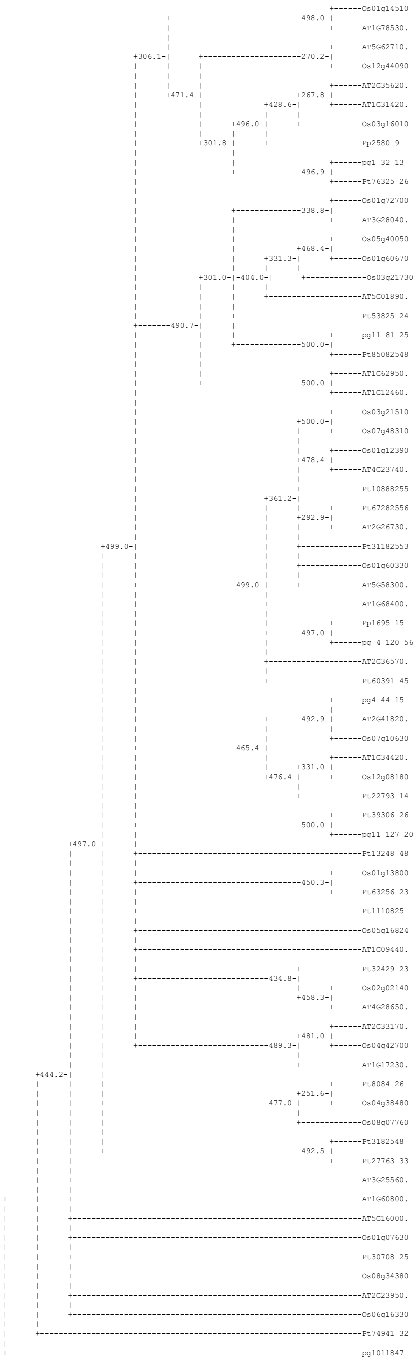

LRR group2 - NJ

```
                                                 +-------------Pp7691
                                          +482.0-|
                                          |      |      +------Pt78235
                                   +500.0-|      +410.0-|
                                   |      |             +------Pt68582554
                                   |      |
                                   |      +--------------------pg3 84 40
                                   |
                                   |      +--------------------Os01g17250
                                   |      |
                                   +352.0-|             +------AT5G21090
                                   |      |      +295.0-|
                                   |      +282.0-|      +------AT3G43740
                            +450.0-|             |
                            |      |             +-------------Os01g59440
                            |      |
                            |      +---------------------------Os03g32580
                            |      |
                            |      |                    +------pg1 112 39
                            |      +--------------500.0-|
                     +500.0-|      |                    +------Pt71982556
                     |      |      |
                     |      |      |                    +------pg2 89 36
                     |      |      +--------------323.0-|
                     |      |                           +------Pt8257
                     |      |
                     |      |             +--------------------AT4G33430
                     |      |             |
              +283.0-|      +-------499.0-|             +------AT1G34210
              |      |                    |      +467.0-|
              |      |                    |      |      +------AT1G71830
              |      |                    +310.0-|
              |      |                           |      +------Os08g07760
              |      |                           +439.0-|
              |      |                                  +------Os04g38480
              |      |
              |      +-----------------------------------------pg1 141 19
              |
              |                                         +------Os05g40050
              +-----------------------------------491.0-|
              |                                         +------Os03g21730
              |
              |                                         +------Pp2313519
              |                                  +317.0-|
              |                           +500.0-|      +------Pt75153
              |                           |      |
              |                    +482.0-|      +-------------pg4 98 84
              |                    |      |
              +--------------499.0-|      +--------------------Pt81825
              |                    |
              |                    +---------------------------AT5G23400
              |
              |                                         +------AT2G33170
              |                                  +345.0-|
              |                                  |      +------AT5G63930
              +----------------------------491.0-|
              |                                  |      +------AT1G17230
              |                                  +370.0-|
              |                                         +------Os07g05740
              |
              |                                         +------Os02g12450
              +-----------------------------------332.0-|
              |                                         +------Os04g03210
       +406.0-|
       |      |                                         +------Pt16978
       |      |                                  +420.0-|
       |      |                                  |      +------pg 12 45 20
       |      +----------------------------500.0-|
       |      |                                  +-------------Pt24269
       |      |                                  |
       |      |                                  +-------------pg 12 46 37
       |      |
       |      +------------------------------------------------Os05g51740
       |      |
       |      +------------------------------------------------Os05g07820
       |      |
       |      |                                         +------AT1G17750
       |      +-----------------------------------500.0-|
       |      |                                         +------AT1G73080
       |      |
       |      +------------------------------------------------Os10g02970
       |      |
       |      +------------------------------------------------pg1 16 8
       |      |
       |      +------------------------------------------------AT1G71400
       |      |
       |      |                                         +------AT5G48940
       |      |                                  +499.0-|
       |      +----------------------------458.0-|      +------AT3G24240
       |      |                                  |
       |      |                                  +-------------Os01g07560
       |      |
       |      +------------------------------------------------Os06g38990
       |      |
       |      |                                         +------Os07g41140
       |      +-----------------------------------372.0-|
       |      |                                         +------AT3G02130
       |      |
       |      +------------------------------------------------AT4G08850
       |
       +-------------------------------------------------------Pt10888255
       |
+------|                                                +------Pt31182553
|      +------------------------------------------484.0-|
|      |                                                +------pg 10 120 16
|      |
|      |                                         +-------------AT1G60630
|      |                                  +367.0-|
|      |                                  |      |      +------Pt69382554
|      |                                  |      +351.0-|
|      |                                  |             +------pg2 100 13
|      |                                  |
|      |                           +270.0-|      +-------------AT5G43020
|      |                           |      +269.0-|
|      |                           |      |      |      +------AT3G50230
|      |                           |      |      +367.0-|
|      |                    +324.0-|      |             +------AT5G67200
|      |                    |      |      |
|      |                    |      |      +--------------------Os04g55620
|      |                    |      |
|      +--------------355.0-|      +---------------------------Os02g58390
|      |                    |
|      |                    |                           +------AT1G68400
|      |                    |                    +276.0-|
|      |                    +--------------330.0-|      +------Os01g04230
|      |                                         |
|      |                                         +-------------Pt46120
|      |
|      +-------------------------------------------------------AT3G02880
|      |
|      +-------------------------------------------------------Os04g38910
|      |
|      |                                                +------AT1G48480
|      +------------------------------------------500.0-|
|      |                                                +------AT3G17840
|      |
|      |                                                +------Os03g21510
|      |                                         +497.0-|
|      +-----------------------------------477.0-|      +------Os07g48310
|      |                                         |
|      |                                         +-------------AT5G58300
|      |
|      +-------------------------------------------------------Os03g12250
|
+--------------------------------------------------------------AT2G26730 +-
```

LRR group2 - PARS

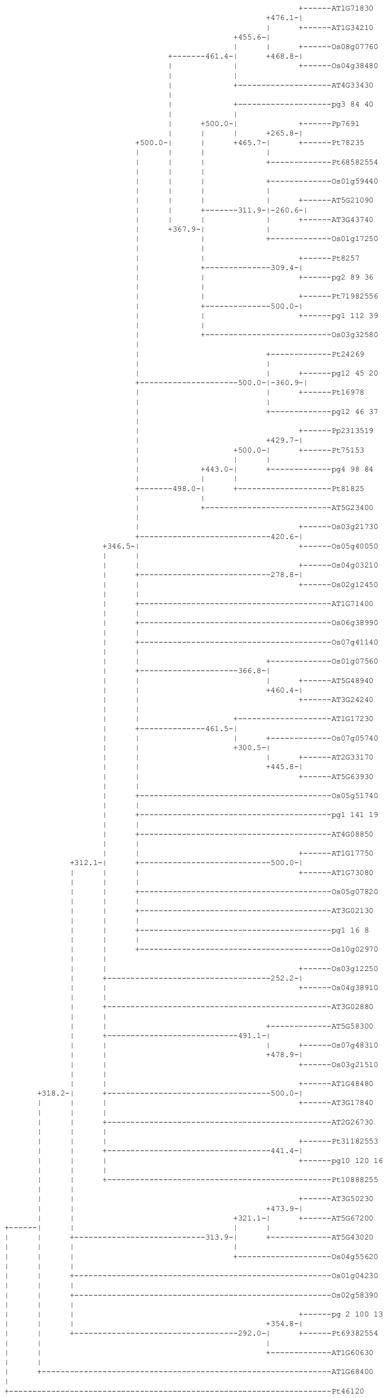

MADS - NJ

```
         +-------------------------------------------------------AT5G15800
         |
         |                                                +------Pt60599 21
         |                           +--------------496.0-|
         |                           |                    +------pg2 80 25
         |                           |
         |      +--------------496.0-|---------------------------AT1G26310
         |      |                    |
         |      |                    |      +--------------------Os07g41370
         |      |                    |      |
         |      |                    +322.0-|             +------AT5G60910
         |      |                           |      +296.0-|
         |      |                           +337.0-|      +------AT1G69120
         |      |                                  |
         |      |                                  +-------------Os03g54160
         |      |
         |      |                                         +------Pt3409 41
         |      |                                  +466.0-|
         |      |                                  |      +------pg6 108put
         |      |                                  |
         |      |                                  |      +------AT2G22540
         |      |                                  +287.0-|
         |      |                           +463.0-|      +------AT4G24540
         |      |                           |      |
         |      |                           |      +-------------Os03g08754
         |      |                           |      |
         |      |                    +481.0-|      |      +------Os02g52340
         |      |                    |      |      +463.0-|
         |      |                    |      |             +------Os06g11330
         |      |             +395.0-|      |
  +------|-385.0-|             |      |      +--------------------pg12 27
39
  |      |      |             |      |
  |      |      |             |      +---------------------------Pt9494 24
  |      |      |             |
  |      |      |      +497.0-|      +---------------------------pg7 3 29
  |      |      |      |      |      |
  |      |      |      |      |      |                    +------AT2G45660
  |      |      |      |      |      |             +405.0-|
  |      |      |      |      |      |      +368.0-|      +------AT4G22950
  |      |      |      |      +300.0-|      |      |
  |      |      +366.0-|             +323.0-|      +-------------Pt15282548
  |      |      |      |             |      |
  |      |      |      |             |      +--------------------AT5G62165
  |      |      |      |             |
  |      |      |      |             +---------------------------Os10g39130
  |      |      |      |
  |      |      |      |                                  +------Os06g06750
  |      |      |      +----------------------------500.0-|
  |      |      |                                         +------Os03g11614
  |      |      |
  |      |      +------------------------------------------------AT2G45650
  |      |      |
  |      |      +------------------------------------------------Os02g45770
  |      |      |
  |      |      +------------------------------------------------Os04g49150
  |      |
  |      +-------------------------------------------------------AT3G02310
  |      |
  |      +-------------------------------------------------------AT1G24260
  |      |
  |      +-------------------------------------------------------Os08g41950
  |
  +--------------------------------------------------------------Os09g32948
```

MADS - PARS

```
       +-------------------------------------------------------Os08g41950
       |
       +-------------------------------------------------------AT3G02310
       |
       |                                                +------Pt60599 21
       |                           +--------------499.0-|
       |                           |                    +------pg2 80 25
       |                           |
       |                           |                    +------AT5G60910
       +---------------------425.0-|             +251.2-|
       |                           |             |      +------Os03g54160
       |                           |      +271.0-|
       |                           |      |      |      +------AT1G26310
       |                           +379.7-|      +340.7-|
       |                                  |             +------AT1G69120
       |                                  |
       |                                  +--------------------Os07g41370
       |
       |                                                +------pg7 3 29
       |                                                |
       |                                                +------AT2G45660
       |                                                |
       |             +----------------------------258.0-|------Pt15282548
       |             |                                  |
       |             |                                  +------AT4G22950
       |             |                                  |
       |             |                                  +------Os10g39130
       |             |
       |             |                                  +------Os02g52340
       |             |                           +465.5-|
+------|             |                    +467.7-|      +------Os06g11330
|      |             |                    |      |
|      |             |                    |      +-------------Os03g08754
|      |             |                    |
|      |      +493.3-|                    +--------------------AT4G24540
|      |      |      |             +475.5-|
|      |      |      |             |      |           +------Pt3409 41
|      |      |      |             |      +-------465.5-|
|      |      |      |      +499.0-|      |           +------pg6 108put
|      |      |      |      |      |      |
|      |      |      |      |      |      +--------------------AT2G22540
|      +330.6-|      +390.6-|      |
|      |      |      |      |      +---------------------------pg12 27 39
|      |      |      |      |
|      |      |      |      +----------------------------------Pt9494 24
|      |      |      |
|      |      |      +-----------------------------------------AT5G62165
|      |      |
|      |      |                                         +------Os06g06750
|      |      +-----------------------------------363.9-|
|      |                                                +------Os03g11614
|      |
|      +-------------------------------------------------------AT1G24260
|      |
|      +-------------------------------------------------------AT2G45650
|      |
|      +-------------------------------------------------------Os04g49150
|      |
|      +-------------------------------------------------------Os02g45770
|      |
|      +-------------------------------------------------------AT5G15800
|
+--------------------------------------------------------------Os09g32948
```

Malate dehydrogenase - NJ

```
                                                               +------Pt29403 28
                                                        +500.0-|
                                                        |      +------pg2 56 34
                                                 +457.0-|
                                                 |      |      +------pg2 45 24
                                          +301.0-|      +486.0-|
                                          |      |             +------Pt27017 31
                                   +473.0-|      |
                                   |      |      +--------------------AT5G09660
                                   |      |
                     +-------500.0-|      +---------------------------Os03g56280
                     |             |
                     |             |                           +------AT2G22780
                     |             +---------------------330.0-|
                     |                                         +------Os12g43630
                     |
                     |                                         +------Os01g61380
              +420.0-|                                  +500.0-|
              |      |                           +489.0-|      +------Os08g33720
              |      |                           |      |
              |      |      +--------------326.0-|      +-------------AT3G47520
              |      |      |                    |
              |      |      |                    +--------------------Os07g43700
              |      |      |
              |      |      |                                  +------AT5G58330
              |      +433.0-|      +---------------------500.0-|
              |             |      |                           +------Os08g44810
              |             |      |
              |             |      |                           +------pg1 13 68
              |             |      |                    +476.0-|
              |             |      |                    |      +------Pt35882551
       +260.0-|             +500.0-|                    |
       |      |                    |             +255.0-|-------------Os10g33800
       |      |                    |             |      |
       |      |                    |             |      |      +------AT5G43330
       |      |                    |      +495.0-|      +397.0-|
       |      |                    |      |      |             +------AT1G04410
       |      |                    +483.0-|      |
       |      |                           |      +--------------------AT5G56720
       |      |                           |
+------|      |                           +---------------------------Os04g46560
|      |      |
|      |      |                                                +------Pt42482550
|      |      +------------------------------------------500.0-|
|      |      |                                                +------pg11 130 2
|      |      |
|      |      +-------------------------------------------------------Os05g49880
|      |
|      |                                                       +------AT1G53240
|      +-------------------------------------------------500.0-|
|                                                              +------AT3G15020
|
+---------------------------------------------------------------------Os01g46070
```

Malate dehydrogenase - PARS

```
                                                        +-------------pg2 45 24
                                                        |
                                                 +427.9-|-------------Pt27017 31
                                                 |      |
                                                 |      |      +------pg2 56 34
                                          +410.7-|      +500.0-|
                                          |      |             +------Pt29403 28
                                          |      |
                                   +266.8-|      |             +------Os03g56280
                                   |      |      +-------289.0-|
                                   |      |                    +------AT5G09660
              +--------------498.7-|      |
              |                    |      +---------------------------Os12g43630
              |                    |
              |                    +----------------------------------AT2G22780
              |
              |                                         +-------------Os10g33800
              |                                  +253.1-|
              |                                  |      |      +------pg1 13 68
              |                                  |      +500.0-|
              |                           +500.0-|             +------Pt35882551
       +499.0-|                           |      |
       |      |                           |      +--------------------AT1G04410
       |      |                    +496.5-|      |
       |      |                    |      |      +--------------------AT5G43330
       |      |             +500.0-|      |
       |      |             |      |      +---------------------------AT5G56720
       |      |             |      |
       |      |      +500.0-|      +----------------------------------Os04g46560
       |      |      |      |
       |      |      |      |                                  +------AT5G58330
       |      |      |      +----------------------------500.0-|
       |      +488.6-|                                         +------Os08g44810
       |             |
+------|             |                                  +-------------AT3G47520
|      |             |                           +494.1-|
|      |             |                           |      |      +------Os08g33720
|      |             +---------------------433.5-|      +499.0-|
|      |                                         |             +------Os01g61380
|      |                                         |
|      |                                         +--------------------Os07g43700
|      |
|      +--------------------------------------------------------------Os05g49880
|      |
|      |                                                       +------AT1G53240
|      |                                                +493.3-|
|      |                                                |      +------AT3G15020
|      +------------------------------------------252.3-|
|                                                       |      +------pg11 130 2
|                                                       +499.7-|
|                                                              +------Pt42482550
|
+---------------------------------------------------------------------Os01g46070
```

MAP kinase mitogen-activated protein kinase - NJ

```
                                                               +------Os08g06060
                                                               |
                                                               +------AT4G01370
                                                               |
                                   +---------------------367.0-|------Pt31182548
                                   |                           |
                                   |                           +------AT1G07880
                                   |                           |
                                   |                           +------Os10g38950
                                   |
                                   |                           +------pg 8 151 2
                            +500.0-|                    +497.0-|
                            |      |                    |      +------Pt92782553
                            |      |             +428.0-|
                            |      |             |      |      +------Os06g06090
                            |      |             |      +472.0-|
                            |      |      +478.0-|             +------AT2G43790
                            |      |      |      |
                     +500.0-|      |      |      |             +------Os03g17700
                     |      |      +492.0-|      +-------498.0-|
                     |      |             |                    +------AT3G45640
                     |      |             |
                     |      |             +---------------------------AT3G59790
                     |      |
              +497.0-|      |                                  +------Pt11782555
              |      |      |                           +500.0-|
              |      |      +---------------------500.0-|      +------pg 6 92 21
              |      |                                  |
       +499.0-|      |                                  +-------------Pt66991 31
       |      |      |
       |      |      +------------------------------------------------AT2G18170
       |      |
+------|      |                                                +------AT1G10210
|      |      +------------------------------------------498.0-|
|      |                                                       +------AT1G59580
|      |
|      +--------------------------------------------------------------Os02g05480
|
+---------------------------------------------------------------------Os06g48590
```

MAP kinase mitogen-activated protein kinase - PARS

```
                                                        +-------------Pt31182548
                                                        |
                                   +--------------302.7-|      +------Os10g38950
                                   |                    |      |
                                   |                    +304.8-|------AT4G01370
                                   |                           |
                                   |                           +------Os08g06060
                                   |
                                   |             +--------------------AT3G59790
                            +500.0-|             |
                            |      |             |             +------Os03g17700
                            |      |      +464.6-|      +499.6-|
                            |      |      |      |      |      +------AT3G45640
                            |      |      |      |      |
                            |      |      |      |      |      +------Pt92782553
                            |      |      |      +371.9-|-499.4-|
                            |      +298.9-|             |      +------pg 8 151 2
                     +490.1-|             |             |
                     |      |             |             |      +------Os06g06090
                     |      |             |             +285.9-|
                     |      |             |                    +------AT2G43790
                     |      |             |
                     |      |             +---------------------------AT1G07880
              +432.0-|      |
              |      |      |                                  +------Pt11782555
              |      |      |                                  |
              |      |      +----------------------------496.5-|------pg 6 92 21
              |      |                                         |
       +500.0-|      |                                         +------Pt66991 31
       |      |      |
       |      |      +------------------------------------------------AT2G18170
       |      |
+------|      +-------------------------------------------------------Os02g05480
|      |      |
|      |      +-------------------------------------------------------Os06g48590
|      |
|      +--------------------------------------------------------------AT1G10210
|
+---------------------------------------------------------------------AT1G59580
```

Mitochondrial substrate carrier - NJ

```
                     +---------------------------Os08g42380
                     |
                     |                    +------Pt13858255
       +-------482.0-|             +476.0-|
       |             |      +467.0-|      +------pg1 139 9
       |             |      |      |
       |             |      |      +-------------Pt3728 9
       |             +435.0-|
       |                    +--------------------Os11g03710
       |                    |
       |                    +--------------------Os12g03460
       |
       +-----------------------------------------pg3 50 12
       |
       +-----------------------------------------Os04g33220
       |
       |                           +-------------Pt15022 10
       |                    +389.0-|
       |                    |      |      +------AT5G55510
+------|             +259.0-|      +453.0-|
|      |             |      |             +------AT4G26670
|      |      +291.0-|      |
|      |      |      |      +--------------------Os03g09870
|      +489.0-|      |
|      |      |      +---------------------------Os10g22039
|      |      |
|      |      +----------------------------------Os12g33020
|      |
|      +-----------------------------------------Pp8185 15
|      |
|      +-----------------------------------------Os02g32530
|      |
|      +-----------------------------------------AT5G24650
|      |
|      +-----------------------------------------Pt37351 22
|      |
|      +-----------------------------------------pg 1 125 2
|
+------------------------------------------------AT3G49560
```

Mitochondrial substrate carrier - PARS

```
                     +---------------------------Os08g42380
                     |
                     |                    +------Pt13858255
       +-------482.0-|             +476.0-|
       |             |      +467.0-|      +------pg1 139 9
       |             |      |      |
       |             |      |      +-------------Pt3728 9
       |             +435.0-|
       |                    +--------------------Os11g03710
       |                    |
       |                    +--------------------Os12g03460
       |
       +-----------------------------------------pg3 50 12
       |
       +-----------------------------------------Os04g33220
       |
       |                           +-------------Pt15022 10
       |                    +389.0-|
       |                    |      |      +------AT5G55510
+------|             +259.0-|      +453.0-|
|      |             |      |             +------AT4G26670
|      |      +291.0-|      |
|      |      |      |      +--------------------Os03g09870
|      +489.0-|      |
|      |      |      +---------------------------Os10g22039
|      |      |
|      |      +----------------------------------Os12g33020
|      |
|      +-----------------------------------------Pp8185 15
|      |
|      +-----------------------------------------Os02g32530
|      |
|      +-----------------------------------------AT5G24650
|      |
|      +-----------------------------------------Pt37351 22
|      |
|      +-----------------------------------------pg 1 125 2
|
+------------------------------------------------AT3G49560
```

MIF2 (Mini Zinc Finger 2) - NJ

```
                                                 +------pg11 34 17
                                          +368.0-|
                                          |      +------Pt77669 12
                                          |
                                          +-------------Pt12383 13
                                          |
                                   +254.0-|-------------Pt34928 10
                                   |      |
                                   |      +-------------Pp7588 12
                                   |      |
                            +362.0-|      +-------------Pt29255 10
                            |      |      |
                            |      |      +-------------pg11 35 51
                            |      |
                            |      +--------------------AT3G50890
                            |
                            +---------------------------Os12g03110
                            |
                     +321.0-|---------------------------AT4G24660
                     |      |
                     |      +---------------------------Os11g03420
                     |      |
                     |      +---------------------------Os11g13930
              +469.0-|      |
              |      |      +---------------------------Os09g29130
              |      |      |
       +325.0-|      |      +---------------------------Os08g37400
       |      |      |
       |      |      +----------------------------------AT2G18350
+------|      |
|      |      +-----------------------------------------AT1G75240
|      |
|      +------------------------------------------------AT3G28917
|
+-------------------------------------------------------AT1G74660
```

MIF2 (Mini Zinc Finger 2) - PARS

```
+-------------------------------------------------------AT1G75240
 |
 |                                                +------AT4G24660
 |                                                |
 |                    +---------------------388.9-|------Os08g37400
 |                    |                           |
 |                    |                           +------Os09g29130
 |                    |
 |                    |                           +------pg11 35 51
 |                    |                    +387.7-|
 |                    |                    |      +------Pt12383 13
 |                    |                    |
 |                    |             +409.9-|      +------Pp7588 12
 |                    |             |      +500.0-|
 |                    |             |      |      +------Pt34928 10
 |                    |      +426.1-|      |
 |             +323.6-|      |      |      +-------------Pt29255 10
 |             |      |      |      |
 |             |      +353.0-|      |             +------pg11 34 17
 |             |      |      |      +-------494.2-|
 |             |      |      |                    +------Pt77669 12
 |             |      |      |
 |             |      |      +---------------------------AT3G50890
 |      +403.2-|      |
 |      |      |      |                           +------Os11g03420
 |      |      |      +---------------------500.0-|
 |      |      |      |                           +------Os12g03110
 |      |      |      |
 +------|      |      +----------------------------------Os11g13930
        |      |
        |      +-----------------------------------------AT2G18350
        |
        |                                         +------AT1G74660
        +-----------------------------------325.0-|
                                                  +------AT3G28917
```

Mitochondrial import inner membrane protein - NJ

```
                   +---------------------------Os08g42380
                   |
                   |                    +------Pt13858255
     +-------482.0-|             +476.0-|
     |             |      +467.0-|      +------pg1 139 9
     |             |      |      |
     |             |      |      +-------------Pt3728 9
     |             +435.0-|
     |                    +--------------------Os11g03710
     |                    |
     |                    +--------------------Os12g03460
     |
     +-----------------------------------------pg3 50 12
     |
     +-----------------------------------------Os04g33220
     |
     |                           +-------------Pt15022 10
     |                    +389.0-|
     |                    |      |      +------AT5G55510
+------|             +259.0-|      +453.0-|
|      |             |      |             +------AT4G26670
|      |      +291.0-|      |
|      |      |      |      +--------------------Os03g09870
|      +489.0-|      |
|      |      |      +---------------------------Os10g22039
|      |      |
|      |      +----------------------------------Os12g33020
|      |
|      +-----------------------------------------Pp8185 15
|      |
|      +-----------------------------------------Os02g32530
|      |
|      +-----------------------------------------AT5G24650
|      |
|      +-----------------------------------------Pt37351 22
|      |
|      +-----------------------------------------pg 1 125 2
|
+------------------------------------------------AT3G49560
```

Mitochondrial import inner membrane protein - PARS

```
                                          +---------------------------Os08g42380
                                          |
                                   +332.9-|                    +------Os12g03460
                                   |      |      +-------405.6-|
                                   |      |      |             +------Os11g03710
                                   |      +485.4-|
                                   |             |             +------pg1 139 9
                            +499.0-|             |      +494.8-|
                            |      |             +496.1-|      +------Pt13858255
                            |      |                    |
                            |      |                    +-------------Pt3728 9
                            |      |
                            |      +----------------------------------pg3 50 12
                     +482.0-|
                     |      |                    +--------------------Os03g09870
                     |      |                    |
                     |      |                    +--------------------Os12g33020
                     |      |             +391.0-|
                     |      |             |      |             +------AT4G26670
                     |      |             |      |      +493.4-|
              +492.3-|      +-------498.5-|      +328.1-|      +------AT5G55510
              |      |                    |             |
              |      |                    |             +-------------Pt15022 10
              |      |                    |
              |      |                    +---------------------------Os10g22039
              |      |
       +491.9-|      |                                         +------Pt37351 22
       |      |      |                                         |
       |      |      +-----------------------------------326.0-|------Pp8185 15
       |      |                                                |
       |      |                                                +------pg 1 125 2
+------|      |
|      |      |                                                +------AT3G49560
|      |      +------------------------------------------499.7-|
|      |                                                       +------AT5G24650
|      |
|      +--------------------------------------------------------------Os04g33220
|
+---------------------------------------------------------------------Os02g32530
```

MIP membrane intrinsic protein - NJ

```
                                                                      +------Pt57582552
                                                        +-------434.0-|
                                                        |             +------Pt75882550
                                                        |
                                                        |             +------AT3G26520
                                                 +266.0-|      +487.0-|
                                                 |      +437.0-|      +------AT2G36830
                                                 |      |      |
                                                 |      |      +-------------Os01g74450
                                                 |      |
                                                 |      +--------------------Os03g05290
                                                 |
                                                 |                    +------AT1G73190
                                                 |             +412.0-|
                                                 +-------417.0-|      +------AT1G17810
                            +--------------339.0-|             |
                            |                    |             +-------------Os10g35050
                            |                    |
                            |                    +---------------------------Os02g44080
                            |                    |
                            |                    |                    +------Pt17782547
                            |                    +--------------485.0-|
                            |                    |                    +------pg 8 12 46
                            |                    |
                            |                    +---------------------------Os06g22960
                            |                    |
                            |                    +---------------------------AT4G01470
                            |
                            |                                         +------AT4G10380
                            |                                  +411.0-|
                     +497.0-|             +--------------471.0-|      +------AT1G80760
                     |      |             |                    |
                     |      |             |                    +-------------Os10g36924
                     |      |      +424.0-|
                     |      |      |      |      +---------------------------Pt81447 14
                     |      |      |      |      |
                     |      |      |      |      |                    +------pg 9 110 9
                     |      |      |      +449.0-|             +374.0-|
                     |      |      |             |      +266.0-|      +------pg 9 104 2
                     |      |      |             |      |      |
                     |      |      |             +454.0-|      +-------------Pt24963 61
                     |      |      |                    |
                     |      |      |                    +--------------------Pt33957 31
                     |      |      |
                     |      |      |                                  +------AT5G37820
                     |      |      +----------------------------500.0-|
              +477.0-|      +496.0-|                                  +------AT5G37810
              |      |             |
              |      |             |                                  +------AT4G18910
              |      |             +----------------------------498.0-|
              |      |             |                                  +------AT4G19030
              |      |             |
              |      |             +-----------------------------------------Os05g11560
              |      |             |
              |      |             +-----------------------------------------Os02g51110
              |      |             |
              |      |             +-----------------------------------------Os06g35930
              |      |             |
              |      |             +-----------------------------------------Os02g13870
              |      |             |
              |      |             +-----------------------------------------Os01g10600
              |      |
              |      |                                                +------Os02g41860
              |      +------------------------------------------422.0-|
              |                                                       +------Os07g26690
              |
       +353.0-|                                  +---------------------------pg 5 130 4
       |      |                                  |
       |      |                                  |                    +------Pp13021 19
       |      |                                  |             +497.0-|
       |      |                                  |      +474.0-|      +------Pt90182548
       |      +----------------------------366.0-|      |      |
       |      |                                  +358.0-|      +-------------pg 4 34 34
       |      |                                  |      |
       |      |                                  |      +--------------------pg 4 35 13
       |      |                                  |
       |      |                                  +---------------------------Pt96982556
+------|      |
|      |      |                                                       +------Os02g57720
|      |      |                                                +369.0-|
|      |      +------------------------------------------287.0-|      +------Os04g47220
|      |      |                                                |
|      |      |                                                +-------------Os02g44630
|      |      |
|      |      +--------------------------------------------------------------AT4G00430
|      |      |
|      |      +--------------------------------------------------------------AT4G23400
|      |
|      |                                                              +------AT2G45960
|      +--------------------------------------------------------493.0-|
|                                                                     +------AT3G61430
|
+----------------------------------------------------------------------------AT1G01620
```

MIP membrane intrinsic protein - PARS

```
                                                        +---------------------------Os10g35050
                                                        |
                                                        |                    +------Pt17782547
                                                        |             +500.0-|
                                                        |             |      +------pg 8 12 46
                                                        |      +270.2-|
                                                        |      |      |      +------Pt57582552
                                                        |      |      +500.0-|
                                                        |      |             +------Pt75882550
                                                        |      |
                                                        |      |      +-------------Os01g74450
                                                        |      +443.2-|
                            +---------------------489.8-|      |      |      +------AT3G26520
                            |                           +386.2-|      +372.6-|
                            |                           |      |             +------AT2G36830
                            |                           |      |
                            |                           |      |             +------Os06g22960
                            |                           |      |      +491.5-|
                            |                           |      +485.5-|      +------Os02g44080
                            |                           |      |      |
                            |                           |      |      +-------------Os03g05290
                            |                           |      |
                            |                           |      +--------------------AT4G01470
                            |                           |
                            |                           |                    +------AT1G17810
                            |                           +--------------432.7-|
                            |                                                +------AT1G73190
                            |
                     +500.0-|                                         +-------------Os06g35930
                     |      |                                         |
                     |      |                                         +-------------Os01g10600
                     |      |                                         |
                     |      |                                         |      +------AT5G37820
                     |      |                                         +488.0-|
                     |      |                                         |      +------AT5G37810
                     |      |      +----------------------------300.8-|
                     |      |      |                                  +-------------Os02g13870
                     |      |      |                                  |
                     |      |      |                                  |      +------AT4G18910
                     |      |      |                                  +500.0-|
                     |      |      |                                  |      +------AT4G19030
                     |      |      |                                  |
                     |      |      |                                  +-------------Os05g11560
                     |      |      |
                     |      |      |                           +--------------------Pt24963 61
              +494.0-|      +290.6-|                           |
              |      |             |                    +262.1-|             +------pg 9 110 9
              |      |             |                    |      |      +260.0-|
              |      |             |                    |      +326.1-|      +------pg 9 104 2
              |      |             |             +495.3-|             |
              |      |             |             |      |             +-------------Pt33957 31
              |      |             |             |      |
              |      |             |      +253.2-|      +---------------------------Pt81447 14
              |      |             |      |      |
              |      |             |      |      |                           +------AT1G80760
              |      |             |      |      |                    +304.2-|
              |      |             +280.7-|      +--------------498.9-|      +------Os10g36924
              |      |                    |                           |
              |      |                    |                           +-------------AT4G10380
       +360.2-|      |                    |
       |      |      |                    +-----------------------------------------Os02g51110
       |      |      |
       |      |      |                                                       +------Os02g41860
       |      |      +-------------------------------------------------482.4-|
       |      |                                                              +------Os07g26690
       |      |
       |      |                                                              +------AT2G45960
       |      |                                                       +368.2-|
       |      |                                                       |      +------AT3G61430
       |      +-------------------------------------------------414.1-|
       |      |                                                       +-------------AT1G01620
       |      |                                                       |
       |      |                                                       +-------------AT4G00430
       |      |
+------|      +---------------------------------------------------------------------AT4G23400
|      |
|      |                                                              +-------------pg 4 35 13
|      |                                                              |
|      |                                                              |      +------Pp13021 19
|      |                                                       +358.6-|-287.7-|
|      |                                                       |      |      +------Pt90182548
|      |                                                +333.7-|      |
|      |                                                |      |      +-------------pg 4 34 34
|      +------------------------------------------411.9-|      |
|      |                                                |      +--------------------Pt96982556
|      |                                                |
|      |                                                +---------------------------pg 5 130 4
|      |
|      +----------------------------------------------------------------------------Os04g47220
|      |
|      +----------------------------------------------------------------------------Os02g57720
|
+-----------------------------------------------------------------------------------Os02g44630
```

MYB-related (TBP like) - NJ

```
                                                               +------Os12g41920
                                                 +-------460.0-|
                                                 |             +------AT1G49950
                                          +330.0-|
                                          |      |      +-------------Pt32199 18
                                          |      +286.0-|
                                   +495.0-|             |      +------Pt7793 21
                                   |      |             +490.0-|
                                   |      |                    +------pg7 159 27
                            +500.0-|      |
                            |      |      +---------------------------Os01g51154
                            |      |
                     +440.0-|      |                           +------AT1G72740
                     |      |      +---------------------373.0-|
                     |      |                                  +------AT1G17520
              +356.0-|      |
              |      |      +-----------------------------------------Os10g30719
       +428.0-|      |
       |      |      +------------------------------------------------AT2G37025
       |      |
+------|      +-------------------------------------------------------pg7 145 9
|      |
|      |                                                       +------pg6 138 27
|      |                                                +403.0-|
|      +------------------------------------------414.0-|      +------Os02g53670
|                                                       |
|                                                       +-------------AT1G72650
|
+---------------------------------------------------------------------AT1G17460
```

MYB-related (TBP like) - PARS

```
                                                        +------AT1G49950
                                                 +477.5-|
                                          +476.9-|      +------Os12g41920
                                          |      |
                                          |      +-------------Pt32199 18
                                   +476.7-|
                                   |      +--------------------Pt7793 21
                            +480.0-|      |
                            |      |      +--------------------pg7 159 27
                            |      |
                     +500.0-|      +---------------------------Os01g51154
                     |      |
                     |      |                           +------AT1G72740
                     |      +--------------------493.4-|
              +370.1-|                                  +------AT1G17520
              |      |
              |      +-----------------------------------------Os10g30719
              |      |
              |      +-----------------------------------------AT2G37025
              |
       +------|                                         +------Os02g53670
       |      +-----------------------------------347.4-|
       |      |                                         +------pg7 145 9
       |      |
       |      +------------------------------------------------AT1G17460
       |      |
       |      +------------------------------------------------AT1G72650
       |
       +-------------------------------------------------------pg6 138 27
```

NAM - NJ

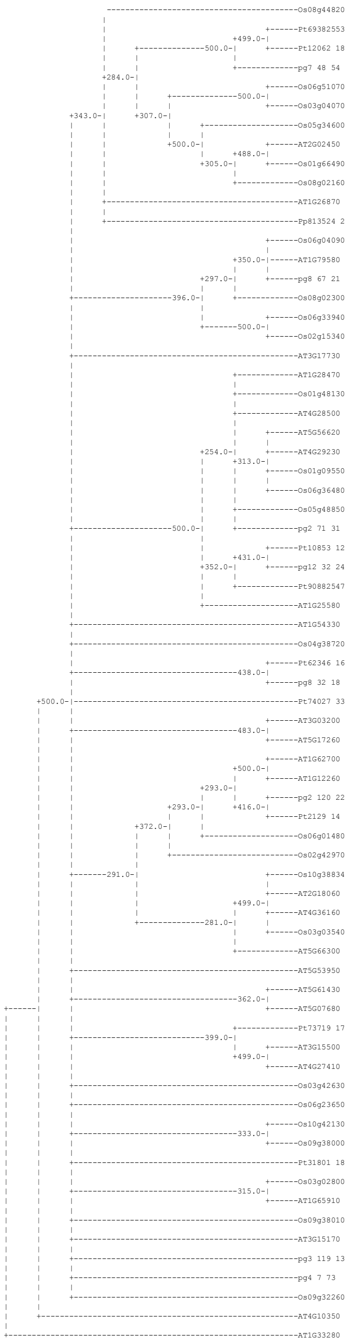
Os08g44820
Pt69382553
499.0
500.0
Pt12062 18
pg7 48 54
284.0
Os06g51070
500.0
Os03g04070
343.0
307.0
Os05g34600
AT2G02450
500.0
488.0
305.0
Os01g66490
Os08g02160
AT1G26870
Pp813524 2
Os06g04090
350.0
AT1G79580
297.0
pg8 67 21
396.0
Os08g02300
Os06g33940
500.0
Os02g15340
AT3G17730
AT1G28470
Os01g48130
AT4G28500
AT5G56620
254.0
AT4G29230
313.0
Os01g09550
Os06g36480
Os05g48850
500.0
pg2 71 31
Pt10853 12
431.0
352.0
pg12 32 24
Pt90882547
AT1G25580
AT1G54330
Os04g38720
Pt62346 16
438.0
pg8 32 18
500.0
Pt74027 33
AT3G03200
483.0
AT5G17260
AT1G62700
500.0
AT1G12260
293.0
pg2 120 22
293.0
416.0
Pt2129 14
372.0
Os06g01480
Os02g42970
291.0
Os10g38834
AT2G18060
499.0
AT4G36160
281.0
Os03g03540
AT5G66300
AT5G53950
AT5G61430
362.0
AT5G07680
Pt73719 17
399.0
AT3G15500
499.0
AT4G27410
Os03g42630
Os06g23650
Os10g42130
333.0
Os09g38000
Pt31801 18
Os03g02800
315.0
AT1G65910
Os09g38010
AT3G15170
pg3 119 13
pg4 7 73
Os09g32260
AT4G10350
AT1G33280

NAM - PARS

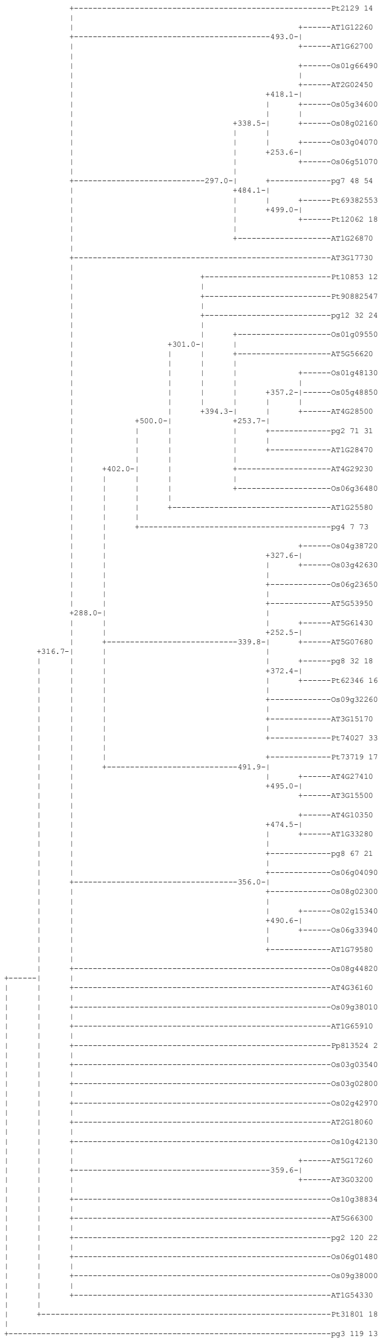
Pt2129 14
AT1G12260
493.0
AT1G62700
Os01g66490
AT2G02450
418.1
Os05g34600
338.5
Os08g02160
Os03g04070
253.6
Os06g51070
297.0
pg7 48 54
484.1
Pt69382553
499.0
Pt12062 18
AT1G26870
AT3G17730
Pt10853 12
Pt90882547
pg12 32 24
Os01g09550
301.0
AT5G56620
Os01g48130
357.2
Os05g48850
394.3
AT4G28500
500.0
253.7
pg2 71 31
AT1G28470
402.0
AT4G29230
Os06g36480
AT1G25580
pg4 7 73
Os04g38720
327.6
Os03g42630
Os06g23650
AT5G53950
288.0
AT5G61430
252.5
339.8
AT5G07680
316.7
pg8 32 18
372.4
Pt62346 16
Os09g32260
AT3G15170
Pt74027 33
Pt73719 17
491.9
AT4G27410
495.0
AT3G15500
AT4G10350
474.5
AT1G33280
pg8 67 21
Os06g04090
356.0
Os08g02300
Os02g15340
490.6
Os06g33940
AT1G79580
Os08g44820
AT4G36160
Os09g38010
AT1G65910
Pp813524 2
Os03g03540
Os03g02800
Os02g42970
AT2G18060
Os10g42130
AT5G17260
359.6
AT3G03200
Os10g38834
AT5G66300
pg2 120 22
Os06g01480
Os09g38000
AT1G54330
Pt31801 18
pg3 119 13

2OG-Fe(II) oxygenase family - NJ

```
                                                                      +----------------------------------pg 1 143 2
                                                                      |
           3                                                           |             +--------------------Pt12368255
                                                               +478.0-|             |
                                                               |      |      +438.0-|             +------Pt61703 23
                                                               |      |      |      |      +311.0-|
                                                               |      |      |      +490.0-|      +------Pt27344 24
                                                               |      +500.0-|             |
                                                               |             |             +-------------Pt15574 18
                                                               |             |
                                                               |             +---------------------------pg 1 151 2
                                                               |
                                                               |                    +--------------------AT1G17010
                                                        +396.0-|                    |
                                                        |      |             +500.0-|             +------AT4G25310
                                                        |      |             |      |      +286.0-|
                                                        |      |             |      |             +------AT4G25300
                                                        |      |             |      +428.0-|
                                                        |      |      +457.0-|             +-------------AT1G78550
                                                        |      |      |      |             |
                                                        |      |      |      |             +-------------AT1G17020
                                                        |      |      |      |
                                                        |      |      |      |                    +------Os01g24980
                                                        |      +482.0-|      +--------------500.0-|
                                                 +489.0-|             |                           +------Os01g25010
                                                 |      |             |
                                                 |      |             |             +--------------------Os03g63900
                                                 |      |             |             |
                                                 |      |             +-------471.0-|             +------Os10g40934
                                                 |      |                           |      +318.0-|
                                                 |      |                           +274.0-|      +------Os10g40880
                                          +354.0-|      |                                  |
                                          |      |      |                                  +-------------Os10g41020
                                          |      |      |
                                          |      |      |                                         +------Os01g70930
                                          |      |      +-----------------------------------497.0-|
                                   +427.0-|      |                                                +------AT3G21420
                                   |      |      |
                                   |      |      +-------------------------------------------------------AT3G11180
                                   |      |
                                   |      |                                                       +------Pt71624 23
                                   |      |                                                +499.0-|
                                   |      +------------------------------------------500.0-|      +------pg 8 53 39
                                   |                                                       |
                                   |                                                       +-------------Pt63541 22
                                   |
                                   |                                                              +------AT4G10500
                                   +--------------------------------------------------------500.0-|
                                   |                                                              +------AT4G10490
                            +432.0-|
                            |      |                                                              +------Os03g03034
                            |      |                                                       +349.0-|
                            |      |                                                +366.0-|      +------Os10g39140
                            |      |                                                |      |
                            |      |                                         +499.0-|      +-------------Os04g49194
                            |      |                                         |      |
                            |      +-----------------------------------397.0-|      +--------------------AT5G24530
                            |      |                                         |
                     +275.0-|      |                                         +---------------------------Os04g49210
                     |      |      |
                     |      |      |                                                              +------Pt47956 28
                     |      |      |                                                       +488.0-|
                     |      |      +-------------------------------------------------500.0-|      +------pg 4 129 3
                     |      |                                                              |
                     |      |                                                              +-------------Pt10882547
              +497.0-|      |
              |      |      |                                                                     +------pg3 135 36
              |      |      |                                                              +314.0-|
              |      |      +--------------------------------------------------------500.0-|      +------pg3 134 33
              |      |                                                                     |
       +500.0-|      |                                                                     +-------------Pt62704 28
       |      |      |
       |      |      |                                                                            +------AT2G44800
       |      |      +----------------------------------------------------------------------500.0-|
+------|      |                                                                                   +------AT3G60290
|      |      |
|      |      +------------------------------------------------------------------------------------------AT2G36690
|      |
|      +-------------------------------------------------------------------------------------------------Os09g39720
|
+--------------------------------------------------------------------------------------------------------Os08g44590
```

2OG-Fe(II) oxygenase family - PARS

```
                                                                             +------AT4G10490
                     +-------------------------------------------------500.0-|
                     |                                                       +------AT4G10500
                     |
                     |                                                +-------------Os04g49194
                     |                                         +418.5-|
                     |                                         |      |      +------Os10g39140
                     +-----------------------------------457.7-|      +280.0-|
                     |                                         |             +------Os03g03034
                     |                                         |
                     |                                         +--------------------AT5G24530
                     |
                     |                                                       +------Pt71624 23
                     |                                                +344.8-|
                     +------------------------------------------497.2-|      +------pg 8 53 39
                     |                                                |
                     |                                                +-------------Pt63541 22
                     |
                     |                                                       +------AT3G60290
                     +-------------------------------------------------500.0-|
                     |                                                       +------AT2G44800
                     |
                     |                                                       +------AT1G78550
                     |                                                       |
                     |                                                +406.5-|------AT4G25310
                     |                                                |      |
                     |                                         +464.9-|      +------AT4G25300
                     |                                         |      |
                     |                                  +500.0-|      +-------------AT1G17020
                     |                                  |      |
                     |                           +378.4-|      +--------------------AT1G17010
                     |                           |      |
                     |                           |      |                    +------Os01g24980
                     |                           |      +--------------499.0-|
                     |                    +480.4-|                           +------Os01g25010
                     |                    |      |
              +484.1-|                    |      |                           +------Os10g40934
              |      |                    |      |                    +256.0-|
              |      |                    |      |                    |      +------Os10g40880
              |      |                    |      +--------------334.6-|
              |      |                    |                           +-------------Os10g41020
              |      |                    |                           |
              |      |             +323.6-|                           +-------------Os03g63900
              |      |             |      |
              |      |             |      +-----------------------------------------pg 1 143 2
              |      |             |      |
              |      |             |      |                                  +------Pt27344 24
              |      |             |      |                           +484.7-|
              |      |             |      |                           |      +------Pt61703 23
              |      |      +275.6-|      |                    +289.5-|
              |      |      |      |      |                    |      +-------------Pt12368255
              |      |      |      |      +--------------500.0-|      |
              |      |      |      |                           |      +-------------Pt15574 18
              |      |      |      |                           |
              |      +406.7-|      |                           +--------------------pg 1 151 2
              |      |      |      |
       +499.0-|      |      |      +------------------------------------------------AT3G11180
       |      |      |      |
       |      |      |      |                                                +------Os01g70930
       |      |      |      +------------------------------------------429.4-|
       |      |      |                                                       +------AT3G21420
       |      |      |
       |      |      +--------------------------------------------------------------Os04g49210
       |      |      |
       |      |      |                                                       +------pg3 135 36
       |      |      |                                                +373.8-|
       |      |      +------------------------------------------500.0-|      +------pg3 134 33
+------|      |      |                                                |
|      |      |      |                                                +-------------Pt62704 28
|      |      |      |
|      |      |      |                                                +-------------Pt10882547
|      |      |      +------------------------------------------500.0-|
|      |      |                                                       |      +------pg 4 129 3
|      |      |                                                       +479.5-|
|      |      |                                                              +------Pt47956 28
|      |      |
|      |      +---------------------------------------------------------------------AT2G36690
|      |
|      +----------------------------------------------------------------------------Os08g44590
|
+-----------------------------------------------------------------------------------Os09g39720
```

Oxidoreductase - NJ

```
                                                 +------AT3G21360
                                          +392.0-|
                                   +407.0-|      +------Os01g09430
                                   |      |
                            +475.0-|      +-------------Pt11778255
                            |      |
                     +494.0-|      +--------------------pg2 36 22
                     |      |
                     |      +---------------------------Os08g29510
                     |
              +457.0-|                    +-------------pg5 106 14
              |      |                    |
              |      |             +460.0-|      +------Os01g03050
              |      |             |      |      |
              |      |             |      +491.0-|------Os02g22260
              |      +-------468.0-|             |
       +458.0-|                    |             +------AT1G15140
       |      |                    |
       |      |                    +--------------------Pt19525 15
       |      |
       |      |                                  +------Os08g01760
       |      |                                  |
+------|      +----------------------------421.0-|------pg8 97 86
|      |                                         |
|      |                                         +------AT5G63620
|      |
|      |                                         +------AT1G77120
|      |                           +-------375.0-|
|      |                           |             +------Os11g10520
|      +---------------------412.0-|
|                                  |      +-------------Os07g42924
|                                  +326.0-|
|                                         |      +------AT5G43940
|                                         +400.0-|
|                                                +------Os02g57040
|
+-------------------------------------------------------AT1G64710
```

Oxidoreductase - PARS

```
                                   +--------------------AT3G21360
                                   |
                            +496.9-|             +------Pt11778255
                            |      |      +497.9-|
                            |      +389.7-|      +------pg2 36 22
              +-------500.0-|             |
              |             |             +-------------Os01g09430
              |             |
              |             +---------------------------Os08g29510
              |
              |             +---------------------------Pt19525 15
              |             |
       +500.0-|      +497.6-|             +-------------Os01g03050
       |      |      |      |      +448.7-|
       |      |      |      |      |      |      +------AT1G15140
       |      |      |      +302.9-|      +396.4-|
       |      |      |             |             +------Os02g22260
       |      |      |             |
       |      |      |             +--------------------pg5 106 14
       |      +320.4-|
       |             |                           +------Os02g57040
       |             |                    +500.0-|
       |             |             +266.1-|      +------AT5G43940
+------|             |             |      |
|      |             |      +269.2-|      +-------------Os07g42924
|      |             |      |      |
|      |             |      |      |             +------Os11g10520
|      |             +250.9-|      +-------492.0-|
|      |                    |                    +------AT1G77120
|      |                    |
|      |                    +---------------------------AT1G64710
|      |
|      +------------------------------------------------Os08g01760
|      |
|      +------------------------------------------------AT5G63620
|
+-------------------------------------------------------pg8 97 86
```

Pectate Lyase - NJ

```
                                                                      +-------------AT3G09540
                                                               +458.0-|
                                                               |      |      +------AT3G55140
                                                        +289.0-|      +467.0-|
                                                        |      |             +------pg 7 107 3
                                                 +500.0-|      |
                                                 |      |      +--------------------Os01g36620
                                          +434.0-|      |
                                          |      |      +---------------------------Os01g62000
                                          |      |
                                          |      +----------------------------------Os05g22800
                                          |
                                          |                                  +------Os06g05209
                                          |                           +500.0-|
                                          |                    +496.0-|      +------Os06g05272
                                   +447.0-|                    |      |
                                   |      +--------------265.0-|      +-------------Os06g05260
                                   |      |                    |
                                   |      |                    |             +------Os02g12300
                                   |      |                    +-------271.0-|
                            +477.0-|      |                                  +------Os06g38510
                            |      |      |
                            |      |      +-----------------------------------------AT5G55720
                            |      |      |
                            |      |      +-----------------------------------------Os10g31910
                            |      |
                            |      +------------------------------------------------AT3G54920
                     +289.0-|
                     |      |                                  +--------------------pg8 120 45
                     |      |                                  |
                     |      |                                  |             +------Pt10668255
                     |      |                                  |      +310.0-|
                     |      |                           +455.0-|-466.0-|      +------pg8 118 40
                     |      |                           |      |      |
                     |      |                           |      |      +-------------pg8 117 79
                     |      +---------------------277.0-|      |
                     |                                  |      +--------------------Pt6458 35
                     |                                  |
              +393.0-|                                  |                    +------Pt65047 23
              |      |                                  +--------------500.0-|
              |      |                                                       +------pg9 43 69
              |      |
              |      |                                                       +------AT4G13710
              |      |                                  +--------------465.0-|
              |      |                                  |                    +------AT1G04680
              |      |                           +283.0-|
              |      |                           |      |      +--------------------AT5G48900
       +499.0-|      |                           |      |      |
       |      |      |                           |      +383.0-|             +------AT3G24670
       |      |      +---------------------386.0-|             |      +269.0-|
       |      |                                  |             +367.0-|      +------AT3G07010
       |      |                                  |                    |
       |      |                                  |                    +-------------AT4G13210
+------|      |                                  |
|      |      |                                  +----------------------------------Os04g05050
|      |      |
|      |      |                                                              +------AT3G27400
|      |      +--------------------------------------------------------287.0-|
|      |                                                                     +------AT1G67750
|      |
|      +----------------------------------------------------------------------------AT4G24780
|
+-----------------------------------------------------------------------------------AT5G63180
```

Pectate Lyase - PARS

```
       +------------------------------------------------AT4G24780
       |
       |                                         +------Pt10668255
       |                                  +399.6-|
       |             +--------------299.5-|      +------pg8 117 79
       |             |                    |
       |             |                    +-------------pg8 118 40
       |             |
       |             |                    +-------------AT1G04680
       |             |                    |
       |             |                    |      +------AT5G48900
       |             |                    |      |
       |             |                    |      +------AT3G07010
       |             |             +344.9-|-358.0-|
       |             |             |      |      +------AT3G24670
       |             |             |      |      |
       |             |      +370.9-|      |      +------AT4G13210
       |             |      |      |      |
       |             |      |      |      +-------------AT4G13710
       |             |      |      |
       |             |      |      +--------------------Os04g05050
       |             |      |
+------|             |      |                    +------Os01g62000
|      |             |      |                    |
|      |             |      |                    +------pg 7 107 3
|      |             |      |                    |
|      |             +265.6-|      +-------500.0-|------AT3G55140
|      |             |      |      |             |
|      |             |      |      |             +------AT3G09540
|      |      +295.4-|      |      |             |
|      |      |      |      |      |             +------Os01g36620
|      |      |      |      |      |
|      |      |      |      |      |             +------Os06g05272
|      |      |      |      |      |      +500.0-|
|      |      |      |      |      +441.3-|      +------Os06g05209
|      |      |      |      |      |      |
|      |      |      |      |      |      +-------------Os06g05260
|      |      |      |      +473.5-|
|      |      |      |             +--------------------AT5G55720
|      |      |      |             |
|      |      |      |             +--------------------Os05g22800
|      |      |      |             |
|      |      |      |             |             +------Os02g12300
|      |      |      |             +-------250.1-|
|      +474.5-|      |             |             +------Os06g38510
|             |      |             |
|             |      |             +--------------------Os10g31910
|             |      |             |
|             |      |             +--------------------AT3G54920
|             |      |
|             |      +----------------------------------Pt6458 35
|             |      |
|             |      +----------------------------------pg8 120 45
|             |      |
|             |      |                           +------pg9 43 69
|             |      +---------------------498.1-|
|             |                                  +------Pt65047 23
|             |
|             |                                  +------AT3G27400
|             +----------------------------324.3-|
|                                                +------AT1G67750
|
+-------------------------------------------------------AT5G63180
```

Pectinacetylesterase - NJ

```
                                                 +--------------------Os02g47400
                                                 |
                                          +318.0-|             +------AT4G19410
                                          |      |      +500.0-|
                                          |      +498.0-|      +------AT5G45280
                                   +338.0-|             |
                                   |      |             +-------------Os04g51340
                                   |      |
                                   |      +---------------------------AT4G19420
                            +394.0-|
                            |      |      +---------------------------Os01g21630
                            |      |      |
                            |      |      |                    +------AT5G26670
                            |      +388.0-|             +492.0-|
                            |             |      +495.0-|      +------AT3G05910
                            |             |      |      |
                            |             +456.0-|      +-------------Os07g44070
                            |                    |
                     +341.0-|                    |             +------AT2G46930
                     |      |                    +-------277.0-|
                     |      |                                  +------AT3G62060
                     |      |
                     |      |                                  +------Os01g66850
                     |      |                           +500.0-|
                     |      |                           |      +------Os01g66830
              +500.0-|      |                    +342.0-|
              |      |      |                    |      |      +------Os01g74330
              |      |      +--------------354.0-|      +500.0-|
              |      |                           |             +------Os05g02120
              |      |                           |
       +500.0-|      |                           +--------------------AT3G09410
       |      |      |
       |      |      +------------------------------------------------AT5G23870
       |      |
+------|      |                                                +------pg 7 29 12
|      |      +------------------------------------------313.0-|
|      |                                                       +------Pt58024 18
|      |
|      +--------------------------------------------------------------pg 6 122 7
|
+---------------------------------------------------------------------Pt45182548
```

Pectinacetylesterase - PARS

```
                                          +------AT3G62060
                                   +265.1-|
                                   |      +------AT2G46930
                                   |
                     +-------471.7-|      +------AT3G05910
                     |             +496.5-|
                     |             |      +------AT5G26670
                     |             |
                     |             +-------------Os07g44070
                     |
                     |             +-------------Pt58024 18
                     |             |
                     |             |      +------pg 6 122 7
                     |      +457.4-|-500.0-|
                     |      |      |      +------Pt45182548
                     +338.5-|      |
              +398.9-|      |      +-------------pg 7 29 12
              |      |      |
              |      |      +--------------------AT5G23870
              |      |
              |      +---------------------------Os01g21630
              |      |
              |      |             +-------------Os04g51340
              |      +-------430.4-|
              |      |             |      +------AT4G19410
       +500.0-|      |             +500.0-|
       |      |      |                    +------AT5G45280
       |      |      |
       |      |      |                    +------AT4G19420
       |      |      +--------------301.9-|
       |      |                           +------Os02g47400
+------|      |
|      |      |                           +------Os05g02120
|      |      |                    +497.0-|
|      |      +--------------348.6-|      +------Os01g74330
|      |                           |
|      |                           +-------------AT3G09410
|      |
|      +------------------------------------------Os01g66830
|
+------------------------------------------------Os01g66850
```

Pectinesterase - NJ

```
                                          +------AT3G43270
              +---------------------491.0-|
              |                           +------AT4G33220
              |
              +----------------------------------AT3G05620
              |
              |                           +------Pt47282553
              +---------------------492.0-|
              |                           +------Pt35782549
              |
              |                           +------AT3G05610
              |      +--------------500.0-|
              |      |                    +------AT5G27870
              |      |
              +372.0-|             +-------------pg 11 139
              |      |      +500.0-|
              |      |      |      |      +------Pt35403 30
              |      |      |      +256.0-|
              |      +440.0-|             +------Pt35402 19
              |             |
              |             |             +------pg5 50 56
              |             +-------475.0-|
              |                           +------Pt76730 32
              |
              |                           +------pg11 140 3
              |                    +319.0-|
              |             +359.0-|      +------pg11 141 1
              |             |      |
              |             |      +-------------Pt38745 17
              |             |
              |             |      +-------------Os04g35770
              +-------381.0-|-264.0-|
              |             |      |      +------AT5G64640
              |             |      +456.0-|
              |             |             +------AT5G09760
              |             |
              |             +--------------------Pt49074 42
              |
              |                           +------Os01g21034
              |                    +288.0-|
              |                    |      +------Pt3375 16
              |                    |
       +500.0-|--------------316.0-|      +------Pt66938 13
       |      |                    +467.0-|
       |      |                    |      +------pg5 144 9
       |      |                    |
       |      |                    +-------------Os07g47830
       |      |
       |      |                           +------pg10 125 2
       |      |                    +467.0-|
       |      +--------------365.0-|      +------Pt68782554
       |      |                    |
       |      |                    +-------------AT2G45220
       |      |
       |      |                           +------AT1G53830
       |      |                    +465.0-|
       |      +--------------271.0-|      +------AT3G14310
       |      |                    |
       |      |                    +-------------Os02g54190
       |      |
       |      +----------------------------------AT3G59010
       |      |
       |      +----------------------------------Os01g15039
       |      |
       |      +----------------------------------pg 7 130 9
       |      |
       |      |                           +------Os01g57854
       |      +---------------------337.0-|
       |      |                           +------Os01g65790
       |      |
       |      +----------------------------------Pt13983 23
       |      |
       |      +----------------------------------AT4G02300
       |      |
       |      +----------------------------------Os08g34900
       |      |
       |      +----------------------------------AT1G11580
       |      |
       |      |                           +------AT5G53370
       |      +---------------------484.0-|
+------|      |                           +------AT3G49220
|      |      |
|      |      |                           +------Os02g18650
|      |      +---------------------367.0-|
|      |                                  +------Os01g13320
|      |
|      |                           +-------------Pt36313 24
|      +---------------------413.0-|
|      |                           |      +------pg6 52 15
|      |                           +499.0-|
|      |                                  +------Pt18335 26
|      |
|      +-----------------------------------------AT2G36710
|      |
|      |                                  +------pg9 115 15
|      |                           +390.0-|
|      +---------------------493.0-|      +------Pt11996 10
|      |                           |
|      |                           +-------------Pt41782556
|      |
|      +-----------------------------------------AT1G05310
|      |
|      |                                  +------Os05g44600
|      |                           +402.0-|
|      +---------------------494.0-|      +------Os01g53990
|      |                           |
|      |                           +-------------AT5G19730
|      |
|      +-----------------------------------------AT2G36700
|      |
|      +-----------------------------------------Os04g46740
|      |
|      +-----------------------------------------Os07g41650
|      |
|      +-----------------------------------------AT5G47500
|      |
|      +-----------------------------------------Os09g39760
|      |
|      +-----------------------------------------Os08g10604
|
+------------------------------------------------AT5G55590
```

Pectinesterase - PARS

```
+-----------------------------------------AT3G05620
 |
 |                                  +------Pt41782556
 |                           +499.9-|
 |                    +499.9-|      +------Pt11996 10
 |                    |      |
 |                    |      +-------------pg9 115 15
 |                    |
 |                    |              +------Pt18335 26
 |                    |       +499.3-|
 |             +253.8-|-409.8-|      +------pg6 52 15
 |             |      |       |
 |             |      |       +-------------Pt36313 24
 |             |      |
 |             |      +--------------------Os04g46740
 |             |      |
 |             |      +--------------------AT5G47500
 |             |
 |             +---------------------------AT2G36710
 |             |
 |             +---------------------------Os08g10604
 |             |
 |             +---------------------------Os09g39760
 |      +499.9-|
 |      |      +---------------------------Os07g41650
 |      |      |
 |      |      |             +-------------AT5G19730
 |      |      +-------482.1-|
 |      |      |             |      +------Os01g53990
 |      |      |             +435.0-|
 |      |      |                    +------Os05g44600
 |      |      |
 |      |      +---------------------------AT2G36700
 |      |      |
 |      |      +---------------------------AT5G55590
 |      |      |
 |      |      +---------------------------AT1G05310
 |      |
 |      |                    +-------------Os02g54190
 |      +--------------263.1-|
 |      |                    |      +------AT1G53830
 |      |                    +315.3-|
 |      |                           +------AT3G14310
 |      |
 |      |                           +------AT4G33220
 |      +---------------------464.1-|
 |      |                           +------AT3G43270
 |      |
 |      |                           +------AT5G27870
 |      +---------------------499.9-|
 |      |                           +------AT3G05610
 |      |
 |      +----------------------------------Pt13983 23
 |      |
 |      |                           +------AT5G64640
 |      |                    +393.0-|
 |      |             +412.7-|      +------AT5G09760
 |      |             |      |
 |      |             |      +-------------Os04g35770
 |      |             |
 |      |             |             +------pg11 140 3
 |      +-------337.5-|      +398.4-|
 |      |             +285.0-|      +------pg11 141 1
 |      |             |      |
 |      |             |      +-------------Pt38745 17
 |      |             |
 |      |             +--------------------Pt49074 42
 |      |
 |      |                           +------Pt47282553
 |      +---------------------393.7-|
 |      |                           +------Pt35782549
 +------|
        +----------------------------------Os02g18650
        |
        |                           +------pg10 125 2
        +---------------------368.2-|
        |                           +------Pt68782554
        |
        |                    +-------------Os07g47830
        |                    |
        |                    +-------------Os01g21034
        +--------------274.8-|
        |                    |      +------Pt3375 16
        |                    |      |
        |                    +253.0-|------pg5 144 9
        |                           |
        |                           +------Pt66938 13
        |
        +----------------------------------pg 7 130 9
        |
        +----------------------------------AT3G59010
        |
        +----------------------------------Os01g15039
        |
        |                           +------Pt76730 32
        |                    +495.9-|
        |                    |      +------pg5 50 56
        +--------------366.8-|
        |                    |      +------Pt35402 19
        |                    |      |
        |                    +496.2-|------pg 11 139
        |                           |
        |                           +------Pt35403 30
        |
        +----------------------------------Os08g34900
        |
        +----------------------------------AT1G11580
        |
        |                           +------AT5G53370
        +---------------------463.5-|
        |                           +------AT3G49220
        |
        +----------------------------------AT4G02300
        |
        +----------------------------------Os01g13320
        |
        +----------------------------------AT2G45220
        |
        |                           +------Os01g57854
        +---------------------380.5-|
                                    +------Os01g65790
```

Peroxidase - NJ

```
                                                                             +------Os12g02080
                                                                      +500.0-|
                                                                      |      +------Os11g02130
                                                               +408.0-|
                                                               |      |      +------AT5G05340
                                                               |      +391.0-|
                                                        +461.0-|             +------Os06g35520
                                                        |      |
                                                        |      |             +------AT5G58400
                                                        |      +-------500.0-|
                                                 +317.0-|                    +------AT5G58390
                                                 |      |
                                                 |      |                    +------pg12 127 2
                                                 |      |             +498.0-|
                                                 |      |             |      +------Pt44182553
                                                 |      +-------461.0-|
                                                 |                    |      +------pg12 128 1
                                          +445.0-|                    +500.0-|
                                          |      |                           +------Pt60190 21
                                          |      |
                                          |      |                           +------Os04g55740
                                          |      +---------------------337.0-|
                                          |      |                           +------Os07g47990
                                   +342.0-|      |
                                   |      |      |                           +------pg6 60 64
                                   |      |      +---------------------500.0-|
                                   |      |                                  +------Pt12997 27
                                   |      |
                                   |      |                                  +------AT1G14540
                                   |      +----------------------------500.0-|
                                   |                                         +------AT1G14550
                                   |
                                   |                                         +------AT5G06720
                                   +-----------------------------------407.0-|
                                   |                                         +------Os03g13200
                                   |
                            +380.0-|                                         +------pg11 64 19
                            |      |                           +-------500.0-|
                            |      |                           |             +------Pt69903 16
                            |      |                           |
                            |      |                           |             +------AT5G64100
                            |      |                           |      +500.0-|
                            |      |                           |      |      +------AT5G64110
                            |      |                           +483.0-|
                            |      |                    +495.0-|      |      +------AT5G39580
                            |      |                    |      |      +496.0-|
                            |      |                    |      |             +------AT5G64120
                     +322.0-|      |                    |      |
                     |      |      |                    |      |             +------Os11g10460
                     |      |      +--------------495.0-|      +-------364.0-|
                     |      |                           |      |             +------AT2G41480
                     |      |                           |      |
                     |      |                           |      +--------------------Os01g07770
                     |      |                           |
                     |      |                           |                    +------Os03g02939
                     |      |                           +--------------500.0-|
                     |      |                                                +------Os10g39170
                     |      |
                     |      |                                                +------Os01g15810
                     |      +------------------------------------------464.0-|
              +500.0-|                                                       +------AT1G44970
              |      |
              |      |                                         +--------------------Os04g34630
              |      |                                         |
              |      +-----------------------------------261.0-|             +------Os01g36240
              |      |                                         |      +482.0-|
              |      |                                         +445.0-|      +------AT5G66390
              |      |                                                |
       +500.0-|      |                                                +-------------AT3G50990
       |      |      |
       |      |      +--------------------------------------------------------------Os01g15830
       |      |      |
       |      |      |                                                       +------pg5 64 22
+------|      |      +-------------------------------------------------500.0-|
|      |      |                                                              +------Pt12082551
|      |      |
|      |      +---------------------------------------------------------------------AT4G36430
|      |
|      +----------------------------------------------------------------------------AT2G18150
|
+-----------------------------------------------------------------------------------AT2G18140
```

Peroxidase - PARS

```
                                                                      +------pg11 64 19
                                                        +-------500.0-|
                                                        |             +------Pt69903 16
                                                        |
                                                 +452.6-|      +-------------AT5G39580
                                                 |      |      |
                                                 |      |      |      +------AT5G64110
                                                 |      +277.0-|-499.0-|
                                                 |             |      +------AT5G64100
                                          +372.0-|             |
                                          |      |             +-------------AT5G64120
                                          |      |
                                   +331.8-|      +---------------------------AT2G41480
                                   |      |      |
                                   |      |      +---------------------------Os01g07770
                            +479.8-|      |
                            |      |      +----------------------------------Os11g10460
                            |      |
                            |      |                                  +------Os03g02939
                            |      +----------------------------499.0-|
                            |                                         +------Os10g39170
                            |
                            |                                         +------Os03g13200
                            |                           +-------353.1-|
                            |                           |             +------AT5G06720
                            |                           |
                            |                           |             +------Pt12997 27
                     +349.4-|                           +-------500.0-|
                     |      |                           |             +------pg6 60 64
                     |      |                           |
                     |      |                           |             +------AT5G05340
                     |      |                           |      +319.0-|
                     |      |                           |      |      +------Os06g35520
                     |      |                           |      |
                     |      |                           |      |      +------AT5G58390
                     |      |                           +417.5-|-402.5-|
                     |      |                           |      |      +------AT5G58400
                     |      |                           |      |
                     |      |                           |      |      +------Os12g02080
                     |      +---------------------270.3-|      +500.0-|
                     |                                  |             +------Os11g02130
                     |                                  |
                     |                                  |             +------Pt60190 21
                     |                                  |      +438.2-|
                     |                                  |      |      +------pg12 128 1
                     |                                  +374.4-|
                     |                                  |      |      +------pg12 127 2
                     |                                  |      +460.5-|
                     |                                  |             +------Pt44182553
              +483.8-|                                  |
              |      |                                  |             +------AT1G14550
              |      |                                  +-------500.0-|
              |      |                                  |             +------AT1G14540
              |      |                                  |
              |      |                                  +--------------------Os04g55740
              |      |                                  |
              |      |                                  +--------------------Os07g47990
              |      |
              |      |                                         +-------------AT5G66390
              |      |                                  +300.1-|
              |      |                                  |      |      +------Os01g36240
              |      +----------------------------260.0-|      +286.5-|
       +500.0-|      |                                  |             +------AT3G50990
       |      |      |                                  |
       |      |      |                                  +--------------------Os01g15830
       |      |      |
       |      |      |                                                +------pg5 64 22
       |      |      +------------------------------------------500.0-|
       |      |      |                                                +------Pt12082551
       |      |      |
+------|      |      |                                                +------Os01g15810
|      |      |      |                                         +278.5-|
|      |      |      +-----------------------------------289.4-|      +------AT1G44970
|      |      |                                                |
|      |      |                                                +-------------Os04g34630
|      |      |
|      |      +--------------------------------------------------------------AT4G36430
|      |
|      +---------------------------------------------------------------------AT2G18150
|
+----------------------------------------------------------------------------AT2G18140
```

Peroxidase PER12 - NJ

```
                                                 +---------------------------AT4G16270
                                                 |
                                                 |             +-------------AT4G36430
                                                 |      +500.0-|
                                                 |      |      |      +------AT2G18140
                                          +500.0-|-447.0-|      +490.0-|
                                          |      |      |             +------AT2G18150
                                          |      |      |
                                          |      |      +--------------------AT1G44970
                                   +329.0-|      |
                                   |      |      +---------------------------AT5G06730
                                   |      |
                                   |      |                           +------Os01g73200
                            +289.0-|      |                    +500.0-|
                            |      |      +--------------464.0-|      +------Os01g73170
                            |      |                           |
                     +500.0-|      |                           +-------------Os01g73220
                     |      |      |
                     |      |      +-----------------------------------------Os04g59150
              +499.0-|      |
              |      |      +------------------------------------------------AT1G71695
              |      |
       +500.0-|      +-------------------------------------------------------pg1 45 25
       |      |
       |      |                                                       +------Pt40769 22
+------|      +-------------------------------------------------500.0-|
|      |                                                              +------Pt94882551
|      |
|      +---------------------------------------------------------------------Pt31442 28
|
+----------------------------------------------------------------------------pg2 140 51
```

Peroxidase PER12 - PARS

```
                                                 +------Pt40769 22
                                          +499.0-|
                                          |      +------Pt94882551
                                   +497.0-|
                                   |      |      +------pg2 140 51
                     +-------480.2-|      +500.0-|
                     |             |             +------Pt31442 28
                     |             |
                     |             +--------------------pg1 45 25
                     |
              +334.2-|             +--------------------AT1G44970
              |      |      +372.3-|
              |      |      |      |      +-------------AT4G36430
              |      |      |      +483.7-|
              |      |      |             |      +------AT2G18150
              |      +489.1-|             +469.2-|
       +285.9-|             |                    +------AT2G18140
       |      |             |
       |      |             |                    +------AT4G16270
       |      |             +--------------299.2-|
       |      |                                  +------AT5G06730
+------|      |
|      |      +-----------------------------------------AT1G71695
|      |
|      |                                         +------Os01g73200
|      |                                  +500.0-|
|      +----------------------------482.0-|      +------Os01g73170
|                                         |
|                                         +-------------Os01g73220
|
+-------------------------------------------------------Os04g59150
```

pfkB-type carbohydrate kinase - NJ

```
                                                  +------Pt42114 22
                                          +499.0-|
                                   +500.0-|       +------pg6 48 21
                                   |      |
       +---------------------500.0-|      +-------------AT1G17160
       |                           |
       |                           +--------------------Os01g47550
       |
       |                    +---------------------------AT5G51830
       |                    |
       |                    |      +--------------------Os01g66940
       |             +460.0-|      |
       |             |      |      |             +------AT3G59480
+------|             |      |      |      +473.0-|
|      |             |      |      |      |      +------AT1G06030
|      |             |      +470.0-|-423.0-|
|      |      +387.0-|             |      |      +------AT4G10260
|      |      |      |             |      +500.0-|
|      |      |      |             |             +------Os08g02120
|      |      |      |             |
|      |      |      |             +--------------------Os01g63220
|      |      |      |
|      +445.0-|      +----------------------------------pg7 78 95
|             |
|             +-----------------------------------------AT1G66430
|             |
|             +-----------------------------------------Pt79682551
|             |
|             +-----------------------------------------Os06g12600
|
+-------------------------------------------------------Pt35408 32
```

pfkB-type carbohydrate kinase - PARS

```
+-------------------------------------------------------pg7 78 95
|
|                                                +------Pt42114 22
|                                         +500.0-|
|                                  +452.7-|      +------pg6 48 21
|                                  |      |
|                           +500.0-|      +-------------AT1G17160
|                           |      |
|                    +492.5-|      +--------------------Os01g47550
|                    |      |
|                    |      +---------------------------Os01g63220
|             +433.1-|
|             |      |             +--------------------Os01g66940
|             |      |             |
|             |      +-------471.1-|             +------AT1G06030
|             |                    |      +253.8-|
|      +443.7-|                    |      |      +------AT3G59480
|      |      |                    +479.8-|
|      |      |                           |      +------Os08g02120
|      |      |                           +498.5-|
|      |      |                                  +------AT4G10260
|      |      |
|      |      +-----------------------------------------AT5G51830
+------|
       +------------------------------------------------Os06g12600
       |
       +------------------------------------------------Pt79682551
       |
       +------------------------------------------------Pt35408 32
       |
       +------------------------------------------------AT1G66430
```

Phenylalanine Ammonia Lyase - NJ

```
                          +------Pt10868254
                   +500.0-|
            +493.0-|      +------pg9 56 74
            |      |
            |      +-------------Os02g41670
            |
            +-------------------AT2G37040
     +261.0-|
     |      |             +------AT3G10340
     |      +-------401.0-|
     |      |             +------AT5G04230
     |      |
+------|      +-------------------AT3G53260
|      |
|      |             +-------------Os04g43800
|      |             |
|      |             |      +------Os12g43520
|      +-------269.0-|-500.0-|
|                    |      +------pg6 94 26
|                    |
|                    +-------------Os02g41680
|
+---------------------------------Os02g41650
```

Phenylalanine Ammonia Lyase - PARS

```
                    +------Os12g43520
              +500.0-|
              |      +------pg6 94 26
       +252.5-|
       |      |      +------pg9 56 74
       |      +500.0-|
       |             +------Pt10868254
       |
       +-------------------AT3G10340
       |
       +-------------------AT2G37040
+------|
|      +-------------------Os02g41670
|      |
|      |             +------Os02g41650
|      |             |
|      +-------342.6-|------Os02g41680
|      |             |
|      |             +------Os04g43800
|      |
|      +-------------------AT3G53260
|
+--------------------------AT5G04230
```

Phosphatase 2C - NJ

```
                                                                             +------Os02g15594
                                                                      +459.0-|
                                                               +428.0-|      +------AT5G53140
                                                               |      |
                                                        +460.0-|      +-------------Os05g50970
                                                        |      |
                                                        |      |             +------Pp1913517
                                                 +500.0-|      +-------500.0-|
                                                 |      |                    +------pg11 76 33
                                                 |      |
                                          +376.0-|      +---------------------------Pt26666 26
                                          |      |
                                          |      |                           +------Pt71730 31
                                          |      |                    +285.0-|
                                          |      +--------------500.0-|      +------pg9 47 78
                                          |                           |
                                   +500.0-|                           +-------------Pp6913519
                                   |      |
                                   |      |                                  +------Os02g05630
                                   |      |                                  |
                                   |      |                                  +------AT4G31750
                                   |      |                                  |
                                   |      |                                  +------Os06g48300
                            +481.0-|      +----------------------------374.0-|
                            |      |                                         +------Os04g56450
                            |      |                                         |
                            |      |                                         +------AT5G24940
                            |      |                                         |
                            |      |                                         +------AT5G10740
                            |      |
                            |      +------------------------------------------------AT1G43900
                            |
                            |                                                +------AT1G72770
                            |                                         +272.0-|
                            |                                  +433.0-|      +------AT1G17550
                     +494.0-|                                  |      |
                     |      |                           +499.0-|      +-------------Os05g46040
                     |      |                           |      |
                     |      |                    +262.0-|      +--------------------Os01g40094
                     |      |                    |      |
                     |      |             +445.0-|      +---------------------------Pt6408 35
                     |      |             |      |
                     |      |             |      +----------------------------------Pt41482550
                     |      |      +388.0-|
                     |      |      |      +-----------------------------------------Os09g15670
                     |      |      |      |
                     |      |      |      +-----------------------------------------Os01g46760
                     |      +500.0-|
                     |             |                              +-------------AT5G59220
                     |             |                       +500.0-|
              +496.0-|             |                       |      |      +------AT1G07430
              |      |             +---------------------395.0-|      +436.0-|
              |      |                                     |             +------AT2G29380
              |      |                                     |
              |      |                                     +--------------------Pt38004 20
              |      |
              |      |                                            +-------------Os07g32380
              |      +------------------------------------------272.0-|
              |      |                                            |      +------AT2G20630
              |      |                                            +500.0-|
       +356.0-|      |                                                   +------AT4G28400
       |      |      |
       |      |      |                                                   +------pg11 124 8
       |      |      |                                            +479.0-|
       |      |      |                                     +296.0-|      +------Pt13111 35
       |      |      |                                     |      |
+------|      |      +-----------------------------------256.0-|      +-------------Pt28599 23
|      |      |                                            |
|      |      |                                            +--------------------Os04g52000
|      |      |
|      |      +---------------------------------------------------------------------AT1G34750
|      |
|      +----------------------------------------------------------------------------AT1G22280
|
+-----------------------------------------------------------------------------------Os04g37904
```

Phosphatase 2C - PARS

```
                                                                                           +------Os02g05630
                                                                                           |
                                                                                           +------Os04g56450
                                                                                           |
                                                                                           +------Os06g48300
                                                               +---------------------363.7-|
                                                               |                           +------AT4G31750
                                                               |                           |
                                                               |                           +------AT5G24940
                                                               |                           |
                                                               |                           +------AT5G10740
                                                               |
                                                        +297.4-|             +--------------------Os02g15594
                                                        |      |             |
                                                        |      |             |             +------pg11 76 33
                                                        |      |             |      +499.8-|
                                                        |      |      +394.9-|-298.6-|      +------Pp1913517
                                                        |      |      |      |      |
                                                        |      |      |      |      +-------------AT5G53140
                                                 +474.7-|      +490.2-|      |
                                                 |      |             |      +--------------------Os05g50970
                                                 |      |             |
                                                 |      |             +---------------------------Pt26666 26
                                                 |      |
                            +---------------------301.1-|      |                                  +------Pp6913519
                            |                    |      |                                  |
                            |                    |      +----------------------------355.0-|------Pt71730 31
                            |                    |                                         |
                            |                    |                                         +------pg9 47 78
                            |                    |
                            |                    +------------------------------------------------AT1G43900
                            |
                            |                                                +--------------------Os01g40094
                            |                                         +476.9-|
                            |                                         |      |      +-------------Os05g46040
                            |                                         |      +446.4-|
                     +263.6-|                                  +375.3-|             |      +------AT1G72770
                     |      |                                  |      |             +461.4-|
                     |      |                                  |      |                    +------AT1G17550
                     |      |                           +347.9-|      |
                     |      |                           |      |      +---------------------------Os09g15670
                     |      |                    +280.3-|      |
                     |      |                    |      |      +----------------------------------Os01g46760
                     |      |             +492.0-|      |
                     |      |             |      |      +-----------------------------------------Pt6408 35
                     |      |      +450.7-|      |
              +276.2-|      |      |      |      +------------------------------------------------Pt41482550
              |      |      |      |      |
              |      |      +500.0-|      +-------------------------------------------------------Pt38004 20
              |      |             |
              |      |             |                                                       +------AT1G07430
              |      |             |                                                +441.0-|
              |      |             +------------------------------------------499.2-|      +------AT2G29380
              |      |                                                              |
              |      |                                                              +-------------AT5G59220
              |      |
              |      |                                                              +-------------Os07g32380
              |      +--------------------------------------------------------420.1-|
       +262.5-|                                                                     |      +------AT4G28400
       |      |                                                                     +500.0-|
       |      |                                                                            +------AT2G20630
       |      |
       |      |                                                                            +------AT1G34750
       |      |                                                                            |
       |      +----------------------------------------------------------------------463.5-|------AT1G22280
+------|      |                                                                            |
|      |      |                                                                            +------Os04g37904
|      |      |
|      |      +-----------------------------------------------------------------------------------Pt28599 23
|      |      |
|      |      +-----------------------------------------------------------------------------------Os04g52000
|      |
|      +------------------------------------------------------------------------------------------Pt13111 35
|
+-------------------------------------------------------------------------------------------------pg11 124 8
```

Phosphatase-serine/threonine protein phosphatase - NJ

```
                                   +------Os06g37660
                                   |
                            +494.0-|------Pt40759 37
                            |      |
                            |      +------Os02g12580
                     +362.0-|
                     |      |      +------AT1G69960
                     |      |      |
                     |      |      +------AT1G59830
                     |      +434.0-|
                     |             +------AT1G10430
                     |             |
                     |             +------Pt76594 31
                     |
       +-------500.0-|             +------Pp12057 11
       |             |             |
       |             |             +------pg 7 117 5
       |             |             |
       |             |             +------AT2G42500
       |             |      +497.0-|
       |             |      |      +------Os03g59060
       |             |      |      |
       |             |      |      +------Pt68453 19
       |             +253.0-|      |
       |                    |      +------AT3G58500
       |                    |
       |                    |      +------Os10g27050
       |                    +500.0-|
       |                           +------Os03g07150
       |
       |                           +------Pt35855 26
       |                           |
       |                           +------AT4G26720
       +---------------------274.0-|
+------|                           +------Pt35857 20
|      |                           |
|      |                           +------pg3 3 64 2
|      |
|      |                           +------pg 6 25 81
|      |                    +391.0-|
|      |                    |      +------Pt71598 13
|      |             +271.0-|
|      |             |      |      +------Pp32 9
|      |             |      +492.0-|
|      |             |             +------Pt81082554
|      |      +325.0-|
|      |      |      +--------------------Os02g13110
|      |      |      |
|      +500.0-|      |             +------Os06g36770
|      |      |      +-------256.0-|
|      |      |                    +------AT1G51690
|      |      |
|      |      +---------------------------AT1G17720
|      |
|      +----------------------------------AT5G55260
|
+-----------------------------------------Os09g11230
```

Phosphatase-serine/threonine protein phosphatase - PARS

```
                                                        +------Os10g27050
                                                 +500.0-|
                                                 |      +------Os03g07150
                                                 |
                                                 |      +------pg 7 117 5
       +-----------------------------------305.2-|      |
       |                                         |      +------AT2G42500
       |                                         |      |
       |                                         |      +------Pt68453 19
       |                                         +496.0-|
       |                                                +------Pp12057 11
       |                                                |
       |                                                +------Os03g59060
       |                                                |
       |                                                +------AT3G58500
       |
       |                                                +------Pt71598 13
       |                                         +493.3-|
       |                                         |      +------pg 6 25 81
       |                                  +456.0-|
       |                                  |      |      +------Pp32 9
       |                           +331.1-|      +489.5-|
       |                           |      |             +------Pt81082554
       |                    +342.5-|      |
       |                    |      |      +--------------------Os02g13110
       |             +290.4-|      |
       |             |      |      +---------------------------AT1G51690
       |      +500.0-|      |
       |      |      |      +----------------------------------Os06g36770
+------|      |      |
|      |      |      +-----------------------------------------AT1G17720
|      |      |
|      +290.0-|                                         +------AT4G26720
|      |      |                                         |
|      |      |                                         +------pg3 3 64 2
|      |      |                                  +357.6-|
|      |      |                                  |      +------Pt35857 20
|      |      |                                  |      |
|      |      +----------------------------340.0-|      +------Pt35855 26
|      |                                         |
|      |                                         +-------------AT5G55260
|      |                                         |
|      |                                         +-------------Os09g11230
|      |
|      +-------------------------------------------------------Os02g12580
|      |
|      +-------------------------------------------------------Os06g37660
|      |
|      +-------------------------------------------------------AT1G69960
|      |
|      +-------------------------------------------------------AT1G59830
|      |
|      +-------------------------------------------------------Pt76594 31
|      |
|      +-------------------------------------------------------AT1G10430
|
+--------------------------------------------------------------Pt40759 37
```

Pinoresinol-lariciresinol reductase - NJ

```
+--------------------------------------------------------------AT4G13660
|
|                                         +--------------------AT4G39230
|                                  +500.0-|
|                                  |      |      +-------------AT1G75290
|                                  |      +492.0-|
|                           +500.0-|             |      +------AT1G75300
|                           |      |             +363.0-|
|                           |      |                    +------AT1G75280
|                    +493.0-|      |
|                    |      |      +---------------------------Os06g27770
|                    |      |
|                    |      |                           +------Pt40554 20
|                    |      +---------------------500.0-|
|             +426.0-|                                  +------pg2 16 20
|             |      |
|             |      |                                  +------pg3 3 20
|             |      |                           +500.0-|
|             |      |                           |      +------Pp1528 15
|      +500.0-|      +---------------------323.0-|
|      |      |                                  |      +------pg8 87 14
|      |      |                                  +500.0-|
|      |      |                                         +------Pt39129 20
+------|      |
       |      |                                         +------Os12g16410
       |      +-----------------------------------482.0-|
       |                                                +------Os12g16220
       |
       +-------------------------------------------------------AT1G32100
```

Pinoresinol-lariciresinol reductase - PARS

```
                                                        +------Pt39129 20
                                                 +439.7-|
                                                 |      +------pg8 87 14
              +----------------------------293.0-|
              |                                  |      +------Pp1528 15
              |                                  +498.0-|
              |                                         +------pg3 3 20
              |
              |             +----------------------------------Os12g16220
              |             |
       +500.0-|             |             +--------------------AT4G39230
       |      |             |      +495.3-|
       |      |             |      |      |      +-------------AT1G75300
       |      |             |      |      +486.3-|
       |      |      +360.3-|-500.0-|              |      +------AT1G75290
       |      |      |      |      |             +443.8-|
       |      |      |      |      |                    +------AT1G75280
       |      |      |      |      |
+------|      +357.6-|      |      +---------------------------Os06g27770
|      |             |      |
|      |             |      +----------------------------------Os12g16410
|      |             |
|      |             |                                  +------AT4G13660
|      |             +-----------------------------500.0-|
|      |                                                +------AT1G32100
|      |
|      +-------------------------------------------------------Pt40554 20
|
+--------------------------------------------------------------pg2 16 20
```

Plasma membrane intrinsic protein - NJ

```
                            +--------------------pg 9 23 27
                            |
                            +--------------------pg 9 25 59
                            |
                            |      +-------------Pt67982556
                            +267.0-|
                            |      |      +------Pt9982549
                     +495.0-|      +477.0-|
                     |      |             +------pg 9 20 19
                     |      |
                     |      +--------------------Pp12189 18
                     |      |
                     |      +--------------------Pt21582555
                     |      |
                     |      +--------------------Pt84382554
              +499.0-|
              |      |             +-------------pg 11 12 1
              |      +-------500.0-|
              |      |             |      +------Pt95582552
              |      |             +494.0-|
              |      |                    +------Pp3409 15
              |      |
              |      |             +-------------Pt46298 26
              |      +-------496.0-|
              |                    |      +------pg 7 52 51
              |                    +477.0-|
              |                           +------Pt39268 16
              |
       +345.0-|                           +------AT4G35100
       |      |                    +479.0-|
       |      +--------------500.0-|      +------AT2G16850
       |      |                    |
       |      |                    +-------------Os04g16450
       |      |
       |      |                           +------AT5G60660
       |      |                    +341.0-|
+------|      +--------------289.0-|      +------Os02g41860
|      |      |                    |
|      |      |                    +-------------Os04g44060
|      |      |
|      |      +----------------------------------Os07g26630
|      |      |
|      |      +----------------------------------Os07g26690
|      |
|      +-----------------------------------------AT2G37170
|
+------------------------------------------------AT3G53420
```

Plasma membrane intrinsic protein - PARS

```
                                          +-------------Pp12189 18
                                          |
                                          |      +------pg 9 20 19
                                          +251.6-|
                                          |      +------Pt9982549
                                          |
                                          +-------------pg 9 25 59
                                   +494.3-|
                                   |      +-------------Pt67982556
                                   |      |
                                   |      +-------------pg 9 23 27
                                   |      |
              +--------------367.0-|      +-------------Pt84382554
              |                    |      |
              |                    |      +-------------Pt21582555
              |                    |
              |                    |             +------Pt39268 16
              |                    |      +495.3-|
              |                    +452.4-|      +------pg 7 52 51
              |                           |
              |                           +-------------Pt46298 26
              |
       +500.0-|                                  +------AT2G16850
       |      |                                  |
       |      |                           +498.4-|------AT4G35100
       |      |                           |      |
       |      |                    +326.7-|      +------Os04g16450
       |      |                    |      |
       |      |             +269.3-|      +-------------Os07g26690
       |      |             |      |
       |      |      +257.0-|      +--------------------AT3G53420
       |      |      |      |
+------|      |      |      +---------------------------AT2G37170
|      |      +500.0-|
|      |             |                           +------Os02g41860
|      |             |                    +336.0-|
|      |             |                    |      +------AT5G60660
|      |             +--------------263.6-|
|      |                                  +-------------Os07g26630
|      |                                  |
|      |                                  +-------------Os04g44060
|      |
|      |                                         +------Pp3409 15
|      +-----------------------------------494.3-|
|                                                +------Pt95582552
|
+-------------------------------------------------------pg 11 12 1
```

pentatricopeptide (PPR) repeat-containing protein -NJ

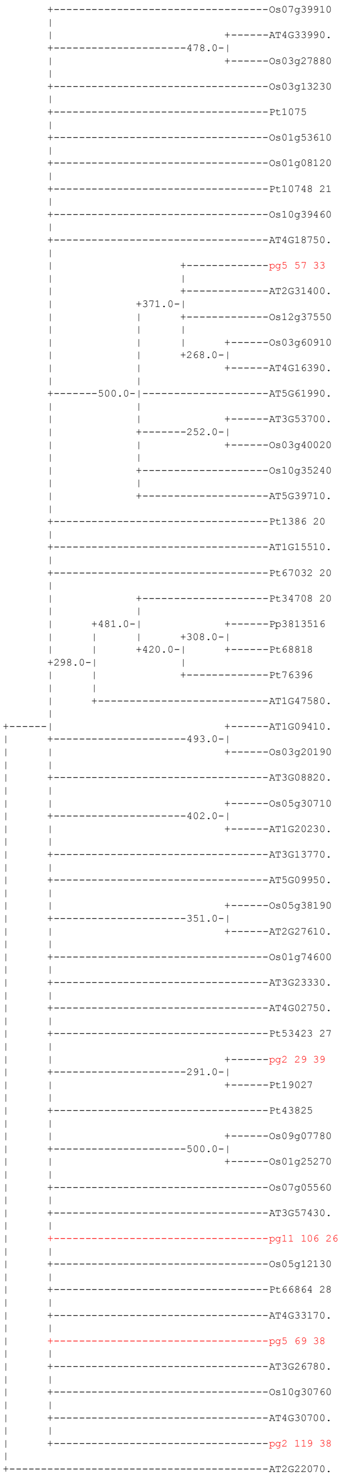

pentatricopeptide (PPR) repeat-containing protein - PARS

```
       +-----------------------------------------Os01g74600
       |
       |                                  +------AT1G20230.
       +----------------------------446.2-|
       |                                  +------Os05g30710
       |
       |                                  +------AT4G33990.
       +----------------------------485.3-|
       |                                  +------Os03g27880
       |
       |                                  +------Pt76396
       |                                  |
       |                           +344.4-|------Pt68818
       |                           |      |
       +---------------------500.0-|      +------Pp3813516
       |                           |
       |                           +-------------Pt34708 20
       |
       |                                  +------Os01g25270
       +----------------------------500.0-|
       |                                  +------Os09g07780
       |
       +-----------------------------------------AT4G30700.
       |
       +-----------------------------------------Os03g13230
       |
       +-----------------------------------------Os01g08120
       |
       |                                  +------Os03g40020
       |                           +499.0-|
       |                    +262.0-|      +------AT3G53700.
       |                    |      |
       |      +-------459.6-|      +-------------Os10g35240
       |      |             |
       |      |             |             +------AT5G61990.
       |      |             +-------431.0-|
       +500.0-|                           +------AT5G39710.
       |      |
       |      |      +--------------------------Os03g60910
       |      |      |
       |      +460.7-|             +-------------Os12g37550
       |             |      +499.0-|
       |             |      |      |      +------AT2G31400.
       |             +340.7-|      +386.6-|
       |                    |             +------pg5 57 33
       |                    |
       |                    +--------------------AT4G16390.
       |
       |                                  +------Pt19027
       +----------------------------262.7-|
       |                                  +------pg2 29 39
       |
       +-----------------------------------------AT4G33170.
       |
       |                                  +------AT1G09410.
       +----------------------------475.5-|
+------|                                  +------Os03g20190
|      |
|      +-----------------------------------------Pt66864 28
|      |
|      +-----------------------------------------Os10g30760
|      |
|      +-----------------------------------------AT3G57430.
|      |
|      +-----------------------------------------AT3G08820.
|      |
|      +-----------------------------------------Os07g05560
|      |
|      +-----------------------------------------Os01g53610
|      |
|      +-----------------------------------------AT3G23330.
|      |
|      +-----------------------------------------pg5 69 38
|      |
|      +-----------------------------------------AT3G13770.
|      |
|      +-----------------------------------------AT1G47580.
|      |
|      +-----------------------------------------Os07g39910
|      |
|      +-----------------------------------------pg211938
|      |
|      +-----------------------------------------AT3G26780.
|      |
|      +-----------------------------------------Pt10748 21
|      |
|      +-----------------------------------------AT5G09950.
|      |
|      +-----------------------------------------AT4G02750.
|      |
|      +-----------------------------------------AT2G22070.
|      |
|      +-----------------------------------------AT1G15510.
|      |
|      +-----------------------------------------AT4G18750.
|      |
|      +-----------------------------------------Pt1386 20
|      |
|      +-----------------------------------------Pt67032 20
|      |
|      +-----------------------------------------Pt1075
|      |
|      |                                  +------Os05g38190
|      +----------------------------433.1-|
|      |                                  +------AT2G27610.
|      |
|      +-----------------------------------------Pt43825
|      |
|      +-----------------------------------------pg11 106 26
|      |
|      +-----------------------------------------Os10g39460
|      |
|      +-----------------------------------------Pt53423 27
|
+------------------------------------------------Os05g12130
```

Potassium transporter - NJ

```
                                   +------Pt67272 17
                            +500.0-|
                     +435.0-|      +------pg 7 89 46
                     |      |
                     |      +-------------Pt56017 19
                     |
              +500.0-|             +------AT2G35060
              |      |      +498.0-|
              |      |      |      +------AT1G31120
              |      |      |
              |      |      +-------------Os09g38960
              |      +344.0-|
              |             +-------------Os04g52390
              |             |
              |             +-------------AT4G19960
              |             |
              |             +-------------Os08g10550
              |
              |                    +------Os07g47350
       +325.0-|--------------352.0-|
       |      |                    +------Os01g70940
       |      |
       |      +---------------------------Pt69617 20
       |      |
       |      |                    +------pg 6 81 12
       |      +--------------332.0-|
       |      |                    +------Pt58266 12
+------|      |
|      |      +---------------------------AT4G23640
|      |      |
|      |      +---------------------------AT2G40540
|      |      |
|      |      |                    +------AT1G70300
|      |      +--------------487.0-|
|      |                           +------AT5G14880
|      |
|      +----------------------------------AT3G02050
|
+-----------------------------------------Pt93282556
```

Potassium transporter - PARS

```
                                      +------AT1G31120
                               +438.4-|
                        +335.1-|      +------AT2G35060
                        |      |
                        |      +-------------AT4G19960
                        |
                 +372.2-|--------------------Os09g38960
                 |      |
                 |      +--------------------Os08g10550
                 |      |
                 |      +--------------------Os04g52390
          +500.0-|
          |      |                    +------pg 7 89 46
          |      +--------------500.0-|
          |      |                    +------Pt67272 17
          |      |
          |      +---------------------------Pt56017 19
          |
          +----------------------------------Pt69617 20
          |
          +----------------------------------AT4G23640
          |
          |                           +------AT5G14880
   +------|--------------------455.0-|
   |      |                           +------AT1G70300
   |      |
   |      |                           +------Os01g70940
   |      +--------------------274.9-|
   |      |                           +------Os07g47350
   |      |
   |      +----------------------------------AT3G02050
   |      |
   |      |                           +------pg 6 81 12
   |      +--------------------261.4-|
   |      |                           +------Pt58266 12
   |      |
   |      +----------------------------------AT2G40540
   |
   +-----------------------------------------Pt93282556
```

Protease FTSH – NJ

```
                                          +-------------pg 8 168 2
                                   +304.0-|
                                   |      |      +------AT2G30950
                            +396.0-|      +306.0-|
                            |      |             +------AT1G06430
                            |      |
                     +500.0-|      +--------------------Os06g45820
                     |      |
                     |      |                    +------AT5G15250
                     |      |             +497.0-|
              +376.0-|      +-------483.0-|      +------Os06g12370
              |      |                    |
              |      |                    +-------------Pt24208 14
       +491.0-|      |
       |      |      +----------------------------------pg 7 25 11
       |      |
+------|      +-----------------------------------------Pt2835 26
|      |
|      |                                         +------Os06g51029
|      +-----------------------------------256.0-|
|                                                +------AT1G50250
|
+-------------------------------------------------------AT5G42270
```

Protease FTSH - PARS

```
                                   +------AT5G15250
                            +475.8-|
              +-------373.2-|      +------Os06g12370
              |             |
       +499.9-|             +-------------Pt24208 14
       |      |
       |      |      +--------------------Os06g45820
       |      +435.3-|
       |             |      +-------------pg 8 168 2
       |             +430.9-|
+------|                    |      +------AT1G06430
|      |                    +262.1-|
|      |                           +------AT2G30950
|      |
|      |                           +------Pt2835 26
|      |                           |
|      |                           +------AT1G50250
|      +---------------------318.5-|
|                                  +------AT5G42270
|                                  |
|                                  +------Os06g51029
|
+-----------------------------------------pg 7 25 11
```

proton-dependent oligopeptide transport - NJ

```
                                                  +------pg10 123 3
       +-----------------------------------500.0-|
       |                                         +------Pt68035 15
       |
       |                                         +------pg8 31 36
       |                    +--------------500.0-|
       |                    |                    +------Pt10578255
       |                    |
       |                    |                    +------Os03g04570
       |             +451.0-|      +-------439.0-|
       |             |      |      |             +------Os10g33170
       |             |      |      |
       |             |      |      |             +------AT5G46040
       |             |      |      |      +480.0-|
       |             |      +254.0-|-286.0-|      +------AT5G46050
       |             |             |      |
       |             |             |      +-------------Pt24644 15
       |             |             |
       |      +445.0-|             |             +------AT2G40460
       |      |      |             +-------325.0-|
+------|      |      |                           +------Os10g33210
|      |      |      |
|      |      |      |                           +------AT5G01180
|      |      |      |                    +417.0-|
|      |      |      |             +357.0-|      +------AT3G54140
|      |      |      |             |      |
|      +411.0-|      |      +443.0-|      +-------------AT2G02040
|      |      |      |      |      |
|      |      |      +493.0-|      +--------------------Os01g04950
|      |      |             |
|      |      |             +---------------------------AT1G62200
|      |      |
|      |      |                                  +------Os05g34030
|      |      |                           +427.0-|
|      |      |                           |      +------Os05g34010
|      |      +---------------------370.0-|
|      |                                  |      +------Os05g33960
|      |                                  +261.0-|
|      |                                         +------Os05g34000
|      |
|      +------------------------------------------------AT1G68570
|
+-------------------------------------------------------Os06g15370
```

proton-dependent oligopeptide transport - PARS

```
                                                  +------Os05g33960
                                          +366.8-|
                                          |      +------Os05g34000
              +---------------------300.0-|
              |                           |      +------Os05g34010
              |                           +447.7-|
              |                                  +------Os05g34030
              |
              |                                  +------pg8 31 36
              |                    +-------500.0-|
              |                    |             +------Pt10578255
              |                    |
              |                    |             +------AT5G46050
       +304.9-|                    |      +435.9-|
       |      |                    +342.8-|      +------AT5G46040
       |      |                    |      |
       |      |      +-------369.2-|      +-------------Pt24644 15
       |      |      |             |
       |      |      |             +--------------------AT2G40460
       |      |      |             |
       |      |      |             |             +------Os10g33170
       |      |      |             +-------349.0-|
       |      |      |             |             +------Os03g04570
       |      |      |             |
       |      +398.9-|             +--------------------Os10g33210
       |             |
+------|             |                           +------AT3G54140
|      |             |                    +391.0-|
|      |             |             +269.4-|      +------AT5G01180
|      |             |             |      |
|      |             |      +387.4-|      +-------------AT2G02040
|      |             |      |      |
|      |             +377.4-|      +--------------------Os01g04950
|      |                    |
|      |                    +---------------------------AT1G62200
|      |
|      +------------------------------------------------AT1G68570
|      |
|      |                                         +------pg10 123 3
|      +-----------------------------------500.0-|
|                                                +------Pt68035 15
|
+-------------------------------------------------------Os06g15370
```

Pyruvate kinase - NJ

```
                                                               +------Os11g05110
                                                        +482.0-|
                                                        |      +------Os12g05110
                                                 +319.0-|
                                                 |      |      +------AT2G36580
                                                 |      +500.0-|
                                          +257.0-|             +------AT3G52990
                                          |      |
                                          |      |             +------pg 7 36 39
                                   +499.0-|      +-------440.0-|
                                   |      |                    +------Pt37176 39
                                   |      |
                                   |      |             +-------------Pt8225 38
                            +500.0-|      +-------500.0-|
                            |      |                    |      +------Pt35382548
                            |      |                    +467.0-|
                            |      |                           +------Pt40252 40
                            |      |
                            |      +----------------------------------Os11g10980
                            |
                            |             +---------------------------Os03g46910
                     +427.0-|             |
                     |      |      +500.0-|             +-------------AT3G22960
                     |      |      |      |      +434.0-|
                     |      |      |      |      |      |      +------Pt14207 32
                     |      |      |      +495.0-|      +500.0-|
                     |      |      |             |             +------pg 7 26 41
                     |      |      |             |
                     |      |      |             +--------------------Os07g08340
                     |      +500.0-|
              +405.0-|             |                           +------AT1G32440
              |      |             |                           |
              |      |             |                    +273.0-|------Os01g47080
              |      |             |                    |      |
              |      |             |             +292.0-|      +------AT5G52920
              |      |             |             |      |
              |      |             +-------500.0-|      +-------------Pt23468 46
       +493.0-|      |                           |
       |      |      |                           +--------------------Os10g42100
       |      |      |
       |      |      +------------------------------------------------AT3G04050
       |      |
       |      |                                                +------AT5G56350
       |      |                                         +481.0-|
+------|      +-----------------------------------444.0-|      +------AT4G26390
|      |                                                |
|      |                                                +-------------Os01g16960
|      |
|      |                                         +--------------------AT5G08570
|      |                                         |
|      |                                         |      +-------------pg 8 33 41
|      |                                         +296.0-|
|      +-----------------------------------437.0-|      |      +------Pt34682553
|                                                |      +500.0-|
|                                                |             +------Pp13416 14
|                                                |
|                                                +--------------------AT5G63680
|
+---------------------------------------------------------------------Os04g58110
```

Pyruvate kinase - PARS

```
                                                               +------Pt35382548
                                                        +496.6-|
                                                 +492.6-|      +------Pt40252 40
                                                 |      |
                                                 |      +-------------Pt8225 38
                                          +292.7-|
                                          |      |      +-------------Os11g05110
                                          |      |      |
                                          |      +399.0-|-------------Os12g05110
                                          |             |
                                   +500.0-|             |      +------AT2G36580
                                   |      |             +461.8-|
                                   |      |                    +------AT3G52990
                                   |      |
                            +500.0-|      +---------------------------pg 7 36 39
                            |      |      |
                            |      |      +---------------------------Pt37176 39
                            |      |
                            |      +----------------------------------Os11g10980
                            |
                            |                                  +------Pt14207 32
                            |                           +500.0-|
                     +482.8-|                           |      +------pg 7 26 41
                     |      |                    +305.9-|
                     |      |                    |      |      +------Os07g08340
                     |      |      +-------500.0-|      +304.8-|
                     |      |      |             |             +------Os03g46910
                     |      |      |             |
                     |      |      |             +--------------------AT3G22960
                     |      |      |
                     |      +500.0-|                           +------AT5G52920
              +256.5-|             |                    +373.7-|
              |      |             |             +331.3-|      +------Pt23468 46
              |      |             |             |      |
              |      |             |      +355.8-|      +-------------Os01g47080
              |      |             |      |      |
              |      |             +500.0-|      +--------------------AT1G32440
              |      |                    |
       +308.5-|      |                    +---------------------------Os10g42100
       |      |      |
       |      |      |                                  +-------------Os01g16960
       |      |      +----------------------------494.7-|
       |      |                                         |      +------AT4G26390
       |      |                                         +488.3-|
       |      |                                                +------AT5G56350
       |      |
       |      +-------------------------------------------------------AT3G04050
+------|
|      +--------------------------------------------------------------AT5G63680
|      |
|      +--------------------------------------------------------------AT5G08570
|      |
|      |                                                       +------Pp13416 14
|      +-------------------------------------------------466.1-|
|      |                                                       +------Pt34682553
|      |
|      +--------------------------------------------------------------pg 8 33 41
|
+---------------------------------------------------------------------Os04g58110
```

Quercetin 3-O-methyltransferase - NJ

```
                                                        +------AT1G77520
                                                 +449.0-|
                                                 |      +------AT1G77530
                                          +500.0-|
                                          |      +-------------AT1G63140
                                          |      |
                                   +303.0-|      +-------------AT5G53810
                                   |      |
                                   |      |             +------Os04g09654
                                   |      |      +480.0-|
                                   |      +500.0-|      +------Os04g09604
                                   |             |
                                   |             +-------------Os04g01470
                                   |
                                   |             +-------------pg10 24 45
                                   |      +274.0-|
                                   |      |      |      +------pg10 23 23
                                   |      |      +317.0-|
                            +343.0-|-499.0-|             +------Pt6594 22
                            |      |      |
                            |      |      |      +-------------Pt74182554
                            |      |      +378.0-|
                            |      |             |      +------pg10 26 67
                            |      |             +421.0-|
                            |      |                    +------Pt4803 24
                     +378.0-|      |
                     |      |      |                    +------Os08g06100
                     |      |      +--------------403.0-|
                     |      |      |                    +------AT5G54160
              +472.0-|      |      |
              |      |      |      +---------------------------AT1G33030
              |      |      |
       +363.0-|      |      +----------------------------------AT1G51990
       |      |      |
       |      |      +-----------------------------------------AT3G53140
+------|      |
|      |      +------------------------------------------------AT4G35160
|      |
|      +-------------------------------------------------------pg2 68 65
|
+--------------------------------------------------------------Pt11735 26
```

Quercetin 3-O-methyltransferase - PARS

```
+----------------------------------------------------------------------------pg2 68 65
|
|                                                +---------------------------AT1G33030
|                                                |
|                                                |                    +------Pt74182554
|                                                |                    |
|                                                |      +-------255.2-|------pg10 26 67
|                                                |      |             |
|                                                +484.1-|             +------Pt4803 24
|                                         +325.8-|      |
|                                         |      |      |      +-------------pg10 24 45
|                                         |      |      +316.0-|
|                                         |      |             |      +------pg10 23 23
|                                         |      |             +326.8-|
|                                         |      |                    +------Pt6594 22
|                                         |      |
|                                  +276.5-|      |                    +------Os08g06100
|                                  |      |      +--------------418.1-|
|                                  |      |                           +------AT5G54160
|                                  |      |
|                                  |      |                           +------AT1G77530
|                                  |      |                    +404.3-|
|                                  |      |             +316.4-|      +------AT1G77520
|                           +427.7-|      |             |      |
|                           |      |      +-------500.1-|      +-------------AT1G63140
|                           |      |                    |
|                           |      |                    +--------------------AT5G53810
|                           |      |
|                    +281.9-|      |                                  +------Os04g09654
|                    |      |      |                           +492.1-|
|                    |      |      +---------------------500.1-|      +------Os04g09604
|                    |      |                                  |
|             +451.8-|      |                                  +-------------Os04g01470
|             |      |      |
|             |      |      +------------------------------------------------AT1G51990
|      +414.5-|      |
|      |      |      +-------------------------------------------------------AT3G53140
+------|      |
       |      +--------------------------------------------------------------AT4G35160
       |
       +---------------------------------------------------------------------Pt11735 26
```

Rab2-like GTP-binding protein - NJ

```
+-----------------------------------------Pp12019 20
|
|                                  +------Os10g14150
|             +--------------299.0-|
|             |                    +------AT4G17160
|             |
|             |      +--------------------AT4G35860
|             +273.0-|
|             |      |      +-------------AT5G59150
|             |      +500.0-|
|      +270.0-|             |      +------AT1G16920
|      |      |             +277.0-|
|      |      |                    +------AT5G45750
|      |      |
|      |      |                    +------pg 8 128 2
|      |      +--------------491.0-|
|      |      |                    +------Pt7972 28
|      |      |
|      |      +---------------------------Pt60324 46
|      |
+------|----------------------------------Pt44119 19
       |
       +----------------------------------Os02g37420
       |
       +----------------------------------Os04g39440
       |
       +----------------------------------pg 1 3 54
       |
       +----------------------------------Pt61685 23
       |
       +----------------------------------AT4G17170
```

Rab2-like GTP-binding protein - PARS

```
                                   +------AT1G16920
                            +482.5-|
                     +500.0-|      +------AT5G45750
                     |      |
              +448.0-|      +-------------AT5G59150
              |      |
              |      +--------------------Os10g14150
       +442.0-|
       |      +---------------------------pg 8 128 2
       |      |
       |      +---------------------------Pt7972 28
       |
       +----------------------------------Os04g39440
       |
       +----------------------------------Pt44119 19
       |
       +----------------------------------Pp12019 20
+------|
|      +----------------------------------Os02g37420
|      |
|      +----------------------------------AT4G35860
|      |
|      +----------------------------------AT4G17170
|      |
|      +----------------------------------AT4G17160
|      |
|      +----------------------------------Pt60324 46
|      |
|      +----------------------------------pg 1 3 54
|
+-----------------------------------------Pt61685 23
```

Ran-binding protein - NJ

```
                                    +------pg 2 27 73
                            +311.0-|
                     +499.0-|      +------Pt24032 25
                     |      |
       +-------500.0-|      +-------------Pt24031 7
       |             |
       |             |             +------AT4G11790
       |             +-------431.0-|
       |                           +------Os03g46480
       |
       |                           +------Pt382547 1
       |                           |
       |                    +360.0-|------Pt525 12
       |                    |      |
       |             +494.0-|      +------Pt64660 18
+------|             |      |
|      |      +379.0-|      +-------------Pp2583 22
|      |      |      |
|      +467.0-|      +--------------------pg 10 74 2
|      |      |
|      |      |                    +------Os03g18180
|      |      +--------------348.0-|
|      |                           +------Os05g28290
|      |
|      +----------------------------------AT2G30060
|      |
|      +----------------------------------AT5G58590
|
+-----------------------------------------AT1G07140
```

Ran-binding protein - PARS

```
                                   +------Pp2583 22
                                   |
                                   +------Pt525 12
                            +297.9-|
                            |      +------Pt382547 1
              +-------365.5-|      |
              |             |      +------Pt64660 18
              |             |
              |             +-------------pg 10 74 2
              |
              |             +-------------Pt24031 7
       +370.5-|             |
       |      |             |      +------Os03g46480
       |      |             +382.7-|
       |      |      +500.0-|      +------AT4G11790
       |      |      |      |
       |      |      |      +-------------pg 2 27 73
       |      |      |      |
+------|      +255.9-|      +-------------Pt24032 25
|      |             |
|      |             |             +------AT5G58590
|      |             |             |
|      |             +-------320.5-|------AT1G07140
|      |                           |
|      |                           +------AT2G30060
|      |
|      +----------------------------------Os03g18180
|
+-----------------------------------------Os05g28290
```

Rapid alkalinization factor - NJ

```
                                                  +------pg 12 98 2
                                          +269.0-|
                            +-------500.0-|      +------Pt16366 9
                            |             |
                            |             +-------------Pt63144 7
                     +300.0-|
                     |      |      +--------------------Os01g10470
                     |      |      |
                     |      +344.0-|             +------Os01g15320
                     |             |      +465.0-|
                     |             +471.0-|      +------Os01g25540
                     |                    |
                     |                    +-------------Os12g35670
              +320.0-|
              |      |                           +------Pt11654 6
              |      |                    +474.0-|
              |      +--------------500.0-|      +------Pp9451 6
              |      |                    |
       +494.0-|      |                    +-------------pg3 3 17 3
       |      |      |
       |      |      |                           +------AT1G28270
       |      |      +---------------------499.0-|
+------|      |                                  +------AT2G33775
|      |      |
|      |      +-----------------------------------------AT1G02900
|      |
|      |                                         +------AT4G15800
|      +-----------------------------------489.0-|
|                                                +------AT3G16570
|
+-------------------------------------------------------AT3G05490
```

Rapid alkalinization factor - PARS

```
                                                        +------Pt16366 9
                                                 +347.1-|
                                   +-------500.0-|      +------Pt63144 7
                                   |             |
                                   |             +-------------pg 12 98 2
                                   |
                            +461.6-|                    +------Os12g35670
                            |      |             +278.8-|
                            |      |      +358.4-|      +------Os01g15320
                            |      |      |      |
                            |      +285.1-|      +-------------Os01g25540
                            |             |
                     +456.8-|             +--------------------Os01g10470
                     |      |
                     |      |                           +------Pt11654 6
                     |      |                    +498.3-|
                     |      |             +499.0-|      +------Pp9451 6
                     |      |             |      |
              +415.4-|      +-------302.6-|      +-------------pg3 3 17 3
              |      |                    |
              |      |                    |             +------AT2G33775
              |      |                    +-------500.0-|
       +497.0-|      |                                  +------AT1G28270
       |      |      |
       |      |      +-----------------------------------------AT1G02900
+------|      |
|      |      +------------------------------------------------AT3G05490
|      |
|      +-------------------------------------------------------AT4G15800
|
+--------------------------------------------------------------AT3G16570
```

Ras-related GTP-binding protein - NJ

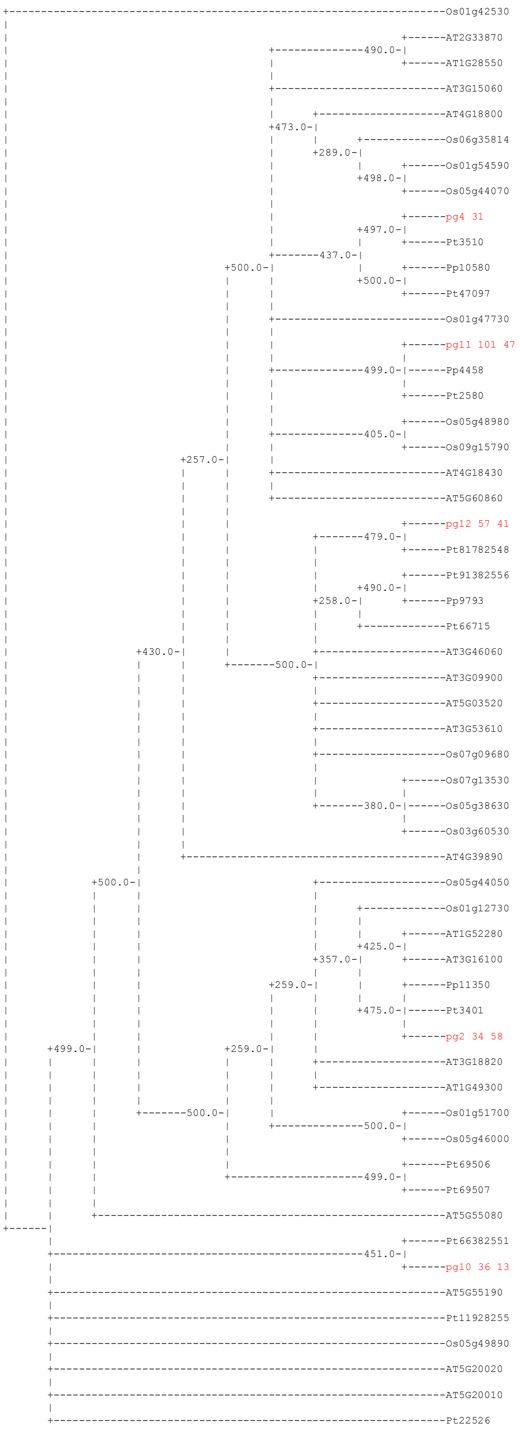

Ras-related GTP-binding protein - PARS

```
                                                        +-------------Pp4458
                                          +-------456.5-|
                                          |             |      +------Pt2580
                                          |             +-255.5-|
                                          |                    +------pg 11 101 47
                                          |
                                          |                    +------pg 4 4 31
                                   +254.0-|             +-480.0-|
                                   |      |             |      +------Pt3510
                                   |      |      +400.0-|
                                   |      |      |      |      +------Pt47097
                                   |      |      |      +-98.7-|
                                   |      |      |             +------Pp10580
                                   |      +-402.0|
                                   |             |             +------Os06g35814
                                   |             |      +-64.2-|
                                   |             |      |      +------AT4G18800
                                   |             +-452.0|
                                   |                    |      +------Os05g44070
                                   |                    +480.0-|
                                   |                           +------Os01g54590
                            +-500.0-|
                            |      +----------------------------------AT4G18430
                            |      |
                            |      +----------------------------------Os05g48980
                            |      |
                            |      +----------------------------------AT5G60860
                            |      |
                            |      +----------------------------------Os01g47730
                            |      |
                            |      +----------------------------------AT3G15060
                            |      |
                            |      +----------------------------------Os09g15790
                            |      |
                            |      |                     +------AT2G33870
                            |      +---------------------444.0-|
                            |                                  +------AT1G28550
                            |
                            |                                  +------Pt81782548
                            |                    +-------480.0-|
                            |                    |             +------pg12 57 41
                            |                    |
                     +409.0-|                    +--------------------AT3G53610
                     |      |                    |
                     |      |                    |             +------AT5G03520
                     |      |                    +-------392.0-|
                     |      |                    |             +------AT3G09900
                     |      |                    |
                     |      |                    |             +------Pt91382556
                     |      +--------------500.0-|     +404.0-|
                     |      |                    +310.0-|      +------Pp9793
                     |      |                    |      |
                     |      |                    |      +-------------Pt66715
                     |      |                    |
                     |      |                    +--------------------AT3G46060
                     |      |                    |
                     |      |                    +--------------------Os07g09680
              +-500.0|      |                    |
              |      |      |                    |             +------Os07g13530
              |      |      |                    |      +347.0-|
              |      |      |                    +363.5-|      +------Os03g60530
              |      |      |                           |
              |      |      |                           +-------------Os05g38630
              |      |      |
              |      |      +-----------------------------------------AT4G39890
              |      |
              |      |                                         +------Os01g51700
              |      |                    +--------------500.0-|
              |      |                    |                    +------Os05g46000
              |      |                    |
              |      |                    |                    +------Pt69506
              |      |                    |      +-------500.0-|
              |      |                    |      |             +------Pt69507
              |      +--------------500.0-|       |
       +495.0-|                           |      |      +-------------Os01g12730
       |      |                           |      +346.5-|
       |      |                           |      |      |      +------AT3G16100
       |      |                           |      |      +392.0-|
       |      |                           |      |             +------AT1G52280
       |      |                           +438.0-|
       |      |                                  |             +------pg2 34 58
       |      |                                  |      +264.0-|
       |      |                                  +451.0-|      +------Pp11350
       |      |                                  |      |
       |      |                                  |      +-------------Pt3401
       |      |                                  |
       |      |                                  |             +------AT3G18820
       |      |                                  |      +381.5-|
       |      |                                  +330.5-|      +------AT1G49300
       |      |                                         |
       |      |                                         +-------------Os05g44050
+------|      |
|      |      +-------------------------------------------------------AT5G55080
|      |
|      |                                  +--------------------Pt11928255
|      |                                  |
|      +-----------------------------------353.0-|             +------pg10 36 13
|      |                                  |      +399.0-|
|      |                                  +362.0-|      +------Pt66382551
|      |                                         |
|      |                                         +-------------Pt22526
|      |
|      +--------------------------------------------------------------Os05g49890
|      |
|      |                                                 +------AT5G20010
|      +-------------------------------------------------287.0-|
|      |                                                 +------AT5G20020
|      |
|      +--------------------------------------------------------------AT5G55190
|
+---------------------------------------------------------------------Os01g42530
```

RNA recognition motif (RRM)- containing protein - NJ

```
                                                 +------pg3 122 14
                     +---------------------500.0-|
                     |                           +------Pp1084 12
                     |
                     +----------------------------------AT3G46020
                     |
                     |                           +------AT2G41060
                     |                    +500.0-|
                     |             +427.0-|      +------AT3G56860
                     |             |      |
                     |             |      +-------------Os01g16090
                     |      +492.0-|
                     |      |      +--------------------Os04g54440
                     +462.0-|      |
                     |      |      +--------------------Os01g42820
                     |      |
                     |      +---------------------------AT3G15010
              +400.0-|
              |      +----------------------------------AT5G06210
              |      |
              |      +----------------------------------Os04g49440
              |      |
              |      |                           +------AT5G54580
              |      |                    +485.0-|
              |      |             +484.0-|      +------Os07g36490
              |      |             |      |
              |      +-------259.0-|      +-------------Os08g04440
              |      |             |
              |      |             +--------------------Os03g17060
       +494.0-|      |
       |      |      +----------------------------------AT1G73530
       |      |      |
       |      |      |                           +------Os02g49820
       |      |      +---------------------499.0-|
       |      |                                  +------AT3G20930
       |      |
       |      |                                  +------pg12 121 2
       |      |                           +492.0-|
       |      |                    +483.0-|      +------Pt9756 31
+------|      |                    |      |
|      |      |             +497.0-|      +-------------Os03g59710
|      |      |             |      |
|      |      +-------499.0-|      +--------------------AT5G12190
|      |                    |
|      |                    +---------------------------AT2G14870
|      |
|      |                           +--------------------AT1G76940
|      |                           |
|      |                           |             +------pg10 55 28
|      +---------------------265.0-|             |
|                                  |      +487.0-|------Pp8499 23
|                                  |      |      |
|                                  +348.0-|      +------Pt2682550
|                                         |
|                                         |      +------Os06g03690
|                                         +445.0-|
|                                                +------Os06g05530
|
+-------------------------------------------------------Os08g43360
```

RNA recognition motif (RRM)- containing protein - PARS

```
                                                        +--------------------Os03g17060
                                                        |
                                   +--------------366.5-|             +------AT5G54580
                                   |                    |      +251.6-|
                                   |                    +283.6-|      +------Os07g36490
                                   |                           |
                                   |                           +-------------Os08g04440
                                   |
                                   |             +---------------------------Os01g42820
                                   |             |
                                   |             |                    +------AT3G56860
                                   |      +459.1-|             +500.0-|
                                   |      |      |      +253.4-|      +------AT2G41060
                                   |      |      |      |      |
                                   +262.4-|      +439.1-|      +-------------Os01g16090
                                   |      |             |
                                   |      |             +--------------------Os04g54440
                                   |      |
                                   |      +----------------------------------AT3G15010
                                   |
                                   |                           +-------------Os03g59710
                                   |                           |
                                   |                    +439.3-|-------------AT5G12190
                            +495.8-|                    |      |
                            |      |                    |      |      +------Pt9756 31
                            |      +--------------500.0-|      +498.3-|
                            |      |                    |             +------pg12 121 2
                            |      |                    |
                            |      |                    +--------------------AT2G14870
                            |      |
                            |      |                                  +------AT3G20930
                            |      +----------------------------500.0-|
                            |      |                                  +------Os02g49820
                            |      |
                     +297.0-|      +-----------------------------------------AT1G73530
                     |      |      |
                     |      |      |                                  +------Pp1084 12
                     |      |      +----------------------------499.8-|
                     |      |      |                                  +------pg3 122 14
                     |      |      |
                     |      |      +-----------------------------------------AT3G46020
              +280.9-|      |      |
              |      |      |      +-----------------------------------------Os04g49440
              |      |      |      |
              |      |      |      +-----------------------------------------AT5G06210
              |      |      |
       +497.2-|      |      +------------------------------------------------Os08g43360
       |      |      |
       |      |      +-------------------------------------------------------AT1G76940
       |      |
       |      |                                                       +------Os06g05530
+------|      +-------------------------------------------------458.1-|
|      |                                                              +------Os06g03690
|      |
|      +---------------------------------------------------------------------pg10 55 28
|      |
|      +---------------------------------------------------------------------Pp8499 23
|
+----------------------------------------------------------------------------Pt2682550
```

sec23/sec24 transport family - NJ

```
                                                 +------Os12g24250
                                          +399.0-|
                                          |      +------Os11g29200
                                   +285.0-|
                                   |      |      +------AT3G44340
                                   |      +500.0-|
                            +500.0-|             +------AT4G32640
                            |      |
                            |      |             +------pg 4 50 43
                     +500.0-|      +-------482.0-|
                     |      |                    +------Pt14782554
                     |      |
              +270.0-|      |                    +------Os04g04020
              |      |      +--------------500.0-|
              |      |                           +------AT3G07100
       +448.0-|      |
       |      |      +----------------------------------Pt606 56
       |      |
+------|      +-----------------------------------------pg10 80 31
|      |
|      +------------------------------------------------AT2G27460
|      |
|      +------------------------------------------------Os05g37120
|
+-------------------------------------------------------Os04g33690
```

sec23/sec24 transport family - PARS

```
                                   +------Os11g29200
                            +398.6-|
                            |      +------Os12g24250
                            |
                            |      +------pg 4 50 43
                     +500.0-|-500.0-|
                     |      |      +------Pt14782554
                     |      |
                     |      |      +------AT3G44340
              +500.0-|      +500.0-|
              |      |             +------AT4G32640
              |      |
       +418.5-|      |             +------AT3G07100
       |      |      +-------500.0-|
       |      |                    +------Os04g04020
       |      |
       |      +---------------------------Os04g33690
+------|
|      |                           +------pg10 80 31
|      +---------------------494.0-|
|      |                           +------Pt606 56
|      |
|      +----------------------------------AT2G27460
|
+-----------------------------------------Os05g37120
```

Senescence-associated protein - NJ

```
                             --------------------Os04g58330
                            |
              +-------374.0-|             +------AT4G28770
              |             |      +410.0-|
              |             +273.0-|      +------AT2G20230
              |                    |
              |                    +-------------Os06g51480
              |
              |                    +-------------AT1G22160
              |                    |
              |                    |      +------pg6 137 19
              |             +489.0-|      |
              |             |      |      +------Os08g31510
              |             |      |      |
       +369.0-|             |      |      +------Pt96082551
       |      |             |      +299.0-|
       |      |      +480.0-|             +------AT1G78020
       |      |      |      |             |
       |      |      |      |             +------Os09g20240
       |      |      |      |             |
       |      +323.0-|      |             +------AT5G47060
       |      |      |      |
+------|      |      |      +--------------------Os02g37970
|      |      |      |
|      |      |      +---------------------------AT1G32400
|      |      |
|      |      |                           +------Pt45370 28
|      |      +---------------------428.0-|
|      |                                  +------pg4 59 25
|      |
|      +-----------------------------------------Os08g18044
|
+------------------------------------------------Os08g06250
```

Senescence-associated protein - PARS

```
                                                        +------pg4 59 25
                     +----------------------------493.9-|
                     |                                  +------Pt45370 28
                     |
                     |             +---------------------------AT5G47060
                     |             |
                     |             |                    +------Os09g20240
                     |             |             +497.6-|
                     |      +487.0-|             |      +------Os08g31510
                     |      |      |      +286.7-|
                     |      |      |      |      |      +------pg6 137 19
              +252.4-|      |      |      |      +500.0-|
              |      |      |      +467.6-|             +------Pt96082551
              |      +485.0-|             |
              |      |      |             |             +------AT1G22160
              |      |      |             +-------494.0-|
              |      |      |                           +------AT1G78020
              |      |      |
              |      |      +----------------------------------Os02g37970
       +269.7-|      |
       |      |      |                    +--------------------Os04g58330
       |      |      +--------------275.9-|
       |      |                           |      +-------------Os06g51480
       |      |                           +419.8-|
+------|      |                                  |      +------AT2G20230
|      |      |                                  +475.2-|
|      |      |                                         +------AT4G28770
|      |      |
|      |      +------------------------------------------------AT1G32400
|      |
|      +-------------------------------------------------------Os08g18044
|
+--------------------------------------------------------------Os08g06250
```

Serine carboxypeptidase S10 - NJ

```
                                                  +------Pt70216 17
                                   +-------360.0-|
                                   |              +------pg 5 19 14
                            +280.0-|
                            |      |             +------AT3G10450
                            |      |      +492.0-|
                            |      +500.0-|      +------AT2G22920
                            |             |
                            |             +-------------Os10g01110
                     +500.0-|
                     |      |                    +------AT3G25420
                     |      +--------------413.0-|
                     |      |                    +------AT4G12910
                     |      |
              +296.0-|      |                    +------Os12g15470
              |      |      +--------------456.0-|
              |      |                           +------Os04g25560
              |      |
              |      |                           +------AT4G30810
              |      +---------------------500.0-|
              |                                  +------Os06g08720
              |
              +-----------------------------------------AT3G07990
              |
              |                    +--------------------AT3G63470
              |                    |
              +--------------414.0-|             +------Pt53700 20
              |                    |      +301.0-|
              |                    +408.0-|      +------pg 1 40 38
              |                           |
              |                           +-------------pg 1 39 12
              |
              |                                  +------pg 8 17 33
              |                    +-------500.0-|
       +455.0-|                    |             +------Pt9379 25
       |      |             +500.0-|
       |      |             |      |             +------AT1G11080
       |      |             |      |      +303.0-|
       |      +-------389.0-|      +444.0-|      +------AT1G61130
       |      |             |             |
       |      |             |             +-------------AT4G15100
       |      |             |
       |      |             +---------------------------Os01g22980
       |      |
       |      +-----------------------------------------Os01g61690
       |      |
       |      +-----------------------------------------AT2G35770
       |      |
+------|      +-----------------------------------------Os01g06490
|      |      |
|      |      +-----------------------------------------Os05g18604
|      |      |
|      |      +-----------------------------------------Os02g42310
|      |      |
|      |      +-----------------------------------------AT2G35780
|      |
|      |                                         +------AT2G24010
|      +-----------------------------------478.0-|
|      |                                         +------AT2G24000
|      |
|      +------------------------------------------------AT4G30610
|      |
|      +------------------------------------------------AT3G02110
|
+-------------------------------------------------------Os02g55130
```

Serine carboxypeptidase S10 - PARS

```
                     +-----------------------------------------AT2G35770
                     |
                     |                    +--------------------AT3G63470
                     |                    |
                     +--------------413.9-|             +------Pt53700 20
                     |                    |      +428.5-|
                     |                    +444.8-|      +------pg 1 40 38
                     |                           |
                     |                           +-------------pg 1 39 12
                     |
                     |                                  +------Os05g18604
                     +----------------------------306.7-|
                     |                                  +------Os01g06490
                     |
                     |                                  +------AT3G10450
                     |                           +492.5-|
                     |             +-------491.0-|      +------AT2G22920
                     |             |             |
                     |             |             +-------------Os10g01110
                     |             |
                     |             |                    +------Os04g25560
                     |      +495.0-|             +500.0-|
                     |      |      |             |      +------Os12g15470
                     |      |      |      +317.9-|
                     |      |      |      |      |      +------AT4G12910
                     |      |      |      |      +447.5-|
                     |      |      +428.9-|             +------AT3G25420
                     +354.9-|             |
                     |      |             |             +------pg 5 19 14
              +265.3-|      |             +-------496.2-|
              |      |      |                           +------Pt70216 17
              |      |      |
              |      |      |                           +------Os06g08720
              |      |      +---------------------250.1-|
              |      |                                  +------AT4G30810
              |      |
              |      |             +---------------------------Os01g22980
              |      |             |
              |      +-------384.0-|                    +------Pt9379 25
              |      |             |      +-------500.0-|
              |      |             |      |             +------pg 8 17 33
              |      |             +492.1-|
              |      |                    |             +------AT4G15100
              |      |                    |      +266.1-|
       +288.9-|      |                    +412.5-|      +------AT1G11080
       |      |      |                           |
       |      |      |                           +-------------AT1G61130
       |      |      |
       |      |      +-----------------------------------------AT2G35780
       |      |      |
       |      |      +-----------------------------------------Os01g61690
       |      |      |
       |      |      +-----------------------------------------Os02g55130
+------|      |      |
|      |      |      +-----------------------------------------Os02g42310
|      |      |      |
|      |      |      +-----------------------------------------AT3G07990
|      |      |      |
|      |      |      +-----------------------------------------AT3G02110
|      |      |
|      |      +------------------------------------------------AT4G30610
|      |
|      +-------------------------------------------------------AT2G24010
|
+--------------------------------------------------------------AT2G24000
```

Short-chain dehydrogenase/reductase - NJ

```
                                          +----------------------------------Os11g30560
                                          |
                                          |                           +------Pt11908255
                                          +---------------------413.0-|
                                          |                           +------AT4G10020
                                          |
                                          |      +---------------------------AT3G47350
                                          |      |
                                   +390.0-|      |                    +------AT5G50700
                                   |      +260.0-|             +499.0-|
                                   |      |      |      +319.0-|      +------AT5G50600
                                   |      |      |      |      |
                                   |      |      +268.0-|      +-------------AT5G50770
                            +474.0-|      |             |
                            |      |      |             +--------------------Os12g27830
                            |      |      |
                            |      |      +----------------------------------Os02g30690
                            |      |
                            |      +-----------------------------------------Os04g32070
                            |
                            |                                  +-------------Os11g25160
                            |                                  |
                            +----------------------------500.0-|-------------AT2G29260
                            |                                  |
                            |                                  |      +------AT2G29370
                            |                                  +492.0-|
                            |                                         +------Os03g16230
                            |
                            |                                  +-------------Os04g52400
                            |                           +299.0-|
                     +463.0-|                           |      |      +------pg9 149 21
                     |      +---------------------500.0-|      +270.0-|
                     |      |                           |             +------Pt59166 22
                     |      |                           |
                     |      |                           +--------------------AT3G12800
                     |      |
                     |      +------------------------------------------------AT1G52340
                     |      |
                     |      |                                         +------AT3G50560
                     |      |                                  +407.0-|
                     |      +----------------------------493.0-|      +------pg3 115 19
                     |      |                                  |
                     |      |                                  +-------------Os08g37130
              +308.0-|      |
              |      |      |                                         +------Os04g33240
              |      |      +-----------------------------------487.0-|
              |      |      |                                         +------Os04g10010
              |      |      |
              |      |      +------------------------------------------------Os03g59610
              |      |
              |      |                                                +------Pt25323 18
              |      |                                  +-------421.0-|
              |      |                                  |             +------Pt69457 20
              |      |                           +483.0-|
              |      |                           |      |      +-------------pg12 113 3
              |      |                           |      +307.0-|
              |      |                           |             |      +------pg12 112 4
              |      +---------------------460.0-|             +500.0-|
              |                                  |                    +------Pt24624 17
              |                                  |
              |                                  |             +-------------pg8 9 36
       +424.0-|                                  +-------423.0-|
       |      |                                                |      +------Pt23982550
       |      |                                                +443.0-|
       |      |                                                       +------Pt46854 20
       |      |
       |      |                                                       +------Os03g18740
       |      +-------------------------------------------------497.0-|
       |      |                                                       +------Os07g49120
       |      |
       |      |                                                       +------Os07g46930
+------|      |                                                +497.0-|
|      |      |                                                |      +------Os07g40250
|      |      +------------------------------------------302.0-|
|      |      |                                                |      +------AT2G47120
|      |      |                                                +487.0-|
|      |      |                                                       +------AT3G29250
|      |      |
|      |      +--------------------------------------------------------------AT3G51680
|      |      |
|      |      +--------------------------------------------------------------AT3G26770
|      |
|      +---------------------------------------------------------------------AT4G03140
|
+----------------------------------------------------------------------------AT3G26760
```

Short-chain dehydrogenase/reductase - PARS

```
                                                  +------Os11g25160
                                          +256.5-|
                                          |      +------AT2G29260
                     +--------------490.7-|
                     |                    |      +------Os03g16230
                     |                    +486.2-|
                     |                           +------AT2G29370
                     |
                     |                           +------pg3 115 19
                     |                    +400.3-|
                     +--------------396.0-|      +------AT3G50560
                     |                    |
                     |                    +-------------Os08g37130
                     |
                     |                           +------Pt59166 22
                     |                    +259.4-|
                     |             +396.3-|      +------pg9 149 21
                     |             |      |
                     +-------500.0-|      +-------------Os04g52400
                     |             |
                     |             +--------------------AT3G12800
                     |
                     |                    +-------------AT5G50770
                     |             +391.4-|
                     |             |      |      +------AT5G50700
              +423.5-|      +410.6-|      +500.0-|
              |      |      |      |             +------AT5G50600
              |      |      |      |
              |      |      |      +--------------------AT3G47350
              |      |      |
              |      |      |                    +------Pt11908255
              |      |      |             +407.9-|
              |      +434.7-|             |      +------AT4G10020
              |      |      +-------270.6-|
              |      |      |             +-------------Os11g30560
              |      |      |             |
              |      |      |             +-------------Os02g30690
              |      |      |
              |      |      |                    +------Os04g32070
              |      |      +--------------437.2-|
              |      |                           +------Os12g27830
              |      |
              |      |                           +------Os04g10010
              |      +---------------------370.3-|
              |      |                           +------Os04g33240
              |      |
       +498.0-|      +----------------------------------Os03g59610
       |      |      |
       |      |      +----------------------------------AT1G52340
       |      |
       |      |                                  +------Os07g46930
       |      +----------------------------499.0-|
       |      |                                  +------Os07g40250
       |      |
       |      +-----------------------------------------AT3G26770
       |      |
       |      +-----------------------------------------AT3G26760
       |      |
       |      |                                  +------Os07g49120
+------|      +----------------------------489.5-|
|      |      |                                  +------Os03g18740
|      |      |
|      |      |                                  +------AT2G47120
|      |      +----------------------------474.6-|
|      |      |                                  +------AT3G29250
|      |      |
|      |      +-----------------------------------------AT3G51680
|      |      |
|      |      +-----------------------------------------AT4G03140
|      |
|      |                    +---------------------------Pt25323 18
|      |                    |
|      +--------------310.1-|      +--------------------pg12 113 3
|                           |      |
|                           |      |             +------Pt23982550
|                           +293.0-|             |
|                                  |      +459.8-|------pg8 9 36
|                                  |      |      |
|                                  +280.7-|      +------Pt46854 20
|                                         |
|                                         |      +------Pt24624 17
|                                         +492.1-|
|                                                +------pg12 112 4
|
+-------------------------------------------------------Pt69457 20
```

Spermine synthase - NJ

```
                                          +------Os02g15550
                                   +500.0-|
                            +499.0-|      +------Os06g33710
                            |      |
                     +499.0-|      +-------------AT5G53120
                     |      |
                     |      |             +------pg6 98 58
              +496.0-|      +-------500.0-|
              |      |                    +------Os02g14190
       +500.0-|      |
       |      |      +---------------------------AT1G70310
       |      |
+------|      +----------------------------------Os07g22600
|      |
|      |                                  +------Pp1213518
|      +----------------------------498.0-|
|                                         +------Pt73158 32
|
+------------------------------------------------pg3 128 80
```

Spermine synthase - PARS

```
                                   +------Os02g14190
                     +-------500.0-|
                     |             +------pg6 98 58
              +455.4-|
              |      |      +-------------AT5G53120
              |      +414.8-|
       +433.6-|             |      +------Os06g33710
       |      |             +466.3-|
       |      |                    +------Os02g15550
       |      |
+------|      +---------------------------AT1G70310
|      |
|      |                           +------pg3 128 80
|      |                           |
|      +---------------------500.0-|------Pt73158 32
|                                  |
|                                  +------Pp1213518
|
+-----------------------------------------Os07g22600
```

Sucrose phosphatase - NJ

```
                                          +------AT3G54270
                     +--------------491.0-|
                     |                    +------pg1 68 31
                     |
              +500.0-|                    +------Os01g27880
              |      |             +500.0-|
              |      |      +481.0-|      +------Os02g05030
              |      |      |      |
              |      +300.0-|      +-------------Os05g05270
       +418.0-|             |
       |      |             |             +------pg3 68 23
       |      |             +-------500.0-|
+------|      |                           +------Pt7773 23
|      |      |
|      |      +----------------------------------AT2G35840
|      |
|      +-----------------------------------------AT3G52340
|
+------------------------------------------------AT1G51420
```

Sucrose phosphatase - PARS

```
                                        +------AT3G52340
                                 +440.8-|
             +-------500.0-|       +------AT1G51420
             |             |
             |             +-------------AT2G35840
      +498.0-|
      |      |                    +------pg1 68 31
      |      |      +-------499.0-|
      |      |      |             +------AT3G54270
      |      +485.7-|
+------|             |             +------Os01g27880
|      |             |      +500.0-|
|      |             +375.5-|      +------Os02g05030
|      |                    |
|      |                    +-------------Os05g05270
|      |
|      +----------------------------------Pt7773 23
|
+-----------------------------------------pg3 68 23
```

Superoxide dismutase (Cu-Zn) - NJ

```
                                                        +------Pt12177 25
                                                 +328.0-|
                                          +315.0-|      +------pg1 36 26
                                          |      |
                                   +500.0-|      +-------------AT1G12520
                                   |      |
                            +499.0-|      +--------------------Os04g48410
                            |      |
                            |      |                    +------AT2G28190
                     +283.0-|      +--------------500.0-|
                     |      |                           +------Os08g44770
                     |      |
              +500.0-|      +----------------------------------pg1 104 14
              |      |
              |      +-----------------------------------------AT5G18100
       +261.0-|      |
       |      |      +-----------------------------------------Pt1682554
+------|      |
|      |      +------------------------------------------------Os03g22810
|      |
|      +-------------------------------------------------------AT1G08830
|
+--------------------------------------------------------------Os07g46990
```

Superoxide dismutase (Cu-Zn) - PARS

```
                                                               +------Pt12177 25
                                                        +282.8-|
                                                 +282.8-|      +------pg1 36 26
                                                 |      |
                                          +500.0-|      +-------------AT1G12520
                                          |      |
                                   +397.0-|      +--------------------Os04g48410
                                   |      |
                            +469.4-|      +---------------------------AT5G18100
                            |      |
                            |      |                           +------Os08g44770
                     +463.1-|      +---------------------500.0-|
                     |      |                                  +------AT2G28190
              +499.0-|      |
              |      |      +-----------------------------------------Pt1682554
       +419.1-|      |
       |      |      +------------------------------------------------pg1 104 14
+------|      |
|      |      +-------------------------------------------------------AT1G08830
|      |
|      +--------------------------------------------------------------Os07g46990
|
+---------------------------------------------------------------------Os03g22810
```

Syntaxin - NJ

```
                                                        +------Os05g48020
                                   +--------------481.0-|
                                   |                    +------Os01g49150
                            +286.0-|
                            |      |      +--------------------Pt34309 26
                            |      |      |
                            |      +468.0-|             +------pg11 100 1
                            |             |      +302.0-|
                     +386.0-|             +320.0-|      +------Pt34282549
                     |      |                    |
                     |      |                    +-------------AT3G09740
                     |      |
              +500.0-|      +----------------------------------AT3G45280
              |      |      |
              |      |      +----------------------------------Os09g19550
              |      |
              |      +-----------------------------------------AT3G61450
              |
              |                                  +-------------Os02g11890
              |                                  |
              |                                  |      +------AT1G61290
              |                                  +500.0-|
              |                                  |      +------AT1G11250
       +500.0-|                           +266.0-|
       |      |                           |      +-------------Os03g57310
       |      |                           |      |
       |      |                    +450.0-|      +-------------Os06g39050
       |      |                    |      |      |
       |      |                    |      |      +-------------AT3G11820
       |      |                    |      |
       |      |                    |      +--------------------AT4G03330
       |      |             +275.0-|
       |      |             |      +---------------------------Os06g07200
+------|      |             |      |
|      |      |             |      |                    +------pg6 11 22
|      |      +-------491.0-|      |                    |
|      |                    |      +--------------304.0-|------AT1G08560
|      |                    |                           |
|      |                    |                           +------Pt1658 24
|      |                    |
|      |                    +----------------------------------Os03g52650
|      |
|      |                                  +--------------------Os01g73230
|      +----------------------------481.0-|
|                                         |      +-------------AT1G28490
|                                         +479.0-|
|                                                |      +------Pt52584 21
|                                                +500.0-|
|                                                       +------pg8 73 36
|
+--------------------------------------------------------------Os11g06740
```

Syntaxin - PARS

```
                                                                                                    +------pg6 11 22
                                                                                                    |
                                                                                                    +------Pt1658 24
                                                                      +-----------------------286.5-|
                                                                      |                             +------AT1G08560
                                                                      |                             |
                                                                      |                             +------Os06g07200
                                                                      |
                                                               +470.6-|         +-----------------------------AT3G11820
                                                            |         |         |
                                                            |         |         |         +-------------------AT4G03330
                                                            |         |            +458.9-|
                                                            |         |         |         |         +------AT1G61290
                                                            |         |         |            +500.0-|
                                                            |            +336.1-|                   +------AT1G11250
                                                     +500.0-|                   |
                                                  |         |                   +-----------------------------Os06g39050
                                                  |         |                   |
                                                  |         |                   +-----------------------------Os03g57310
                                                  |         |                   |
                                                  |         |                   +-----------------------------Os02g11890
                                                  |         |
                                                  |         +-------------------------------------------------Os03g52650
                                           +500.0-|
                                        |         |                                       +-------------------Pt34309 26
                                        |         |                                +498.5-|
                                        |         |                             |         |         +------pg11 100 1
                                        |         |                             |            +492.7-|
                                        |         |                             |                   +------Pt34282549
                                        |         |                             |
                                        |         |                             +-----------------------------AT3G61450
                                        |         |                             |
                                 +492.0-|         +-----------------------500.0-|-----------------------------AT3G09740
                              |         |                                       |
                              |         |                                       |                   +------Os01g49150
                              |         |                                       +-------------500.0-|
                              |         |                                       |                   +------Os05g48020
                              |         |                                       |
                       +486.8-|         |                                       +-----------------------------AT3G45280
                    |         |         |                                       |
                    |         |         |                                       +-----------------------------Os09g19550
                    |         |         |
             +500.0-|         |         +---------------------------------------------------------------------Os11g06740
          |         |         |
          |         |         +-------------------------------------------------------------------------------Os01g73230
+---------|         |
|         |         +-----------------------------------------------------------------------------------------AT1G28490
|         |
|         +---------------------------------------------------------------------------------------------------Pt52584 21
|
+-------------------------------------------------------------------------------------------------------------pg8 73 36
```

Thioredoxin - NJ

```
+------------------------------------------------------------------------------------------Os01g34620
|
|                                                                                   +------Os07g09310
|                                                                            +489.0-|
|                                                                     +285.0-|      +------Os03g58630
|                                                                     |      |
|                                                +--------------298.0-|      +-------------AT5G39950
|                                                |                    |
|                                                |                    +--------------------AT1G19730
|                                                |
|                                                |                                  +------Pt21182554
|                                                +----------------------------500.0-|
|                                                |                                  +------pg1 55 7
|                                                |
|                                                |                    +--------------------Os05g40190
|                                                |                    |
|                                                |             +368.0-|             +------Os01g07376
|                                                |             |      |      +264.0-|
|                                                |             |      +474.0-|      +------AT3G08710
|                                                |             |             |
|                                                |             |             +-------------Os05g07690
|                                                |      +372.0-|
|                                         +339.0-|      |      |                    +------pg11 66 15
|                                         |      |      |      +--------------366.0-|
|                                         |      |      |      |                    +------Pt50684 14
|                                         |      |      |      |
|                                         |      +277.0-|      +---------------------------AT2G40790
|                                         |      |      |
|                                         |      |      |                           +------AT3G17880
|                                         |      |      +---------------------499.0-|
|                                         |      |      |                           +------Os09g23650
|                                         |      |      |
|                                         |      |      +----------------------------------Os07g08840
|                                  +271.0-|      |
|                                  |      |      |                                  +------AT5G42980
|                                  |      |      +----------------------------459.0-|
|                                  |      |      |                                  +------AT1G45145
|                                  |      |      |
|                                  |      |      +-----------------------------------------AT3G51030
|                                  |      |
|                                  |      |                                         +------Os04g57930
|                                  |      |                                  +362.0-|
|                                  |      |                           +466.0-|      +------AT1G50320
|                                  |      |                           |      |
|                                  |      +---------------------326.0-|      +-------------Os12g08730
|                           +490.0-|                                  |
|                           |      |                                  +--------------------Os01g73234
|                           |      |
|                           |      |                                                +------AT3G53220
|                           |      +------------------------------------------469.0-|
|                           |      |                                                +------pg1 33 18
|                           |      |
|                    +298.0-|      |                                                +------pg3 22 15
|                    |      |      +------------------------------------------472.0-|
|                    |      |      |                                                +------Pt57897 14
|                    |      |      |
|                    |      |      |                                                +------AT3G06730
|             +346.0-|      |      +------------------------------------------451.0-|
|             |      |      |                                                       +------Os08g29110
|             |      |      |
|             |      |      +--------------------------------------------------------------AT2G38270
|             |      |
|      +434.0-|      |                                                              +------AT4G04950
|      |      |      +--------------------------------------------------------366.0-|
|      |      |                                                                     +------AT3G54900
|      |      |
+------|      |                                                                     +------Pt56411 72
       |      +---------------------------------------------------------------315.0-|
       |                                                                            +------pg5 35 34
       |
       +-----------------------------------------------------------------------------------AT3G15660
```

Thioredoxin - PARS

```
                                                                                                  +------Os05g07690
                                                                                           +304.4-|
                                                                                    +349.0-|      +------Os01g07376
                                                                                    |      |
                                                                             +453.0-|      +-------------AT3G08710
                                                                             |      |
                                                                      +370.3-|      +--------------------Os05g40190
                                                                      |      |
                                                               +279.3-|      +---------------------------AT2G40790
                                                               |      |
                                                               |      |                           +------pg11 66 15
                                                               |      +---------------------341.0-|
                                                               |                                  +------Pt50684 14
                                                        +323.0-|
                                                        |      |                                  +------AT3G17880
                                                        |      +----------------------------462.6-|
                                                        |      |                                  +------Os09g23650
                                                        |      |
                                                        |      +-----------------------------------------Os07g08840
                                                        |
                                                        |                                         +------Pt21182554
                                                        +-----------------------------------500.0-|
                                                        |                                         +------pg1 55 7
                                                        |
                                                 +350.7-|                                         +------AT5G42980
                                                 |      |                                  +260.3-|
                                                 |      +----------------------------256.4-|      +------AT1G45145
                                                 |      |                                  |
                                                 |      |                                  +-------------AT1G19730
                                                 |      |
                                                 |      |                                  +-------------AT5G39950
                                                 |      +----------------------------250.3-|
                                          +270.1-|      |                                  |      +------Os03g58630
                                          |      |      |                                  +493.0-|
                                          |      |      |                                         +------Os07g09310
                                          |      |      |
                                          |      |      +------------------------------------------------AT3G51030
                                          |      |
                                          |      +-------------------------------------------------------Os01g73234
                                          |      |
                                          |      |                                                +------Pt57897 14
                                   +287.3-|      +------------------------------------------480.3-|
                                   |      |                                                       +------pg3 22 15
                                   |      |
                                   |      |                                                +-------------Os12g08730
                                   |      +------------------------------------------433.9-|
                                   |      |                                                |      +------Os04g57930
                                   |      |                                                +368.0-|
                            +493.0-|      |                                                       +------AT1G50320
                            |      |      |
                            |      |      |                                                       +------Os08g29110
                            |      |      +-------------------------------------------------484.1-|
                            |      |                                                              +------AT3G06730
                     +481.9-|      |
                     |      |      |                                                              +------AT3G53220
                     |      |      +--------------------------------------------------------398.9-|
                     |      |                                                                     +------pg1 33 18
              +496.0-|      |
              |      |      +----------------------------------------------------------------------------AT2G38270
              |      |
              |      +-----------------------------------------------------------------------------------AT4G04950
       +484.9-|      |
       |      |      +-----------------------------------------------------------------------------------AT3G54900
       |      |
+------|      |                                                                                   +------pg5 35 34
|      |      +-----------------------------------------------------------------------------495.5-|
|      |                                                                                          +------Pt56411 72
|      |
|      +-------------------------------------------------------------------------------------------------AT3G15660
|
+--------------------------------------------------------------------------------------------------------Os01g34620
```

TIP tonoplast integral protein - NJ

```
+-----------------------------------------Os06g22960
|
|                                  +------AT2G36830
|                           +481.0-|
|                           |      +------AT3G26520
|                           |
|                           +-------------Os03g05290
|                    +497.0-|
|                    |      |      +------Os10g35050
|                    |      +264.0-|
|                    |      |      +------AT4G01470
|                    |      |
|             +500.0-|      +-------------Os01g74450
|             |      |
|             |      |             +------pg2 72 11
|             |      |      +500.0-|
|             |      |      |      +------Pt53472 15
|             |      |      |
|             |      +500.0-|      +------Os01g13120
|             |             +500.0-|
|             |             |      +------Os05g14240
|             |             |
|             |             +-------------AT2G25810
|      +437.0-|
|      |      |                    +------pg2 73 42
|      |      +--------------500.0-|
|      |      |                    +------Pt22115 11
|      |      |
|      |      |                    +------AT5G47450
|      |      |             +496.0-|
+------|      +-------444.0-|      +------AT4G17340
       |      |             |
       |      |             +-------------Os02g44080
       |      |
       |      |                    +------pg2 74 11
       |      +--------------500.0-|
       |                           +------Pt95282549
       |
       +----------------------------------AT3G16240
```

TIP tonoplast integral protein - PARS

```
                                                        +------Os05g14240
                                                 +469.6-|
                                          +261.1-|      +------Os01g13120
                                          |      |
                            +-------500.0-|      +-------------AT2G25810
                            |             |
                            |             |             +------Pt53472 15
                            |             +-------500.0-|
                            |                           +------pg2 72 11
                     +494.0-|
                     |      |      +---------------------------AT4G01470
                     |      |      |
                     |      |      |                    +------AT2G36830
                     |      |      |             +371.1-|
                     |      |      |      +316.5-|      +------AT3G26520
                     |      +407.6-|      |      |
              +378.6-|             +428.2-|      +-------------Os01g74450
              |      |             |      |
              |      |             |      +--------------------Os03g05290
              |      |             |
              |      |             +---------------------------Os10g35050
       +360.1-|      |
       |      |      |                                  +------pg2 73 42
       |      |      +----------------------------500.0-|
       |      |                                         +------Pt22115 11
       |      |
       |      |                                         +------pg2 74 11
+------|      +-----------------------------------500.0-|
|      |                                                +------Pt95282549
|      |
|      +-------------------------------------------------------AT3G16240
|      |
|      |                                                +------Os02g44080
|      |                                         +321.9-|
|      +-----------------------------------428.8-|      +------AT5G47450
|                                                |
|                                                +-------------AT4G17340
|
+--------------------------------------------------------------Os06g22960
```

Trans-cinnamate 4-monooxygenase - NJ

```
+------------------------------------------------Os05g25640
|
|                                         +------Os05g41440
|                                  +496.0-|
|                           +499.0-|      +------AT2G40890
|                           |      |
|                           |      +-------------AT1G74550
|                    +500.0-|
|                    |      |             +------AT4G22710
|                    |      |      +500.0-|
|                    |      +379.0-|      +------AT4G22690
|             +500.0-|             |
|             |      |             +-------------AT3G26280
|             |      |
|             |      |                    +------Os02g26810
|             |      +--------------500.0-|
|      +486.0-|                           +------Os02g26770
|      |      |
|      |      |                           +------Pt18882551
|      |      |                    +500.0-|
|      |      |                    |      +------pg5 118 65
+------|      +--------------499.0-|
       |                           |      +------pg11 84 62
       |                           +480.0-|
       |                                  +------Pt12448255
       |
       +-----------------------------------------AT2G30490
```

Trans-cinnamate 4-monooxygenase - PARS

```
                                          +------AT2G40890
                                   +499.0-|
                            +500.0-|      +------Os05g41440
                            |      |
                            |      +-------------AT1G74550
                     +500.0-|
                     |      +--------------------AT3G26280
                     |      |
                     |      |             +------AT4G22710
              +495.0-|      +-------500.0-|
              |      |                    +------AT4G22690
              |      |
              |      |                    +------Os02g26810
              |      +--------------500.0-|
       +490.0-|                           +------Os02g26770
       |      |
       |      |                           +------Pt12448255
       |      |                    +463.8-|
       |      |                    |      +------pg11 84 62
+------|      +--------------500.0-|
|      |                           |      +------Pt18882551
|      |                           +500.0-|
|      |                                  +------pg5 118 65
|      |
|      +-----------------------------------------AT2G30490
|
+------------------------------------------------Os05g25640
```

Transcription factor CBF/NF-Y/archaeal - NJ

```
                                                 +------pg 6 20 11
              +----------------------------500.0-|
              |                                  +------Pt57094 20
              |
              |                                  +------pg 4 80 11
              |                           +500.0-|
              |             +-------500.0-|      +------Pt95882551
              |             |             |
       +497.0-|             |             +-------------Pt40232 15
       |      |             |
       |      |             |                    +------Pt39382551
       |      |             |             +359.0-|
       |      |      +261.0-|      +500.0-|      +------pg 11 17 1
       |      |      |      |      |      |
       |      |      |      +463.0-|      +-------------Pt40382549
+------|      |      |      |      |
|      |      +358.0-|      |      +--------------------Os03g14669
|      |             |      |
|      |             |      +---------------------------AT1G08970
|      |             |
|      |             +----------------------------------AT1G56170
|      |
|      +------------------------------------------------Os06g45640
|
+-------------------------------------------------------Os02g07450
```

Transcription factor CBF/NF-Y/archaeal - PARS

```
                                                 +------Pt39382551
                                                 |
                                          +500.0-|------pg 11 17 1
                                          |      |
                                   +469.6-|      +------Pt40382549
                                   |      |
                                   |      +-------------Os03g14669
                            +415.6-|
                            |      |             +------Pt95882551
                            |      |             |
                     +447.9-|      +-------488.7-|------pg 4 80 11
                     |      |                    |
                     |      |                    +------Pt40232 15
              +448.3-|      |
              |      |      +---------------------------AT1G08970
              |      |
       +499.7-|      |                           +------Os06g45640
       |      |      +---------------------489.3-|
       |      |                                  +------Os02g07450
+------|      |
|      |      +-----------------------------------------AT1G56170
|      |
|      +------------------------------------------------Pt57094 20
|
+-------------------------------------------------------pg 6 20 11
```

Transducin - NJ

```
                                          +------Os03g27970
                                   +491.0-|
              +--------------500.0-|      +------AT1G10580
              |                    |
              |                    +-------------pg2 158 27
              |
              |                           +------Pt3182547
              |                    +466.0-|
              |             +496.0-|      +------pg4 46 55
              |             |      |
              |      +489.0-|      +-------------pg8 138 19
              |      |      |
              |      |      |             +------Os07g38430
              |      |      +-------485.0-|
              +276.0-|                    +------AT4G02730
              |      |
              |      +---------------------------AT3G49660
              |      |
              |      +---------------------------Os03g51550
              |
              |                           +------Os03g23909
              |                    +497.0-|
              +--------------500.0-|      +------Os03g23929
              |                    |
              |                    +-------------AT2G41500
              |
              |                           +------Os06g44370
              +---------------------500.0-|
              |                           +------AT2G43770
              |
       +270.0-|                           +------Pt10098255
       |      |                    +500.0-|
       |      +--------------500.0-|      +------pg11 83 20
       |      |                    |
       |      |                    +-------------Os11g43890
       |      |
       |      |                           +------pg7 114 25
       |      +---------------------500.0-|
       |      |                           +------AT2G16405
       |      |
       |      |                           +------Os02g21490
       |      +---------------------500.0-|
       |      |                           +------AT5G54520
       |      |
       |      |             +--------------------Os03g48090
+------|      |             |
|      |      +-------495.0-|             +------pg3 59 59
|      |      |             |      +471.0-|
|      |      |             +496.0-|      +------Pt768 42
|      |      |                    |
|      |      |                    +-------------AT1G71840
|      |      |
|      |      |                           +------AT5G23430
|      |      +---------------------500.0-|
|      |      |                           +------Os10g35200
|      |      |
|      |      +----------------------------------AT5G64730
|      |      |
|      |      +----------------------------------AT5G08560
|      |
|      +-----------------------------------------AT5G67320
|
+------------------------------------------------AT5G13480
```

Transducin - PARS

```
                            +-------------AT5G64730
                            |
       +--------------259.2-|      +------AT1G10580
       |                    |      |
       |                    +500.0-|------pg2 158 27
       |                           |
       |                           +------Os03g27970
       |
       |                           +------Os06g44370
       +---------------------500.0-|
       |                           +------AT2G43770
       |
       |                           +------Os03g23909
       |                    +500.0-|
       +--------------500.0-|      +------Os03g23929
       |                    |
       |                    +-------------AT2G41500
       |
       |                           +------AT2G16405
       +---------------------500.0-|
       |                           +------pg7 114 25
       |
       |                    +-------------Os11g43890
       +--------------500.0-|
       |                    |      +------pg11 83 20
       |                    +500.0-|
       |                           +------Pt10098255
       |
       |                           +------Os02g21490
       +---------------------500.0-|
       |                           +------AT5G54520
+------|
|      |                           +------Pt768 42
|      |                    +500.0-|
|      |             +500.0-|      +------pg3 59 59
|      |             |      |
|      +-------382.5-|      +-------------AT1G71840
|      |             |
|      |             +--------------------Os03g48090
|      |
|      |                           +------Os10g35200
|      +---------------------500.0-|
|      |                           +------AT5G23430
|      |
|      |      +---------------------------AT3G49660
|      |      |
|      |      |             +-------------pg8 138 19
|      |      |      +499.0-|
|      |      |      |      |      +------Pt3182547
|      |      |      |      +433.4-|
|      +319.5-|-472.0-|             +------pg4 46 55
|      |      |      |
|      |      |      |             +------Os07g38430
|      |      |      +-------474.0-|
|      |      |                    +------AT4G02730
|      |      |
|      |      +---------------------------Os03g51550
|      |
|      +----------------------------------AT5G67320
|      |
|      +----------------------------------AT5G08560
|
+-----------------------------------------AT5G13480
```

Transducin - NJ

```
                     +-----------------------------------------AT3G49660
                     |
                     |                                  +------AT2G16405
                     |                    +-------500.0-|
                     |                    |             +------pg7 114 25
                     |                    |
                     |             +403.0-|      +-------------AT4G03020
              +353.0-|             |      |      |
              |      |             |      |      |      +------Os05g33710
              |      |             |      |      +349.0-|
              |      |             |      +484.0-|      +------pg12 24 33
              |      |      +448.0-|             |
              |      |      |      |             +-------------Os05g05210
              |      |      |      |             |
              |      +454.0-|      |             +-------------Os01g28680
       +359.0-|             |      |
       |      |             |      +---------------------------AT2G41500
       |      |             |
       |      |             +----------------------------------Os03g51550
       |      |
       |      |                           +--------------------Pt16984 18
       |      |                           |
       |      |                           |      +-------------pg8 138 19
       |      |                           +494.0-|
       |      +---------------------484.0-|      |      +------Pt3182547
       |                                  |      +377.0-|
+------|                                  |             +------pg4 46 55
|      |                                  |
|      |                                  |             +------Os07g38430
|      |                                  +-------446.0-|
|      |                                                +------AT4G02730
|      |
|      +-------------------------------------------------------AT3G21540
|      |
|      |                                                +------AT2G43770
|      +------------------------------------------499.0-|
|      |                                                +------Os06g44370
|      |
|      +-------------------------------------------------------AT5G64730
|
+--------------------------------------------------------------AT5G08560
```

Transducin - PARS

```
       +----------------------------------AT5G08560
       |
       |                           +------Os06g44370
       +---------------------500.0-|
       |                           +------AT2G43770
       |
       |                    +-------------Os05g33710
       |                    |
       |             +267.7-|-------------AT4G03020
       |             |      |
       |             |      |      +------Os05g05210
       |      +500.0-|      +340.9-|
       |      |      |             +------Os01g28680
       |      |      |
       +332.4-|      +--------------------pg12 24 33
       |      |
       |      |                    +------AT2G16405
       |      +--------------500.0-|
       |                           +------pg7 114 25
+------|
|      +----------------------------------AT2G41500
|      |
|      |                           +------AT4G02730
|      |             +-------445.9-|
|      |             |             +------Os07g38430
|      |             |
|      |             |             +------pg4 46 55
|      |      +482.5-|      +448.5-|
|      |      |      +366.5-|      +------Pt3182547
|      |      |      |      |
|      |      |      |      +-------------pg8 138 19
|      +311.8-|      |
|      |      |      +--------------------Pt16984 18
|      |      |
|      |      +---------------------------Os03g51550
|      |      |
|      |      +---------------------------AT3G49660
|      |
|      +----------------------------------AT5G64730
|
+-----------------------------------------AT3G21540
```

Transferase - NJ

```
                                          +-------------AT5G41040
                                   +459.0-|
                                   |      |      +------AT5G63560
                            +483.0-|      +500.0-|
                            |      |             +------AT3G48720
                            |      |
                            |      +--------------------Os11g31090
                     +500.0-|
                     |      +---------------------------AT1G65450
                     |      |
                     |      |                    +------Pt20373 27
                     |      +--------------500.0-|
                     |                           +------pg11 20 52
                     |
              +500.0-|----------------------------------AT5G57840
              |      |
              |      |                           +------pg10 101 6
              |      +---------------------500.0-|
              |      |                           +------Pt6396 36
       +500.0-|      |
       |      |      |                           +------pg6 118 17
       |      |      +---------------------400.0-|
+------|      |                                  +------AT2G19070
|      |      |
|      |      +-----------------------------------------AT5G48930
|      |
|      +------------------------------------------------Os02g39850
|
+-------------------------------------------------------Os04g42250
```

Transferase - PARS

```
                                                               +------Pt20373 27
                                                 +-------500.0-|
                                                 |             +------pg11 20 52
                                                 |
                                                 +--------------------AT1G65450
                                          +500.0-|
                                          |      |      +-------------AT5G41040
                                          |      |      |
                                          |      |      |      +------AT3G48720
                                   +250.7-|      +263.9-|-485.7-|
                                   |      |             |      +------AT5G63560
                                   |      |             |
                            +430.2-|      |             +-------------Os11g31090
                            |      |      |
                            |      |      +---------------------------AT2G19070
                     +264.5-|      |
                     |      |      +----------------------------------pg6 118 17
                     |      |
              +408.7-|      |                                  +------pg10 101 6
              |      |      +----------------------------500.0-|
              |      |                                         +------Pt6396 36
       +500.0-|      |
       |      |      +------------------------------------------------AT5G57840
+------|      |
|      |      +-------------------------------------------------------AT5G48930
|      |
|      +--------------------------------------------------------------Os04g42250
|
+---------------------------------------------------------------------Os02g39850
```

TUBBY - NJ

```
                                    +------Os03g22800
                            +500.0-|
              +--------------481.0-|      +------Os07g47110
              |                    |
              |                    +-------------AT2G47900
              |
              |                           +------AT1G76900
              |      +--------------317.0-|
              |      |                    +------AT1G43640
              |      |
              |      |      +------------------Os05g36190
              |      |      |
              +396.0-|-425.0-|                     +------Os12g06630
              |      |      |      +492.0-|
              |      |      +479.0-|      +------Os11g06420
              |      |             |
              |      |             +-------------Os01g64700
              |      |
              |      +--------------------------AT1G25280
              |
              +---------------------------------AT2G18280
              |
       +302.0-|                           +------pg 6 15 58
       |      |                    +490.0-|
       |      |             +465.0-|      +------pg 5 37 58
       |      |             |      |
       |      +-------266.0-|      +-------------Pt74182554
       |      |             |
       |      |             +------------------Pt45290 19
       |      |
       |      +---------------------------------AT1G53320
       |      |
       |      +---------------------------------Pt1408 39
+------|      |
|      |      |                           +------pg8 25put
|      |      |                    +413.0-|
|      |      +--------------380.0-|      +------Pt3734 33
|      |      |                    |
|      |      |                    +-------------pg6 65put
|      |      |
|      |      +---------------------------------AT1G47270
|      |      |
|      |      +---------------------------------Os02g47640
|      |
|      |                                  +------AT5G18680
|      +---------------------------500.0-|
|                                         +------AT3G06380
|
+--------------------------------------------Os08g01290
```

TUBBY - PARS

```
                                            +------AT5G18680
                            +-------500.0-|
                            |             +------AT3G06380
                     +376.3-|
                     |      |      +-------------AT2G47900
                     |      +323.4-|
                     |             |      +------Os03g22800
                     |             +492.9-|
                     |                    +------Os07g47110
                     |
                     +---------------------------AT2G18280
                     |
                     |                    +------AT1G53320
                     +--------------285.4-|
                     |                    +------Pt1408 39
                     |
                     |                    +------pg 5 37 58
              +287.3-|             +373.7-|
              |      +-------320.7-|      +------pg 6 15 58
              |      |             |
              |      |             +-------------Pt74182554
              |      |
              |      +---------------------------Os02g47640
              |      |
              |      +---------------------------Pt3734 33
              |      |
              |      +---------------------------pg8 25put
              |      |
              |      +---------------------------AT1G47270
              |      |
       +465.2-|      +---------------------------Pt45290 19
       |      |      |
       |      |      +---------------------------pg6 65put
       |      |
       |      +----------------------------------AT1G43640
       |      |
       |      +----------------------------------Os01g64700
       |      |
+------|      +----------------------------------AT1G25280
|      |      |
|      |      +----------------------------------Os08g01290
|      |      |
|      |      +----------------------------------Os05g36190
|      |      |
|      |      +----------------------------------AT1G76900
|      |
|      +-----------------------------------------Os11g06420
|
+------------------------------------------------Os12g06630
```

Tubulin alpha - NJ

```
                                   +------pg 2 35 67
                                   |
                                   +------Pt10028255
                                   |
                                   +------Pp3713522
                                   |
                     +-------440.0-|------Pt11008255
                     |             |
                     |             +------Pp12052 28
                     |             |
                     |             +------pg 8 162 5
                     |             |
                     |             +------Pt23182550
                     |
                     |             +------AT4G14960
                     |      +304.0-|
              +342.0-|-483.0-|      +------AT1G04820
              |      |      |
              |      |      +-------------AT1G50010
              |      |
              |      |             +------Os11g14220
              |      |      +452.0-|
              |      +280.0-|      +------Os03g51600
       +461.0-|      |      |
       |      |      |      +-------------Os07g38730
       |      |      |
       |      |      |             +------pg 7 150 8
       |      |      +-------467.0-|
+------|      |                    +------Pt58310 30
|      |      |
|      |      |                    +------Os03g11970
|      |      +--------------492.0-|
|      |                           +------pg2 39put
|      |
|      +----------------------------------AT5G19780
|
+-----------------------------------------AT5G19770
```

Tubulin alpha - PARS

```
+---------------------------pg 7 150 8
|
|      +--------------------pg 2 35 67
|      |
|      |             +------AT4G14960
|      |      +321.1-|
|      +422.2-|      +------AT1G50010
|      |      |
|      |      +-------------AT1G04820
|      |
|      +--------------------Pt11008255
|      |
|      +--------------------Pp3713522
|      |
|      +--------------------Os07g38730
|      |
|      +--------------------Pp12052 28
|      |
|      |             +------Os11g14220
+------|-------382.0-|
       |             +------Os03g51600
       |
       +--------------------Os03g11970
       |
       +--------------------pg 8 162 5
       |
       |             +------AT5G19780
       +-------388.9-|
       |             +------AT5G19770
       |
       +--------------------Pt10028255
       |
       +--------------------pg2 39put
       |
       +--------------------Pt23182550
       |
       +--------------------Pt58310 30
```

Ubiquitin-conjugating enzyme - NJ

```
                                                                                    +------pg6 100 40
                                                                             +496.0-|
                                                                             |      +------Pt26698 18
                                   +-----------------------------------500.0-|
                                   |                                         |      +------AT5G25760
                                   |                                         +491.0-|
                                   |                                                +------Os02g42314
                                   |
                                   |                                                +------Os03g47770
                                   |                           +--------------301.0-|
                                   |                           |                    +------Os12g44000
                                   |                           |
                                   |                           |                    +------pg7 158 14
                                   |                           |             +368.0-|
                                   |                           |             |      +------Pt42807 14
                            +500.0-|                    +276.0-|      +498.0-|
                            |      |                    |      |      |      |      +------Pt85082553
                            |      |                    |      +272.0-|      +491.0-|
                            |      |                    |      |      |             +------pg5 153 12
                            |      |             +416.0-|      |      |
                            |      |             |      |      |      +--------------------AT1G75440
                            |      |             |      |      |
                            |      |      +500.0-|      |      +---------------------------AT4G36410
                            |      |      |      |      |
                            |      |      |      |      +----------------------------------AT5G42990
                     +300.0-|      |      |      |
                     |      |      |      |      +-----------------------------------------AT1G45050
                     |      |      +308.0-|
                     |      |             |                                         +------Pt72782549
                     |      |             |                                  +500.0-|
                     |      |             |                           +342.0-|      +------pg1 135 18
              +335.0-|      |             |                           |      |
              |      |      |             +---------------------499.0-|      +-------------Os01g70140
              |      |      |                                         |
              |      |      |                                         +--------------------AT5G50870
              |      |      |
              |      |      +--------------------------------------------------------------Os02g02830
              |      |
              |      +---------------------------------------------------------------------AT2G16740
              |
              |                                                       +--------------------Os04g57220
              |                                                       |
       +474.0-|-------------------------------------------------294.0-|             +------Os01g60360
       |      |                                                       |      +500.0-|
       |      |                                                       +311.0-|      +------Os01g60410
       |      |                                                              |
       |      |                                                              +-------------Os06g30970
       |      |
       |      |                                                                     +------pg11 67 9
       |      +---------------------------------------------------------------496.0-|
       |      |                                                                     +------Pt4582553
+------|      |
|      |      |                                                                     +------AT5G41700
|      |      |                                                                     |
|      |      +---------------------------------------------------------------274.0-|------AT3G08690
|      |                                                                            |
|      |                                                                            +------AT5G53300
|      |
|      +-----------------------------------------------------------------------------------AT1G64230
|      |
|      +-----------------------------------------------------------------------------------Os02g16040
|
+------------------------------------------------------------------------------------------AT4G27960
```

Ubiquitin-conjugating enzyme - PARS

```
                                                                      +------Os01g60410
       +--------------------------------------------------------395.5-|
       |                                                              +------Os01g60360
       |
       |                                                              +------Os02g42314
       |                                                       +473.3-|
       |                                                       |      +------AT5G25760
       |                    +----------------------------499.9-|
       |                    |                                  |      +------pg6 100 40
       |                    |                                  +499.9-|
       |                    |                                         +------Pt26698 18
       |                    |
       |                    |                                         +------Pt42807 14
       |                    |                                  +492.9-|
       |                    |                                  |      +------pg7 158 14
       |                    |                           +331.8-|
       |                    |                           |      |      +------pg5 153 12
       |                    |                           |      +481.2-|
       |             +497.9-|                           |             +------Pt85082553
       |             |      |                    +267.3-|
       |             |      |                    |      |             +------AT1G45050
       |             |      |                    |      |      +388.6-|
       |             |      |                    |      |      |      +------AT5G42990
       |             |      |             +449.6-|      +364.8-|
       |             |      |             |      |             |      +------Os12g44000
       |             |      |             |      |             +416.6-|
       |             |      |      +499.9-|      |                    +------Os03g47770
       |             |      |      |      |      |
       |             |      |      |      |      +---------------------------AT1G75440
       |      +269.6-|      |      |      |
+------|      |      |      +279.9-|      +----------------------------------AT4G36410
|      |      |      |             |
|      |      |      |             |                           +-------------Os01g70140
|      |      |      |             |                    +440.4-|
|      |      |      |             |                    |      |      +------pg1 135 18
|      +296.0-|      |             +--------------443.9-|      +499.9-|
|      |      |      |                                  |             +------Pt72782549
|      |      |      |                                  |
|      |      |      |                                  +--------------------AT5G50870
|      |      |      |
|      |      |      +-------------------------------------------------------AT2G16740
|      |      |
|      |      +--------------------------------------------------------------Os02g02830
|      |
|      +---------------------------------------------------------------------Os02g16040
|      |
|      |                                                              +------Pt4582553
|      +--------------------------------------------------------294.1-|
|      |                                                              +------pg11 67 9
|      |
|      +---------------------------------------------------------------------AT3G08690
|      |
|      +---------------------------------------------------------------------AT1G64230
|      |
|      +---------------------------------------------------------------------AT4G27960
|      |
|      +---------------------------------------------------------------------Os06g30970
|      |
|      +---------------------------------------------------------------------AT5G41700
|      |
|      +---------------------------------------------------------------------AT5G53300
|
+----------------------------------------------------------------------------Os04g57220
```

ubiquitin interaction motif-containing protein / LIM domain-containing protein NJ

```
                                    +------pg 4 128 5
                    +-------500.0-|
                    |             +------Pt39952 14
                    |
                    |             +------Pp4363 13
                    |             |
                    |      +500.0-|------Pt65247 38
              +256.0-|      |      |
              |      +478.0-|      +------Pt65248 14
              |      |      |
              |      |      +-------------pg 4 15 30
              |      |
              |      |      +-------------Os12g40490
       +267.0-|      +335.0-|
       |      |             |      +------Os06g08400
       |      |             +465.0-|
       |      |                    +------AT4G36860
+------|      |
|      |      |                    +------AT1G19270
|      |      +--------------257.0-|
|      |                           +------AT5G66620
|      |
|      +----------------------------------AT2G39830
|
+-----------------------------------------Os03g16090
```

ubiquitin interaction motif-containing protein / LIM domain-containing protein PARS

```
                                   +-------------pg 4 15 30
                                   |
                            +462.1-|      +------Pp4363 13
                            |      |      |
                            |      +497.5-|------Pt65248 14
                     +313.9-|             |
                     |      |             +------Pt65247 38
                     |      |
                     |      |             +------pg 4 128 5
              +400.7-|      +-------500.0-|
              |      |                    +------Pt39952 14
              |      |
              |      |             +-------------Os12g40490
              |      +-------460.2-|
       +442.0-|                    |      +------Os06g08400
       |      |                    +492.9-|
       |      |                           +------AT4G36860
       |      |
+------|      |                           +------AT2G39830
|      |      +---------------------318.7-|
|      |                                  +------Os03g16090
|      |
|      +-----------------------------------------AT1G19270
|
+------------------------------------------------AT5G66620
```

Ubiquitin-protein ligase - NJ

```
                                          +------Os03g47949
                                   +500.0-|
                                   |      +------AT3G53090
                            +500.0-|
                            |      |      +------AT3G17205
                     +500.0-|      +500.0-|
                     |      |             +------Os05g06690
              +363.0-|      |
              |      |      +--------------------Pt55252 36
              |      |
              |      +---------------------------Os12g24080
              |
       +492.0-|                           +------pg12 16 19
       |      +---------------------497.0-|
       |      |                           +------Pt11956 33
       |      |
       |      |                           +------pg9 38 45
+------|      |                    +500.0-|
|      |      +--------------441.0-|      +------Pt40134 20
|      |                           |
|      |                           +-------------Os09g07900
|      |
|      +-----------------------------------------AT1G55860
|
+------------------------------------------------AT1G70320
```

Ubiquitin-protein ligase - PARS

```
                                 +------Os03g47949
                          +500.0-|
                          |      +------AT3G53090
                   +500.0-|
                   |      |      +------Os05g06690
            +500.0-|      +500.0-|
            |      |             +------AT3G17205
            |      |
     +269.7-|      +-------------------Pt55252 36
     |      |
     |      |                    +------Pt11956 33
     |      +-------------489.3-|
     |                           +------pg12 16 19
+------|
|      |                  +-------------Os09g07900
|      |           +280.3-|
|      |           |      |      +------pg9 38 45
|      |           |      +500.0-|
|      +-------274.7-|             +------Pt40134 20
|                  |
|                  |             +------AT1G55860
|                  +-------495.6-|
|                                +------AT1G70320
|
+--------------------------------------Os12g24080
```

UDP-glucose 6-dehydrogenase - NJ

```
                                          +------pg 1 127 9
                                   +500.0-|
                     +-------446.0-|      +------Pt11368254
                     |             |
                     |             +-------------AT1G26570
                     |
                     |                    +------Os03g55070
              +499.0-|             +480.0-|
              |      |             |      +------Os12g25690
              |      |      +500.0-|
              |      |      |      +-------------Os12g25700
              |      |      |      |
       +395.0-|      +483.0-|      +-------------pg 2 99 17
       |      |             |
       |      |             |             +------Pt84282554
       |      |             +-------500.0-|
+------|      |                           +------Pp5213523
|      |      |
|      |      +----------------------------------AT5G39320
|      |
|      +-----------------------------------------AT3G29360
|
+------------------------------------------------AT5G15490
```

UDP-glucose 6-dehydrogenase - PARS

```
          +---------------------------------AT5G15490
          |
          |                          +------pg 1 127 9
          |                   +500.0-|
          |      +-------497.5-|      +------Pt11368254
          |      |             |
          |      |             +-------------AT1G26570
          |      |
          +319.6-|             +-------------pg 2 99 17
          |      |             |
   +------|      |      +491.9-|-------------Os12g25700
   |      |      |      |      |
   |      |      |      |      |      +------Os03g55070
   |      |      +500.0-|      +494.3-|
   |      |             |             +------Os12g25690
   |      |             |
   |      |             |             +------Pp5213523
   |      |             +-------500.0-|
   |      |                           +------Pt84282554
   |      |
   |      +---------------------------------AT3G29360
   |
   +----------------------------------------AT5G39320
```

ubx domain-containing protein - NJ

```
                                          +-------------Os10g37630
                                   +283.0-|
                                   |      |      +------Pt19160 12
                            +288.0-|      +486.0-|
                            |      |             +------pg 10 49 3
                     +500.0-|      |
                     |      |      +--------------------AT4G10790
              +492.0-|      |
              |      |      +---------------------------Os09g30411
              |      |
       +500.0-|      +----------------------------------AT4G23040
       |      |
       |      |                                  +------Os08g43300
       |      |                           +473.0-|
+------|      +---------------------351.0-|      +------Os09g35710
|      |                                  |
|      |                                  +-------------AT4G11740
|      |
|      +------------------------------------------------pg 1 153 1
|
+-------------------------------------------------------Pt29782549
```

ubx domain-containing protein - PARS

```
                            +------pg 10 49 3
                     +500.0-|
              +483.9-|      +------Pt19160 12
              |      |
       +498.0-|      +-------------AT4G10790
       |      |
       |      +--------------------Os10g37630
       |
       |                    +------pg 1 153 1
+------|             +500.0-|
|      |             |      +------Pt29782549
|      |             |
|      |      +380.1-|-------------AT4G11740
|      |      |      |
|      |      |      |      +------Os08g43300
|      +500.0-|      +436.6-|
|             |             +------Os09g35710
|             |
|             +--------------------AT4G23040
|
+----------------------------------Os09g30411
```

VHS domain-containing protein - NJ

```
                                          +------AT1G76970
                                   +500.0-|
                            +500.0-|      +------AT1G21380
                            |      |
                     +354.0-|      +-------------Os11g09329
                     |      |
                     |      |             +------pg2 5 45
              +334.0-|      +-------500.0-|
              |      |                    +------Pt57729 41
       +270.0-|      |
       |      |      +---------------------------Os01g12880
       |      |
       |      +----------------------------------AT2G38410
       |
       |                           +-------------Os10g42724
       |                    +474.0-|
       |                    |      |      +------Os02g46962
       |                    |      +465.0-|
       |             +326.0-|             +------AT5G16880
       |             |      |
+------|             |      |             +------Pt9182552
|      |             |      +-------500.0-|
|      |      +500.0-|                    +------pg6 90 48
|      |      |      |
|      |      |      +---------------------------AT1G06210
|      +282.0-|      |
|      |      |      +---------------------------Pt23114 30
|      |      |
|      |      +----------------------------------AT4G32760
|      |
|      |                                  +------Os02g17350
|      +----------------------------318.0-|
|                                         +------Os06g22650
|
+------------------------------------------------AT3G08790
```

VHS domain-containing protein - PARS

```
                                                        +------Os02g17350
                            +---------------------364.0-|
                            |                           +------Os06g22650
                            |
                            |                           +------Pt57729 41
                            |             +-------500.0-|
                            |             |             +------pg2 5 45
                            |      +262.4-|
                     +332.8-|      |      |             +------AT1G21380
                     |      |      |      |      +500.0-|
                     |      +280.9-|      +500.0-|      +------AT1G76970
                     |      |      |             |
                     |      |      |             +-------------Os11g09329
              +301.0-|      |      |
              |      |      |      +---------------------------AT2G38410
              |      |      |
       +500.0-|      |      +----------------------------------Os01g12880
       |      |      |
       |      |      +-----------------------------------------AT3G08790
       |      |
+------|      +------------------------------------------------AT4G32760
|      |
|      |                           +---------------------------Pt23114 30
|      |                           |
|      +---------------------300.0-|                    +------pg6 90 48
|                                  |      +-------500.0-|
|                                  |      |             +------Pt9182552
|                                  +357.1-|
|                                         |      +-------------AT5G16880
|                                         +354.8-|
|                                                |      +------Os10g42724
|                                                +266.8-|
|                                                       +------Os02g46962
|
+--------------------------------------------------------------AT1G06210
```

XET - NJ

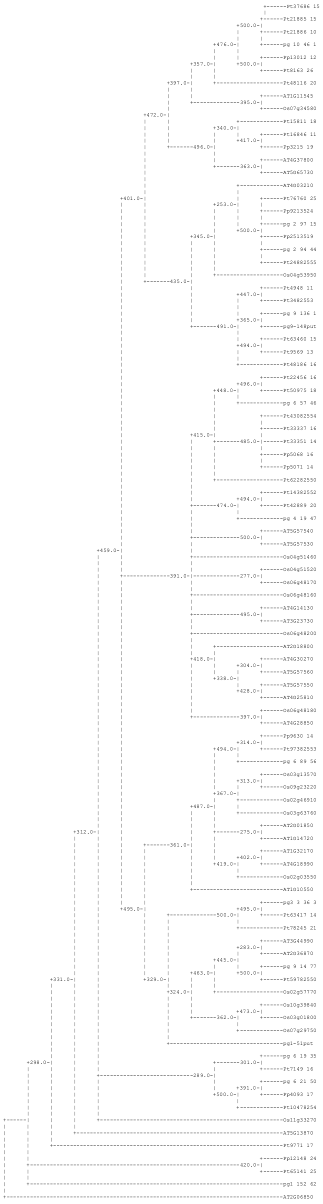
Pt37686 15
Pt21885 15
Pt21886 10
pg 10 46 1
Pp13012 12
Pt8163 26
Pt48116 20
AT1G11545
Os07g34580
Pt15811 18
Pt16846 11
Pp3215 19
AT4G37800
AT5G65730
AT4G03210
Pt76760 25
Pp9213524
pg 2 97 15
Pp2513519
pg 2 94 44
Pt24882555
Os04g53950
Pt4948 11
Pt3482553
pg 9 136 1
pg9-148put
Pt63460 15
Pt9569 13
Pt48186 16
Pt22456 16
Pt50975 18
pg 6 57 46
Pt43082554
Pt33337 16
Pt33351 14
Pp5068 16
Pp5071 14
Pt62282550
Pt14382552
Pt42889 20
pg 4 19 47
AT5G57540
AT5G57530
Os04g51460
Os04g51520
Os06g48170
Os06g48160
AT4G14130
AT3G23730
Os06g48200
AT2G18800
AT4G30270
AT5G57560
AT5G57550
AT4G25810
Os06g48180
AT4G28850
Pp9630 14
Pt97382553
pg 6 89 56
Os03g13570
Os09g23220
Os02g46910
Os03g63760
AT2G01850
AT1G14720
AT1G32170
AT4G18990
Os02g03550
AT1G10550
pg3 3 36 3
Pt63417 14
Pt78245 21
AT3G44990
AT2G36870
pg 9 14 77
Pt59782550
Os02g57770
Os10g39840
Os03g01800
Os07g29750
pg1-51put
pg 6 19 35
Pt7149 16
pg 6 21 50
Pp4093 17
Pt10478254
Os11g33270
AT5G13870
Pt9771 17
Pp12148 24
Pt65141 25
pg1 152 62
AT2G06850

XET - PARS

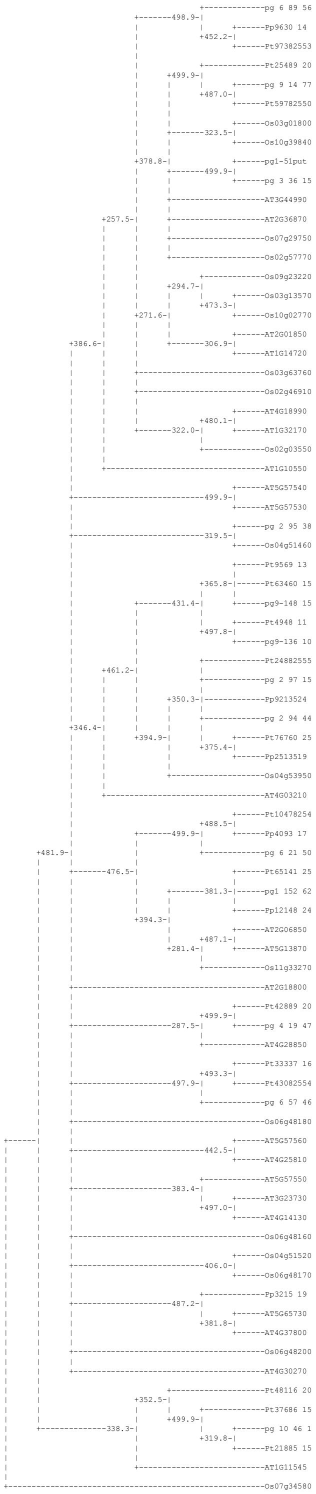

pg 6 89 56
498.9
Pp9630 14
452.2
Pt97382553
Pt25489 20
499.9
pg 9 14 77
487.0
Pt59782550
Os03g01800
323.5
Os10g39840
pg1-51put
378.8
499.9
pg 3 36 15
AT3G44990
257.5
AT2G36870
Os07g29750
Os02g57770
Os09g23220
294.7
Os03g13570
473.3
271.6
Os10g02770
AT2G01850
386.6
306.9
AT1G14720
Os03g63760
Os02g46910
AT4G18990
480.1
322.0
AT1G32170
Os02g03550
AT1G10550
AT5G57540
499.9
AT5G57530
pg 2 95 38
319.5
Os04g51460
Pt9569 13
365.8
Pt63460 15
431.4
pg9-148 15
Pt4948 11
497.8
pg9-136 10
Pt24882555
461.2
pg 2 97 15
350.3
Pp9213524
pg 2 94 44
346.4
394.9
Pt76760 25
375.4
Pp2513519
Os04g53950
AT4G03210
Pt10478254
488.5
499.9
Pp4093 17
481.9
pg 6 21 50
476.5
Pt65141 25
381.3
pg1 152 62
Pp12148 24
394.3
AT2G06850
487.1
281.4
AT5G13870
Os11g33270
AT2G18800
Pt42889 20
499.9
287.5
pg 4 19 47
AT4G28850
Pt33337 16
493.3
497.9
Pt43082554
pg 6 57 46
Os06g48180
AT5G57560
442.5
AT4G25810
AT5G57550
383.4
AT3G23730
497.0
AT4G14130
Os06g48160
Os04g51520
406.0
Os06g48170
Pp3215 19
487.2
AT5G65730
381.8
AT4G37800
Os06g48200
AT4G30270
Pt48116 20
352.5
Pt37686 15
499.9
338.3
pg 10 46 1
319.8
Pt21885 15
AT1G11545
Os07g34580

WRKY IIc - NJ

```
                     +------Os01g08710
       +-------335.0-|
       |             +------pg8 136 75
       |
       +--------------------AT1G69310
       |
       |             +------Os07g02060
       +-------359.0-|
       |             +------Os03g55080
       |
       +--------------------AT5G49520
+------|
|      |             +------AT1G29860
|      +-------364.0-|
|      |             +------AT4G18170
|      |
|      |             +------Os05g50610
|      |      +426.0-|
|      +351.0-|      +------Os01g43650
|      |      |
|      |      +-------------Os01g47560
|      |
|      +--------------------pg8 175 18
|
+---------------------------AT4G39410
```

WRKY IIc - PARS

```
                            +-------------Os01g47560
                            |
                            +-------------Os05g50610
                            |
                            +-------------Os01g43650
                     +267.9-|
                     |      |      +------pg8 175 18
                     |      +259.2-|
                     |      |      +------AT1G69310
              +280.6-|      |
              |      |      +-------------AT5G49520
              |      |
              |      +--------------------AT1G29860
              |      |
       +291.3-|      +--------------------AT4G18170
       |      |
       |      +---------------------------pg8 136 75
       |      |
+------|      +---------------------------Os01g08710
|      |      |
|      |      +---------------------------AT4G39410
|      |
|      +----------------------------------Os07g02060
|
+-----------------------------------------Os03g55080
```

WRKY I - NJ

```
                                                 +------AT4G04450
                                          +470.0-|
                                   +330.0-|      +------AT4G22070
                                   |      |
                                   |      +-------------AT1G62300
                                   |
                                   +--------------------Os01g14440
                            +269.0-|
                            |      |             +------Pt18456 26
                            |      +-------464.0-|
                     +500.0-|      |             +------pg1 107 34
                     |      |      |
                     |      |      +--------------------Os05g49210
                     |      |
              +284.0-|      +---------------------------Os01g18584
              |      |
              |      +----------------------------------Os03g53050
       +252.0-|      |
       |      |      +----------------------------------Os04g51560
       |      |
       |      +-----------------------------------------Os04g21950
+------|
|      |                                         +------Pp4313524
|      |                                         |
|      |                                  +496.0-|------pg1 50 60
|      |                                  |      |
|      +----------------------------305.0-|      +------Pt61318 36
|                                         |
|                                         +-------------AT4G24240
|
+-------------------------------------------------------AT4G31550
```

WRKY I - PARS

```
                                          +------Pp4313524
                                   +405.1-|
                            +500.0-|      +------Pt61318 36
                            |      |
                     +294.8-|      +-------------pg1 50 60
                     |      |
                     |      |      +-------------AT4G31550
                     |      +422.2-|
              +272.0-|             |      +------Os04g21950
              |      |             +342.3-|
              |      |                    +------Os04g51560
       +500.0-|      |
       |      |      +---------------------------AT4G24240
       |      |
       |      +----------------------------------Os03g53050
       |
       |                                  +------AT4G22070
+------|                           +488.0-|
|      |                    +346.0-|      +------AT4G04450
|      |                    |      |
|      |                    |      +-------------AT1G62300
|      |                    |
|      |                    |             +------pg1 107 34
|      +--------------432.5-|-------488.3-|
|                           |             +------Pt18456 26
|                           |
|                           +--------------------Os01g14440
|                           |
|                           +--------------------Os05g49210
|
+------------------------------------------------Os01g18584
```
