# Supplementary material for: A spruce gene map infers ancient plant genome reshuffling and subsequent slow evolution in the gymnosperm lineage leading to extant conifers
Source: BMC Biol. 2012 Oct 26;10:84. doi: 10.1186/1741-7007-10-84 (PMC3519789; doi:10.1186/1741-7007-10-84)
Supplement: Additional file 1 — Composite spruce gene linkage map. This composite linkage map consisted of 1,801 genes, including 1,743 genes from white spruce and 58 genes from black spruce, positioned onto the 12 linkage groups corresponding to the 12 spruce chromosomes. Genes highlighted in grey were positioned on both spruce genomes and genes written in red were positioned only onto the black spruce composite map [82]. All other genes were positioned on the white spruce composite map. Genetic distances are indicated in cM (Kosambi) at the left of each linkage group. For magnification, zoom into the figure. [file 1741-7007-10-84-S1.PDF]

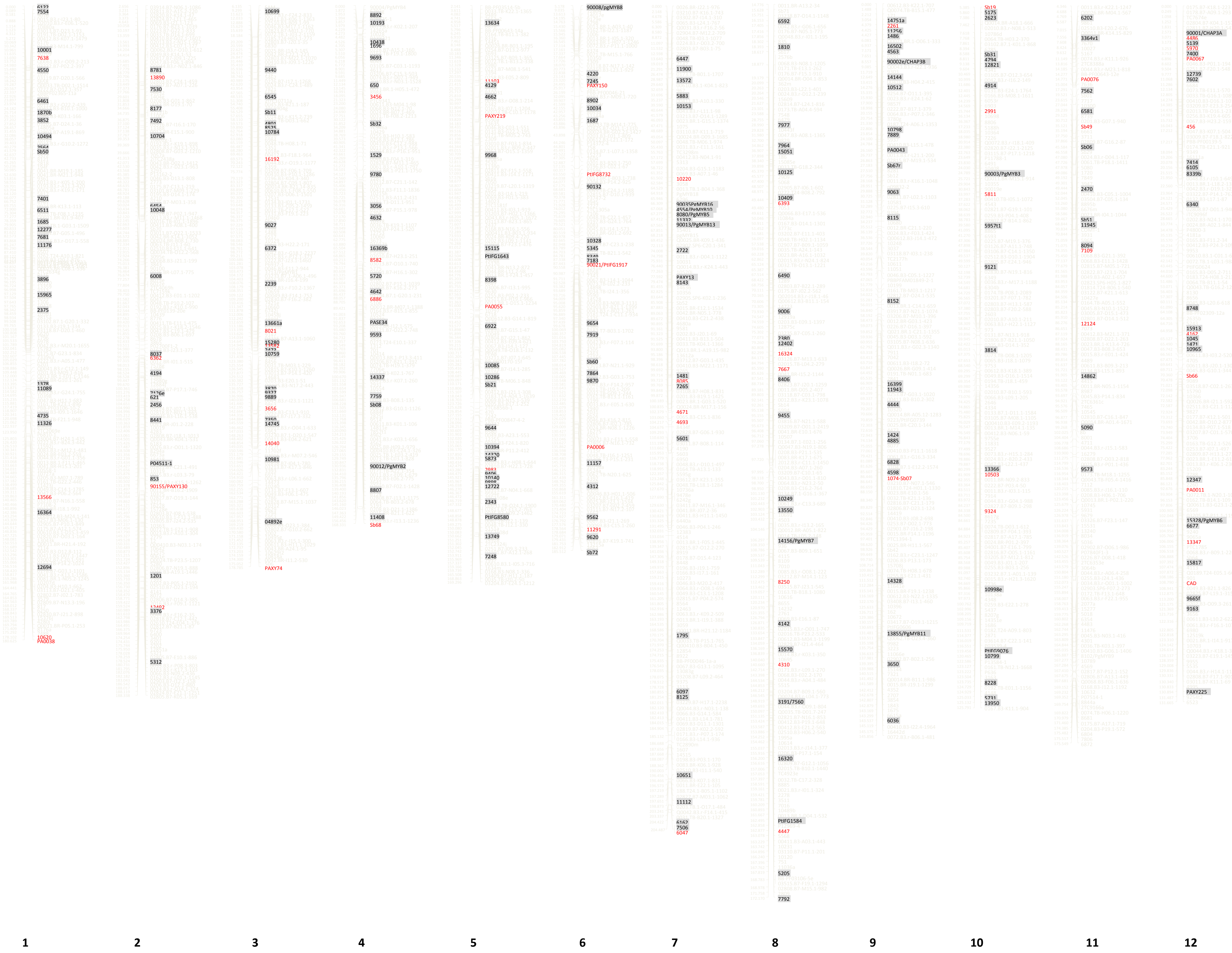
6122
7554
10001
7638
4550
6461
1870b
3852
10494
Sb50
7401
6511
1685
12277
7681
11176
3896
15965
2375
11089
4735
11326
13566
16364
12694
10620
PA0038
8781
13890
7530
8177
7492
10704
10048
6008
8037
6362
4194
621
2456
8441
P04511-1
853
90155/PAXY130
1201
3376
5312
10699
9440
6545
Sb11
10784
16192
9027
6372
2239
13661a
8021
15280
10759
9889
3656
14745
14040
10981
04892e
PAXY74
8892
10193
10438
1696
9693
650
3456
Sb32
1529
9780
3056
4632
16369b
8582
5720
4642
6886
PASE34
9593
14337
7759
Sb08
90012/PgMYB2
8807
11408
Sb68
13634
11103
4129
4662
PAXY219
9968
15115
PtIFG1643
8398
PA0055
6922
10085
10286
Sb21
9644
10394
5873
10140
12722
2343
PtIFG8580
13749
7248
90008/pgMYB8
4220
7245
PAXY150
8902
10034
1687
PtIFG8732
90132
10328
5345
7183
90021/PtIFG1917
9654
7919
Sb60
7864
9870
PA0006
11157
4312
9562
11291
9620
Sb72
6447
11900
13572
5883
10153
10220
90035PgMYB16
8080/PgMYB5
90013/PgMYB13
2722
PAXY13
8143
1481
8085
7265
4671
4693
5601
1795
6097
8125
10651
11112
6162
7506
6047
6592
1810
7977
7964
15051
10125
10409
6393
6490
9006
12402
16324
7667
8406
9455
10249
13550
14156/PgMYB7
8250
4142
15570
4310
3191/7560
16320
PtIFG1584
4447
5205
7792
14751a
2261
1486
16502
4563
90002e/CHAP3B
14144
10512
10798
7889
PA0043
Sb67r
9063
8115
8152
16399
11943
4444
1424
4885
6828
4598
1074-Sb07
14328
13855/PgMYB11
3650
6036
Sb19
5175
2623
Sb31
4794
12821
4914
2991
90003/PgMYB3
S811
5957t1
9121
3814
13366
10503
9324
10998e
PtIFG9076
10799
8228
13950
6202
3364v1
PA0076
7562
6581
Sb49
Sb06
2470
Sb51
11945
8094
7109
12124
14862
5090
9573
90001/CHAP3A
4486
5139
5970
7400
PA0067
12739
7602
456
7414
6105
8339b
6340
8748
15913
4162
1045
10965
Sb66
12347
PA0011
15328/PgMYB6
6677
13347
15817
CAD
9665f
9163
PAXY225
1
2
3
4
5
6
7
8
9
10
11
12
